# Orally Bioavailable Cyclin A/B RxL Inhibitors: Optimization of a Novel Class of Macrocyclic Peptides That Target E2F-High and G1–S-Checkpoint-Compromised Cancers

**DOI:** 10.1021/acs.jmedchem.5c02445

**Published:** 2026-02-21

**Authors:** Justin A. Shapiro, Nathan J. Dupper, Breena Fraga-Walton, Andrew T. Bockus, Siegfried S.F. Leung, Kai Yang, Chinmay Bhatt, Megan K. DeMart, Miguel P. Baldomero, Luis Hernandez, Gabriel Fung, Sammy Metobo, Steven Xie, Bryan M. Lent, David C. Spellmeyer, Joshua Luna, Dalena Hoang, Manesh Chand, Yuliana Gritsenko, Catherine E. Gleason, Frances Hamkins-Indik, Jie Zheng, Ranya Odeh, Meisam Nosrati, Daphne He, Ramesh Bambal, Peadar Cremin, Jinshu Fang, Bernard Levin, Evelyn W. Wang, Marie Evangelista, David Earp, Constantine Kreatsoulas, Rajinder Singh, Pablo D. Garcia, James B. Aggen

**Affiliations:** 692489 Circle Pharma, 169 Harbor Way, South San Francisco, California 94080, United States

## Abstract

Cyclins A and B bind and activate their cognate cyclin-dependent kinase (CDK) to regulate progression through the S and G2/M phases of the cell cycle, respectively. Cyclins recruit substrates and regulators through the binding of an RxL motif with a Hydrophobic Patch (HP) on the cyclin surface. We recently disclosed the first class of passively permeable macrocyclic peptides that bind to the HP of both Cyclin A and Cyclin B and selectively kill cancer cells with high E2F activity. We used a lead example to demonstrate *in vivo* tumor regression in cell-line-derived xenograft models of small-cell lung cancer (SCLC) via intraperitoneal dosing. Here we describe the optimization of this series for drug-like properties and oral bioavailability, resulting in the discovery of a lead compound, which demonstrates tumor regression in CDX models of SCLC via oral dosing. We are currently evaluating Cyclin A/B inhibition in a Phase 1 clinical trial.

## Graphical abstract

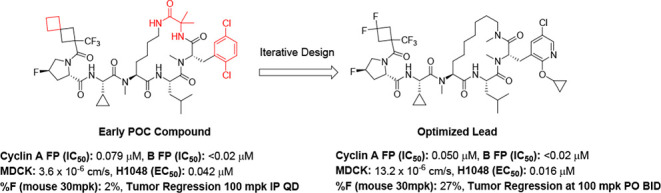

## Introduction

### Oral Peptide Drug Discovery for Intracellular PPIs

The inhibition of protein–protein interactions (PPIs) has seen increased focus in therapeutic discovery and development campaigns in recent years.^1^ Despite increased interest, many PPIs have historically been considered undruggable by small molecules due to the nature of their binding surfaces. Traditional drug targets typically consist of pockets that are small, three-dimensional, partially or completely buried inside a protein, and/or have catalytic activity. These targets are readily occupied by small molecules that follow Lipinski’s rules of drug-likeness, such as low molecular weight and rotatable bond count. By contrast, small drug-like molecules are ill-suited to disrupt the large, flat, nonpolar surfaces through which proteins associate to form complexes.^2^ Many PPIs are critical to the maintenance of biological functions and are at the heart of many disease pathologies, and so medicinal chemists increasingly search beyond the rule of five (bRo5) chemical space for molecules that can “drug the undruggable” to modulate these sites.

A modality that has shown great promise in PPI inhibitor development has been macrocyclic peptides.^3,4^ Because they share intrinsic structural and architectural features with larger proteins (both being polymers of amino acids linked through amide backbones), they can more readily mimic the geometric arrangements of the target’s natural substrates.^4^ They are more resistant to enzymatic degradation than their linear peptide counterparts^5^ while also having fewer degrees of freedom, which can allow for potency enhancements by biasing them toward an active conformation.^6^ Because they tend to rely on a diffuse array of weak binding interactions as opposed to just a few extremely strong binding interactions, they are highly target-specific and generally well-tolerated.^4^ Due to their modular nature, they are readily optimized and amenable to high-throughput synthetic techniques and library generation platforms.^7^ Despite these advantages, many challenges remain with the development of macrocyclic peptide therapeutics.

Macrocyclic peptide PPI inhibitors fall into four categories: injectables targeting extracellular proteins, injectables targeting intracellular proteins, orally delivered compounds targeting extracellular proteins, and orally delivered compounds targeting intracellular proteins^8^ (Figure 1). Many such compounds are highly polar and can neither cross the cell membrane nor the gut wall and thus fall into the first category (e.g., Motixafortide).^9^ Macrocycles that can cross the cell membrane to reach their target, either by passive permeation or by active transport, but are not absorbed into systemic circulation from the gut can be administered by injection (e.g., Sulanemadlin).^10^ Inhibitors of proteins on the cell surface do not require intrinsic permeability and can therefore be administered orally with the help of enabling formulations containing permeation enhancers and picomolar affinity (e.g., Enlicitide).^11^ Reported examples of macrocyclic peptides that inhibit intracellular PPIs and can be administered orally are few and largely limited to previously drugged targets (e.g., Paluratide).^12^

**Figure 1:**
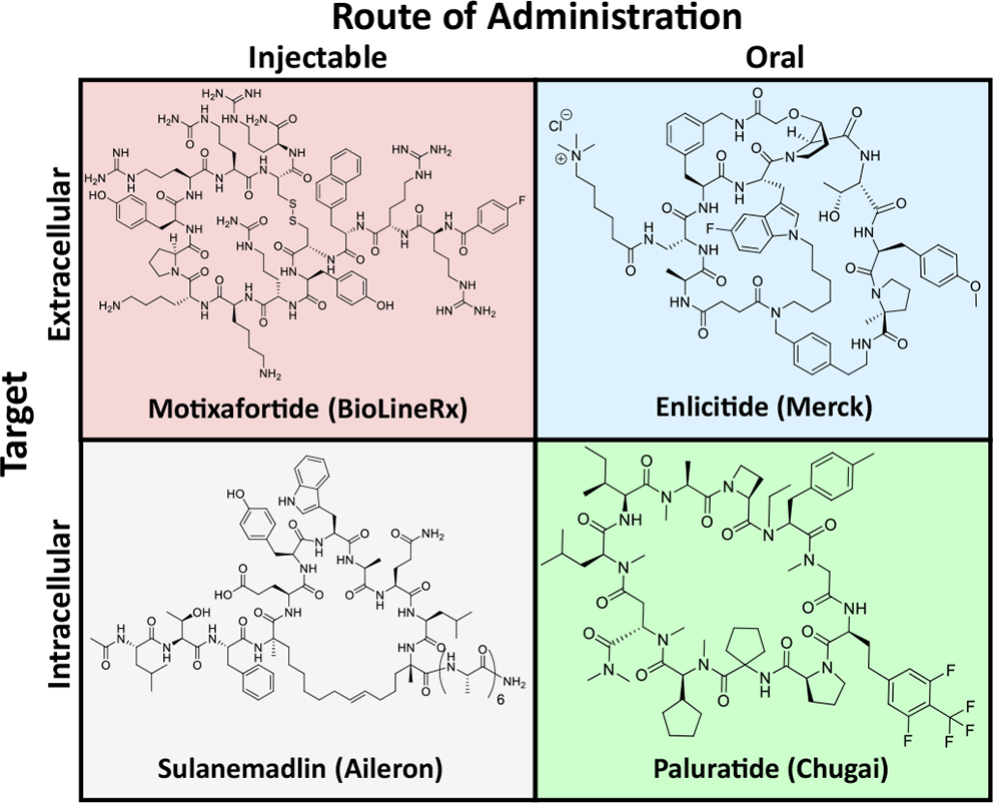
Representative macrocyclic peptide therapeutics organized by route of administration and location of molecular target. (Top left) Motixafortide, an injectable drug with an extracellular target. (Bottom left) Sulanemadlin, an injectable drug with an intracellular target. (Top right) Enlicitide, an oral drug with an extracellular target. (Bottom right) Paluratide, an oral drug with an intracellular target.

Recent publications on passively permeable and orally bioavailable peptide macrocycles emphasize the importance of property-based drug design principles.^12,13^ Removal of all charged or highly polar residues, biasing toward lipophilic character, and a high proportion of backbone *N*-alkylation are all correlated with higher drug-likeness. Critically, peptides that are too large are unlikely to be permeable/oral, but reducing size too much is also detrimental, presumably due to a loss of conformational flexibility (i.e., “chameleonicity”).^14^ Total polar surface area (TPSA), H-bond donor/acceptor count (HBD/HBA), and number of rotatable bonds (#RotB) have also been identified as important properties to track in the bRo5 space.^15–19^ These lessons provide powerful examples to extend the principles of oral peptide drug discovery to previously undrugged intracellular mechanisms.

### Cyclins as a Drug Target

The process by which a cell grows, duplicates DNA, and divides in two (hereafter the cell cycle) is divided into different phases (G1, S, G2, M) during which different critical activities are orchestrated by distinct Cyclin/CDK complexes at each phase (Figure 2A).^20^ In a healthy cell, the orderly transition between these phases, activities within each phase, and critical checkpoints are tightly regulated by phosphorylation events catalyzed by cyclin-dependent kinases (CDKs), which are themselves activated by their cognate cyclins.^21–24^ While the cyclin family is diverse in structure and function,^25^ in the context of the cell cycle they serve two key roles: (1) binding to the CDK causes a conformational change in the kinase necessary for its catalytic activity,^26^ and (2) the cyclin recruits protein substrates and regulators, such as Rb and E2F (Figure 2A), through PPIs between their RxL motif with the Hydrophobic Patch (HP) on cyclins (Figure 2B).^27^ Following these two events, the phosphorylation site of such substrates can access the catalytic binding pocket of the CDK, enabling phosphorylation to occur. Direct inhibition of the catalytic site of CDKs is a validated strategy for the treatment of various cancers,^28^ and small-molecule inhibitors with varying selectivity profiles are in clinical trials.^29,30^ Although a valuable approach, high structural homology between kinase binding pockets can lead to off-target toxicity^31^ and frequently lead to the development of resistance.^32^ For these reasons, alternative mechanisms for modulating Cyclin/CDK complexes are highly attractive.

**Figure 2:**
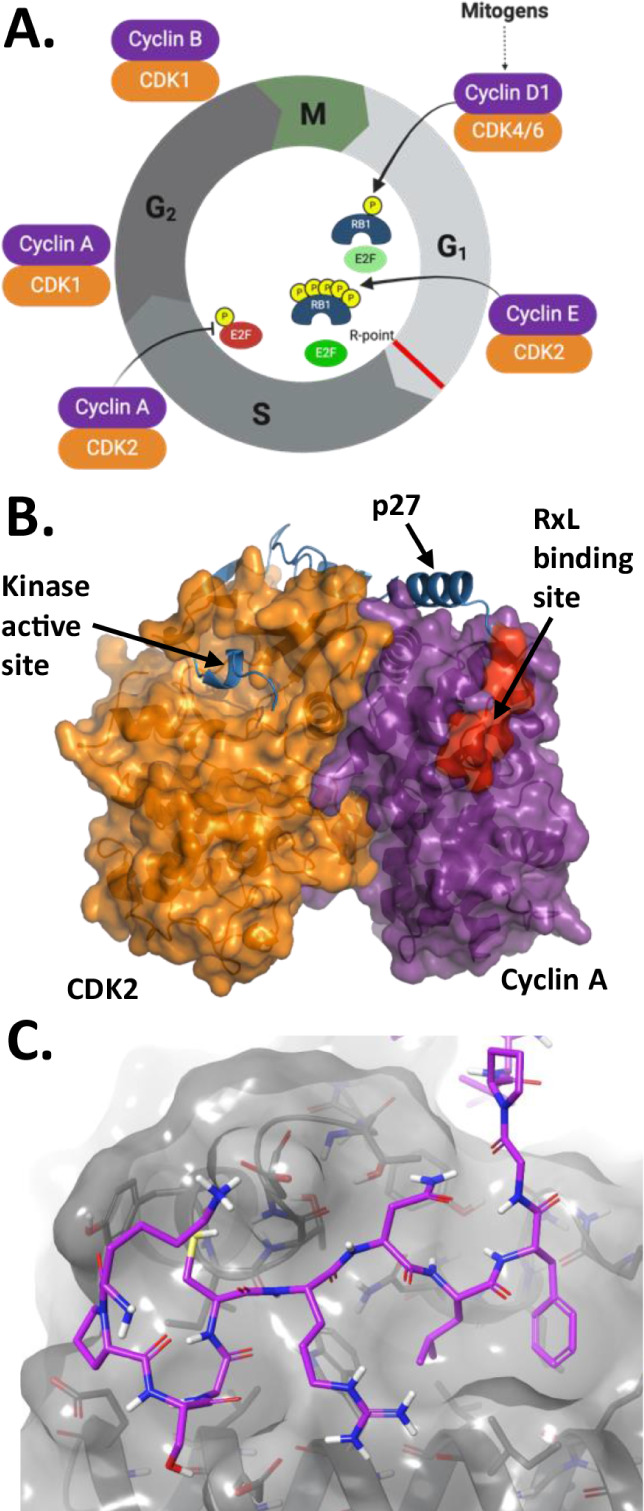
(A) Representation of the cell cycle as regulated by phosphorylation of retinoblastoma (Rb) and early region 2 binding factor (E2F) by CDK/Cyclin complexes. (B) Crystal structure of regulatory peptide p27 bound to the CDK2/Cyclin A complex (PDB: 1JSU). RxL motif of substrate bound to the hydrophobic patch is highlighted in red. (C) Crystal structure of cyclin A RxL binding site with endogenous ligand p27 shown in purple (PDB: 1JSU).

Kaelin and coworkers first demonstrated the therapeutic potential of inhibiting the interaction of RxL-containing substrates with the Cyclin A/CDK2 complex (Figure 2C), using peptides based on the RxL of p27 conjugated to the cell-penetrating TAT peptide.^33^ They showed selective lethality in cells with high levels of E2F activity due to retinoblastoma (Rb) dysregulation, while having minimal effect on nontransformed fibroblasts.^33^ Attempts to develop peptides or small molecules inhibiting the RxL binding site have been historically hampered by weak binding and a reliance on conserved positively charged groups to form salt bridges with the target, thwarting permeability and cellular inhibition.^34–36^ Some efforts to design small molecule inhibitors of the Cyclin A RxL binding site that adhere to Lipinski’s rules have been described. However, these compounds did not display cellular activity, and no further development of these series has been reported.^37^

We recently disclosed a series of passively permeable macrocyclic peptides that mimic the RxL substrate motif and tightly bind Cyclins A, B, and E, or combinations thereof. Using such tool compounds with differential Cyclin selectivity profiles, we showed: (1) significant antiproliferative effects with dual Cyclin A/B RxL inhibitors in E2F-high small-cell lung cancer cell lines, (2) activity in Cyclin E was not required, and (3) selective Cyclin A or Cyclin B inhibitors had a modest effect on proliferation.^38^ We developed a mechanistic model for the dual Cyclin A/B RxL inhibition-driven effects in E2F-high cancers, as represented in Figure 3. Briefly, the combined effects from Cyclin A RxL inhibition (replication stress, DNA damage) and Cyclin B RxL inhibition (displacement of Myt1 resulting in Cyclin B/CDK complex activation) trigger hyperactivation of the spindle-assembly checkpoint (SAC), ultimately leading to apoptotic cell death selectively in cancers with G1/S-compromised checkpoints and E2F pathway-high signatures. These effects are not observed in nontransformed fibroblasts or primary human progenitor stem cells,^38^ suggesting a large therapeutic window. Indeed, these tool Cyclin A/B RxL inhibitors were well tolerated and efficacious *in vivo* in several cell-line-derived xenograft (CDX) mouse models when administered via intraperitoneal^39^ or intravenous^38^ dosing.

**Figure 3:**
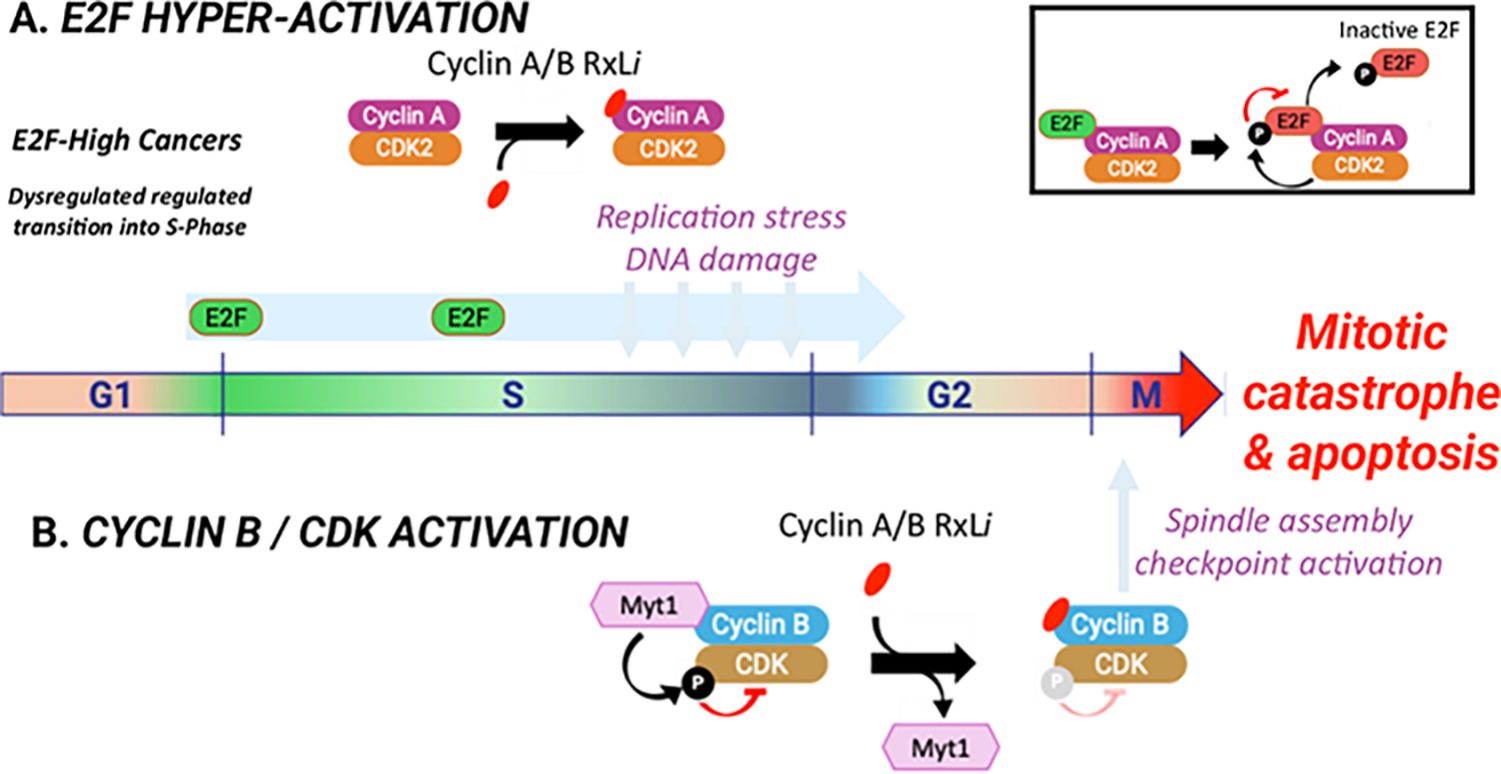
Model for the mechanism of action of Cyclin A/B RxL inhibitors. Cancers with dysregulated G1/S checkpoints (e.g., Rb loss) have high levels of E2F activity, which leads to replication stress and DNA damage during the S-phase, making them dependent on later checkpoints of the cell cycle to proliferate. For example, the S/G2 transition requires attenuation of E2F activity for cells to complete the S-phase. This process depends on the RxL motif of E2F interacting with Cyclin A, for CDK2 to phosphorylate and inactivate E2F (inset top right). Cyclin A/B RxL inhibitors block this interaction, leading to hyperactivity of E2F throughout S-phase and into G2, thus further increasing replication stress, DNA damage, and dysregulated S/G2 transition (Panel A). In G2/M, the Cyclin A/B RxL inhibitor blocks the kinase Myt1 RxL-dependent interaction with the Cyclin B/CDK complex. Myt1 phosphorylates CDK, which results in CDK kinase activity inhibition. Thus, blocking the Myt1 interaction results in the activation of the Cyclin B/CDK complex (Panel B). The combination of hyperactive E2F, replication stress, DNA damage, and activation of Cyclin B/CDK leads to persistent spindle assembly checkpoint activity, blocks progression at G2/M, and ultimately results in mitotic catastrophe and cell death by apoptosis in E2F-high cancers.

Our larger goal was to identify a compound from this series suitable for advancement into clinical investigation as an orally delivered drug candidate. While the lead Compound **2** (Compound 34 in ref. 39) (Figure 4) demonstrated encouraging properties and the first *in vivo* proof-of-concept efficacy for this mechanism of action, its low oral bioavailability (2%) highlighted a key shortcoming, which we aimed to address in the subsequent optimization campaign. Based on our observation that achieving free-drug concentration in plasma above the GI_50_ for a few hours resulted in tumor regressions, we were able to set a benchmark for exposure during our campaign to develop oral inhibitors. In order to provide focus for our optimization efforts, we contrasted the characteristics of Compound **2** to the reported physicochemical property space permissive of oral bioavailability relevant to macrocyclic peptides.^12,13,16,17,19^ While some properties of **2** fell within the permissive space, such as TPSA (177) and #RotB (10), some were less optimal, including LogD (5.54) and the HBD count (4), which might be limiting its observed bioavailability.^12,13,16,17,19^ With these findings in mind, we began our lead optimization campaign to develop an orally bioavailable peptide macrocycle inhibitor of Cyclin A/B. This required that we first improve oral bioavailability sufficient to achieve efficacy in a relevant tumor model via oral dosing at reasonable and tolerated dose levels, followed by confirmation of oral absorption across preclinical species. Based on our prior results with the NCI-H69 tumor model, we anticipated oral efficacy would require achieving free drug *C*_max_ levels in excess of the GI_50_. To meet this goal, we focused our optimization on: (i) improving oral bioavailability with a target of ≥20% by modulating compound properties such as TPSA, HBD count, and LogD; (ii) lowering the exposure target by increasing cellular potency, with a target of GI_50_ < 20 nM vs our screening SCLC cell line, and by ensuring that plasma protein binding did not increase to the point where the unbound fraction would become limiting.

**Figure 4:**
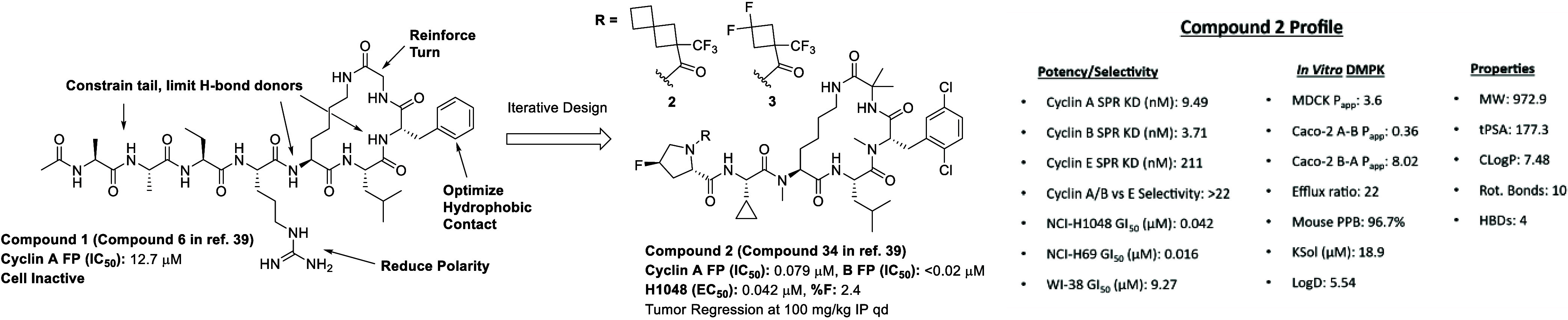
(Left) Summary of hit-to-lead campaign, detailed in ref 39. (Right) Profile of biophysical and cellular potencies, *in vitro* DMPK parameters, and molecular properties of Compound **2**.

## Results

### Identifying Sites for Structural Diversification

While the potential for structural diversity in macrocyclic peptides is very useful during the library generation and hit discovery phases of a campaign, lead optimization can often be a daunting prospect because the number of sites for variation in larger scaffolds multiplies the challenge for optimization relative to that of typical small-molecule scaffolds. Fortunately, in the structure-guided discovery of Compound **2**, we established a robust SAR dataset supported by models derived from published cocrystal structures of Cyclin A. Specifically, we generated binding models based on structures most relevant to our macrocycle series and informative of key interactions at the binding site, including the macrocycle-bound structure (PDB: 1URC) and the linear peptide-bound structure (PDB: 1JSU).^39^ These models were successfully applied in our structure-based design efforts to improve both potency and permeability. Leveraging insights from the historical SAR, we identified three high-value regions of the scaffold on which to focus (Figure 5a).

**Figure 5:**
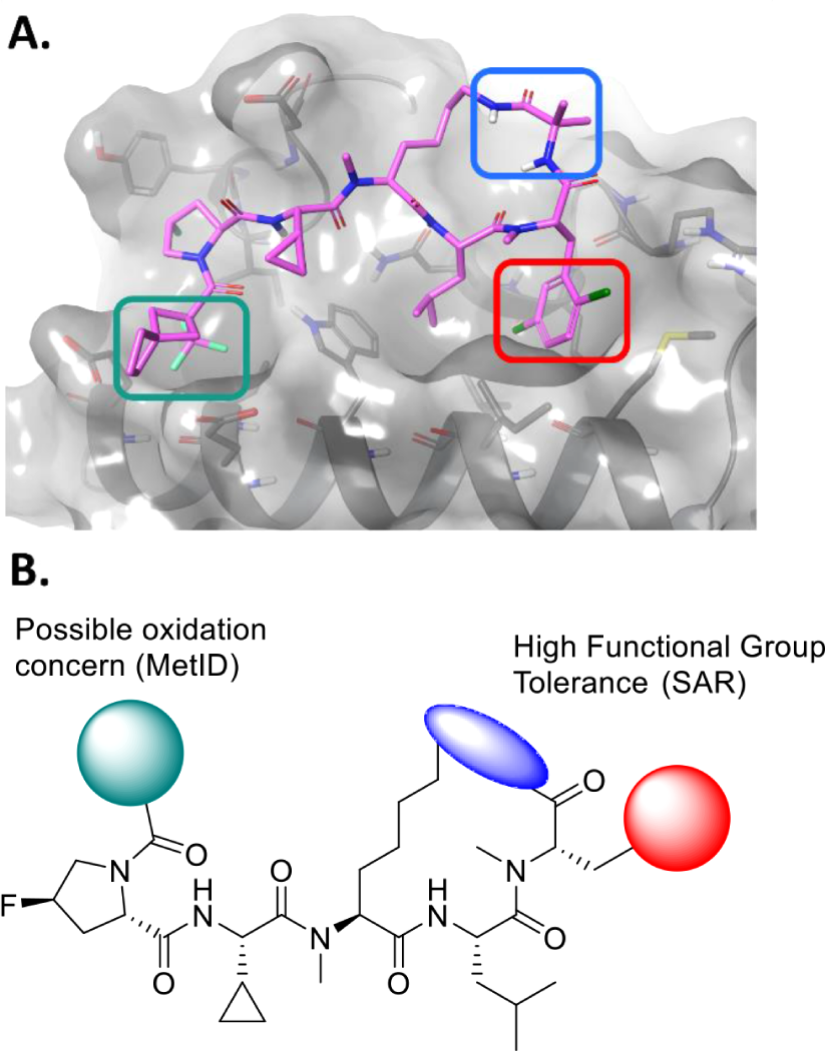
(A) Computational model of Compound **2** in the RxL binding site of Cyclin A, with sites of interest for lead optimization highlighted. (B) Summary of the lead optimization strategy. Historical SAR^39^ indicates a diverse range of functional groups tolerated on the phenylalanine residue (red) and the bridging residue (blue). The *N*-terminal capping motif (green) was identified as a potential metabolic liability, and so alternative caps were also considered.

#### Phenylalanine Residue Ortho-Position

During the hit-to-lead campaign, we derived significant benefit by first monochlorinating the phenylalanine at the meta-position, which provided improved potency by burying a lipophilic substituent into a deep pocket. Later we discovered that further 2,5-dichlorination was also tolerated, as the *ortho*-chloro rested in a partially exposed shelf based on molecular modeling (Figure 5a, highlighted in red) and conferred improvements in PK parameters. Although not disclosed previously, we discovered that this position was broadly tolerant of a variety of substituents. We hypothesized that the 2-position of Phe could be a synthetic handle by which we could modulate physicochemical properties.

#### Bridge from Lariat to Phenylalanine Residue

We also noted that the residue bridging the Nε-position of the lysine lariat and the C-terminus of the phenylalanine (2-Aminoisobutyric acid in Compound **2**) could be replaced with a variety of residues without significant detrimental effects on biochemical or cellular activity, as long as the residue’s side chain was not highly polar and the residue was either a *D*-stereoisomer or alpha-disubstituted. Our docking model indicated that this portion of the lariat was not involved in any key interactions with the Cyclin A binding pocket but was likely involved somehow in the preorganization of the ligand (Figure 5a, highlighted in blue).

Interestingly, replacement of this position with *D*-proline counterintuitively reduced passive permeability as measured by MDCK, even though this side chain reduces the HBD count by one (Supplementary Figure S1). Because proline enforces conformational rigidity, this may indicate that the macrocycle flexibility in this region is important for permeability. In contrast to the proline analog mentioned above, an early matched pair, **4** (Compound 13 in ref. 39) and **6** (Table 1), indicated that removal of the Nε-H is beneficial to permeability and, in this case, does not negatively impact potency. However, as we highlighted in our prior report,^39^ *N*-methylation at this position was not consistently tolerated from a potency perspective. It is also noteworthy that an ester linkage (**5**) in place of the amide improves the permeability to the same degree as *N*-methylation. Based on the molecular modeling and the encouraging trends in MDCK for this early SAR series, we highlighted the region from Nε-lysine to the N-terminus of the bridging residue as the second major site for diversification (Figure 5b, highlighted in blue).

**Table 1:**
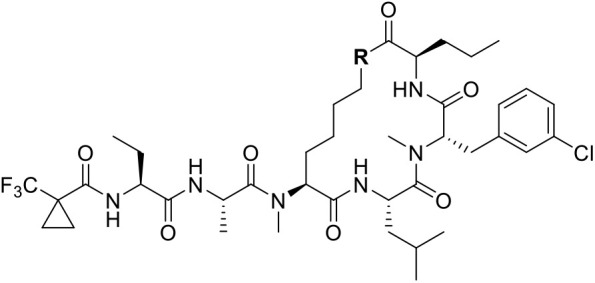
Replacement of the Lariat Amide with Ester or *N*-Methyl

ID	R	Cyclin A FP-2 IC_50_(μM)	MDCK Papp × 10^–6^ (cm/s)
4	–NH–	2.843	0.5
5	–O–	4.442	6.9
6	–NMe–	1.277	7.2

#### N-Terminal Acid Cap

Finally, while Compound **2** was acceptable for an IP proof-of-concept efficacy demonstration, we wanted to continue addressing potential metabolic liabilities that might limit oral exposure. We noted that the highly lipophilic spirocyclic N-terminal capping portion of the tail was a site of oxidation (see Supporting Information), and while not an extreme concern, we were aware that it could get worse as the overall properties of the scaffold were modified. From the hit-to-lead campaign, we identified other capping groups of interest that we could substitute should we encounter unanticipated problems with the spirocycle. Specifically, Compound **3** (Compound 32 in ref. 39) (Figure 4) showed similar *in vivo* PK, improved solubility, and would theoretically be more resistant to alkyl oxidation due to the presence of the terminal difluoromethylene group. Knowing that the tail species may need further optimization led to our choice of this region as our final diversification site (Figure 5b, highlighted in green).

With these three high-value regions of the scaffold identified (2-Phe substituent, bridging residue from lariat to Phe, capping group), we proceeded with our structure- and property-based lead optimization campaign to generate orally bioavailable macrocyclic inhibitors of the Cyclin A/B RxL binding site for the treatment of E2F-high cancers.

### Diversification of 2-Phe Substituents on Compound 2 Scaffold

Substituents ranging from alkyl groups (**7**), ethers (**8)**, heterobiaryls (**9** and **11**), and amines (**10**) were selected based on predicted tolerability in molecular modeling (Table 2). All were generally well tolerated in a biochemical FP assay and in an antiproliferative cellular assay against small-cell lung cancer line NCI-H1048, in line with expectations and historical SAR. Additionally, these compounds maintained high selectivity between the Rb-dysregulated cell line NCI-H1048 and our negative control WI-38 nontransformed fibroblast cell line (Supplementary Table S5). While the spread of values is not large enough to draw definitive conclusions, there appears to be a slight trend of smaller volume substituents correlating with more potent cellular activity. Unfortunately, all modifications sampled reduced the MDCK permeability, suggesting that they are unlikely to increase oral exposure. Unexpectedly, matched pairs **12** and **13**, which differ only by a carbon-to-nitrogen substitution at the 3-position of the phenyl ring, are differentiated in cellular activity and permeability despite having equivalent biochemical activity. Despite an increase in TPSA, the pyridone-ether appears to confer a special advantage. Although all modifications in this series showed reduced MDCK values relative to Compound **2**, the broad tolerability was encouraging because it hinted that we could significantly alter the 2-position of the phenylalanine in the hopes of improving other pharmacokinetic parameters of our inhibitors without a loss of biochemical/cellular potency. While none of these compounds themselves were selected for further *in vitro* or *in vivo* PK profiling, the structure–activity relationship was noted for future exploration in more advanced iterations of the scaffold.

**Table 2:**
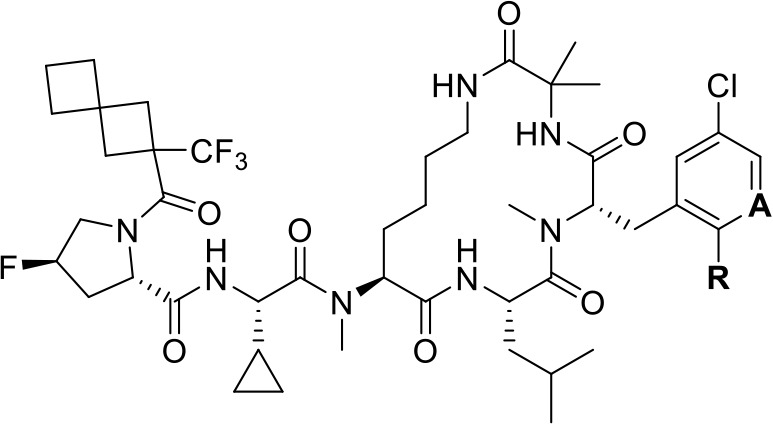

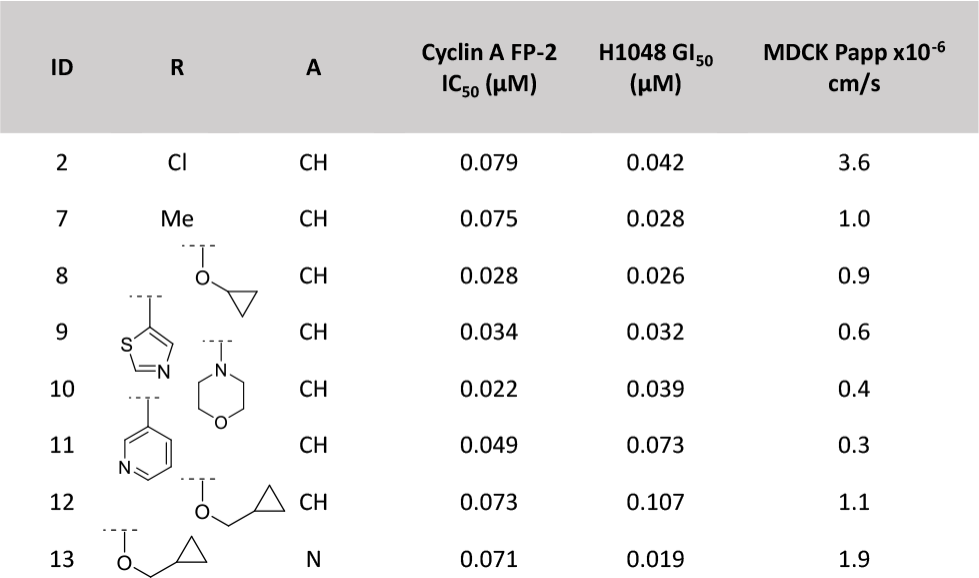
Diversification of Phenylalanine Residue of Compound 2

### Methylation of the Nε-Position of the Lariat Lysine

Based on observations from modeling and early SAR that the Nε-position of the lariat lysine does not appear to participate in an H-bonding interaction with the Cyclin A/B RxL binding sites, we synthesized an *N*-methyl analogue of Compound 2 (Table 3). To our surprise, the *N*-methylated analog **14** lost over 10-fold biochemical potency and showed lower MDCK permeability. To assess if this phenomenon was general or isolated to this compound, we synthesized a closely related matched pair, **15**/**16**. While the effect was not as stark in this case, there was still a notable 2-fold loss of potency in both biochemical and cellular assays and no improvement in MDCK. Noting that Compound **2** had relatively low KSol and the introduction of this methylation marker has been associated with solubility decreases, we hypothesized that we were hitting a threshold of solubility where compounds were precipitating out of assay media and interfering with accurate evaluation. Given the improved KSol from Compound **2** (18.9 μM) to Compound **3** (109 μM),^39^ we decided to synthesize a nonmethylated/methylated matched pair in the context of the difluorocyclobutyl capping group. Gratifyingly, **18** maintained biochemical and cellular potency and showed a 5-fold improvement in MDCK when compared to **17**. Based on these results, we continued to utilize both the spiro-cap and the difluorocyclobutyl-cap as SAR continued.

**Table 3:**
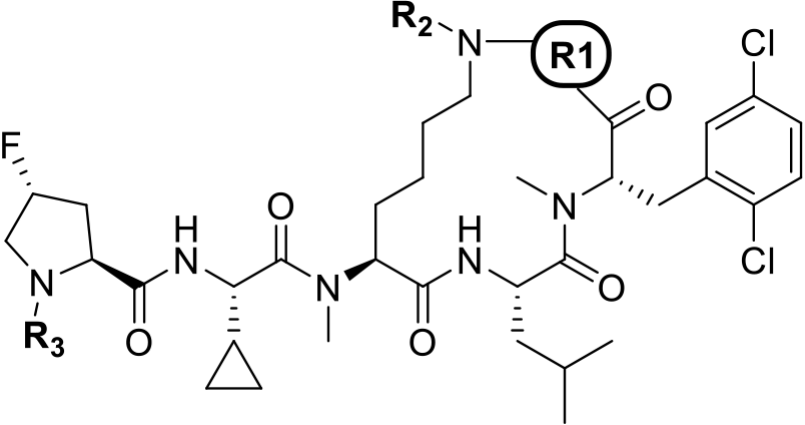

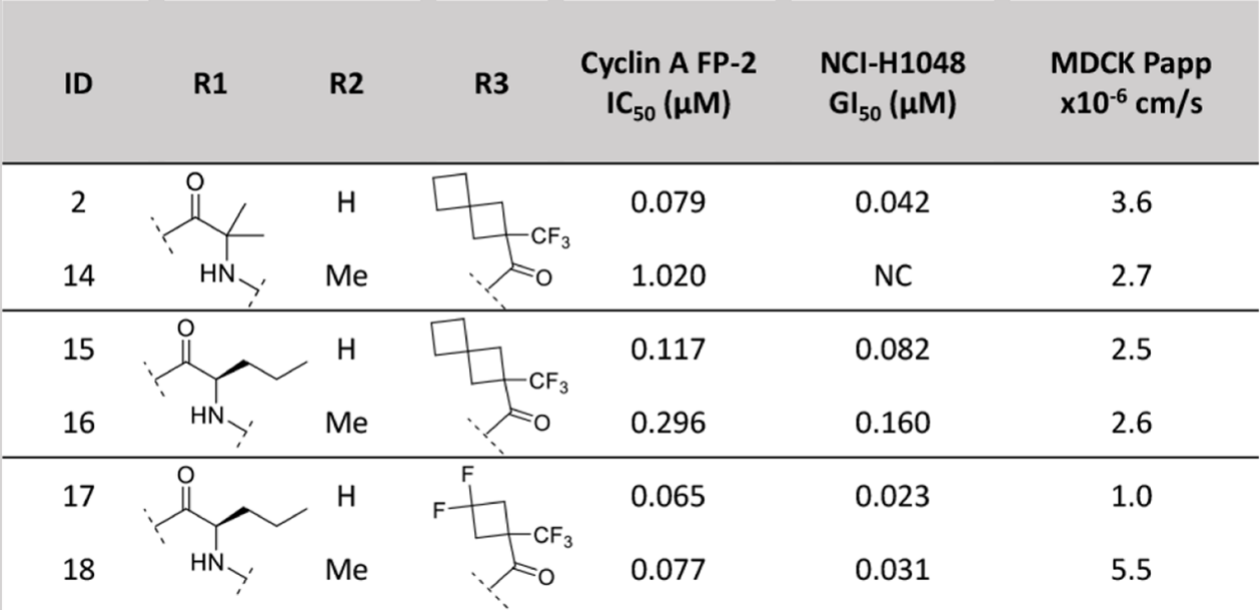
Methylation of the Lariat Nitrogen

### Replacement of Bridging Residue with Extended Alkyl Chain and Methylation of Extended Lariat

Based on encouraging results from Nε-position lysine lariat methylation, we wanted to remove additional heteroatoms from the macrocycle while still maintaining the same macrocyclic ring size. To achieve this, we required an alternative synthetic route to our standard SPPS lariat scheme. We conceptualized two potential alternative synthetic routes: (1) solid-phase peptide synthesis in which custom-synthesized extended lysine analogs are cyclized with the C-terminus of the phenylalanine position via lactamization (Scheme 1, left) or (2) a solution-phase approach in which a C-terminal olefin is cyclized with a lariat olefin via a ring-closing metathesis reaction (Scheme 1, right). While the advantage of SPPS is clear in library synthesis and early discovery, as the lead optimization campaign progressed and increasing quantities of material were required, we were interested in developing robust synthetic methods with lower usage of precious custom building blocks, higher yield, and an improved ability to generate larger quantities of intermediates which could be modified by late-stage derivatization. Furthermore, methods to synthesize extended lysine intermediates were low-yielding, cumbersome, and had long lead times. Finally, the phenylalanine derivatives were problematic in terms of resin loading, challenging subsequent couplings/methylations, and epimerization during the final macrocyclization step. For these reasons, we chose to pursue a solution-phase synthetic route based on an RCM macrocyclization followed by late-stage diversification.

**Scheme 1:**
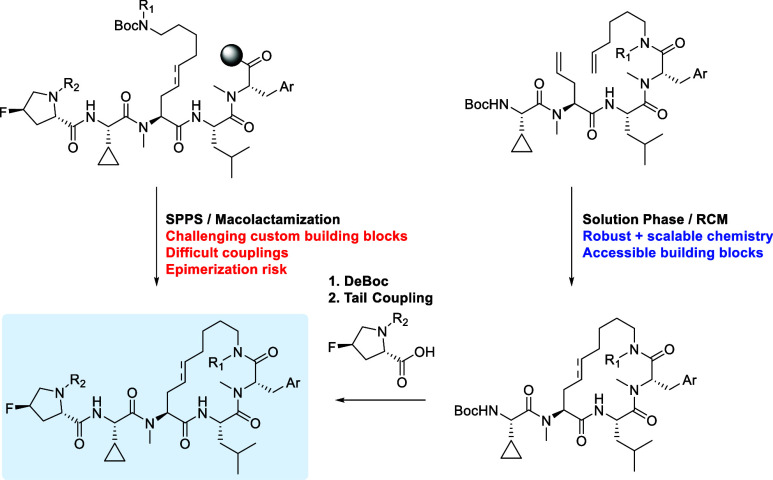
Comparison of Synthetic Strategies for “Extended Lariat” Compounds

Comparison of matched pairs **19** and **20** shows a substantial advantage of the difluorocyclobutyl-cap over the spiro-cap in the context of a fully alkyl replacement for the bridging residue in both biochemical and cellular assays (Table 4). This is consistent with **16/18**, in which the lariat was made more lipophilic by the alkylation of the Nε-position of the extended lysine lariat and, thus, the removal of an H-bond donor. This trend is continued, albeit less dramatically, when the scaffold is further methylated in the case of **21** and **22**, where the difluorocyclobutyl-cap is again more active. Furthermore, in this case, MDCK is improved more than 2-fold over the spiro-cap. To evaluate the generality of this trend, we conducted a database-wide analysis on what effect changing a spiro-cap to a difluorocyclobutyl-cap (Figure 6), with all else being equal, has on the binding affinity, cellular inhibition, and MDCK permeability. When a bridging residue is present and the Nε-position of the lariat nitrogen is not methylated, biochemical potency is generally held steady or improved, but no clear relationship is observed for cellular potency or MDCK (blue dots). However, when a bridging residue is present and the Nε-position of the lysine lariat is methylated (orange dots) or when the bridging residue is replaced with an extended alkyl chain (purple dots), a clear advantage across all three parameters can be observed.

**Table 4:**
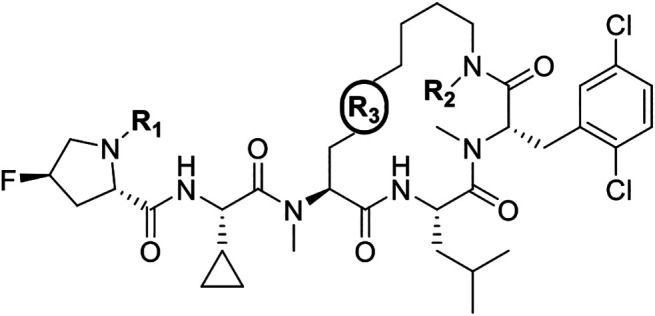

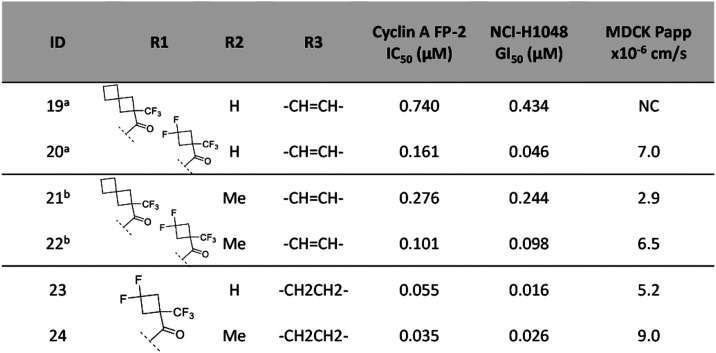
Initial Extended Lariat Compounds^a^,^b^

a Compound was tested as a mixture of *E/Z* isomers (∼9:1), major isomer identity not elucidated.

b Alkene isomer identity was not elucidated.

**Figure 6:**
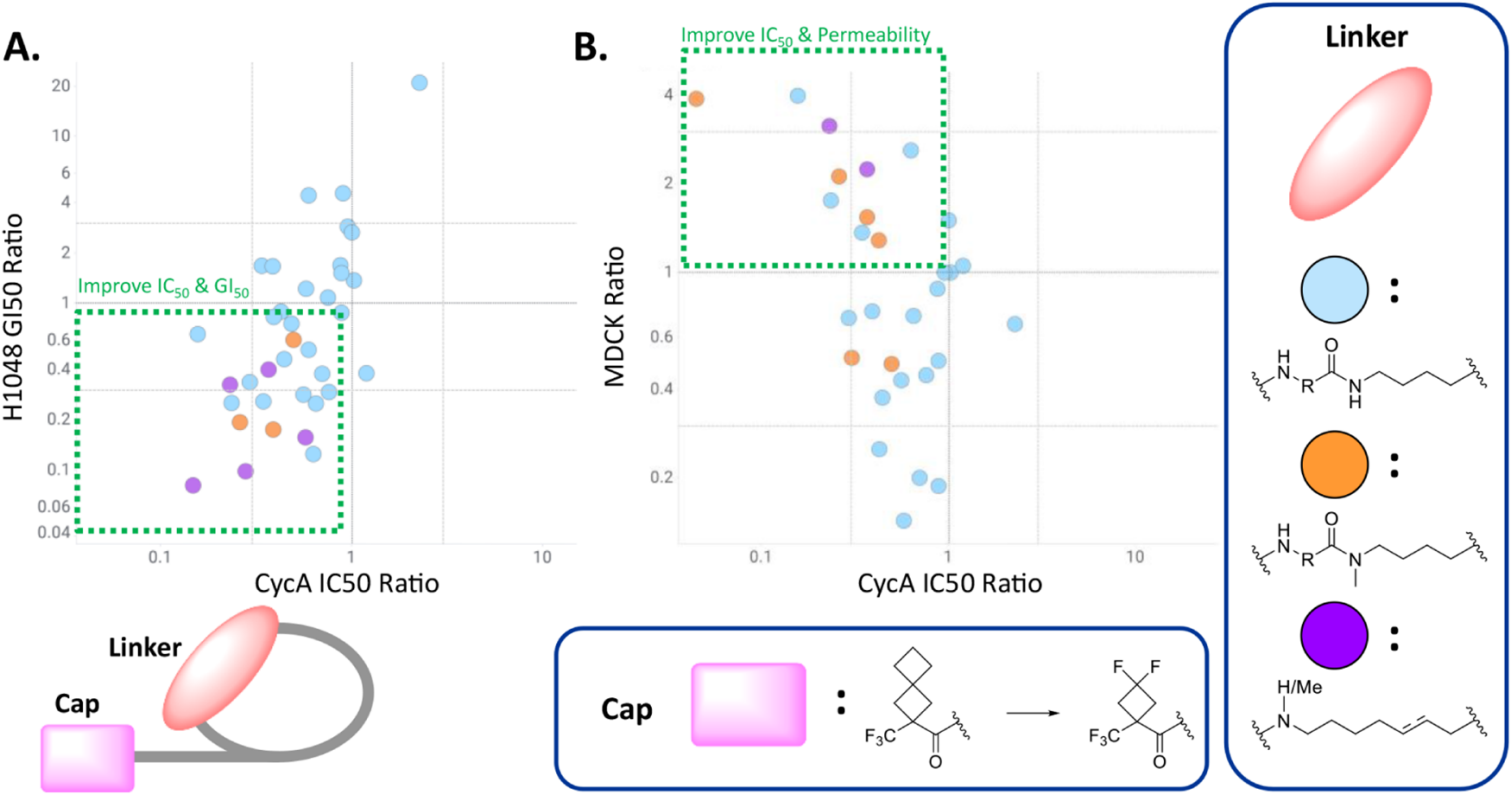
Database-wide matched-pair analysis of the effects of swapping from a spirocyclic-cap to a difluorocyclobutyl-cap. Each point represents one matched pair. Compounds containing a bridging residue between the lariat and the phenylalanine and not containing a methylation on the lariat amide are represented by blue dots. Compounds containing a bridging residue and a methylation on the lariat amide are represented by orange dots. Compounds containing an extended lariat in place of a bridging residue are represented by purple dots. (A) Correlation plot between ratios of biochemical activity in Cyclin A FP binding assay (*y*-axis) to ratios of antiproliferative activity in NCI-H1048 cell culture (*x*-axis). (B) Correlation plot between ratios of biochemical activity in Cyclin A FP binding assay (*y*-axis) to ratios of permeability as measured by MDCK (*x*-axis).

Mixtures of E and Z isomeric products were obtained from the RCM reaction, possibly due to the flexibility of the linear starting material. Reaction mixtures showed a ratio of roughly 3:1 for the unmethylated Nε-position lariat (**19** and **20**), which could be further purified to ∼9:1. However, the methylated Nε-position lariat (**21** and **22**) was significantly more selective (>10:1). For these early RCM-based alkene-containing compounds, complete separation proved challenging, and they were tested as mixtures. For this reason, assigning E/Z ratios was not undertaken. Thankfully, hydrogenation of this double bond to the saturated alkyl chain from **20** to **23** and **22** to **24** (Table 4) provided a potency advantage in biochemical and cellular assays in both cases as well as avoided the synthetic challenge of selectively synthesizing or separating E/Z isomers. Both compounds display impressive MDCK, with **24** having the best potency/permeability profile that we had seen in our campaign to this point. Encouraged, we decided to profile these compounds further.

### PK Profiling of Extended + Methylated Lariat Scaffold

To determine where our compounds stood in comparison to our starting points (Compounds **2** and **3**) and determine where we still needed to improve, we evaluated calculated parameters, *in vitro* ADME properties, and mouse PK readouts (Table 5). We significantly reduced TPSA and increased CLogP in both **22** and **24**. Although this appears to help increase MDCK permeability, it comes at the cost of dramatically reduced solubility. Furthermore, this class of compounds displays high plasma protein binding, which may limit free-drug exposure above the GI_50_. We noted some encouraging signals from *in vivo* PK; **22** has similar IV clearance, and **24** has reduced IV clearance when compared to Compounds **2** and **3**. Furthermore, **22** shows 10% oral bioavailability at 10 mpk, which is a dramatic improvement over 2% at 30 mpk seen in the early lead. While these compounds were not predicted to demonstrate oral efficacy based on free-drug exposure, we determined that this was a much-improved scaffold for optimization, and we opted to continue diversification from **24**, given its superior cellular potency and high MDCK permeability.

**Table 5:**
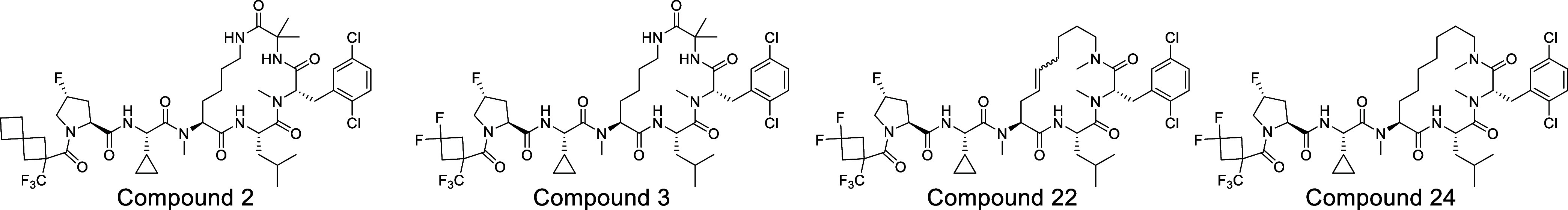
Comparison of Activity and DMPK Parameters of Extended Lariat Compounds **22** and **24** Compared to Original Leads **2** and 3

ID	Cyclin A FP-2 IC_50_ (μM)	NCI-H1048 GI_50_ (μM)	MDCK Papp × 10^–6^ (cm/s)	Ksol (μM)	%PPB	tPSA	cLogP	Mouse Cl (mL/min/kg)(2 mpk IV)	%F (PO)
2	0.079	0.042	3.6	18.9	96.7	177.3	7.48	36.3	2 (30 mpk)
3	0.053	0.025	0.7	106.6	96.7	177.33	6.82	31.21	NC
22	0.101	0.098	6.5	NC	99.0	139.4	7.87	36.4	10 (10 mpk)
24	0.035	0.026	9.0	2.8	99.0	139.4	8.36	14.81	NC

### Revisiting 2-Phe Diversification on 24 Scaffold

We wished to revisit the 2-substituted phenylalanine derivatives exemplified in Table 2 in the context of the promising, more lipophilic macrocyclic core to explore these substituents’ effects on cellular potency and PK parameters. We utilized an enabling Suzuki reaction (Scheme 2) to efficiently diversify a late-stage intermediate. In this way, compounds such as **25–28** were synthesized and evaluated (Table 6).

**Scheme 2:**
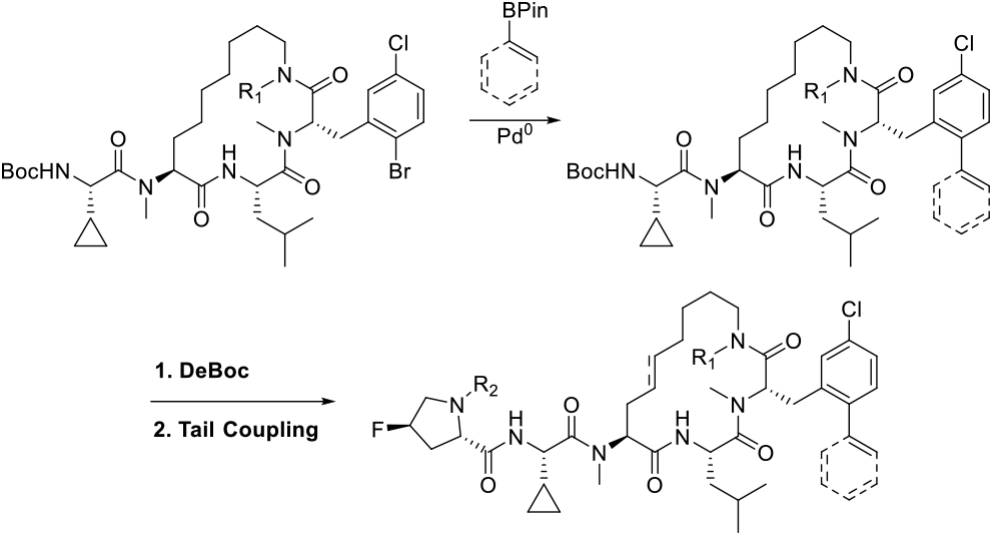
Late-Stage Diversification Strategy for *Ortho*-Position of Phenylalanine on Extended Lariat Scaffolds

**Table 6:**
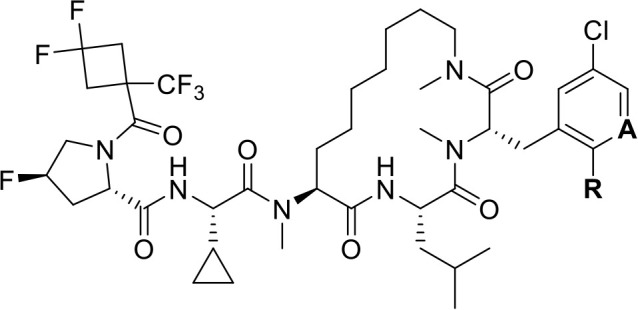

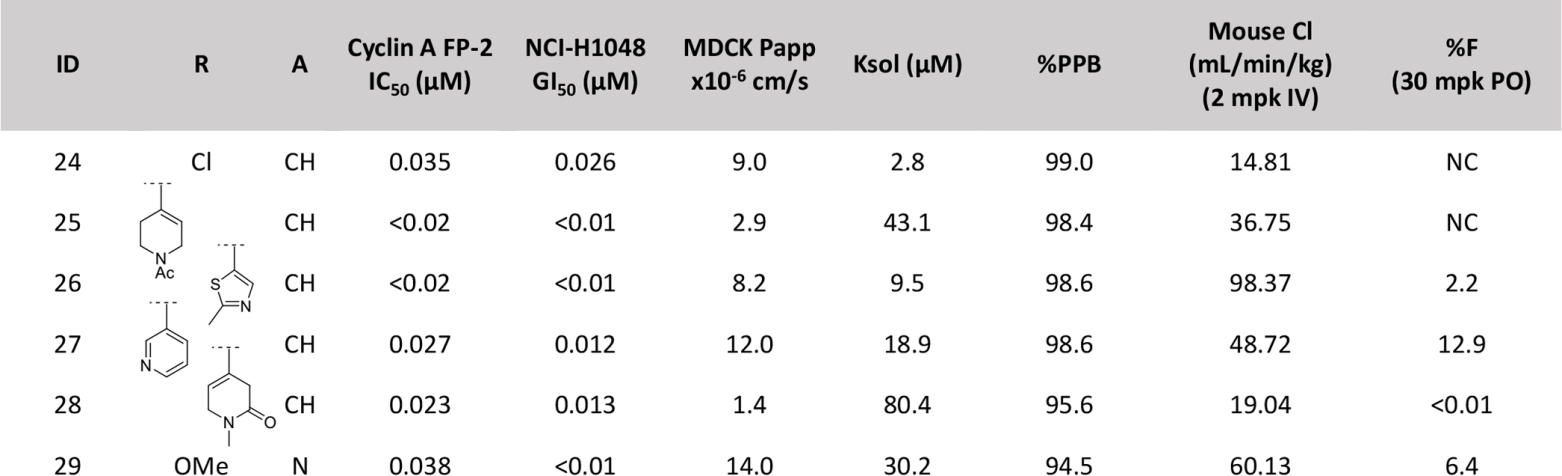
Diversification of Phenylalanine Residue of Compound 24 Leads to Pyridone-Ether 29 with Free *C*_max_ above GI50

Both saturated and unsaturated heterocycles were tolerated at the 2-position of the phenylalanine. There appeared to be a general improvement in both the cellular and biochemical activity, in line with our potency goals for compounds substituted with larger groups at this position. In addition to the potency increases, several groups led to improved permeability and increased solubility. However, plasma protein binding generally remained very high, which limited free drug exposure. This was true even in cases where %F was improved (e.g., **27**). Modestly increasing the polarity through the introduction of a methylated lactam (**28**) did reduce plasma protein binding, but this was at the expense of any measurable oral exposure (likely due to a dramatic reduction in MDCK).

Looking back at the earlier SAR generated on the ortho position of the Phe for the Compound 2 scaffold, we took inspiration from the matched pair between **12** and **13** (Table 2). In this case, the conversion of an ether to a pyridone-ether resulted in an unexpected improvement (given the increased TPSA) in MDCK and was well tolerated in biochemical and cellular potency. With this in mind, we synthesized **29** and were excited to see not only very potent cellular activity but also exceptional permeability combined with a reduced %PPB and improved solubility. While IV clearance was moderate, the compound still displayed 6.4% oral bioavailability and achieved free-drug exposure above GI_50_. Encouraged, we proceeded to optimize this series to develop an analog which met our >20% oral bioavailability goal in preparation for PO efficacy. A novel lipid-based oral formulation system was developed to improve the bioavailability of these bRo5 compounds. The formulation, composed of PEG 400:Solutol HS15:Phosal 53 MCT (30:20:50, v/v/v), was used as the standard vehicle to evaluate oral pharmacokinetics in mice. Unlike conventional cosolvent formulations, this lipid system was designed to overcome the dual challenges of poor solubility and limited permeability by maintaining the compound in a supersaturated, solubilized, and readily absorbable state throughout the gastrointestinal (GI) tract, while also enhancing transcellular diffusion across the intestinal membrane for moderately lipophilic molecules.

### Optimizing Pyridone-Ether Phe Series and Nomination of 33 for EfficacyMinor

We hypothesized that increasing the alkyl group size on the pyridone ether could benefit this series in two ways: first, by blocking metabolic demethylation, and second, by increasing the lipophilic character to aid in passive crossing of the gut wall. Based on the broad substituent tolerance that we had seen in both the extended lariat series (Table 6) and the earlier bridging residue-containing series (Table 2), we anticipated relatively flat SAR as we increased the size of the alkyl ether portion of **29** (Table 7). To our surprise, however, a change from methyl to ethyl in **30** marginally reduces the cellular potency, even though the biochemical potency remains unchanged. Furthermore, increasing the alkyl character in this way does not improve the MDCK as expected and reduces the KSol, so this compound was not profiled further. Increasing steric bulk and lipophilic character further by introducing the branched isopropyl (**31**) or isobutyl (**32**) also negatively impacted cellular potency outside our target of <20 nM and was thus not profiled further. To strike a balance between potency and retained or improved PK parameters, we replaced the methyl ether with a cyclopropyl ether in **33** (CIRc-014 in ref. 38) Gratifyingly, this compound maintains a cellular potency near the bottom of the assay and is nearly unchanged in MDCK, KSol, or %PPB. While the IV clearance of **33** remains moderate/high, it nevertheless displays an oral bioavailability of 27.2% in mice at 30 mg/kg and achieves free-drug exposure above the GI_50_.

**Table 7:**
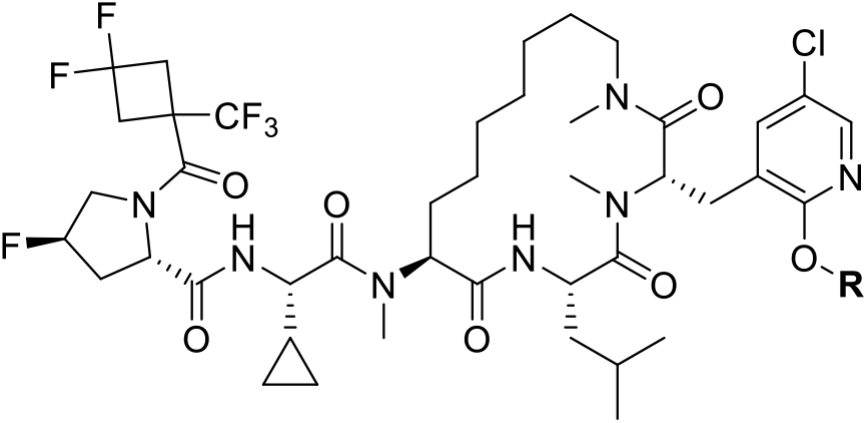
Alkyl Scan of Pyridone-Ethers Leads to Compound **33**, Which Retains Potency and *In Vitro* DMPK While Boosting Oral Bioavailability

ID	R	Cyclin A FP-2 IC_50_ (μM)	NCI-H1048 GI_50_ (μM)	MDCK Papp × 10^–6^ (cm/s)	Ksol (μM)	%PPB	Mouse Cl (mL/min/kg)(2 mpk IV)	%F (30 mpk PO)
29	–Me	0.038	<0.01	14.0	30.2	94.5	60.13	6.4
30	–Et	0.025	0.021	13.4	11.5	95.3	NC	NC
31	–iPr	0.033	0.100	NC	NC	NC	NC	NC
32	–iBu	0.027	0.057	NC	NC	NC	NC	NC
33	–cyPr	0.050	0.015	13.2	27.3	96.6	68.05	27.2

We evaluated the mouse liver microsomal stability at various stages of the SAR campaign. Unfortunately, we found poor *in vitro*–*in vivo* correlation (IVIVC). For instance, representative compounds (**2**, **29**, and **33**) displayed *in vitro* *t*_1/2_ values of 4.66, 4.53, and 12.6 min, corresponding to predicted hepatic clearances of 83.9, 84.2, and 75.6 mL/min/kg and hepatic extraction ratios (*E*_H_) of 0.93, 0.94, and 0.83, respectively (Supplementary Table S6). All three compounds therefore display rapid microsomal turnover; however, these values substantially overpredict the *in vivo* clearance measured after IV dosing in mice (36.3, 60.1, and 68.1 mL/min/kg for Compounds **2**, **29**, and **33**, respectively), indicating poor IVIVC. Microsomal assays capture CYP450-mediated intrinsic metabolism but do not account for permeability or protein-binding effects that constrain clearance *in vivo*.

Compounds **29** and **33** were administered in the same formulation and show comparable IV clearance in mice, kinetic solubility, MDCK passive permeability, and plasma protein binding, yet oral bioavailability differs markedly (6.4% vs 27.2%). Because observed systemic clearance is similar, the higher exposure of Compound **33** may reflect reduced presystemic loss, potentially through lower intestinal metabolism or efflux. The −OMe to O–cyclopropyl substitution could sterically reduce metabolic accessibility in the gut without affecting hepatic clearance. These results illustrate that structural modifications can alter presystemic disposition even when kinetic solubility, passive permeability, or microsomal stability appear similar.

**33** was selected for further profiling in preparation for an *in vivo* efficacy study. A more in-depth analysis of **33** can be seen in Figure 7. Compound **33** binds to Cyclins A and B at 2.7 and 1.0 nM, respectively, in a surface plasmon resonance (SPR) assay, confirming the highly potent activity measured in our screening FP assay. Furthermore, the binding of **33** is >12-fold less potent to Cyclin E, demonstrating selectivity for our desired mechanism of action. At the outset of the lead optimization campaign, we identified HBD count and LogD as properties to monitor and lower to improve oral bioavailability. From Compound **2** to **33**, the HBD count was lowered from 4 to 2, and LogD was lowered from 5.54 to 3.86, representing a successful improvement in the drug-like character of our macrocyclic peptide series.

**Figure 7:**
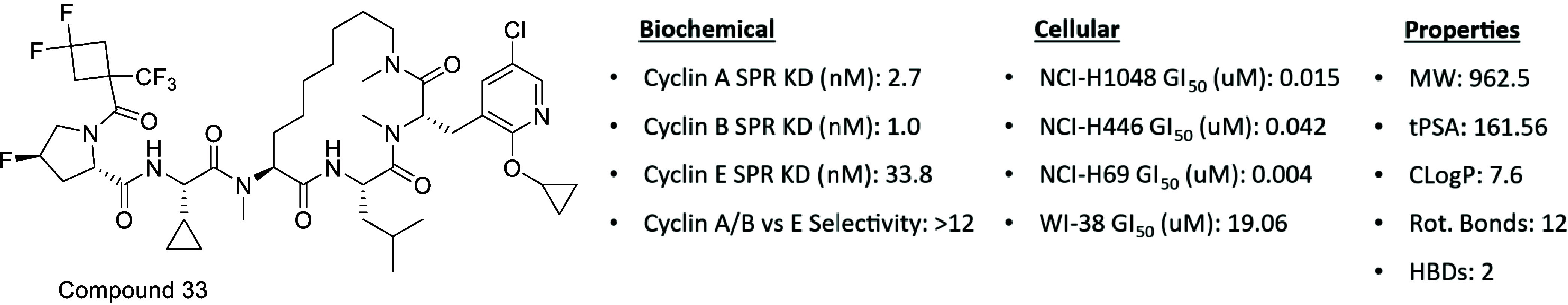
Profile of biophysical and cell potency , molecular properties, and *in vitro* DMPK parameters of Compound **33.**

**33** was modeled in the RxL binding site of Cyclin A (Figure 8) based on published cocrystal structures as previously described.^39^ Similar to the case for **2** (Figure 5a), the backbone of **33** is expected to form multiple H-bonds at the binding interface. The two remaining H-bond donors are involved in polar contacts with Gln254 and Ile281. All amide bonds not directly engaging in productive interaction with the target are alkylated. The replacement of the bridging residue in the macrocycle with an alkyl chain and the methylation of the extended lariat are not predicted to disrupt the ability of the scaffold to adopt an active conformation. The replacement of the spiro-cap with the difluorocyclobutyl-cap does not alter the predicted orientation of the trifluoromethyl group into the S-pocket. The 5-chloro and 2-pyridone cyclopropyl ether substituents do not appear to make any clashes with either the HP or the shallow shelf consisting of Ile231, Met210, and Arg250.

**Figure 8:**
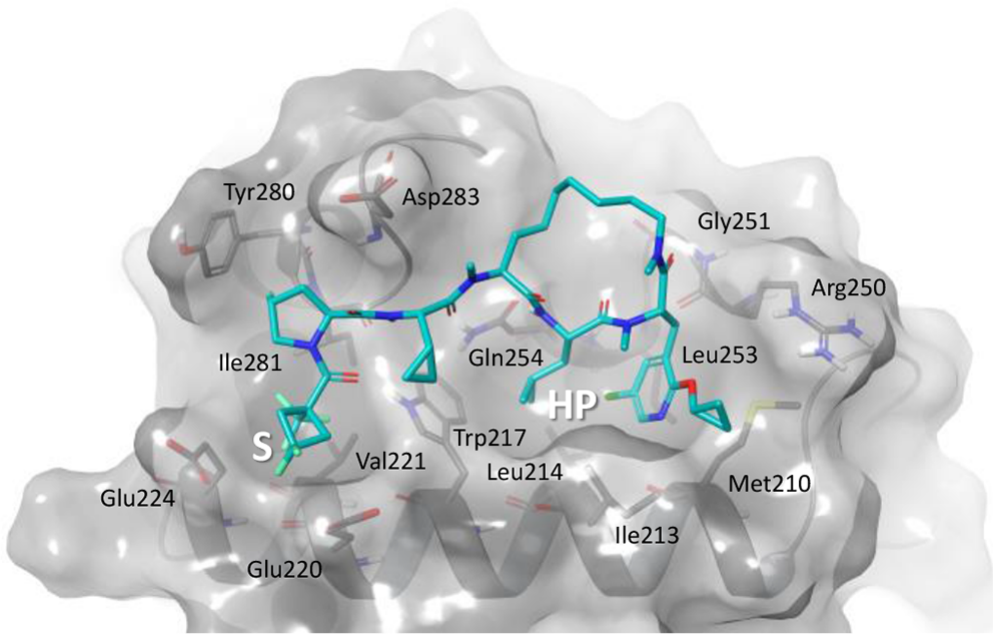
Computational docking model of Compound **33** in the RxL binding site of Cyclin A.

In addition to our general screening antiproliferation assay against NCI-H1048, two additional SCLC lines were evaluated. Based on the efficacy results of Compound **2** against NCI-H69 and NCI-H1048 *in vivo*,^39^ we wanted to evaluate **33** in NCI-H69 and a more challenging model (besides NCI-H1048) using a cell line of intermediate sensitivity to our compounds. We determined that **33** inhibits NCI-H446 with a GI_50_ of 42 nM, compared to GI_50_ values of 4 nM in NCI-H69 and 15 nM in NCI-H1048. Therefore, NCI-H69 and NCI-H446 were selected for *in vivo* efficacy studies. To ensure dose linearity, **33** was administered via PO dosing in mice at 100 mpk and showed proportional exposure (Table 8). With these results in hand, **33** was nominated to progress to PO efficacy evaluation against two CDX mouse models of SCLC.

**Table 8:** Dose-Escalating Pharmacokinetics in a Mouse for Compound **33** ^a^

Route (Mouse)	Dose (mg/kg)	*T*_max_ (h)	*C*_max_ Total drug (ng/mL)	*C*_max_ Free drug (ng/mL)	AUC-inf (ng·h/mL)	*T*_1/2_ (h)	Cl (mL/min/kg)	%F PO
IV	2	–	–	–	489.86	1.37	68.05	–
PO	30	1.0	1210.00	60.52	2002.23	0.99	–	27.2
PO	100	0.5	2990.00	101.66	8112.69	3.15	–	34.9

a Oral bioavailability maintained from 30 to 100 mpk.

### *In Vivo* Oral Efficacy of Cyclin A/B RxL Inhibitor 33 in SCLC Tumor Xenograft Mouse Models

We evaluated the *in vivo* antitumor activity of **33** in two SCLC cell-line-derived xenograft (CDX) models, NCI-H69 and NCI-H446 (Figure 9). In NCI-H69, **33** was dosed at 25 and 50 mg/kg via oral dosing (PO) twice daily (BID) for 14 days. Treatment with **33** induced substantial dose-dependent tumor growth inhibition (TGI), including regression, in the NCI-H69 model, with a mean % tumor growth inhibition (%TGI) value of 53% at 25 mg/kg PO BID and a mean regression value of 40% at 50 mg/kg PO BID. In the NCI-H446 model, **33** was dosed at 100 mg/kg PO three times daily (TID), 25 mg/kg PO TID, 100 mg/kg PO BID, and 50 mg/kg PO BID for 14 days. Substantial dose-dependent TGI and regression were observed in all dosing groups, with %TGI of 79% and 85% for 25 mg/kg PO TID and 50 mg/kg BID, respectively, and mean regression values of 80% and 44% for 100 mg/kg PO TID and 100 mg/kg PO BID, respectively. Tumors in the NCI-H446 model were monitored for 27 days after the last dose, and a clear dose response in regrowth was observed in order from highest to lowest response as follows: 100 mg/kg PO TID, 100 mg/kg PO BID, 50 mg/kg PO BID, and 25 mg/kg PO TID.

**Figure 9:**
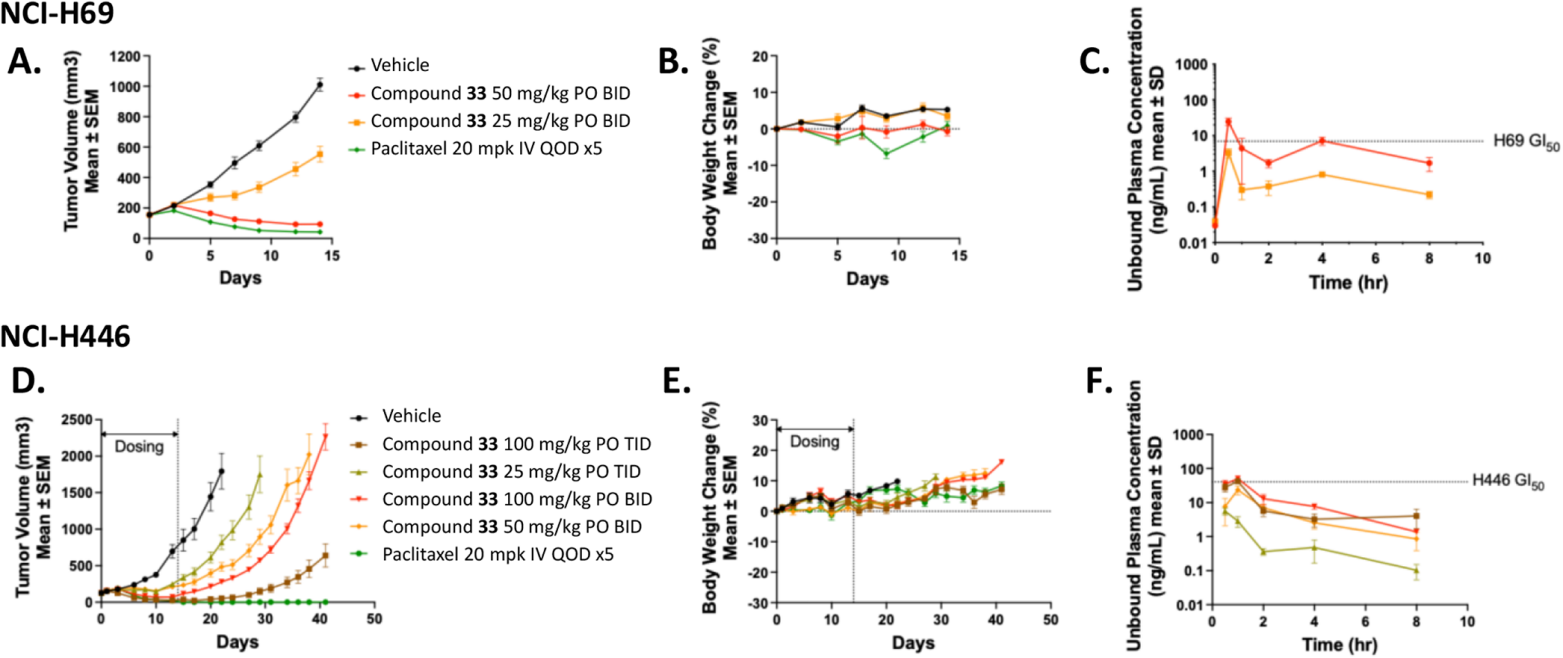
Orally bioavailable Cyclin A/B RxL inhibitor has antitumor activity in the NCI-H69 and NCI-H446 SCLC xenograft models. (A) Tumor volume curves for NCI-H69 xenograft tumors treated with vehicle or Compound 33 at 25 and 50 mg/kg PO BID for 14 days or paclitaxel at 20 mg/kg IV every other day (QOD) × 5 (9 days). (B) Body weight change for treated animals from (A). (C) Unbound plasma concentration of Compound 33 in animals after the final dose from (A). (D) Tumor volume curves for NCI-H446 xenograft tumors treated with vehicle or Compound 33 at 50 and 100 mg/kg PO BID or 25 and 100 mg/kg TID for 14 days or paclitaxel at 20 mg/kg IV QOD × 5 (9 days). (E) Body weight change for treated animals from (D). (F) Unbound plasma concentration of Compound 33 in animals after the final dose from (D). For (A–E), *n* = 10–11 mice per arm, and data are mean ± SEM. For (C–F), *n* = 3–5 mice per time point, and data are mean ± 1 SD.

In both models, treatment was well tolerated, with mean body weight loss not exceeding 10% over the course of the studies. Plasma PK analysis in both studies showed a clear correlation between unbound drug exposure relative to GI_50_ and increased antitumor activity. These results confirm that orally dosed Cyclin A/B inhibitors may present a new treatment option for patients with SCLC.

### Preclinical Pharmacokinetics (PK) of 33

PK of **33** was evaluated in single-dose oral (PO) and intravenous (IV) dose studies in the mouse, rat, dog, and minipig (Table 9). Following a single IV dose of **33**, moderate to high plasma clearance (CL) was observed, ranging from 70% and 74% of total hepatic blood flow rate in the dog and minipig to 94% and 84% of total hepatic blood flow rate in the mouse and rat, respectively. The values of the volume of distribution at steady state (*V*_ss_) were slightly greater than their estimated total body water, suggesting a minor distribution of **33** into tissues. Beyond Rule of 5 compounds like compound **33** often showed some distribution into tissues despite high PPB, due to high lipophilicity. The distribution into tissues suggests that tissue binding or partitioning is even stronger than plasma protein binding in determining overall distribution. The mean elimination half-life (*t*_1/2_) ranged from 0.7 h in mouse to 4.7 h in dog. Following a single PO dose of **33** in the mouse, rat, dog, and minipig, oral bioavailability was found to be greater than 17% in the mouse (27%), rat (17%) and dog (23%), and slightly lower at 6.2% in the minipig.

**Table 9:** Cross-Species Pharmacokinetics of Compound **33**

Parameter	Species	Compound 33
Clearance (mL/min/kg)	Mouse	68
	Rat	46
	Dog	22.4
	Mini-Pig	28
Volume of Distribution (L/kg)	Mouse	1.1
	Rat	3.0
	Dog	2.6
	Mini-Pig	1.8
*t*_1/2_ (h)	Mouse	1.4
	Rat	1.3
	Dog	4.7
	Mini-Pig	1.5
Oral %F	Mouse	27.2
	Rat	17.4
	Dog	22.6
	Mini-Pig	6.2

### *In Vitro* Evaluation of Potential Off-Target Toxicity and Drug–Drug Interaction with 33

As with the IP-administered Cyclin A/B inhibitor Compound **2**, **33** was evaluated in a panel of 489 kinases at 1 and 10 μM test concentrations and showed a clean profile, as expected, given the mechanism of action. **33** also demonstrated no significant off-target activity when tested at up to 10 μM in a safety pharmacology functional panel SAFETYscan47 E/IC50 (Eurofins Discovery) that included cell-based assays to evaluate agonistic and antagonistic activity against GPCR proteins and ion channels, enzyme inhibitors, and transporter blockers (see Supporting Information). Compound **33** also showed no off-target activity in the KINOMEscan assay (Eurofins Discovery) (see Supporting Information). Finally, **33** showed some levels of reversible inhibition against human cytochrome P450 (CYP) 3A4 and 2D6 and potential irreversible inhibition against CYP3A4 (see Supporting Information).

## Conclusions

Herein, we report the lead optimization campaign from a proof-of-concept molecule with IP efficacy to a novel class of orally bioavailable Cyclin A/B RxL inhibitors. Advanced lead compound **33** displays potent and selective antiproliferative activity against SCLC tumor cells and *in vivo* efficacy against CDX mouse models of SCLC when dosed orally. This was achieved by leveraging both computationally guided structure- and property-based design, which is part of what we refer to as our MXMO platform, and the rich SAR data obtained in the Compound **2** development campaign. In the early stages of the project, progress was driven entirely by high-throughput solid-phase peptide synthesis, but as the needs of the project changed and the scaffold design evolved beyond standard SPPS capabilities, solution-phase chemistry was required, highlighting successful cross-functional collaboration and the value of adaptability in peptide drug discovery.

During this project, we identified Circle’s first clinical candidate, CID-078, which is structurally related but distinct from **33** and has a similar *in vitro* and *in vivo* profile. CID-078 is currently being investigated in a Phase 1 clinical trial for patients with SCLC, triple-negative breast cancer, or solid tumors harboring the RB1 mutation (NCT06577987). This highlights the power of medicinal chemistry to discover drug-like compounds in the bRo5 peptide space and marks a major milestone for Cyclin A/B inhibition as a clinical-stage target. The structure, preclinical profile, and human trial data for CID-078 will be disclosed in future communications.

## Experimental Section

### Synthesis and Characterization

Unless otherwise mentioned, all solvents and chemicals were obtained from commercial sources and used without purification. Compounds **2**–**18** were synthesized using SPPS. Linear sequences were synthesized in parallel on a Biotage Syro II peptide synthesizer equipped with 48 reaction vials using 2-chlorotrityl chloride resin, Fmoc-protected amino acids, and HATU coupling. Site-specific on-resin methylation was achieved using a modified Fukuyama-Mitsunobu protocol in toluene on the same instrument. Linear peptides were cleaved from the resin with TFA or HFIP, concentrated under reduced pressure, and cyclized using T3P with DIEA in DCM/DMF. Compounds **19**–**33** were synthesized using the solution-phase chemistry outlined in the Supporting Information. All products were purified via HPLC. The progress of reactions was typically monitored by LC–MS (Waters UPLC Acquity I-Class equipped with Acquity QDa). Purification of final compounds to >95% purity was carried out by reverse-phase C18 chromatography using a Waters HPLC system equipped with the following components: Waters 2767 Sample Manager, Waters 1525 Binary HPLC Pump, Waters 2545 Binary Gradient Module, Waters SFO System Fluidics Organizer, 515 HPLC Pump, Waters Acquity QDa, and Waters 2998 Photodiode Array Detector. Proton (1H) and carbon (13C) NMR spectra were recorded on a Bruker Avance 400 (500 MHz for 1H; 1101 MHz for 13C). Chemical shifts are given in parts per million (ppm) (δ relative to the residual solvent peak for 1H and 13C).

### Synthesis of Compound 23

#### *Tert-butyl (S)-(3-(2,5-dichlorophenyl)-1-(hex-5-en-1-ylamino)-1-oxopropan-2-yl)­(methyl)­carbamate* **(SI-10)**

To a 500 mL round-bottom flask were added (S)-2-((tert-butoxycarbonyl)­(methyl)­amino)-3-(2,5-dichlorophenyl)­propanoic acid (2.5 g, 7.2 mmol, 1 equiv) and HATU (3.3 g, 8.6 mmol, 1.2 equiv). The solids were dissolved in DMF (20 mL), and to the solution was added hex-5-en-1-amine (0.85 g, 1.0 mL, 8.6 mmol, 1.2 equiv) followed by DIPEA (3.2 g, 4.4 mL, 25 mmol, 3.5 equiv). The solution was confirmed basic by pH paper and stirred for 2 h. The reaction mixture was diluted in water (100 mL) and extracted with ethyl acetate (100 mL × 3). The combined organics were washed with brine (150 mL), dried over anhydrous magnesium sulfate, and filtered over Celite. The organic extract was concentrated under rotary evaporation to give a yellow-orange oil. The crude material was purified by silica gel chromatography (0–100% ethyl acetate in hexanes), and the eluent was concentrated to give *tert*-butyl (S)-(3-(2,5-dichlorophenyl)-1-(hex-5-en-1-ylamino)-1-oxopropan-2-yl)­(methyl)­carbamate (2.2 g, 5.1 mmol, 71% yield) as a colorless oil.

**HRMS:** MS (ESI) mass calcd. for C_21_H_30_Cl_2_N_2_O_3_: 438.16 *m*/*z*; Found 373.1096 [M-tBu]^+^.

^**1**^ **H NMR:** (400 MHz, D_3_COD) δ = 7.49–7.16 (m, 3H), 5.85–5.72 (m, 1H), 5.04–4.93 (m, 4H), 3.50–3.35 (m, 1H), 3.27–3.14 (m, 2H), 3.11–2.98 (m, 1H), 2.77 (s, 3H), 2.08 (br d, J = 6.8 Hz, 2H), 1.52 (br s, 2H), 1.44–1.40 (m, 1H), 1.39–1.22 (m, 9H)

^**13**^ **C NMR:** (101 MHz, D_3_COD) δ = 170.494, 170.487, 170.465, 170.313, 170.302, 155.978, 155.483, 138.302, 137.94, 137.709, 137.612, 137.601, 132.552, 132.45, 132.227, 131.15, 130.919, 130.887, 130.482, 130.471, 128.04, 127.972, 127.838, 113.745, 80.585, 80.516, 80.252, 59.167, 57.975, 48.461, 48.378, 48.324, 48.252, 48.169, 48.111, 48.039, 47.956, 47.898, 47.825, 47.746, 47.681, 47.612, 47.533, 47.399, 47.186, 46.973, 39.154, 39.146, 39.114, 39.013, 38.984, 38.966, 33.086, 32.013, 31.854, 31.814, 31.738, 30.861, 30.807, 30.46, 30.315, 28.549, 26.971, 26.797, 25.941

#### Tert-butyl ((S)-1-(((S)-3-(2,5-dichlorophenyl)-1-(hex-5-en-1-ylamino)-1-oxopropan-2-yl)­(methyl)­amino)-4-methyl-1-oxopentan-2-yl)­carbamate (**SI-11**)

*tert*-butyl (S)-(3-(2,5-dichlorophenyl)-1-(hex-5-en-1-ylamino)-1-oxopropan-2-yl)­(methyl)­carbamate (2.2 g, 5.1 mmol, 1 equiv) was dissolved in DCM (15 mL) and TFA (15 mL) and allowed to sit at room temperature for 30 min until the Boc group was removed, as confirmed by LCMS. The reaction mixture was concentrated by rotary evaporation. The residue was resuspended in toluene and concentrated to remove residual TFA, and this procedure was repeated two times. The crude residue was used in the next reaction without further purification.

To a 500 mL round-bottom flask containing the residue was added a solution of Boc-l-Leucine monohydrate (1.5 g, 6.1 mmol, 1.2 equiv), HATU (2.3 g, 6.1 mmol, 1.2 equiv), and DIPEA (2.3 g, 3.1 mL, 18 mmol, 3.5 equiv) in DMF (20 mL). The reaction mixture was checked by pH paper, and DIPEA was added in 1 equiv portions until the reaction was confirmed to be basic (pH ∼ 9). The reaction was stirred for 2 h at room temperature. The reaction mixture was diluted in water (100 mL) and extracted with ethyl acetate (100 mL × 3). The combined organics were washed with brine (150 mL), dried over anhydrous magnesium sulfate, and filtered over Celite. The organic extract was concentrated under rotary evaporation to give a yellow-orange oil. The crude material was purified by silica gel chromatography (0–100% ethyl acetate in hexanes), and the eluent was concentrated to give *tert*-butyl ((S)-1-(((S)-3-(2,5-dichlorophenyl)-1-(hex-5-en-1-ylamino)-1-oxopropan-2-yl)­(methyl)­amino)-4-methyl-1-oxopentan-2-yl)­carbamate (2.4 g, 4.4 mmol, 86% yield) as a colorless oil.

**HRMS:** MS (ESI) mass calcd. for C_27_H_41_Cl_2_N_3_O_4_: 541.25 *m*/*z*; Found 542.2565 [M + H]^+^.

^**1**^ **H NMR:** (400 MHz, D_3_COD) δ = 7.48–7.35 (m, 1H), 7.31 (qd, J = 2.4, 4.5 Hz, 1H), 7.27–7.20 (m, 1H), 5.79 (tdd, J = 6.8, 10.3, 17.0 Hz, 1H), 5.03–4.91 (m, 4H), 4.58 (s, 2H), 4.50–4.18 (m, 1H), 3.55–3.36 (m, 1H), 3.28–3.20 (m, 1H), 3.20–3.06 (m, 2H), 3.01 (s, 1H), 2.90 (s, 1H), 2.13–1.98 (m, 2H), 1.73–1.62 (m, 1H), 1.61–1.52 (m, 2H), 1.51–1.45 (m, 2H), 1.42 (d, J = 1.2 Hz, 9H), 1.36–1.30 (m, 1H), 1.28–1.10 (m, 1H), 0.92 (dd, J = 6.8, 10.4 Hz, 3H), 0.71 (dd, J = 4.8, 6.4 Hz, 2H)

^**13**^ **C NMR:** (101 MHz, D_3_COD) δ = 175.037, 174.214, 169.8, 168.872, 157.256, 156.682, 138.298, 138.172, 137.63, 137.225, 132.884, 132.44, 132.414, 132.306, 131.631, 131.295, 131.11, 130.489, 128.709, 128.163, 113.846, 113.759, 79.602, 79.191, 60.192, 58.986, 49.646, 48.324, 48.252, 48.111, 48.039, 47.959, 47.898, 47.825, 47.746, 47.681, 47.612, 47.399, 47.186, 46.977, 40.165, 39.334, 38.958, 38.352, 33.357, 33.075, 33.031, 31.868, 31.529, 29.174, 28.426, 28.376, 27.365, 26.017, 25.84, 24.493, 23.803, 22.369, 22.117, 20.303, 19.328

#### *Tert-butyl ((S)-1-(((S)-1-(((S)-3-(2,5-dichlorophenyl)-1-(hex-5-en-1-ylamino)-1-oxopropan-2-yl)­(methyl)­amino)-4-methyl-1-oxopentan-2-yl)­amino)-1-oxopent-4-en-2-yl)­(methyl)­carbamate* **(SI-12)**

*tert*-butyl ((S)-1-(((S)-3-(2,5-dichlorophenyl)-1-(hex-5-en-1-ylamino)-1-oxopropan-2-yl)­(methyl)­amino)-4-methyl-1-oxopentan-2-yl)­carbamate (2.4 g, 4.4 mmol, 1 equiv) was dissolved in DCM (15 mL) and TFA (15 mL) and allowed to sit at room temperature for 30 min until the Boc group was removed, as confirmed by LCMS. The reaction mixture was concentrated by rotary evaporation. The residue was resuspended in toluene and concentrated to remove residual TFA, and this procedure was repeated two times. The crude residue was used in the next reaction without further purification.

To a 500 mL round-bottom flask containing the residue was added a solution of (S)-2-((tert-butoxycarbonyl)­(methyl)­amino)­pent-4-enoic acid (1.2 g, 5.3 mmol, 1.2 equiv), HATU (2.0 g, 5.3 mmol, 1.2 equiv), and DIPEA (2.0 g, 2.7 mL, 15 mmol, 3.5 equiv) in DMF (20 mL). The reaction mixture was checked by pH paper, and DIPEA was added in 1 eq. portions until the reaction was confirmed to be basic (pH ∼ 9). The reaction was stirred for 2 h at room temperature. The reaction mixture was diluted in water (100 mL) and extracted with ethyl acetate (100 mL × 3). The combined organics were washed with brine (150 mL), dried over anhydrous magnesium sulfate, and filtered over Celite. The organic extract was concentrated under rotary evaporation to give a yellow-orange oil. The crude material was purified by silica gel chromatography (0–100% ethyl acetate in hexanes), and the eluent was concentrated to give *tert*-butyl ((S)-1-(((S)-1-(((S)-3-(2,5-dichlorophenyl)-1-(hex-5-en-1-ylamino)-1-oxopropan-2-yl)­(methyl)­amino)-4-methyl-1-oxopentan-2-yl)­amino)-1-oxopent-4-en-2-yl)­(methyl)­carbamate (2.72 g, 4.16 mmol, 94% yield) as a pale yellow oil.

**HRMS:** MS (ESI) mass calcd. for C C_33_H_50_Cl_2_N_4_O_5_: 652.32 *m*/*z*; Found 653.3250 [M + H]^+^.

^**1**^ **H NMR:** (400 MHz, D_3_COD) δ = 8.51–8.06 (m, 1H), 7.94–7.62 (m, 1H), 7.48–7.35 (m, 1H), 7.34–7.30 (m, 1H), 7.26–7.22 (m, 1H), 5.90–5.66 (m, 2H), 5.18–5.04 (m, 2H), 4.99–4.91 (m, 2H), 4.72 (ddd, J = 3.2, 7.5, 10.8 Hz, 1H), 4.67–4.42 (m, 2H), 3.51–3.36 (m, 1H), 3.28–3.22 (m, 1H), 3.21–3.09 (m, 2H), 3.03 (s, 1H), 2.90 (s, 1H), 2.82 (s, 2H), 2.58 (td, J = 5.2, 9.7 Hz, 1H), 2.49–2.39 (m, 1H), 2.17–2.00 (m, 2H), 1.69–1.52 (m, 3H), 1.45 (d, J = 8.4 Hz, 10H), 1.40–1.33 (m, 1H), 1.26–1.16 (m, 1H), 0.92 (br dd, J = 6.4, 13.3 Hz, 4H), 0.79–0.68 (m, 2H)

^**13**^ **C NMR:** (101 MHz, D_3_COD) δ = 173.369, 169.743, 168.771, 138.298, 138.244, 137.605, 137.207, 134.206, 134.058, 132.92, 132.469, 132.432, 132.27, 131.681, 131.284, 131.143, 130.511, 128.748, 128.178, 116.732, 113.864, 113.799, 60.283, 58.888, 48.335, 48.263, 48.122, 48.049, 47.963, 47.909, 47.836, 47.692, 47.623, 47.41, 47.197, 46.984, 39.865, 39.32, 39.005, 33.418, 33.089, 33.053, 31.883, 31.626, 29.16, 28.43, 28.206, 27.285, 27.26, 25.978, 25.869, 24.518, 23.901, 22.413, 22.138, 20.249, 19.321

#### *Tert-butyl ((1S,4S,7S,10S)-4-allyl-1-cyclopropyl-10-(2,5-dichlorobenzyl)-7-isobutyl-3,9-dimethyl-2,5,8,11-tetraoxo-3,6,9,12-tetraazaoctadec-17-en-1-yl)­carbamate* **(SI-13)**

*tert*-butyl ((S)-1-(((S)-1-(((S)-3-(2,5-dichlorophenyl)-1-(hex-5-en-1-ylamino)-1-oxopropan-2-yl)­(methyl)­amino)-4-methyl-1-oxopentan-2-yl)­amino)-1-oxopent-4-en-2-yl)­(methyl)­carbamate (2.7 g, 4.1 mmol, 1 equiv) was dissolved in DCM (15 mL) and TFA (15 mL) and allowed to sit at room temperature for 30 min until the Boc group was removed, as confirmed by LCMS. The reaction mixture was concentrated by rotary evaporation. The residue was resuspended in toluene and concentrated to remove residual TFA, and this procedure was repeated two times. The crude residue was used in the next reaction without further purification.

To a 500 mL round-bottom flask containing the residue was added a solution of (S)-2-((tert-butoxycarbonyl)­amino)-2-cyclopropylacetic acid (1.1 g, 5.0 mmol, 1.2 equiv), HATU (1.9 g, 5.0 mmol, 1.2 equiv), and DIPEA (1.9 g, 2.5 mL, 14 mmol, 3.5 equiv) in DMF (20 mL). The reaction mixture was checked by pH paper, and DIPEA was added in 1 eq. portions until the reaction was confirmed to be basic (pH ∼ 9). The reaction was stirred for 2 h at room temperature. The reaction mixture was diluted in water (100 mL) and extracted with ethyl acetate (100 mL × 3). The combined organics were washed with brine (150 mL), dried over anhydrous magnesium sulfate, and filtered over Celite. The organic extract was concentrated under rotary evaporation to give a yellow-orange oil. The crude material was purified by silica gel chromatography (0–100% ethyl acetate in hexanes), and the eluent was concentrated to give *tert*-butyl ((1S,4S,7S,10S)-4-allyl-1-cyclopropyl-10-(2,5-dichlorobenzyl)-7-isobutyl-3,9-dimethyl-2,5,8,11-tetraoxo-3,6,9,12-tetraazaoctadec-17-en-1-yl)­carbamate (3.02 g, 4.02 mmol, 97% yield) as a pale yellow oil.

**HRMS:** MS (ESI) mass calcd. for C_38_H_5_7Cl_2_N_5_O_6_: 749.37 *m*/*z*; Found 750.3776 [M + H]^+^.

^**1**^ **H NMR:** (400 MHz, D_3_COD) δ = 8.74–8.25 (m, 1H), 8.01–7.49 (m, 1H), 7.47–7.36 (m, 1H), 7.35–7.28 (m, 1H), 7.28–7.17 (m, 1H), 5.89–5.64 (m, 2H), 5.24–5.04 (m, 2H), 5.03–4.90 (m, 5H), 4.75–4.62 (m, 2H), 4.61–4.34 (m, 1H), 4.29–3.96 (m, 1H), 3.53–3.35 (m, 1H), 3.29–3.21 (m, 1H), 3.18–3.11 (m, 1H), 3.11–3.01 (m, 3H), 2.90 (s, 1H), 2.76 (d, J = 13.6 Hz, 1H), 2.65–2.36 (m, 2H), 2.12–1.75 (m, 2H), 1.65–1.47 (m, 3H), 1.46–1.42 (m, 9H), 1.42–1.39 (m, 2H), 1.38–1.04 (m, 3H), 0.95–0.85 (m, 3H), 0.74–0.67 (m, 2H), 0.66–0.56 (m, 1H), 0.56–0.30 (m, 4H)

^**13**^ **C NMR:** (101 MHz, D_3_COD) δ = 174.401, 174.143, 174.087, 173.893, 173.7, 173.26, 173.161, 172.994, 171.599, 171.515, 171.129, 170.954, 170.875, 170.518, 170.427, 169.851, 169.767, 169.68, 168.808, 168.774, 156.502, 156.187, 150.518, 138.326, 138.288, 138.269, 137.659, 137.613, 137.313, 137.215, 133.756, 133.688, 133.521, 132.933, 132.482, 132.444, 132.285, 131.697, 131.303, 131.17, 130.601, 130.548, 128.83, 128.777, 128.466, 128.224, 120.681, 117.473, 117.427, 116.896, 113.938, 113.9, 113.84, 79.255, 79.19, 79.08, 60.445, 60.316, 59.713, 59.05, 58.936, 58.872, 58.86, 57.396, 56.653, 56.183, 53.964, 53.233, 53.153, 49.562, 49.467, 48.454, 48.36, 48.291, 48.079, 47.863, 47.65, 47.438, 47.226, 47.104, 47.013, 39.869, 39.402, 39.368, 39.19, 39.156, 39.031, 38.45, 37.98, 37.313, 34.177, 33.475, 33.43, 33.122, 33.103, 32.296, 32.262, 31.901, 31.613, 31.552, 31.094, 31.01, 29.224, 28.803, 28.655, 28.454, 28.412, 28.306, 27.563, 27.434, 27.343, 26.008, 25.894, 24.579, 24.469, 23.816, 23.71, 22.481, 22.258, 22.22, 20.248, 19.944, 19.342, 19.015, 12.69, 12.531, 12.189, 12.121, 3.831, 3.74, 2.739, 2.694, 2.375, 2.326, 1.704, 1.617

#### *Tert-butyl ((S)-1-cyclopropyl-2-(((3S,6S,9S)-3-(2,5-dichlorobenzyl)-6-isobutyl-4-methyl-2,5,8-trioxo-1,4,7-triazacyclohexadec-11-en-9-yl)­(methyl)­amino)-2-oxoethyl)­carbamate* **(SI-14)**

*tert*-butyl ((1S,4S,7S,10S)-4-allyl-1-cyclopropyl-10-(2,5-dichlorobenzyl)-7-isobutyl-3,9-dimethyl-2,5,8,11-tetraoxo-3,6,9,12-tetraazaoctadec-17-en-1-yl)­carbamate (3.02 g, 4.02 mmol, 1 equiv) was dissolved in DCE (300 mL) to give a 13.4 mM solution. The solution was degassed with nitrogen for 20 min. To the solution was added Hoveyda-Grubbs II catalyst (M720 Umicore, 378 mg, 0.15 equiv), and the solution was degassed for an additional 5 min. The reaction was heated to 70 °C and stirred under nitrogen overnight. Consumption of the starting material was confirmed by LCMS. The reaction was concentrated by rotary evaporation and purified by reverse-phase flash chromatography (50–100% acetonitrile in water, 0.1% TFA buffer) to give *tert*-butyl ((S)-1-cyclopropyl-2-(((3S,6S,9S)-3-(2,5-dichlorobenzyl)-6-isobutyl-4-methyl-2,5,8-trioxo-1,4,7-triazacyclohexadec-11-en-9-yl)­(methyl)­amino)-2-oxoethyl)­carbamate (1.65 g, 2.3 mmol, 57.3% yield) as a dark brown solid, which was carried on without further purification. An aliquot was further purified by reversed-phase HPLC (50–100% acetonitrile in water, 0.05% formic acid buffer) for analytical purposes. Isolated as a mixture of isomers (∼3:1). Analytical data are given for the major peak.

**HRMS:** MS (ESI) mass calcd. for C_36_H_53_Cl_2_N_5_O_6_: 721.34 *m*/*z*; Found 722.3469 [M + H]^+^.

^**1**^ **H NMR:** (400 MHz, D_3_COD) δ = 7.48–7.38 (m, 1H), 7.35–7.27 (m, 1H), 7.16–7.02 (m, 1H), 5.56–5.40 (m, 1H), 5.40–5.07 (m, 1H), 4.67 (br dd, J = 2.0, 12.4 Hz, 2H), 4.59 (br s, 9H), 4.22–4.12 (m, 1H), 3.67–3.49 (m, 1H), 3.49–3.46 (m, 1H), 3.09 (s, 1H), 2.79 (d, J = 7.2 Hz, 4H), 2.21–1.84 (m, 2H), 1.83–1.52 (m, 2H), 1.52–1.39 (m, 9H), 1.38–1.21 (m, 2H), 1.20–1.06 (m, 1H), 1.01–0.70 (m, 6H), 0.70–0.30 (m, 4H)

^**13**^ **C NMR:** (101 MHz, D_3_COD) δ = 173.832, 172.782, 172.532, 171.887, 170.181, 169.703, 156.642, 137.996, 132.789, 132.478, 132.312, 131.856, 130.692, 130.605, 128.33, 128.311, 125.797, 124.943, 79.277, 79.228, 63.502, 59.482, 56.096, 54.294, 53.388, 48.568, 48.261, 48.049, 47.836, 47.624, 47.411, 47.195, 46.983, 39.74, 38.822, 38.735, 38.523, 38.007, 32.353, 31.716, 31.442, 31.37, 30.912, 30.237, 28.564, 28.306, 27.798, 27.696, 27.472, 27.298, 25.428, 25.018, 24.931, 24.499, 22.523, 22.341, 19.482, 12.603, 2.826, 2.682, 1.761, 1.7

#### *Tert-butyl ((S)-1-cyclopropyl-2-(((3S,6S,9S)-3-(2,5-dichlorobenzyl)-6-isobutyl-4-methyl-2,5,8-trioxo-1,4,7-triazacyclohexadecan-9-yl)­(methyl)­amino)-2-oxoethyl)­carbamate* **(SI-22)**

*tert*-butyl ((S)-1-cyclopropyl-2-(((3S,6S,9S)-3-(2,5-dichlorobenzyl)-6-isobutyl-4-methyl-2,5,8-trioxo-1,4,7-triazacyclohexadec-11-en-9-yl)­(methyl)­amino)-2-oxoethyl)­carbamate (1.6 g, 2.2 mmol, 1 equiv) was dissolved in EtOAc (200 mL). To the solution was added 10% Palladium on Carbon (1.6 g, 1.5 mmol, 0.68 equiv) as a solid. The reaction vessel was evacuated and backfilled with hydrogen gas 10 times. The reaction was stirred under 1 atm of hydrogen for 2 h. Consumption of the starting material was confirmed by LCMS. The reaction was filtered through a pad of Celite, and the filtrate was concentrated by rotary evaporation to give *tert*-butyl ((S)-1-cyclopropyl-2-(((3S,6S,9S)-3-(2,5-dichlorobenzyl)-6-isobutyl-4-methyl-2,5,8-trioxo-1,4,7-triazacyclohexadecan-9-yl)­(methyl)­amino)-2-oxoethyl)­carbamate (1.5 g, 2.1 mmol, 93% yield) as a pale brown solid, which was carried on without further purification. An aliquot was further purified by reverse-phase HPLC (50–100% acetonitrile in water, 0.05% formic acid buffer) for analytical purposes.

**HRMS:** MS (ESI) mass calcd. for C_36_H_56_Cl_2_N_5_O_6_: 724.36 *m*/*z*; Found 724.3622 [M + H]^+^.

^**1**^ **H NMR:** (400 MHz, CD_3_OD_3_) δ = 7.66–7.57 (m, 1H), 7.53–7.45 (m, 1H), 7.37–7.20 (m, 1H), 5.38–5.23 (m, 1H), 4.93–4.84 (m, 2H), 4.73–4.64 (m, 1H), 4.62–4.31 (m, 2H), 3.94–3.76 (m, 1H), 3.72–3.64 (m, 1H), 3.48–3.26 (m, 1H), 3.26–3.19 (m, 1H), 3.18–2.91 (m, 5H), 1.90–1.74 (m, 1H), 1.73–1.54 (m, 17H), 1.51–1.35 (m, 2H), 1.31–1.21 (m, 1H), 1.12–1.04 (m, 2H), 1.00–0.95 (m, 2H), 0.93–0.78 (m, 3H), 0.75 (br s, 1H)

^**13**^ **C NMR:** (101 MHz, CD_3_OD_3_) δ = 223.748, 209.755, 174.41, 174.118, 172.277, 172.145, 171.86, 171.784, 171.481, 170.779, 170.487, 170.115, 168.84, 156.235, 156.227, 137.786, 137.354, 132.933, 132.444, 132.341, 132.273, 132.144, 131.795, 131.184, 130.588, 128.854, 128.763, 128.289, 79.341, 79.277, 59.963, 58.878, 56.484, 55.896, 53.145, 52.432, 48.759, 48.736, 48.709, 48.531, 48.227, 47.802, 47.587, 47.374, 46.949, 46.668, 39.432, 39.235, 38.95, 37.926, 36.81, 31.889, 31.775, 31.688, 30.136, 30.003, 29.1, 28.891, 28.622, 28.478, 28.383, 28.079, 27.95, 27.635, 27.464, 27.411, 27.271, 26.967, 26.315, 26.265, 26.14, 25.84, 24.418, 23.924, 23.757, 23.617, 23.386, 23.044, 22.911, 22.494, 22.35, 22.126, 22.069, 21.09, 19.614, 19.269, 18.923, 12.598, 12.461, 12.203, 2.524, 2.437, 2.247, 1.788, 1.727, 1.53, 0.767

#### *(2S,4R)-N-((S)-1-cyclopropyl-2-(((3S,6S,9S)-3-(2,5-dichlorobenzyl)-6-isobutyl-4-methyl-2,5,8-trioxo-1,4,7-triazacyclohexadecan-9-yl)­(methyl)­amino)-2-oxoethyl)-1-(3,3-difluoro-1-(trifluoromethyl)­cyclobutane-1-carbonyl)-4-fluoropyrrolidine-2-carboxamide* **(Compound 23)**

*tert*-butyl ((S)-1-cyclopropyl-2-(((3S,6S,9S)-3-(2,5-dichlorobenzyl)-6-isobutyl-4-methyl-2,5,8-trioxo-1,4,7-triazacyclohexadecan-9-yl)­(methyl)­amino)-2-oxoethyl)­carbamate (0.05 g, 0.069 mmol, 1 equiv) was dissolved in DCM (1 mL) and TFA (1 mL) and allowed to sit at room temperature for 30 min until the Boc group was removed, as confirmed by LCMS. The reaction mixture was concentrated by rotary evaporation. The residue was resuspended in toluene and concentrated to remove residual TFA, and this procedure was repeated two times. The crude residue was used in the next reaction without further purification.

To a vial containing the residue was added a solution of (2*S*,4*R*)-1-(3,3-difluoro-1-(trifluoromethyl)­cyclobutane-1-carbonyl)-4-fluoropyrrolidine-2-carboxylic acid (0.026 g, 0.083 mmol, 1.2 equiv), HATU (0.031 g, 0.083 mmol, 1 equiv), and DIPEA (0.031 g, 0.042 mL, 0.24 mmol, 3.5 equiv) in DMF (1 mL). The reaction mixture was checked by pH paper, and DIPEA was added in 1 eq. portions until the reaction was confirmed to be basic (pH ∼ 9). The reaction was allowed to sit for 2 h at room temperature. The reaction mixture was diluted in water (100 mL) and extracted with ethyl acetate (100 mL × 3). The combined organics were washed with brine (150 mL), dried over anhydrous magnesium sulfate, and filtered over Celite. The organic extract was concentrated under rotary evaporation to give a yellow-orange oil. The crude material was purified by reverse-phase HPLC (50–100% acetonitrile in water, 0.05% formic acid buffer), and the eluent was lyophilized to give (2*S*,4*R*)-N-((S)-1-cyclopropyl-2-(((3S,6S,9S)-3-(2,5-dichlorobenzyl)-6-isobutyl-4-methyl-2,5,8-trioxo-1,4,7-triazacyclohexadecan-9-yl)­(methyl)­amino)-2-oxoethyl)-1-(3,3-difluoro-1-(trifluoromethyl)­cyclobutane-1-carbonyl)-4-fluoropyrrolidine-2-carboxamide (0.044 g, 0.048 mmol, 69% yield) as a fluffy white solid.

**HRMS:** MS (ESI) mass calcd. for C_42_H_57_Cl_2_F_6_N_6_O_6_: 925.36 *m*/*z*; Found 925.3624 [M + H]^+^.

^**1**^ **H NMR:** (400 MHz, CD_3_OD_3_) δ = 7.66–7.56 (m, 1H), 7.53–7.42 (m, 2H), 7.35–7.21 (m, 1H), 5.61–5.37 (m, 1H), 5.11–5.05 (m, 1H), 4.95–4.65 (m, 4H), 4.55–4.29 (m, 1H), 4.15–4.00 (m, 1H), 3.96–3.78 (m, 2H), 3.72–3.61 (m, 2H), 3.45–3.27 (m, 6H), 3.27–3.21 (m, 1H), 3.13–3.04 (m, 2H), 3.03–2.92 (m, 2H), 2.84–2.68 (m, 1H), 2.44–2.09 (m, 2H), 1.96–1.73 (m, 2H), 1.68 (br s, 13H), 1.11–1.02 (m, 2H), 0.99–0.95 (m, 2H), 0.92–0.87 (m, 1H), 0.86–0.77 (m, 2H), 0.76–0.47 (m, 4H)

^**13**^ **C NMR:** (101 MHz, CD_3_OD_3_) δ = 174.156, 172.13, 171.644, 171.625, 171.105, 170.585, 170.13, 168.836, 165.626, 137.889, 137.441, 132.971, 132.501, 132.395, 132.319, 132.205, 131.863, 131.207, 131.135, 130.615, 128.786, 128.3, 126.362, 118.913, 116.185, 93.096, 91.324, 63.697, 63.663, 60.256, 60.017, 59.557, 59.231, 56.404, 55.831, 54.344, 54.219, 52.762, 52.045, 48.778, 48.531, 48.25, 47.611, 46.971, 46.725, 41.291, 41.029, 40.764, 39.553, 39.436, 39.261, 39.034, 38.491, 37.956, 36.818, 34.966, 31.942, 31.726, 30.189, 30.03, 28.884, 28.368, 28.132, 27.013, 26.436, 26.319, 25.829, 24.6, 24.524, 23.867, 23.773, 23.681, 23.116, 22.923, 22.513, 22.399, 22.228, 22.092, 21.113, 19.64, 19.307, 19.132, 11.999, 11.714, 2.486, 1.989, 1.921, 1.856, 1.207

### Calculated and Measured Compound Properties

ClogP and TPSA values were calculated by using the ChemDraw model. MDCK monolayer cell permeability, kinetic solubility (Ksol), mouse plasma protein binding via ultracentrifugation (%PPB), and Caco-2 monolayer permeability (A-B and B-A) were generated by Pharmaron using standard experimental conditions (see Supporting Information).

### Binding Model Prediction

Binding model of **33** to Cyclin A was generated using the Schrödinger suite 2024-3, following previously reported procedures.^39^ In brief, the protein model of Cyclin A was based on the cocrystal structure of Cyclin A and a lariat macrocycle (PDB: 1URC). The protein was prepared using the Protein Preparation Workflow, and the resulting model was used to create the docking grid. The 3D structure of **33** was generated from its SMILES structure using the LigPrep module. The initial binding pose of **33** was obtained by performing alignment to the previously published binding model of Compound **2**. The Ligand Alignment module was utilized to align common structures by the maximum common substructure, with the ligand specified as a macrocycle and the binding site of Cyclin A specified as the receptor. The aligned model was subsequently refined by docking using Glide SP with the “Refine only” option.

### Molecular Matched-Pair Analysis

A sequence-based matched pair analysis was performed to compare molecules at the residue level. With compounds represented as linear peptide sequences, a matched pair is defined as 2 aligned sequences with only 1 residue difference. For each molecular pair, property ratios were calculated to examine the effect of the corresponding residue transformation. For compounds 1 and 2 of a matched pair, the ratio of property A is calculated by the following formula:

$$Ratio_{A}=\frac{A_{compound2}}{A_{compound1}}$$

### Cell Lines and Cell Culture

NCI-H1048, NCI-H446, NCI-H69, and WI-38 cell lines were originally obtained from the American Type Culture Collection (ATCC). NCI-H69 and NCI-H446 cells were maintained in RPMI-1640 media supplemented with 10% fetal bovine serum (FBS), and NCI-H1048 cells were maintained in HITES medium (base medium: DMEM:F12 supplemented with 0.001 mg/mL insulin, 0.01 mg/mL transferrin, 30 nM selenium, 10 nM hydrocortisone, 10 nM beta-estradiol, and an additional 2 mM glutamine to a final concentration of 4.5 mM) supplemented with 5% fetal bovine serum (FBS). WI-38 cells were maintained in DMEM supplemented with 10% FBS.

Early passage cells of all the cell lines listed above were frozen using Recovery Cell Culture Freezing Media (Gibco) and were maintained in culture no more than 4 months, where early passage vials were thawed.

### MTT Proliferation Assay

NCI-H1048 and WI-38 cell lines were plated in 96-well plates at 5 × 10^3^ cells/well with 100 mL of media/well. NCI-H69 cells were grown to confluency in a T150 flask. Cells were collected by centrifugation at 1100 r.p.m. and resuspended in 30 mL media, of which 10 mL was used to seed six 96-well plates. The following day, cells were dosed using a Bravo Liquid Handler (Agilent Technologies). The plate controls, Roscovitine and Staurosporine, were used to define the top and bottom of the growth inhibition curves, respectively. Inhibitors were dosed in duplicate in either an 8- (WI-38, NCI-H1048) or 10-point (NCI-H69) 1:3 serial dilution with 10 μM maximum concentration. Roscovitine was dosed in singlet in an 8- or 10-point 1:2 serial dilution with 100 μM maximum concentration. Staurosporine was dosed in singlet in an 8- or 10-point 1:2 serial dilution with 1 μM maximum concentration. After dosing, plates were maintained in tissue culture incubators (37 °C; 5% CO_2_) for 3 days (WI-38) or 5 days (NCI-H1048, NCI-H69) to allow for at least 2 cell doublings before processing in an MTT proliferation assay (R&D Systems, #4890-050-K) performed according to manufacturer instructions. The average absorbance value obtained with the highest two concentrations dosed for roscovitine and staurosporine was used for background subtraction. The top of the assay (100% growth) was determined by normalization with the top of the roscovitine curve. Growth inhibition 50 (GI_50_) was determined by nonlinear regression analysis using log­(inhibitor) vs response-variable slope (four parameters) using GraphPad Prism (10.1.0) software.

### Fluorescence Polarization Assay

Binding affinity for the compounds of Formula I was determined by Fluorescence Polarization (FP) competitive assay based on previously established protocols^14,27^ with modifications as described below. Cyclin/CDK protein complexes were sourced as follows: CyclinA2/CDK2 (CRELUX Protein Services), CyclinB1/CDK1 (Eurofins Discovery, Cat. No. 14-450), and CyclinE1/CDK2 (Eurofins Discovery, Cat. No. 14-475). FP binding assays were performed in 25 mM HEPES pH 7.5, 100 mM NaCl, 1 mM DTT, 0.01% NP-40, and 1 mg/mL BSA for all 3 protein complexes in black 96-well plates. After experimental plates are set, they were equilibrated by gentle mixing by placing them on an orbital shaker at 100 rpm for 2 h at RT and then read on a SpectraMax i3X Multi-Mode Microplate Detection platform. Affinity of the Cyclin/CDK complexes for the fluorescently labeled probe was determined by adding increasing concentrations of each protein complex in buffer containing a carboxyfluorescein-labeled probe (FAM probe) at 2 nM. For the “FP2 Probe” (Supporting Information), we used 8 nM for Cyclin A2/CDK2 and 10 nM for both Cyclin B1/CDK1 and Cyclin E1/CDK2. Methods to prepare the FAM probes are described in the heading below. Under these conditions, the dynamic range was >100 mP between 100% binding of the FAM probe and complete inhibition of binding by saturating excess of an unlabeled competitor compound, with all experiments showing a Z′ factor >0.80. IC_50_ values for test compounds were determined in eight-point serial dilution dose-response curves. Reported IC_50_ values are the average of 2–3 independent experiments.

### Single-Dose Intravenous (IV) and Oral (PO) PK in Mice

All experiments were performed by following the guidance of the Association for the Assessment and Accreditation of Laboratory Animal Care (AAALAC). All procedures were approved by the Institutional Animal Care and Use Committee of the testing facilities. Single-dose intravenous (IV) and oral (PO) mouse PK were dosed in male C57/BL6 mouse, respectively. The dosing formulation was prepared by dissolving the test article in 5% DMSO:10% Solutol HS15:85% DI water to yield a concentration of 0.5 mg/mL for IV and prepared in 30% PEG400:20% Solutol HS15:50% Phosal 53 MCT to yield a concentration of 3.0 mg/mL for the PO dosing. Three mice each were dosed at 2 mg/kg via tail vein (IV) and at 30 mg/kg via oral gavage (PO). Approximately 0.05 mL of blood was collected from a tail or facial vein in tubes containing K3 EDTA at 0.083 (IV only), 0.25, 0.5, 1, 2, 4, 8, and 24 h post-IV and post-PO dose. Plasma samples were obtained via centrifugation, and sample cleanup was conducted by protein precipitation with acetonitrile that contained an internal standard (IS, Dexamethasone). LC–MS/MS (Shimadzu LCMS-8060 and L-40D) quantitation of test articles and IS was achieved with positive ion MRM transitions. The reversed-phase chromatographic system consisted of a gradient mobile phase at a flow rate of 0.6 mL/min, containing 0.1% formic acid in 5% acetonitrile in water (mobile phase A) and 95% acetonitrile in water (mobile phase B), and an ES-CN column. Analyst version 1.6.2 was used to measure peak areas and peak area ratios of test articles to the IS. A calibration curve was constructed from the peak area ratios (test article to IS) with a weighted (1/×2) linear regression using Watson version 7.5 LIMS and a calibration curve ranging between 0.5 and 1000 ng/mL. Individual test article’s plasma concentration vs time profiles were used to calculate the pharmacokinetic parameters by employing a noncompartmental analysis (Phoenix WinNonlin 8.3).

### Single-Dose Intravenous (IV) and Oral (PO) PK in Mouse, Rat, Dog, and Minipig of 33

All experiments were performed following the guidance of the Association for the Assessment and Accreditation of Laboratory Animal Care (AAALAC). All procedures were approved by the Institutional Animal Care and Use Committee of the testing facilities. Preclinical single-dose IV and PO PK of **33** was performed in male C57/BL6 mice, male Sprague–Dawley rats, male beagle dogs, and male Bama minipigs. The dosing formulation was prepared by dissolving **33** in 5% DMSO:10% Solutol HS15:85% DI water to yield a concentration of 2 mg/mL for IV dose in mice and a concentration of 1 mg/mL for IV dose in rats, dogs, and minipigs. The dosing formulation was prepared in 30% PEG400:20% Solutol HS15:50% Phosal 53 MCT to yield concentrations of 6.0 and 3.0 mg/mL for the PO dosing in mice and rats, respectively. Similarly, PO dosing of dogs was prepared by dissolving **33** in the same formulation and yielding a final concentration of 41.7 mg/mL. 0.8 mL of the formulation was then filled into a size#00 Gelatin capsule and sealed before dosing. PO dosing minipigs was prepared by dissolving **33** in the same formulation and yielding a final concentration of 22.5 mg/mL. 8.0 mL of the formulation was then filled into a size 11 gelatin capsule before dosing. Twelve mice were dosed in single-dose IV, and another 12 mice were dosed in single-dose PO, followed by sparse sampling up to 24 h post-dose. Approximately 0.05 mL of blood was collected from a tail or facial vein in tubes containing K3 EDTA. Similarly, rat, dog, and minipig PK were performed with 3 rats per dose group with serial blood sampling in tubes containing K3 EDTA up to 24 h post-dose. Plasma samples were obtained via centrifugation and stored at −80 °C before processing. Protein precipitation with acetonitrile containing an internal standard was conducted to process the plasma samples, followed by vortex mixing and centrifugation. Afterward, clean supernatant was further diluted with water before being injected into LC–MS/MS for quantitative analysis. Shimadzu (LC-30AD or LC-40D) HPLCs were coupled with triple quadrupole tandem mass spectrometers (AB API 5500, 6500+, or Shimadzu LCMS-8060) to quantify **33** and internal standards. The quantitation of **33** was achieved with a positive ion MRM transition at 962.2/171.1, 962.3/422.9, or 962.3/373.1. Internal standard Dexamethasone or Tolbutamide was quantified using positive ion MRM transition 393.2/373.1 or 271.1/155.1, respectively. The reversed-phase chromatographic system consisted of a gradient mobile phase at a flow rate of 0.6 mL/min, containing 0.1% formic acid in water (mobile phase A) and acetonitrile (mobile phase B), and an ES-CN column (for rat and mouse), a Raptor Biphenyl column (for dog), or a Waters XBridge C18 column. Analyst version 1.6.2 was used to measure peak areas and peak area ratios of **33** to the IS. A calibration curve was constructed from the peak area ratios (**33** to IS) with a weighted (1/×2) linear regression using Watson version 7.5 LIMS and a calibration curve ranging between 0.5 and 1000 ng/mL. **33** plasma concentration vs time profiles were used to calculate the pharmacokinetic parameters by employing a noncompartmental analysis (Phoenix WinNonlin 8.3).

### SCLC Cell Line Xenograft Studies

All experiments were performed following the guidance of the Association for Assessment and Accreditation of Laboratory Animal Care (AAALAC). The NCI-H446 xenograft study was approved by the Institutional Animal Care and Use Committee (IACUC) and performed at Pharmaron (Ningbo) Technology Development Co., Ltd. (Animal Use Protocol Number: ON-CELL-XEN-06012021). The NCI-H69 xenograft study was approved by the IACUC and performed at Labcorp Early Development Laboratories, Inc. (Animal Use Protocol Number: VUF05). NCI-H69 and NCI-H446 human small-cell lung cancer cells were obtained from ATCC. Tumor volume (TV) was determined by measuring the length (*L*) and perpendicular width (*W*) with calipers and calculated using the formula

$$TV=0.5\times L\times W^{2}$$

As a measure of efficacy, the % tumor growth inhibition (%TGI) is calculated at the end of treatment using the following formula

$$mean\%TGI=100-\left(\frac{\Delta T_{mean}}{\Delta C_{mean}}\right)\times 100$$

Δ*T*_mean_ and Δ*C*_mean_ represent the mean volume on the day of evaluation minus the tumor volume at the start of dosing for the treatment and control groups, respectively. In cases where the mean tumor volume in treatment groups is smaller at the end of the study in comparison to the start of dosing, regression values are calculated instead using the formula

$$\mathrm{mean \% Regression}=-\left(\frac{\Delta T_{\mathrm{mean}}}{T_{0,\mathrm{mean}}}\right)\times 100$$

Δ*T*_0,mean_ represents the mean tumor volume at the start of dosing for the treatment groups.

For the NCI-H69 model, 5 × 10^6^ NCI-H69 cells were suspended in serum-free RPMI-1640 medium, combined with an equal volume of ECM gel (Sigma-Aldrich, #E1270), and implanted subcutaneously into the flanks of 6–7-week-old female nude mice (Envigo, Hsd:Athymic Nude-Foxn1^nu^). When tumors were 100–200 mm^3^ in size, 13 days after implantation, the mice were randomized and put into treatment groups. Mice were treated twice daily (BID) with oral (PO) doses of **33** at 50 or 25 mg/kg or vehicle (30% PEG400, 20% Solutol HS15, 50% Phosal 53 MCT) for 14 days or with intravenous (IV) injections of 20 mg/mkg paclitaxel every other day (QOD) for 5 doses. Tumors and body weights were measured three times per week for the duration of the study. Plasma samples were collected for PK analysis by LC–MS/MS at multiple time points shown on day 14.

For the NCI-H446 model, 5 × 10^6^ NCI-H446 cells were suspended in serum-free RPMI-1640 medium, combined with an equal volume of ECM gel (Sigma-Aldrich, #E1270), and implanted subcutaneously into the flanks of 7–8-week-old female nude mice (Envigo, Hsd:Athymic Nude-Foxn1^nu^). When tumors were 100–200 mm^3^ in size, 12 days after implantation, the mice were randomized and put into treatment groups. Mice were treated with PO doses of **33** three times daily (TID) at 100 and 25 mg/kg and BID at 100 and 50 mg/kg or with vehicle (30% PEG400, 20% Solutol HS15, 50% Phosal 53 MCT) TID for 14 days or with intravenous (IV) injections of 20 mg/mkg paclitaxel every other day (QOD) for 5 doses. Tumors and body weight were measured three times per week for the duration of the study. Plasma samples were collected from 3 animals per **33** treatment group for PK analysis by LC–MS/MS at multiple time points shown on day 14. The remaining animals were monitored for an additional 27 days with tumor and body weight measurements.

### *In Vitro* Safety and Metabolism Testing

Eurofins Discovery Services carried out the *in vitro* safety testing of **33**. The tests selected were from Eurofins product list and included (i) the KINOMEscan kinase panel assay set containing 468 kinases; (ii) the SAFETYscan47 E/IC50 predominantly cell-based functional panel that includes 24 pharmacology targets tested for both agonist and antagonist activities, 10 enzymes and transporters tested for antagonist activities, and an Ion-Profiler panel containing 6 voltage- and ligand-gated ion channels. In each case, **33** was tested at either a single concentration or at two test concentrations. **33** was also evaluated using the ADME-Tox *In Vitro* Metabolism Inhibition panel of 7 major human cytochrome P450 enzymes to determine the IC_50_ of inhibition against P450 enzymes.

## Supplementary Material





## Notes

Supplementary figures; solid-phase peptide synthesis, including methods, building blocks used, and peptide sequences; final compound purification; analysis of final compounds; solution phase synthesis methods, including custom building blocks, key intermediates, and final compounds; biochemical probe synthesis; DMPK analysis methods, including MDCK-MDR1, plasma protein binding, and kinetic solubility; MetID of Compound **2**; *In vitro* safety panel data (PDF)
SMILES of macrocycle inhibitors (CSV)

## ‡

Prelude Therapeutics, Wilmington, DE 19805

## §

Evelyn Wang Consulting, San Francisco, CA 94114.

## ∥

Stealth Biotech, San Carlos, CA.

## ⊥

Olema Oncology, San Francisco, CA 94103.

## #

New Culture, Inc., San Leandro, CA 94577.

## ∇

University of California, Davis, CA 95616.

## Abbreviations

bRo5: beyond rule-of-five
CDK: cyclin-dependent kinase
CDX: cell line-derived xenograft
CR: complete regression
cyPr: cyclopropyl
DCM: dichloromethane
DIEA: diisopropylethylamine
DMF: dimethylformamide
E2F: early region 2 binding factor
Et: ethyl
FAM: 5-carboxyfluorescein
FP: fluorescence polarization assay
HATU: hexafluorophosphate azabenzotriazole tetramethyl uronium
HBA: hydrogen bond acceptor
HBD: hydrogen bond donor
HFIP: hexafluoro-2-propanol
HP: hydrophobic patch
IMHB: intramolecular hydrogen bond
IP: intraperiotoneal
iBu: isobutyl
iPr: isopropyl
LOQ: limit of quantitation
MDCK: Madin-Darby canine kidney cells
Me: methyl
MRAIL: domain composed of methionine, arginine, alanine, isoleucine, leucine in cyclins
Papp: apparent permeability
PO: “per os” oral dosing
PPB: plasma protein binding
PPI: protein–protein interaction
QD: once daily dosing
QOD: every other day dosing
RB/RB1: retinoblastoma protein
RCM: ring-closing metathesis
RxL: motif composed of arginine, a variable moiety, and leucine
SAR: structure–activity relationship
SCLC: small cell lung cancer
T3P: propanephosphonic acid anhydride
TAT: trans-activator of transcription
TFA: trifluoroacetic acid
TGI: tumor growth inhibition
TPSA: total polar surface area
