# Supplementary material for: Orally Bioavailable Cyclin A/B RxL Inhibitors: Optimization of a Novel Class of Macrocyclic Peptides That Target E2F-High and G1–S-Checkpoint-Compromised Cancers
Source: J Med Chem. 2026 Feb 21;69(5):5441–60. doi: 10.1021/acs.jmedchem.5c02445 (PMC12990038; doi:10.1021/acs.jmedchem.5c02445)
Supplement: Supplementary file 1 [file jm5c02445_si_001.pdf]

Supporting Information

Orally Bioavailable Cyclin A/B RxL Inhibitors: optimization of a novel class of macrocyclic peptides that target E2F high and G1–S-checkpoint compromised cancers

Justin A. Shapiro,[1‡] Nathan J. Dupper,[1‡] Breena Fraga-Walton,[1] Andrew T. Bockus,[1] Siegfried S.F. Leung,[1] Kai Yang,[1] Chinmay Bhatt,[1] Megan K. DeMart,[1] Miguel P. Baldomero,[1] Luis Hernandez,[1] Gabriel Fung,[1] Sammy Metobo,[1] Steven Xie,[1] Bryan M. Lent,[1] David C. Spellmeyer,[1] Joshua Luna,[1] Dalena Hoang,[1] Manesh Chand,[1] Yuliana Gritsenko,[1] Catherine E. Gleason,[1] Frances Hamkins-Indik,[1] Jie Zheng,[1] Ranya Odeh,[1] Meisam Nosrati,[1] Daphne He,[1] Ramesh Bambal,[1] Peadar Cremin,[1] Jinshu Fang,[1] Bernard Levin,[1] Evelyn W. Wang,[1] Marie Evangelista,[1] David Earp,[1] Constantine Kreatsoulas,[1] Rajinder Singh,[1] Pablo D. Garcia,[1] James B. Aggen[1]*

[1]*Circle Pharma, 169 Harbor Way, South San Francisco, CA 94080*

[‡]*These authors contributed equally to this work.*

Corresponding Author Email: jim.aggen@circlepharma.com

Contents

Supplementary Figures 2

Solid Phase Peptide Synthesis 3

  SPPS Methods 3

  Table S1: SPPS Building Blocks Used 5

  Table S2: SPPS Sequences 6

Final Compound Purification 6

Analysis of Final Compounds 7

  Table S3: Analytical Data of SPPS Compounds 7

Solution Phase Synthesis 8

Synthesis of Custom Building blocks 9
Method A – Synthesis of Macrocyclic Core 24
Method B – Hydrogenation 35
Method C – Suzuki Coupling 40
Method D – Dipeptide Addition 43
Table S4: HPLC purity of Solution Phase Compounds 54
Biochemical Probe Synthesis 55
Spectra For the Synthesis of Compound 23 57
DMPK Assays 68
Met ID of Compound 2 70
Table S5: WI-38 GI_{50} Values 71
Table S6: Mouse Liver Microsome Stability 72
In Vitro Safety Panels 72
References 101

Supplementary Figures

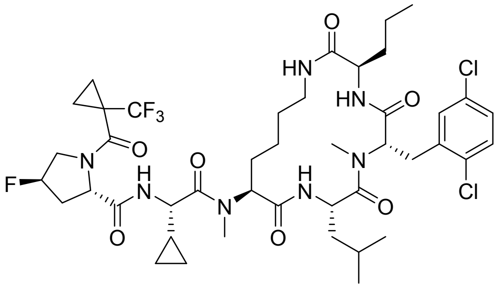

Compound 26 (from ref. 1)
ALogP = 4.5, CycA FP IC_{50} = 0.188 μM
H1048 GI_{50} = 0.096 μM, ***MDCK P_{app} A->B = 2.3 x 10^6 cm/s***

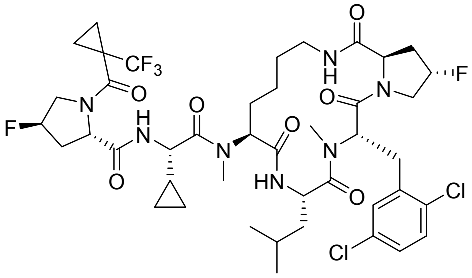

Compound 30 (from ref. 1)
ALogP = 4.1, CycA FP IC_{50} = 0.132 μM
H1048 GI_{50} = 0.176 μM, ***MDCK P_{app} A->B = 0.3 x 10^6 cm/s***

Supplementary Figure 1: Match-pair analysis of bridging residue between lariat *N-ε* and Phenylalanine residue. Despite reduction in H-bond donor count going from Compound 26 to Compound 30 (compound numbers taken from ref. 1), the MDCK value of 30 is dramatically reduced, suggesting the importance of macrocycle conformation in membrane permeability.

Solid Phase Peptide Synthesis

SPPS Methods

All peptides were synthesized using a Biotage Syro II automated peptide synthesizer. The reaction was were performed on a 50 µmol scale using 50-100 mg of 2-chlorotritylchloride polystyrene resin.

CTC Resin Loading: Fmoc-AA-OH (4 Equiv.) was dissolved in 1.0 mL of anhydrous NMP. Neat DIEA (8 Equiv.) was added to the Fmoc-AA-OH solution. The solution was dispensed in a peptide reactor vessel containing 100 mg of 2-chlorotrytl chloride (CTC) resin and was agitated for 2 hours at room temperature. The Fmoc-AA-OH solution was drained then the resin was washed with 1.0 mL DMF three times. Unreacted CTC resin was capped with 1.0 mL solution of methanol:DMF (50:50), and DIEA (8 Equiv.) for 10 minutes at room temperature. The methanol solution was drained then the resin was washed with 1.0 mL DMF three times.

Removal of Fmoc protecting group: A mixture of piperidine:DMF (20:80, 1 mL) was added to the resin and agitated for 10 to 15 minutes at room temperature. The piperidine solution was drained then the resin was washed with 1.0 mL DMF three times.

HATU Coupling: A solution of Fmoc-AA-OH (4 Equiv.), HATU (4 Equiv.), and DIEA (8 Equiv.) in 1.0 mL of anhydrous NMP was prepared. The mixture was allowed to react at room temperature for 5 minutes then was added to the resin and was agitated at 35 to 45°C for 10 to 90 minutes. The mixture was drained then the resin was washed with 1.0 mL of DMF three times.

HATU Coupling onto Secondary Amines: When coupling onto secondary amines, The HATU Coupling Method (above) was repeated.

On-Resin Mitsunobu Alkylation: This procedure is a modified version of the procedure outlined by Chatterjee et al.[1] After Fmoc Deprotection, 2,6-lutidine (6 Equiv.) dissolved in 0.5 mL of anhydrous DCE was added to the resin. 2-nitrobenzenesulfonyl chloride (5 Equiv.) dissolved in 0.5 mL anhydrous toluene was added to the resin then was agitated at 40 to 45°C for 10 to 15

minutes. The mixture was drained then the resin was washed with 1.0 mL of anhydrous toluene three times. The method was repeated twice.

Triphenylphosphine (10 Equiv.) dissolved in 0.7 mL anhydrous toluene was added to the resin. The appropriate primary alcohol (20 Equiv. Of methanol, ethanol, propanol, butanol, or other) was added to the resin suspension. Azodicarboxylate (10 Equiv.) was added to the resin and the suspension was agitated at 35 to 45°C for 15 to 30 minutes. The mixture was drained then the resin was washed with 1.0 mL of anhydrous DMF three times. The method was repeated twice.
2-mercaptoethanol (5 Equiv.) and 1,8-Diazabicyclo[5.4.0]undec-7-ene (5 Equiv.) in 1.0 mL NMP was added to the resin and was agitated at 35 to 45°C for 15 to 30 minutes. The mixture was drained then the resin was washed with 1.0 mL of anhydrous DMF three times. The method was repeated twice.

Resin Cleavage: For peptide sequences with NMeKMe, KMe, and K, a solution of 30% TFA and 5% TIPS in DCM (2 mL) was added to 50-100 mg of resin in a solid phase reaction vessel. For peptide sequences containing **KMtt**, a solution of 90% HFIP, 2% TIPS in DCM (2 mL) was added to 50-100 mg of resin in a solid phase reaction vessel. The contents of the vessel were shaken for one hour. The liquid phase of the reaction was filtered into a 50 mL conical vial. The cleaved resin was washed with an additional DCM (2 mL) and the wash was collected in the conical vial. Toluene (2 mL) was added to the cleaved peptide solution and the solution was concentrated. Linear peptides could be purified at this stage or taken to the cyclization step without additional purification.

Cyclization of Linear Peptides: The deprotected linear product (50 μmol) was dissolved in NMP (500 uL), DIEA (250 uL), and DCM (0.75 mL). T3P (31uL, 3 Equiv.) is added, the solution was shaken for 1-10 minutes at room temperature. Reaction completion was confirmed via LCMS analysis.

Yamaguchi Cyclization of Compound 5: The deprotected linear product (25 μmol) was dissolved in TH F(500 uL). Then, 2,4,6-Trichlorobenzoyl chloride (6.0 mg, 3.8 μL, 1 Eq, 25 μmol), TEA (7.3 mg, 10 μL, 2.9 Eq, 72 μmol) and DMAP (0.3 mg, 0.1 Eq, 2 μmol) were added

sequentially. The reaction was stirred overnight at ambient temperature. The reaction mixture was directly purified by RP-HPLC.

Table S1: SPPS Building Blocks Used

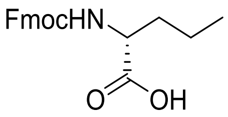	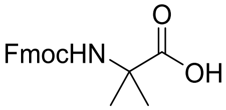	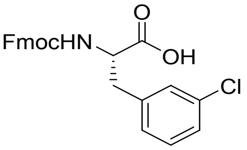	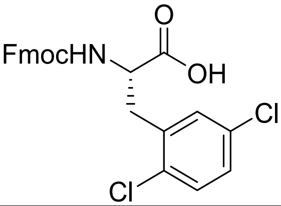
**nva**	**Aib**	**3ClF**	**25ClF**
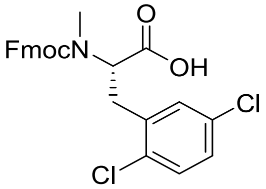	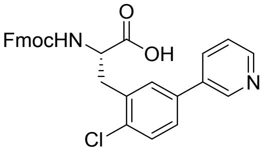	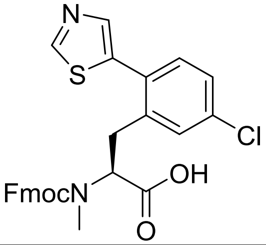	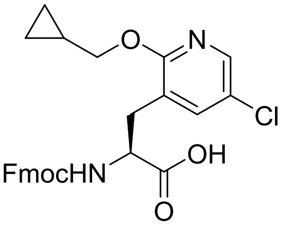
**NMe25ClF**	**23Pyr5ClF**	**NMe2H55ClF**	**Phe0042**
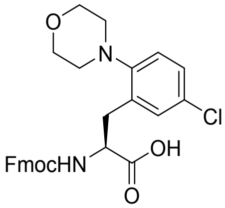	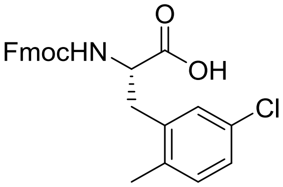	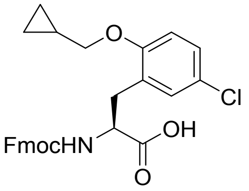	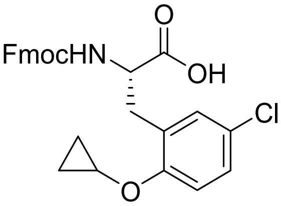
**Phe0064**	**2Me5ClF**	**5Cl2cPrF**	**Phe0034**
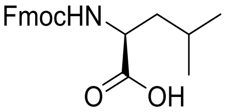	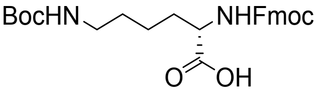	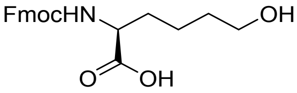	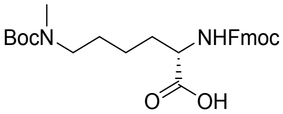
**L**	**K**	**LyxO**	**KMe**
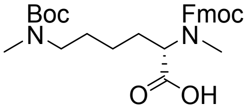	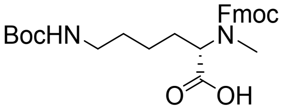	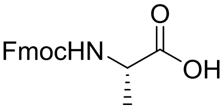	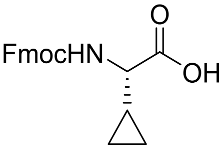
**NMeKMe**	**NMeK**	**A**	**cPrG**
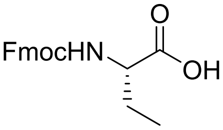	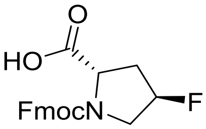	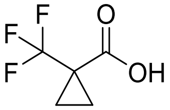	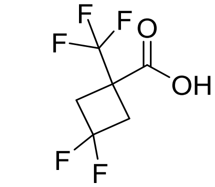
**Abu**	**AA0011**	**Acd0317**	**Acd0588**

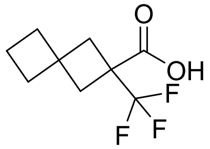

Acd0540

Table S2: SPPS Sequences

Comp #	R1	R2	R3	R4	R5	R6	R7
**2**	Aib	25ClF[A]	L	K[A]	cPrG	AA0011	Acd0540
**3**	Aib	25ClF[A]	L	K[A]	cPrG	AA0011	Acd0588
**4**	nva	3ClF[A]	L	K[A]	A	Abu	Acd0317
**5**	nva	3ClF[A]	L	LyxO[A]	A	Abu	Acd0317
**6**	nva	3ClF[A]	L	KMe[A]	A	Abu	Acd0317
**7**	Aib	2Me5ClF[A]	L	K[A]	cPrG	AA0011	Acd0540
**8**	Aib	Phe0034[A]	L	NMeK	cPrG	AA0011	Acd0540
**9**	Aib	NMe2H55ClF	L	NMeK	cPrG	AA0011	Acd0540
**10**	Aib	Phe0064[A]	L	NMeK	cPrG	AA0011	Acd0540
**11**	Aib	23Pyr5ClF[A]	L	NMeK	cPrG	AA0011	Acd0540
**12**	Aib	5Cl2cPrF[A]	L	NMeK	cPrG	AA0011	Acd0540
**13**	Aib	Phe0042[A]	L	NMeK	cPrG	AA0011	Acd0540
**14**	Aib	NMe25ClF	L	NMeKMe	cPrG	AA0011	Acd0540
**15**	nva	NMe25ClF	L	K[A]	cPrG	AA0011	Acd0540
**16**	nva	NMe25ClF	L	KMe[A]	cPrG	AA0011	Acd0540
**17**	nva	NMe25ClF	L	K[A]	cPrG	AA0011	Acd0588
**18**	nva	NMe25ClF	L	KMe[A]	cPrG	AA0011	Acd0588

[A] Mitsonobu

Final Compound Purification

Cyclic compounds were purified on a Xbridge (C18 column 10mm x 150mm) using a mass-triggered prep Waters HPLC system in a dual column set up. Components of the Waters HPLC system include: Waters 2767 Sample Manager, Waters 1525 Binary HPLC Pump, Waters 2545 Binary Gradient Module, Waters SFO System Fluidics Organizer, 515 HPLC Pump, Waters QDA

and Waters 2998 Photodiode Array Detector. **Solvent A**: Water (+0.1% TFA). **Solvent B**: MeCN (+0.1% TFA). An exemplary method can be found below:

Time (min)	Flowrate (mL/min)	%A	%B
0	15.00	80	20
1.60	15.00	80	20
2.00	20.00	80	20
7.50	20.00	45	55
8.00	20.00	0	100
9.50	20.00	0	100
9.75	20.00	80	20
10.00	20.00	80	20

Analysis of Final Compounds

Method A: Purity of samples were analyzed using as Agilent Infinitylab LC/MSD with 6140 Quadrapole LCMS equipped with a BEH C18 Column (4.6x100mm; 3.5µm) using Solvent A (Water +0.1% TFA) and Solvent B (MeCN + 0.1% TFA). A ramp from 10-100% Solvent B was run over 8 min (flowrate: 1.2 mL/min). Ionization Mode: ESI+.

Method B: Purity of samples were analyzed using a Waters UPLC Aquity I-class with Acquity QDa equipped with a BEH C18 Column (4.6x100mm; 3.5µm) using Solvent A (Water +0.1% formic acid) and Solvent B (MeCN + 0.1% formic acid). A ramp from 10-100% Solvent B was run over 1 min (flowrate: 1.2 mL/min). Ionization Mode: ESI+.

Method C: Purity of samples were analyzed using a Waters UPLC Aquity H-class with SQ Detector 2 equipped with a BEH C18 Column (2.1x50mm; 1.7µm) using Solvent A (Water), Solvent B (MeCN), and Solvent C (0.4% TFA in Water). A ramp from 30-85% Solvent B + 5% Solvent C was run over 6 min (flowrate: 0.5 mL/min). Ionization Mode: ESI+.

Table S3: Analytical Data of SPPS Compounds

Compound ID	Analytical Method	Rt (min)	% Purity	Yield (mg)	*m/z* observed	Calcd *m/z*

**5**	A	7.11	91	0.6	843.9	843.4
**6**	A	6.87	99	13.0	856.6	856.43
**7**	B	0.812	99	46.95	952.43	952.46
**8**	B	0.830	99	45.43	994.40	994.48
**9**	B	0.789	96	42.47	1021.48	1021.43
**10**	B	0.793	99	9.4	1023.57	1023.5
**11**	B	0.748	88	2.2	1015.59	1015.48
**12**	B	0.843	99	1.60	1008.53	1008.49
**13**	B	0.857	99	4.13	1009.37	1009.49
**14**	B	0.822	99	29.76	986.97	986.43
**15**	B	0.861	99	22.64	986.53	986.43
**16**	B	0.910	99	17.01	1000.51	1000.44
**17**	B	0.800	99	5.27	982.44	982.38
**18**	B	0.838	99	46.23	996.39	996.39

SPPS compounds **2-4** were reported previously.[1]

Solution Phase Synthesis

All reactions were performed under open to air unless otherwise noted. Anhydrous solvents were used directly from commercial vendors. All chemicals were purchased from commercial vendors unless otherwise noted. Reactions were monitored using a Waters ACQUITY UPLC system coupled to a Waters SQ Detector 2 mass spectrometer with a Waters Acquity UPLC BEH C18 column (1.7 µm, 2.1 x 50 mm) using water (+0.5% Formic Acid) and MeCN (+0.5% Formic Acid) as mobile phases. NMR spectra were recorded with Bruker 400 Advance and Bruker 500 Advance instruments, Data for ^{1}H NMR spectra are reported as follows: chemical shift (δ ppm), multiplicity, coupling constant (Hz), and integration. The following abbreviations are used for the multiplicities: s = singlet; d = doublet; t = triplet; q = quartet; m = multiplet; br = broad; app = apparent.

Synthesis of Custom Building blocks

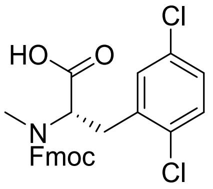

(S)-2-((((9H-fluoren-9-yl)methoxy)carbonyl)(methyl)amino)-3-(2,5-dichlorophenyl)propanoic acid (NMe25ClF) was synthesized as previously reported.[1]

LCMS: MS (ESI) mass calcd. for $C_{25}H_{22}Cl_2NO_4$: 470.08 m/z; found 470.00 $[M+H]^+$.

^{1}H NMR (400 MHz, DMSO) δ 13.12 (br. zzs, 1H), 7.87 (t, *J* = 7.4 Hz, 2H), 7.54 (dt, *J* = 14.3, 7.3 Hz, 2H), 7.49 – 7.24 (m, 7H), 5.76 (s, 1H), 4.89 (app. ddd, *J* = 23.3, 11.0, 4.6 Hz, 1H), 4.33 – 4.06 (m, 3H), 3.39 – 2.97 (m, 2H), 2.87 – 2.75 (m, , 3H). Reported as a mixture of rotamers.

^{13}C NMR (101 MHz, DMSO) δ 171.94, 171.77, 156.19, 155.91, 144.15, 144.07, 143.98, 141.17, 141.14, 141.11, 138.19, 138.09, 132.56, 132.52, 132.04, 132.00, 131.60, 131.51, 131.34, 131.30, 128.93, 128.81, 128.20, 128.15, 127.60, 125.45, 125.38, 125.26, 120.62, 67.48, 58.93, 58.77, 55.40, 47.07, 46.92, 40.60, 40.39, 40.19, 39.98, 39.77, 39.56, 39.35, 32.63, 32.48, 32.27, 26.82. Reported as a mixture of rotamers.

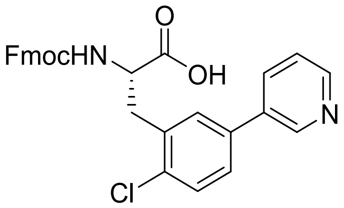

(S)-2-((((9H-fluoren-9-yl)methoxy)carbonyl)amino)-3-(2-chloro-5-(pyridin-3-yl)phenyl)propanoic acid (23Pyr5ClF) was synthesized as previously reported.[2]

LCMS: MS (ESI) mass calcd. for $C_{29}H_{24}ClN_2O_4$: 499.14 m/z; found 499.1 $[M+H]^+$.

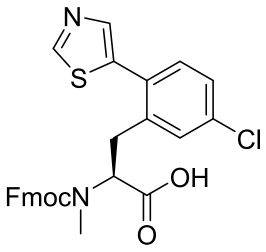

(S)-2-((((9H-fluoren-9-yl)methoxy)carbonyl)(methyl)amino)-3-(5-chloro-2-(thiazol-5-yl)phenyl)propanoic acid (NMe2H55ClF) The starting material, (S)-2-((((9H-fluoren-9-yl)methoxy)carbonyl)amino)-3-(5-chloro-2-(thiazol-5-yl)phenyl)propanoic acid, was synthesized as previously reported.[2]

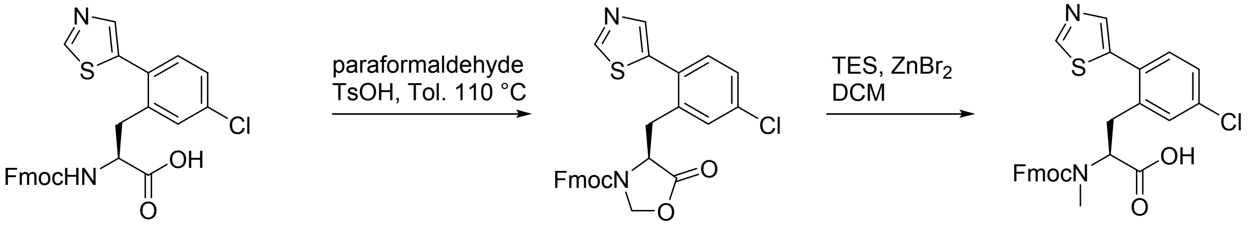

NMe2H55ClF: A mixture of (2S)-3-[5-chloro-2-(1,3-thiazol-5-yl)phenyl]-2-{[(9H-fluoren-9-ylmethoxy)carbonyl] amino}propanoic acid (10.0 g, 19.8 mmol, 1 Eq.), paraformaledyde (2.97g, 99 mmol, 5 Eq.) and 4-methylbenzene-1-sulfonic acid (341 mg, 1.98 mmol, 0.1 Eq.) in Toluene (100 mL) was stirred overnight at 90 ºC. The resulting mixture was filtered, concentrated. The resulting crude product was used in the next reaction without further purification.

LCMS: MS (ESI) mass calcd. for $C_{28}H_{22}ClN_2O_4S$: 517.10 m/z; found 517.00 $[M+H]^+$.

To a stirred mixture of 9H-fluoren-9-ylmethyl (4S)-4-{[5-chloro-2-(1,3-thiazol-5-yl)phenyl]methyl}-5-oxo-1,3-oxazolidine-3-carboxylate (10 g, 19.34 mmol, 1 Eq.) and $ZnBr_2$ (17.42 g, 77.37 mmol, 4 Eq.) in DCM (150 mL) was added TES (9.00 g, 77.37 mmol, 4 Eq.) dropwise at 0 ºC. The resulting mixture was stirred for 7 days at room temperature. The resulting mixture was diluted with EtOAc (100 mL), filtered, and concentrated to afford a crude residue.The residue was purified by RP chromatography (C18, 45 to 80% MeCN+0.1% formic acid in water+0.1% formic acid) This resulted in (2S)-3-[5-chloro-2-(1,3-thiazol-5-yl)phenyl]-2-{[(9H-fluoren-9-ylmethoxy)carbonyl] (methyl)amino}propanoic acid (6.32 g, 12.17 mmol, 62.9%) as a white solid.

LCMS: MS (ESI) mass calcd. for $C_{28}H_{24}ClN_2O_4S$: 519.12 m/z; found 519.01 $[M+H]^+$.

^{1}H NMR: (400 MHz, DMSO-d6): δ 2.61 (s, 3H), 2.99-3.24 (m, 1H), 3.37-3.50 (m, 1H), 4.00-4.30 (m, 3H), 4.61 (dd, J = 11.3, 4.7 Hz, 1H), 7.22-7.62 (m, 9H), 7.82-8.02 (m, 3H), 9.18 (d, J = 8.1 Hz, 1H), 12.97 (s, 1H).

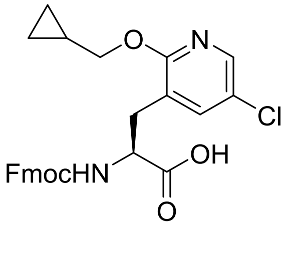

(S)-2-((((9H-fluoren-9-yl)methoxy)carbonyl)amino)-3-(5-chloro-2-(cyclopropylmethoxy)pyridin-3-yl)propanoic acid (Phe0042) was synthesized as previously reported.[2]

LCMS: MS (ESI) mass calcd. for $C_{27}H_{26}ClN_2O_5$: 493.16 m/z; found 493.0 $[M+H]^+$.

(S)-2-((((9H-fluoren-9-yl)methoxy)carbonyl)amino)-3-(5-chloro-2-morpholinophenyl)propanoic acid (Phe0064)

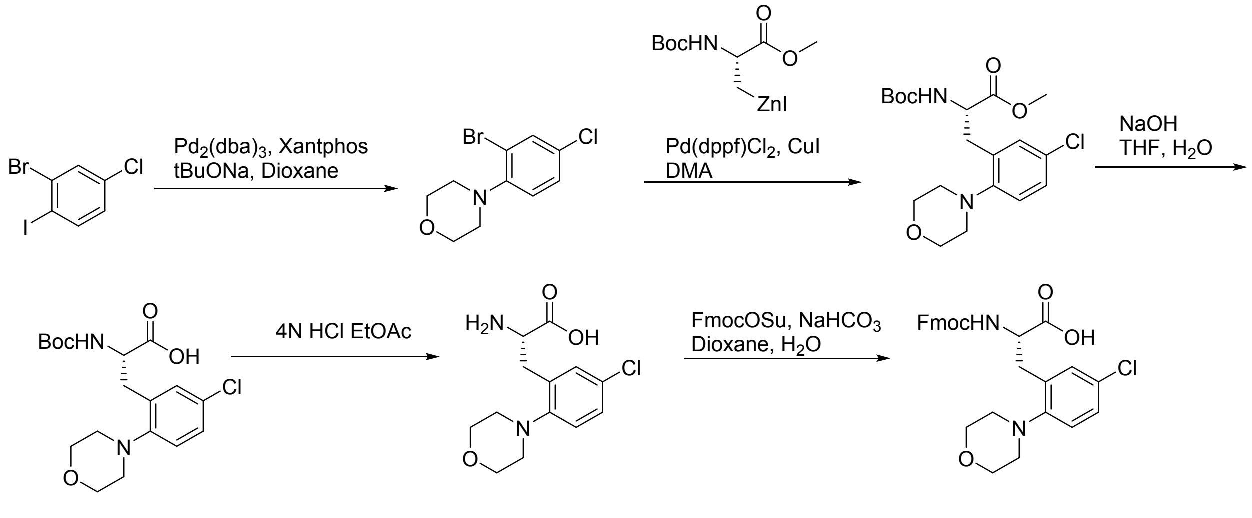

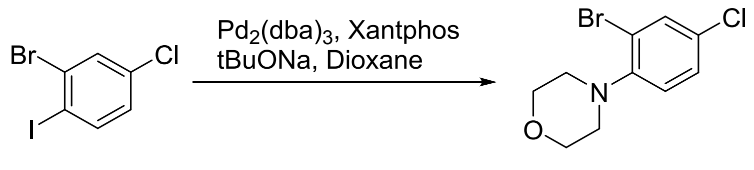

4-(2-bromo-4-chlorophenyl)morpholine: Into a 500ml round-bottom flask, 2-bromo-4-chloro-1-iodobenzene (25 g, 78.8 mmol, 1 Eq.), morpholine (6.86 g, 78.8 mmol, 1 Eq.), Xantphos (18.23 g, 31.5 mmol, 0.4 Eq.), $Pd_2(dba)_3$ (14.5 g, 15.8 mmol, 0.2 Eq.) and *t*-BuONa (22.71 g, 236.3 mmol, 3 Eq.) were dissolved / suspended in 1,4-dioxane (100 mL) at room temperature under a nitrogen atmosphere. The resulting mixture was stirred for 2 hr at 80 ºC. The reaction was quenched with ice water (500 ml) and extracted with EtOAc (3 x 200 mL). The combined organic layers were washed with brine (300 mL), dried with anhydrous Na_2SO_4, filtered and concentrated in vacuum to give the crude product. The residue was purified by silica gel column chromatography (Pet. 0-10% EtOAc in pet ether) to afford 4-(2-bromo-4-chlorophenyl)morpholine (14 g, 50.6 mmol, 64.26%) as a yellow solid.

LCMS: MS (ESI) mass calcd. for $C_{10}H_{12}BrClNO$: 275.98 m/z; found 275.90 $[M+H]^+$.

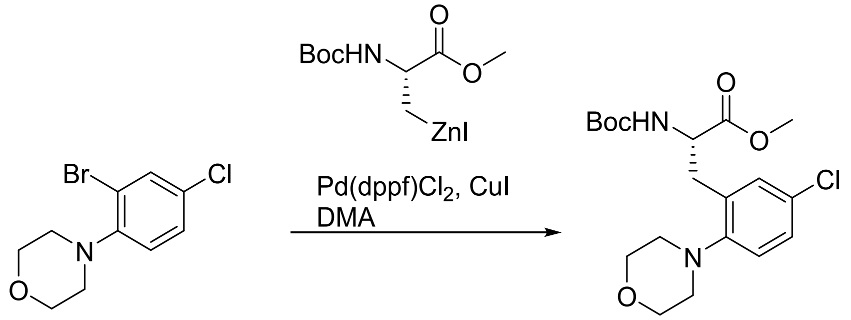

Methyl (S)-2-((tert-butoxycarbonyl)amino)-3-(5-chloro-2-morpholinophenyl)propanoate: To a suspension of Zn (5.63 g, 86.5 mmol, 1.7 Eq.) in DMA (40 mL) was added ethylene dibromide (1.3 g, 7.1 mmol, 0.14 Eq.) in one portion under a nitrogen atmosphere. Then TMSCl (0.6 g, 5.1 mmol, 0.1 Eq.) was added slowly. The zinc was allowed to activate for 30 min at room temperature. A solution of methyl (2R)-2-[(tert-butoxycarbonyl)amino]-3-iodopropanoate (25.1 g, 76.4 mmol, 1.5 Eq.) in DMA (40 mL) was added dropwise (over 30 min) to maintain temperature below 50 ºC. The resulting mixture was stirred at room temperature for 2 hr. the zincate solution was next added via a cannula to a solution of 4-(2-bromo-4-chlorophenyl)morpholine (14 g, 50.9 mmol, 1 Eq.), $Pd(dppf)Cl_2.CH_2Cl_2$ (8.3 g, 10.1 mmol, 0.2 Eq.) and CuI (1.9 g, 10.1 mmol, 0.2 Eq.) in DMA (80 mL),The mixture was stirred at room temperature for 5 min, then the mixture was heated and stirred at 80 ºC for 2 hr under nitrogen. The mixture was quenched with ice-water (500 mL) and extracted with EtOAc (3 x 200 mL). The organic layers were combined and washed with brine (300 mL), dried with anhydrous Na_2SO_4, filtered and concentrated in vacuum to give the crude

product. The product was purified by silica gel chromatography (0-30% EtOAc in pet. ether) to give methyl (2S)-2-[(tert-butoxycarbonyl)amino]-3-[5-chloro-2-(morpholin-4-yl)phenyl]propanoate (3.2 g, 8.0 mmol, 15.85%) as a yellow oil.

LCMS: MS (ESI) mass calcd. for $C_{19}H_{28}ClN_2O_5$: 399.17 m/z; found 399.05 $[M+H]^+$.

^{1}H NMR: (400 MHz, DMSO-d6): δ 1.30 (s, 9H), 2.74 (ddd, J = 13.6, 9.7, 5.8 Hz, 3H), 2.87 (ddd, J = 11.8, 5.6, 3.2 Hz, 2H), 3.21 (dd, J = 13.7, 4.3 Hz, 1H), 3.64 (s, 3H), 3.73 (tt, J = 11.0, 5.6 Hz, 4H), 4.46 (ddd, J = 10.2, 8.3, 4.2 Hz, 1H), 7.20 (d, J = 8.4 Hz, 1H), 7.24 – 7.33 (m, 3H).

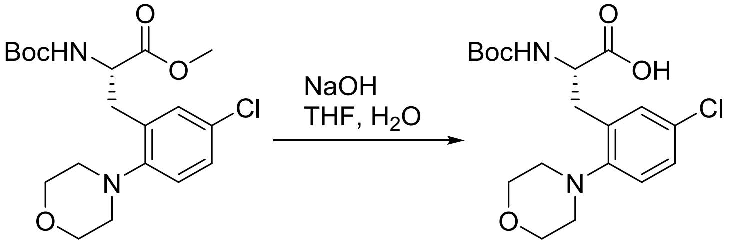

(S)-2-((tert-butoxycarbonyl)amino)-3-(5-chloro-2-morpholinophenyl)propanoic acid: Into a 100 mL round bottom was placed methyl (2S)-2-[(tert-butoxycarbonyl)amino]-3-[5-chloro-2-(morpholin-4-yl)phenyl]propanoate (3.2 g, 8.0 mmol, 1 equiv) in THF (40 mL). NaOH (1.6 g, 40.1 mmol, 5 equiv) in H_2O (8 mL) was added at 0 ºC. The resulting mixture was stirred for 2 hr at room temperature. The mixture was acidified to ~4 pH with 2N HCl (aq.). The acidified mixture was extracted with EtOAc (3 x 200 mL). The organic layers were combined, washed with brine and dried over with anhydrous Na_2SO_4, filtered, and the filtrate was concentrated under reduced pressure. The residue was purified by silica gel column chromatography (0-50% EtOAc in pet. ether) to afford (2S)-2-[(tert-butoxycarbonyl)amino]-3-[5-chloro-2-(morpholin-4-yl)phenyl]propanoic acid (3 g, 7.8 mmol, 97.17%) as a yellow oil.

LCMS: MS (ESI) mass calcd. for $C_{18}H_{26}ClN_2O_5$: 385.16 m/z; found 385.05 $[M+H]^+$.

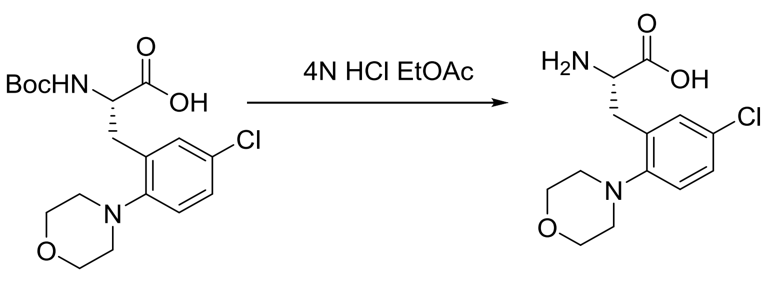

(S)-2-amino-3-(5-chloro-2-morpholinophenyl)propanoic acid: Into a 100 ml round bottom was placed (2S)-2-[(tert-butoxycarbonyl)amino]-3-[5-chloro-2-(morpholin-4-yl)phenyl]propanoic acid (3 g, 7.8 mmol, 1 equiv) in EtOAc (20 mL), 4N HCl in EtOAc (20 mL) was added at room temperature under an atmosphere of nitrogen. The resulting mixture was stirred for 2hr at room temperature. The solvent was removed under reduced pressure and crude product was used in the next step directly.

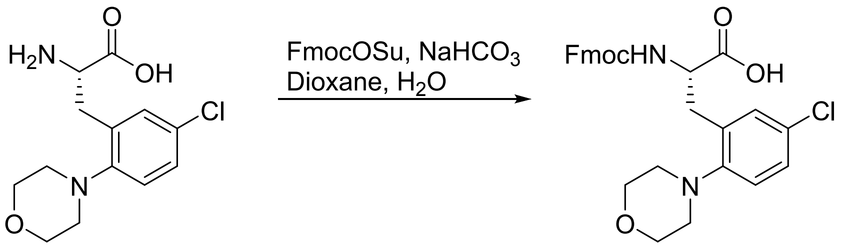

Phe0064: The above crude product was dissolved in 1,4-dioxane (30 mL) and H_2O (10 mL), $NaHCO_3$ (3.69 g, 43.90 mmol, 5.6 equiv) and FmocOSu (3.58 g, 7.02 mmol, 0.9 equiv) were added at room temperature. The resulting mixture was stirred at room temperature for 16hr. The mixture was acidified to pH ~2 with 2N HCl (aq) and extracted with EtOAc (3 x 100 mL). The organic layers were combined and dried over anhydrous Na_2SO_4. After filtration, the filtrate was concentrated under reduced pressure. The residue was purified by RP chromatography(C18, 10 to 50% MeCN+0.1% TFA in water+0.1% TFA). This resulted in (2S)-3-[5-chloro-2-(morpholin-4-yl)phenyl]-2-{[(9H-fluoren-9-ylmethoxy)carbonyl]amino}propanoic acid (1.63 g, 3.2 mmol, 41.0%) as a white solid after lyophilization.

LCMS: MS (ESI) mass calcd. for $C_{28}H_{28}ClN_2O_5$: 507.17 m/z; found 507.10. $[M+H]^+$.

^{1}H NMR: (400 MHz, DMSO-d6): δ 2.77 (dtd, J = 34.4, 11.6, 10.8, 4.1 Hz, 5H), 3.26 (dd, J = 13.7, 4.2 Hz, 1H), 3.69 (p, J = 6.1, 4.7 Hz, 4H), 4.04 – 4.32 (m, 3H), 4.46 (td, J = 9.8, 4.1 Hz, 1H), 7.18 (d, J = 8.5 Hz, 1H), 7.23 – 7.36 (m, 4H), 7.41 (t, J = 7.5 Hz, 2H), 7.64 (dd, J = 22.9, 8.0 Hz, 3H), 7.88 (d, J = 7.5 Hz, 2H), 12.72 (s, 1H).

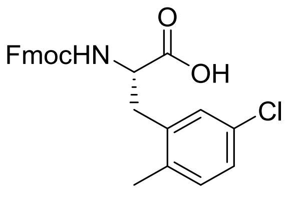

(S)-2-((((9H-fluoren-9-yl)methoxy)carbonyl)amino)-3-(5-chloro-2-methylphenyl)propanoic acid (2Me5ClF) was synthesized as previously reported.[2]

LCMS: MS (ESI) mass calcd. for $C_{25}H_{23}ClNO_4$: 436.13 m/z; found 436.13 $[M+H]^+$.

(S)-2-((((9H-fluoren-9-yl)methoxy)carbonyl)amino)-3-(5-chloro-2 (cyclopropylmethoxy)phenyl)propanoic acid (5Cl2cPrF):

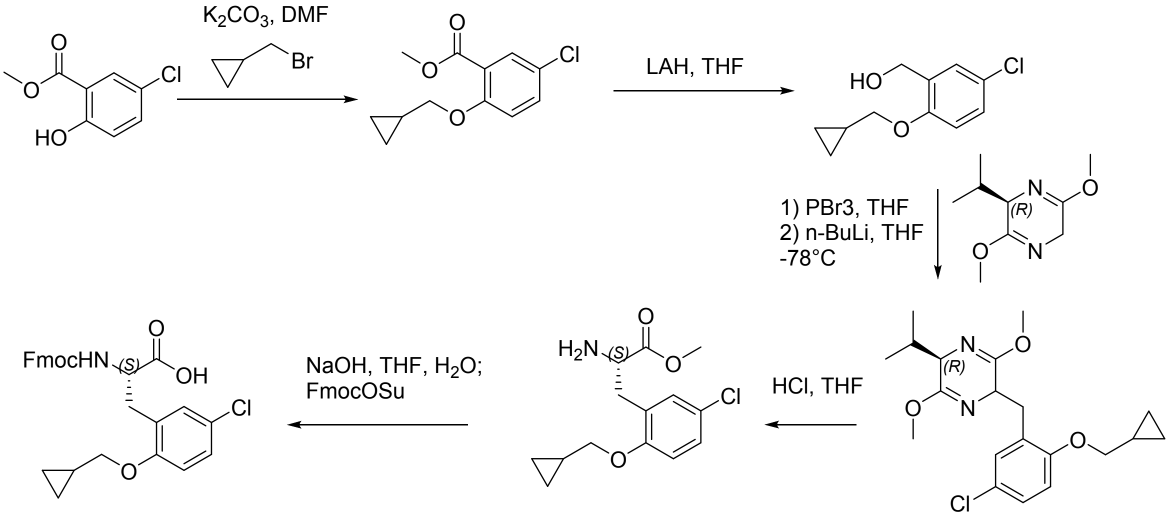

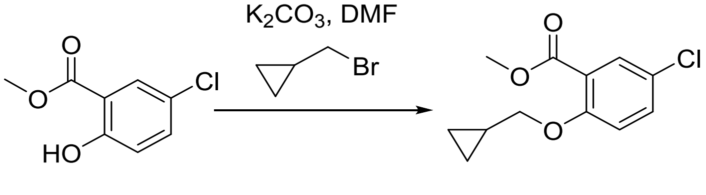

methyl 5-chloro-2-(cyclopropylmethoxy)benzoate: A solution of methyl 5-chloro-2-hydroxybenzoate (10 g, 53.59 mmol, 1 equiv), (bromomethyl)cyclopropane (7.24 g, 53.59 mmol, 1equiv) and K_2CO_3 (8.89 g, 64.31 mmol, 1.2 equiv) in DMF (150 mL) was stirred for 16hr at 40°C. The resulting mixture was diluted with water (300 mL) and extracted with EtOAc (3 x 100 mL). The organic layers were combined and dried over anhydrous Na_2SO_4. After filtration, the filtrate was concentrated under reduced pressure. The residue was purified by RP chromatography (C18, 0-100% MeCN+0.1% TFA in water+0.1%TFA). Methyl 5-chloro-2-(cyclopropylmethoxy) benzoate (9.8 g, 40.7 mmol, 75.9%) as a white solid after lyophilization.

LCMS: MS (ESI) mass calcd. for $C_{12}H_{14}ClO_3$: 241.07 m/z; found 241.06 $[M+H]^+$.

^{1}H NMR: (300 MHz, DMSO-d_6): δ 0.29 – 0.45 (m, 2H), 0.47 – 0.61 (m, 2H), 1.11 – 1.30 (m, 1H), 3.81 (s, 2H), 3.92 (d, J = 6.5 Hz, 2H), 7.16 (dd, J = 9.0, 1.3 Hz, 1H), 7.55 (dt, J = 8.9, 2.0 Hz, 1H), 7.63 (d, J = 2.7 Hz, 1H).

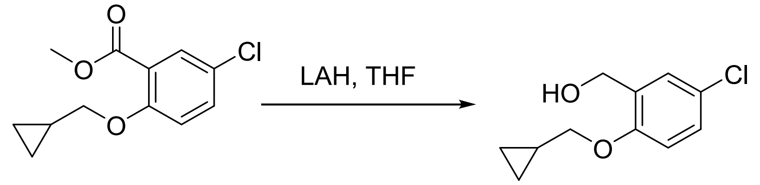

(5-chloro-2-(cyclopropylmethoxy)phenyl)methanol: A solution of methyl 5-chloro-2-(cyclopropylmethoxy) benzoate (9.8 g, 40.7 mmol, 1 equiv) and $LiAlH_4$ (3.09g, 81.4 mmol, 2 equiv) in THF (200 mL) was stirred for 3hr at 0°C under nitrogen atmosphere. The reaction was carefully quenched by the slow addition of water at 0°C. The resulting mixture was extracted with EtOAc (3 x 100 mL). The combined organic layers were washed with brine (50 mL), dried over anhydrous Na_2SO_4. After filtration, the filtration was concentrated under reduced pressure to afford [5-chloro-2-(cyclopropylmethoxy) phenyl] methanol (8.6g 40.7 mmol, quant.) as a light yellow oil. The resulting compound was used without further purification.

LCMS: MS (ESI) mass calcd. for $C_{11}H_{14}ClO_2$: 213.07 m/z; found 213.06 $[M+H]^+$.

^{1}H NMR: (400 MHz, DMSO-d_6): δ 0.24 – 0.37 (m, 2H), 0.47 – 0.62 (m, 2H), 1.13 – 1.26 (m, 1H), 3.83 (d, J = 6.8 Hz, 2H), 4.51 (s, 2H), 5.18 (s, 1H), 6.92 (d, J = 8.7 Hz, 1H), 7.20 (dd, J = 8.7, 2.8 Hz, 1H), 7.31 – 7.41 (m, 1H).

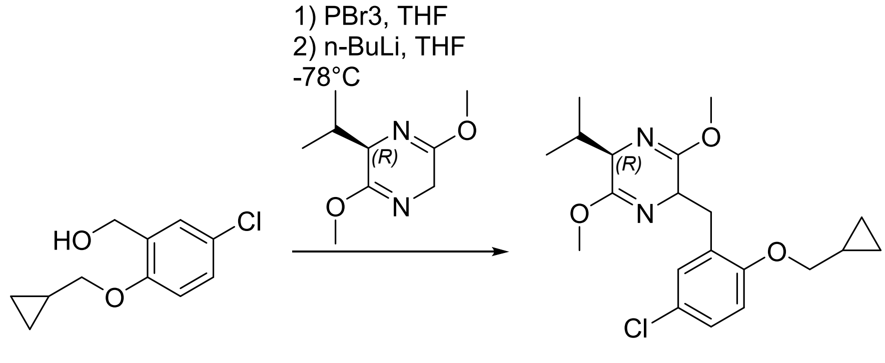

(5R)-2-(5-chloro-2-(cyclopropylmethoxy)benzyl)-5-isopropyl-3,6-dimethoxy-2,5-dihydropyrazine: A solution of [5-chloro-2-(cyclopropylmethoxy) phenyl] methanol (5 g, 23.51 mmol, 1 equiv) and PBr3 (8.27 g, 30.5 mmol, 1.3 equiv) in THF (100 mL) was stirred for 15 min at 0°C under an atmosphere of nitrogen. The reaction was quenched with water (25 mL) at 0°C. The resulting mixture was extracted with EtOAc (3 x 100mL). The combined organic layers were dried over anhydrous Na_2SO_4. After filtration, the filtrate was concentrated under reduced pressure. 2-(bromomethyl)-4-chloro-1-(cyclopropylmethoxy) benzene (6.4g, 23.51 mmol, quant.)

A solution of (3R)-3-isopropyl-2,5-dimethoxy-3,6-dihydropyrazine (3.68 g, 19.96 mmol, 1.1equiv) and nBuLi (13.61 mL, 27.2 mmol, 1.5equiv) in THF (100 mL) was stirred for 1hr at -78°C under an atmosphere of nitrogen. 2-(bromomethyl)-4-chloro-1-(cyclopropylmethoxy) benzene (5 g, 18.14 mmol, 1equiv) in THF was added to the solution and stirred for 30 min. The reaction was quenched with water (50mL) at 0°C.The resulting mixture was extracted with EtOAc (3 x 50mL). The combined organic layers were dried over anhydrous Na_2SO_4. After filtration, the filtrate was concentrated under reduced pressure to afford (5R)-2-{[5-chloro-2-(cyclopropylmethoxy) phenyl]methyl }-5-isopropyl-3,6-dimethoxy-2,5-dihydropyrazine (6 g, 15.8 mmol, 87.27%) as a light-yellow oil. The product was used without further purification.

LCMS: MS (ESI) mass calcd. for $C_{20}H_{28}ClN_2O_3$: 379.18 m/z; found 379.15 $[M+H]^+$.

^{1}H NMR: (300 MHz, DMSO-d6): δ 0.25 – 0.39 (m, 2H), 0.56 (s, 2H), 0.49 – 0.71 (m, 9H), 0.98 (dd, J = 6.9, 2.4 Hz, 7H), 1.13 – 1.29 (m, 1H), 2.17 (dtq, J = 10.3, 6.9, 3.4 Hz, 2H), 2.71 (dd, J = 13.1, 8.1 Hz, 1H), 3.03 – 3.21 (m, 1H), 3.45 – 3.70 (m, 11H), 3.79 (dd, J = 9.4, 5.8 Hz, 1H), 3.79 – 3.94 (m, 1H), 3.89 – 4.00 (m, 4H), 4.27 (ddd, J = 8.2, 4.8, 3.5 Hz, 1H), 6.90 (d, J = 8.8 Hz, 1H), 7.05 – 7.22 (m, 2H).

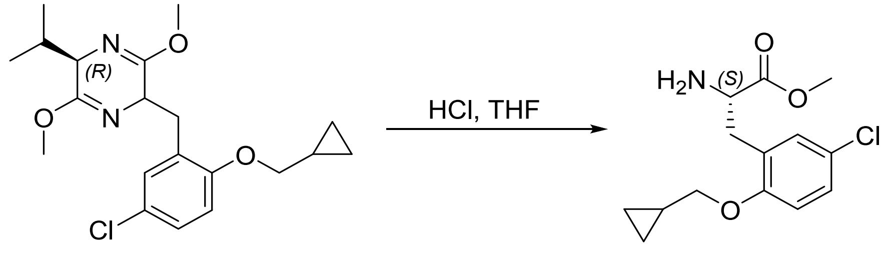

Methyl (S)-2-amino-3-(5-chloro-2-(cyclopropylmethoxy)phenyl)propanoate: A solution of (5R)-2-{[5-chloro-2-(cyclopropylmethoxy) phenyl] methyl}-5-isopropyl-3,6-dimethoxy-2,5-

dihydropyrazine (6 g, 15.8 mmol, 1 equiv) was dissolved in 4N HCl in dioxane (50 mL). The reaction was stirred for 1 hr at room temperature.. The mixture was neutralized to pH 7 with 1 N NaOH. The resulting mixture was extracted with EtOAc (3 x 200mL). The combined organic layers were washed with brine (100 mL), dried over anhydrous Na_2SO_4. After filtration, the filtrate was concentrated under reduced pressure. The residue was purified by RP chromatography (C18, 0-100% MeCN+0.1% TFA in water+0.1%TFA). Pure fractions were evaporated to dryness to afford methyl (2S)-2-amino-3-[5-chloro-2-(cyclopropylmethoxy)phenyl] propanoate as the TFA salt (4.2 g, 11.9 mmol, 75.5%) as a white solid.

LCMS: MS (ESI) mass calcd. for $C_{14}H_{19}ClNO_3$: 284.11 m/z; found 284.20 $[M+H]^+$.

^{1}H NMR: (300 MHz, DMSO-d_6): δ 0.28 – 0.40 (m, 2H), 0.56 (ddt, J = 7.3, 3.9, 1.4 Hz, 2H), 1.15 – 1.29 (m, 1H), 2.51 (q, J = 1.9 Hz, 2H), 3.00 (dd, J = 13.8, 7.1 Hz, 1H), 3.17 (dd, J = 13.8, 7.5 Hz, 1H), 3.66 (s, 1H), 3.86 (dt, J = 6.9, 1.6 Hz, 2H), 4.23 (t, J = 7.2 Hz, 1H), 7.00 (dd, J = 8.8, 1.3 Hz, 1H), 7.23 (dd, J = 2.7, 1.3 Hz, 1H), 7.29 (ddd, J = 8.7, 2.8, 1.3 Hz, 1H), 8.50 (s, 3H).

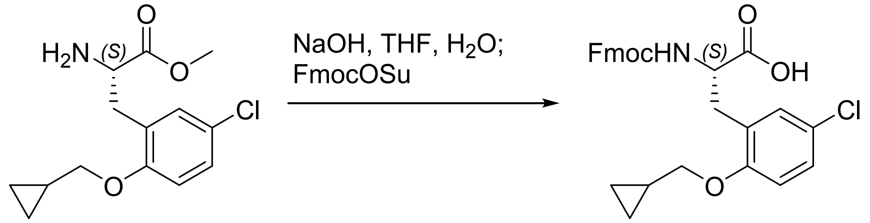

5Cl2cPrF: To a solution of methyl (2S)-2-amino-3-[5-chloro-2-(cyclopropylmethoxy) phenyl] propanoate (4.2 g, 11.9 mmol, 1.0 equiv) in THF (60 mL) a solution of NaOH (1.57g, 39.27 mmol, 3.3 equiv) in water (30mL) was added. The reaction was stirred for 2 hr. After the saponification was complete, 2N HCl was added to adjust the pH to ~8. FmocOSu (4.4 g, 13.09 mmol, 1.1 equiv). After the protection was compete the mixture was acidifed to pH ~3 and extracted with EtOAc (3 x 200mL). The combined organic layers were washed with brine (100 mL), dried over anhydrous Na_2SO_4. After filtration, the filtrate was concentrated under reduced pressure. The residue was purified by RP chromatography (C18, 0-100% MeCN+0.1% TFA in water+0.1%TFA). Pure fractions were evaporated to afford (2S)-3-[5-chloro-2-(cyclopropylmethoxy) phenyl]-2-{[(9H-fluoren-9-ylmethoxy) carbonyl] amino} propanoic acid (4.09 g, 8.3 mmol 69.8%) as a white solid.

LCMS: MS (ESI) mass calcd. for $C_{28}H_{27}ClNO_5$: 492.16 m/z; found 492.10 $[M+H]^+$.

^{1}H NMR: (300 MHz, DMSO-*d_6*): δ 0.35 (q, J = 5.3, 4.9 Hz, 2H), 0.54 (h, J = 3.9 Hz, 2H), 1.24 (s, 1H), 2.63 – 2.80 (m, 1H), 3.20 (dd, J = 13.6, 4.7 Hz, 1H), 3.77 – 3.95 (m, 2H), 4.18 (s, 3H), 4.33 (s, 1H), 6.95 (d, J = 8.7 Hz, 1H), 7.17 – 7.50 (m, 7H), 7.63 (t, J = 7.1 Hz, 3H), 7.88 (d, J = 7.6 Hz, 2H), 12.65 (s, 1H).

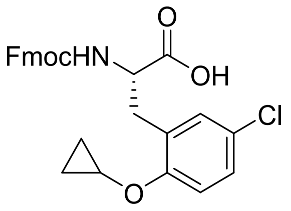

(S)-2-((((9H-fluoren-9-yl)methoxy)carbonyl)amino)-3-(5-chloro-2-cyclopropoxyphenyl)propanoic acid (Phe0034) was synthesized as previously reported.[2]

LCMS: MS (ESI) mass calcd. for $C_{27}H_{24}ClNO_5Na$: 500.12 m/z; found 500.10 $[M+Na]^+$.

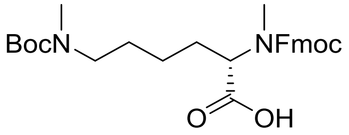

N2-(((9H-fluoren-9-yl)methoxy)carbonyl)-N6-(tert-butoxycarbonyl)-N2,N6-dimethyl-L-lysine (NMeKMe) was synthesized as previously reported.[1]

LCMS: MS (ESI) mass calcd. for $C_{28}H_{36}N_2NaO_6$: 519.25 *m/z*; found: 519.0 $[M+Na]^+$.

^{1}H NMR (400 MHz, DMSO) δ 7.89 (t, *J* = 7.3 Hz, 2H), 7.64 (ddd, *J* = 11.8, 7.4, 3.7 Hz, 2H), 7.44 – 7.27 (m, 4H), 4.40 – 4.22 (m, 3H), 3.14 (t, *J* = 7.2 Hz, 2H), 2.73 (si, 6H), 1.96 – 0.91 (m, 16H).

^{13}C NMR (101 MHz, DMSO) δ 173.09, 156.56, 156.12, 155.26, 144.27, 141.25, 128.13, 127.58, 125.45, 120.59, 78.69, 67.27, 58.36, 47.17, 33.93, 30.59, 28.49, 27.98, 27.13, 26.77, 23.25.

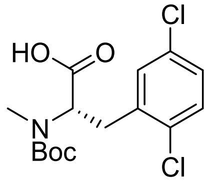

(S)-2-((tert-butoxycarbonyl)(methyl)amino)-3-(2,5-dichlorophenyl)propanoic acid (SI-1) was synthesized as previously reported.[3]

LCMS: MS (ESI) mass calcd. For $C_{15}H_{19}Cl_2NO_4Na$: 370.06 m/z; Found 370.0 $[M+H]^+$

^{1}H NMR: δ ppm 7.34 - 7.41 (m, 1 H) 7.20 - 7.32 (m, 2 H) 4.82 (dd, J=4.00, 4.00 Hz, 1 H) 3.39 - 3.48 (m, 1 H) 3.11 - 3.23 (m, 1 H) 2.69 - 2.81 (m, 3 H) 1.28 - 1.39 (m, 9 H)

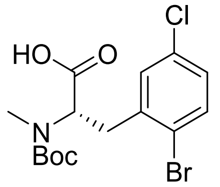

(S)-3-(2-bromo-5-chlorophenyl)-2-((tert-butoxycarbonyl)(methyl)amino)propanoic acid (SI-2) was synthesized as previously reported.[3]

LCMS: MS (ESI) mass calcd. For $C_{15}H_{20}BrClNO_4$: 392.03 m/z; Found 292.9 $[M-Boc]^+$

^{1}H NMR: (400 MHz, DMSO-d_6): 1.23 (d, 9H), 2.66 (s, 3H), 3.05 – 3.32 (m, 2H), 4.62 – 4.93 (m, 1H), 7.16 – 7.74 (m, 3H), 13.06 (s, 1H).

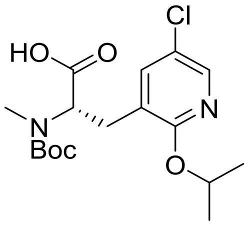

(S)-2-((tert-butoxycarbonyl)(methyl)amino)-3-(5-chloro-2-isopropoxypyridin-3-yl)propanoic acid (SI-3) was synthesized as previously reported.[3]

LCMS: MS (ESI) mass calcd. For $C_{17}H_{26}ClN_2O_5$: 373.15 m/z; Found 373.40 [M+H]+

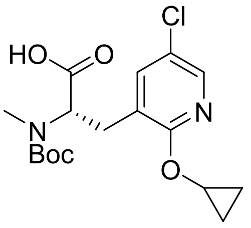

(S)-2-((tert-butoxycarbonyl)(methyl)amino)-3-(5-chloro-2-cyclopropoxypyridin-3-yl)propanoic acid (SI-4) was synthesized as previously reported.[3]

LCMS: MS (ESI) mass calcd. for $C_{17}H_{24}ClN_2O_5$: 371.13 m/z; Found 371.00. $[M+H]^+$

^{1}H NMR: (400 MHz, DMSO-DF_6) δ 0.79 – 0.61 (m, 4H), 1.21 (s, 9H), 2.95 – 2.84 (m, 2H), 3.00 (s, 3H), 3.09 – 3.01 (m, 1H), 4.86 (dd, J = 11.3, 4.6 Hz, 1H), 7.67 (d, J = 2.6 Hz, 1H), 8.11 (dd, J = 18.4, 2.6 Hz, 1H).

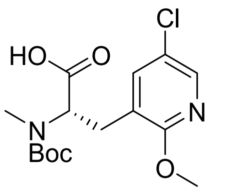

(S)-2-((tert-butoxycarbonyl)(methyl)amino)-3-(5-chloro-2-methoxypyridin-3-yl)propanoic acid (SI-5) was synthesized as previously reported.[3]

LCMS: MS (ESI) mass calcd. For $C_{15}H_{22}ClN_2O_5$: 345.12 m/z; Found 345.29 [M+H]+

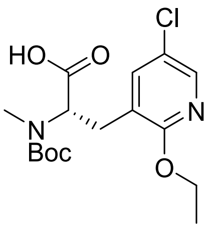

(S)-2-((tert-butoxycarbonyl)(methyl)amino)-3-(5-chloro-2-ethoxypyridin-3-yl)propanoic acid (SI-6) was synthesized as previously reported.[3]

LCMS: MS (ESI) mass calcd. For $C_{16}H_{23}ClN_2O_5$: 359.14 m/z; Found 359.31 [M+H]+

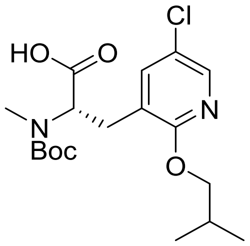

(S)-2-((tert-butoxycarbonyl)(methyl)amino)-3-(5-chloro-2-isobutoxypyridin-3-yl)propanoic acid (SI-7) was synthesized as previously reported.[3]

LCMS: MS (ESI) mass calcd. For $C_{18}H_{28}ClN_2O_5$: 387.17 m/z; Found 387.34 [M+H]+

(2S,4R)-1-(3,3-difluoro-1-(trifluoromethyl)cyclobutane-1-carbonyl)-4-fluoropyrrolidine-2-carboxylic acid (SI-8):

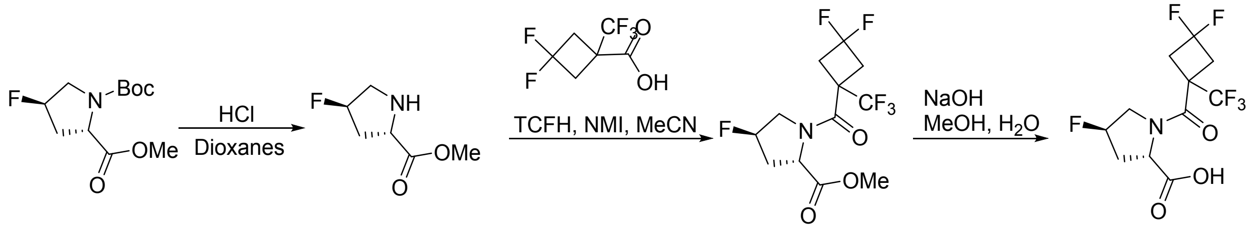

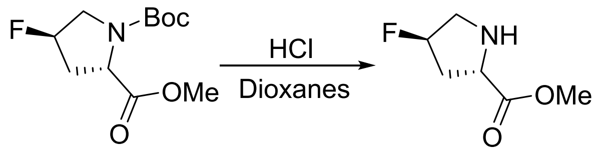

methyl (2S,4R)-4-fluoropyrrolidine-2-carboxylate: A solution of 1-tert-butyl 2-methyl (2S,4R)-4-fluoropyrrolidine-1,2-dicarboxylate (150 g, 606 mmol, 1 equiv) and 4N HCl 1,4-dioxane (758. mL) was stirred for 30 min at room temperature and then concentrated under vacuum to afford the desired product as the HCl salt (110 g, 599 mmol, 98%). The crude product was used in the next step directly without further purification.

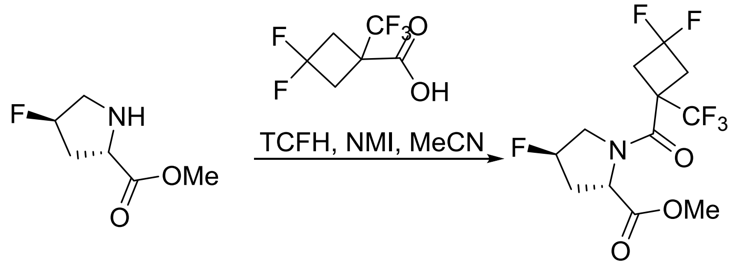

methyl (2S,4R)-1-(3,3-difluoro-1-(trifluoromethyl)cyclobutane-1-carbonyl)-4-fluoropyrrolidine-2-carboxylate: To a stirred solution of methyl (2S,4R)-4-fluoropyrrolidine-2-carboxylate (110 g, 599 mmol, 1 equiv), 3,3-difluoro-1-(trifluoromethyl)cyclobutane-1-carboxylic acid (122.06 g, 598 mmol, 1 equiv) and TCFH (251.69 g, 897 mmol, 1.5 equiv) in MeCN(1100 mL) was added NMI (245.51 g, 2.99 mol, 5 equiv) dropwise over 45 min at 0°C. The mixture was slowly warmed up to room temperature and stirred overnight at room temperature. The mixture was concentrated under vacuum at 28°C and diluted with EtOAc (1L) . The organic layer was and washed with 1N HCl (500 mL). The aqueous layer was extracted again with EtOAc (500mL)

and the combined organic layers were washed with saturated $NaHCO_3$ (500mL) and brine (500mL), dried over anhydrous Na_2SO_4 and filtered. After filtration, the filtrate was concentrated under vacuum to afford the desired product (180 g, 540 mmol, 90%) as a light yellow solid. The crude product was used in the next step directly without further purification.

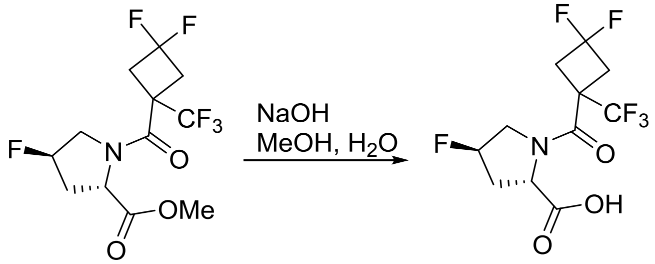

SI-8: To a stirred solution of methyl (2S,4R)-1-[3,3-difluoro-1-(trifluoromethyl)cyclobutanecarbonyl]-4-fluoropyrrolidine-2-carboxylate (180 g, 540 mmol, 1 equiv) in MeOH (1400 mL) was added dropwise NaOH (58.33 g, 1.45 mol, 2.7 equiv) in H_2O (400 mL) over 30 min at 0-20°C. The mixture was stirred for 2 hr at room temperature. MeOH was evaporated under vacuum. The residue was diluted with water and acidified with 3N HCl at 0-20 °C. Then the precipitated solids were collected by filtration and washed with water. The resulting filtrate was reacidified with 3N HCl and the resulting precipitate was filtered, washed with water and collected. The solids were combined and dried in an oven for 48hr at 40°C. **SI-8** was isolated as a white solid and used with no further purification (150.65 g, 472 mmol, 87.4%) as a white solid.

LCMS: MS (ESI) mass calcd. For $C_{11}H_{12}F_6NO_3$: 320.07 m/z; Found 319.95 $[M+H]^+$

^{1}H NMR (400 MHz, DMSO-d_6): δ 2.11 (dddd, *J* = 40.2, 14.7, 8.8, 4.0 Hz, 1H), 2.53 – 2.65 (m, 1H), 3.10 (q, *J* = 14.1 Hz, 1H), 3.31 (tt, *J* = 10.5, 2.7 Hz, 3H), 3.58 – 3.88 (m, 2H), 4.49 (t, *J* = 8.7 Hz, 1H), 5.39 (dt, *J* = 52.5, 3.2 Hz, 1H), 12.88 (s, 1H).

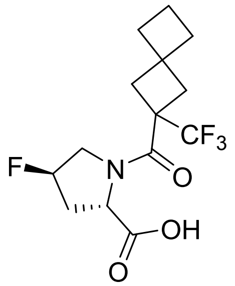

(2S,4R)-4-fluoro-1-(2-(trifluoromethyl)spiro[3.3]heptane-2-carbonyl)pyrrolidine-2-carboxylic acid (SI-9) was synthesized as previously reported.[1]

LCMS: MS (ESI): mass calcd. For $C_{14}H_{18}F_4NO_3$ 324.29 m/z found 324.41 [M+H]+.

^{1}H NMR (400 MHz, DMSO) δ 5.34 (dt, J = 52.5, 3.2 Hz, 1H), 4.41 (t, J = 8.7 Hz, 1H), 3.85 – 3.68 (m, 2H), 3.57 (ddd, J = 37.6, 12.8, 2.6 Hz, 1H), 2.71 – 2.54 (m, 1H), 2.47 – 2.43 (m, 1H), 2.29 – 2.08 (m, 1H), 2.04 (t, J = 7.6 Hz, 2H), 2.00 – 1.84 (m, 2H), 1.74 (q, J = 7.6 Hz, 2H).

^{13}C NMR (101 MHz, DMSO) δ 172.84, 167.06, 167.04, 131.47, 128.69, 125.90, 123.11, 94.04, 92.28, 58.69, 54.01, 53.80, 47.05, 46.78, 39.00, 36.78, 35.25, 34.96, 34.91, 34.69, 15.61.

Method A – Synthesis of Macrocyclic Core

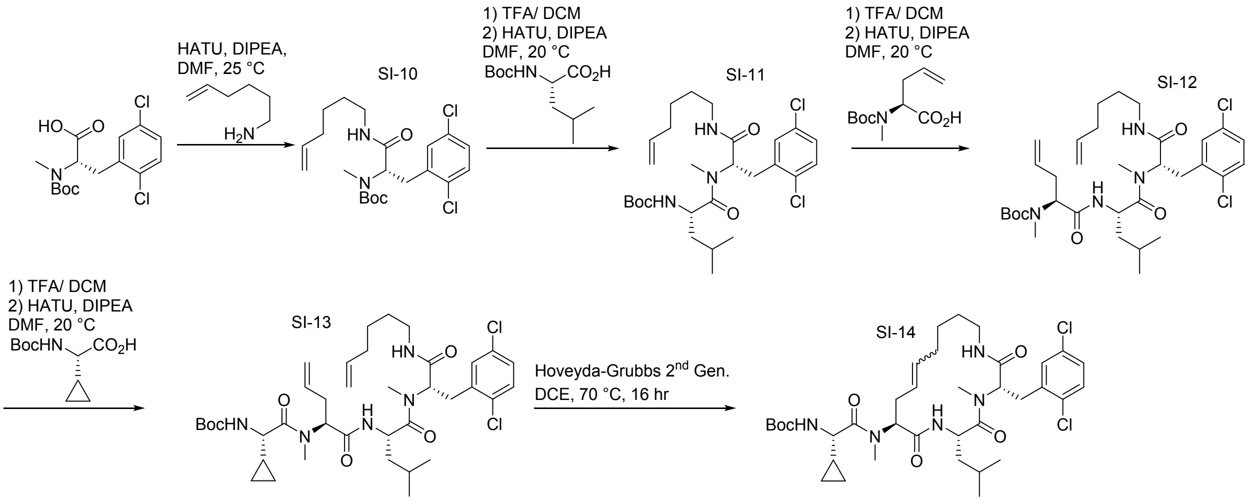

Representative procedure for Method A:

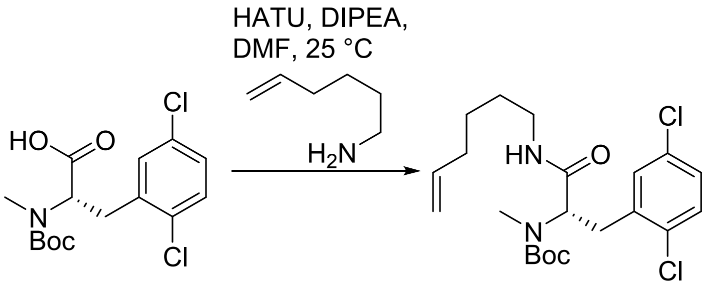

Tert-butyl (S)-(3-(2,5-dichlorophenyl)-1-(hex-5-en-1-ylamino)-1-oxopropan-2-yl)(methyl)carbamate (SI-10): To a 500mL round-bottom flask were added (S)-2-((tert-butoxycarbonyl)(methyl)amino)-3-(2,5-dichlorophenyl)propanoic acid (2.5 g, 7.2 mmol, 1 eq.)

and HATU (3.3 g, 8.6 mmol, 1.2 eq.) The solids were dissolved in DMF (20 mL), and to the solution was added hex-5-en-1-amine (0.85 g, 1.0 mL, 8.6 mmol, 1.2 eq.) followed by DIPEA (3.2 g, 4.4 mL, 25 mmol, 3.5 eq.). The solution was confirmed basic by pH paper and stirred for 2 hours. The reaction mixture was diluted in water (100 mL) and extracted with ethyl acetate (100 mL x 3). The combined organics were washed with brine (150 mL), dried over anhydrous magnesium sulfate, and filtered over celite. The organic extract was concentrated under rotary evaporation to give a yellow-orange oil. The crude material was purified by silica gel chromatography (0-100% ethyl acetate in hexanes) and the eluent was concentrated to give tert-butyl (S)-(3-(2,5-dichlorophenyl)-1-(hex-5-en-1-ylamino)-1-oxopropan-2-yl)(methyl)carbamate (2.2 g, 5.1 mmol, 71% yield) as a colorless oil.

HRMS: MS (ESI) mass calcd. for : $C_{21}H_{30}Cl_2N_2O_3$: 438.16 m/z; Found 373.1096 $[M\text{-}tBu]^+$.

^{1}H NMR: (400 MHz, D_3COD) δ = 7.49 - 7.16 (m, 3H), 5.85 - 5.72 (m, 1H), 5.04 - 4.93 (m, 4H), 3.50 - 3.35 (m, 1H), 3.27 - 3.14 (m, 2H), 3.11 - 2.98 (m, 1H), 2.77 (s, 3H), 2.08 (br d, J = 6.8 Hz, 2H), 1.52 (br s, 2H), 1.44 - 1.40 (m, 1H), 1.39 - 1.22 (m, 9H)

^{13}C NMR: (400 MHz, D_3COD) δ = 170.494, 170.487, 170.465, 170.313, 170.302, 155.978, 155.483, 138.302, 137.94, 137.709, 137.612, 137.601, 132.552, 132.45, 132.227, 131.15, 130.919, 130.887, 130.482, 130.471, 128.04, 127.972, 127.838, 113.745, 80.585, 80.516, 80.252, 59.167, 57.975, 48.461, 48.378, 48.324, 48.252, 48.169, 48.111, 48.039, 47.956, 47.898, 47.825, 47.746, 47.681, 47.612, 47.533, 47.399, 47.186, 46.973, 39.154, 39.146, 39.114, 39.013, 38.984, 38.966, 33.086, 32.013, 31.854, 31.814, 31.738, 30.861, 30.807, 30.46, 30.315, 28.549, 26.971, 26.797, 25.941.

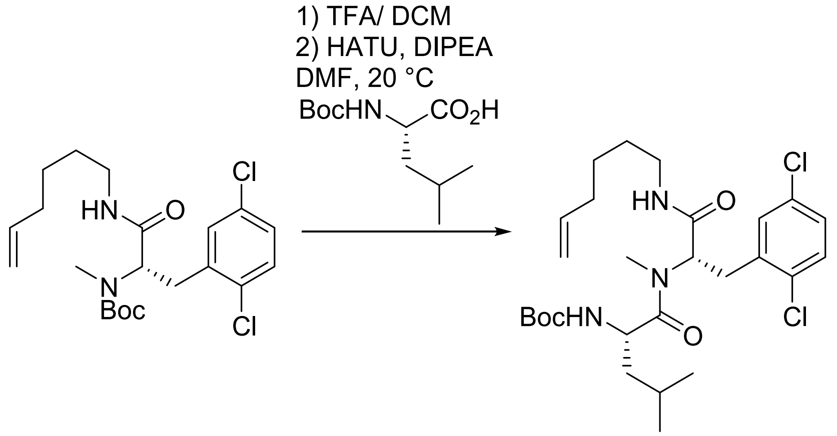

Tert-butyl ((S)-1-(((S)-3-(2,5-dichlorophenyl)-1-(hex-5-en-1-ylamino)-1-oxopropan-2-yl)(methyl)amino)-4-methyl-1-oxopentan-2-yl)carbamate (SI-11): tert-butyl (S)-(3-(2,5-dichlorophenyl)-1-(hex-5-en-1-ylamino)-1-oxopropan-2-yl)(methyl)carbamate (2.2 g, 5.1 mmol, 1 eq.) was dissolved in DCM (15 mL) and TFA (15 mL) and allowed to sit at room temperature for 30 minutes until the Boc group was removed as confirmed by LCMS. The reaction mixture was concentrated by rotary evaporation. The residue was resuspended in toluene and concentrated to remove residual TFA, and this procedure was repeated two times. The crude residue was used in the next reaction without further purification.

To a 500mL round-bottom flask containing the residue was added a solution of Boc-L-Leucine monohydrate (1.5 g, 6.1 mmol, 1.2 eq.), HATU (2.3 g, 6.1 mmol, 1.2 eq.), and DIPEA (2.3 g, 3.1 mL, 18 mmol, 3.5 eq.). in DMF (20 mL). The reaction mixture was checked by pH paper and DIPEA was added in 1 eq. portions until the reaction was confirmed to be basic (pH ~9). The reaction was stirred for 2 hours at room temperature. The reaction mixture was diluted in water (100 mL) and extracted with ethyl acetate (100 mL x 3). The combined organics were washed with brine (150 mL), dried over anhydrous magnesium sulfate, and filtered over celite. The organic extract was concentrated under rotary evaporation to give a yellow-orange oil. The crude material was purified by silica gel chromatography (0-100% ethyl acetate in hexanes) and the eluent was concentrated to give tert-butyl ((S)-1-(((S)-3-(2,5-dichlorophenyl)-1-(hex-5-en-1-ylamino)-1-oxopropan-2-yl)(methyl)amino)-4-methyl-1-oxopentan-2-yl)carbamate (2.4 g, 4.4 mmol, 86% yield) as a colorless oil.

HRMS: MS (ESI) mass calcd. for $C_{27}H_{41}Cl_2N_3O_4$: 541.25 m/z; Found 542.2565 $[M+H]^+$.

^{1}H NMR: (400 MHz, D_3COD) δ= 7.48 - 7.35 (m, 1H), 7.31 (qd, J = 2.4, 4.5 Hz, 1H), 7.27 - 7.20 (m, 1H), 5.79 (tdd, J = 6.8, 10.3, 17.0 Hz, 1H), 5.03 - 4.91 (m, 4H), 4.58 (s, 2H), 4.50 - 4.18 (m, 1H), 3.55 - 3.36 (m, 1H), 3.28 - 3.20 (m, 1H), 3.20 - 3.06 (m, 2H), 3.01 (s, 1H), 2.90 (s, 1H), 2.13 - 1.98 (m, 2H), 1.73 - 1.62 (m, 1H), 1.61 - 1.52 (m, 2H), 1.51 - 1.45 (m, 2H), 1.42 (d, J = 1.2 Hz, 9H), 1.36 - 1.30 (m, 1H), 1.28 - 1.10 (m, 1H), 0.92 (dd, J = 6.8, 10.4 Hz, 3H), 0.71 (dd, J = 4.8, 6.4 Hz, 2H)

^{13}C NMR: (400 MHz, D_3COD) δ = 175.037, 174.214, 169.8, 168.872, 157.256, 156.682, 138.298, 138.172, 137.63, 137.225, 132.884, 132.44, 132.414, 132.306, 131.631, 131.295, 131.11, 130.489, 128.709, 128.163, 113.846, 113.759, 79.602, 79.191, 60.192, 58.986, 49.646, 48.324, 48.252,

48.111, 48.039, 47.959, 47.898, 47.825, 47.746, 47.681, 47.612, 47.399, 47.186, 46.977, 40.165, 39.334, 38.958, 38.352, 33.357, 33.075, 33.031, 31.868, 31.529, 29.174, 28.426, 28.376, 27.365, 26.017, 25.84, 24.493, 23.803, 22.369, 22.117, 20.303, 19.328.

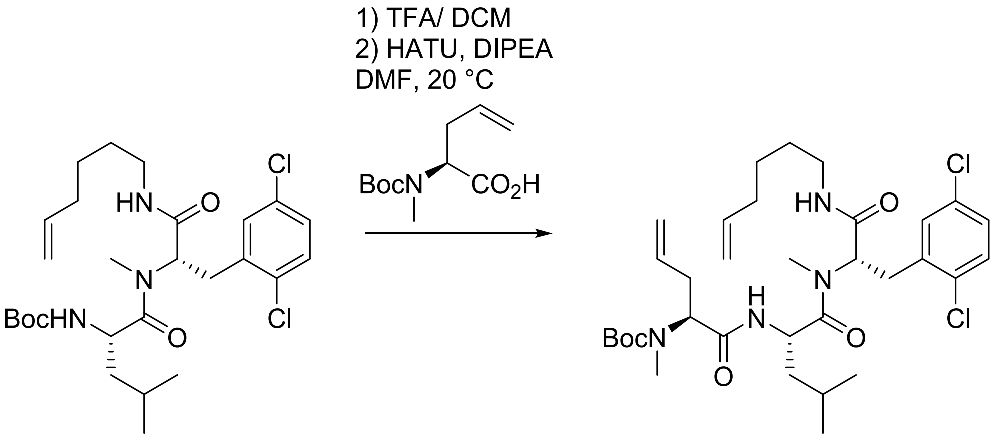

Tert-butyl ((S)-1-(((S)-1-(((S)-3-(2,5-dichlorophenyl)-1-(hex-5-en-1-ylamino)-1-oxopropan-2-yl)(methyl)amino)-4-methyl-1-oxopentan-2-yl)amino)-1-oxopent-4-en-2-yl)(methyl)carbamate (SI-12): tert-butyl ((S)-1-(((S)-3-(2,5-dichlorophenyl)-1-(hex-5-en-1-ylamino)-1-oxopropan-2-yl)(methyl)amino)-4-methyl-1-oxopentan-2-yl)carbamate (2.4 g, 4.4 mmol, 1 eq.) was dissolved in DCM (15 mL) and TFA (15 mL) and allowed to sit at room temperature for 30 minutes until the Boc group was removed as confirmed by LCMS. The reaction mixture was concentrated by rotary evaporation. The residue was resuspended in toluene and concentrated to remove residual TFA, and this procedure was repeated two times. The crude residue was used in the next reaction without further purification.

To a 500mL round-bottom flask containing the residue was added a solution of (S)-2-((tert-butoxycarbonyl)(methyl)amino)pent-4-enoic acid (1.2 g, 5.3 mmol, 1.2 eq.), HATU (2.0 g, 5.3 mmol, 1.2 eq.), and DIPEA (2.0 g, 2.7 mL, 15 mmol, 3.5 eq.). in DMF (20 mL). The reaction mixture was checked by pH paper and DIPEA was added in 1 eq. portions until the reaction was confirmed to be basic (pH ~9). The reaction was stirred for 2 hours at room temperature. The reaction mixture was diluted in water (100 mL) and extracted with ethyl acetate (100 mL x 3). The combined organics were washed with brine (150 mL), dried over anhydrous magnesium sulfate, and filtered over celite. The organic extract was concentrated under rotary evaporation to give a yellow-orange oil. The crude material was purified by silica gel chromatography (0-100% ethyl acetate in hexanes) and the eluent was concentrated to give tert-butyl ((S)-1-(((S)-1-(((S)-3-(2,5-

dichlorophenyl)-1-(hex-5-en-1-ylamino)-1-oxopropan-2-yl)(methyl)amino)-4-methyl-1-oxopentan-2-yl)amino)-1-oxopent-4-en-2-yl)(methyl)carbamate (2.72 g, 4.16 mmol, 94% yield) as a pale yellow oil.

HRMS: MS (ESI) mass calcd. for C $C_{33}H_{50}Cl_2N_4O_5$: 652.32 m/z; Found 653.3250 $[M+H]^+$.

^{1}H NMR: (400 MHz, D_3COD) δ=8.51 - 8.06 (m, 1H), 7.94 - 7.62 (m, 1H), 7.48 - 7.35 (m, 1H), 7.34 - 7.30 (m, 1H), 7.26 - 7.22 (m, 1H), 5.90 - 5.66 (m, 2H), 5.18 - 5.04 (m, 2H), 4.99 - 4.91 (m, 2H), 4.72 (ddd, J = 3.2, 7.5, 10.8 Hz, 1H), 4.67 - 4.42 (m, 2H), 3.51 - 3.36 (m, 1H), 3.28 - 3.22 (m, 1H), 3.21 - 3.09 (m, 2H), 3.03 (s, 1H), 2.90 (s, 1H), 2.82 (s, 2H), 2.58 (td, J = 5.2, 9.7 Hz, 1H), 2.49 - 2.39 (m, 1H), 2.17 - 2.00 (m, 2H), 1.69 - 1.52 (m, 3H), 1.45 (d, J = 8.4 Hz, 10H), 1.40 - 1.33 (m, 1H), 1.26 - 1.16 (m, 1H), 0.92 (br dd, J = 6.4, 13.3 Hz, 4H), 0.79 - 0.68 (m, 2H)

^{13}C NMR: (400 MHz, D_3COD) δ = 173.369, 169.743, 168.771, 138.298, 138.244, 137.605, 137.207, 134.206, 134.058, 132.92, 132.469, 132.432, 132.27, 131.681, 131.284, 131.143, 130.511, 128.748, 128.178, 116.732, 113.864, 113.799, 60.283, 58.888, 48.335, 48.263, 48.122, 48.049, 47.963, 47.909, 47.836, 47.692, 47.623, 47.41, 47.197, 46.984, 39.865, 39.32, 39.005, 33.418, 33.089, 33.053, 31.883, 31.626, 29.16, 28.43, 28.206, 27.285, 27.26, 25.978, 25.869, 24.518, 23.901, 22.413, 22.138, 20.249, 19.321.

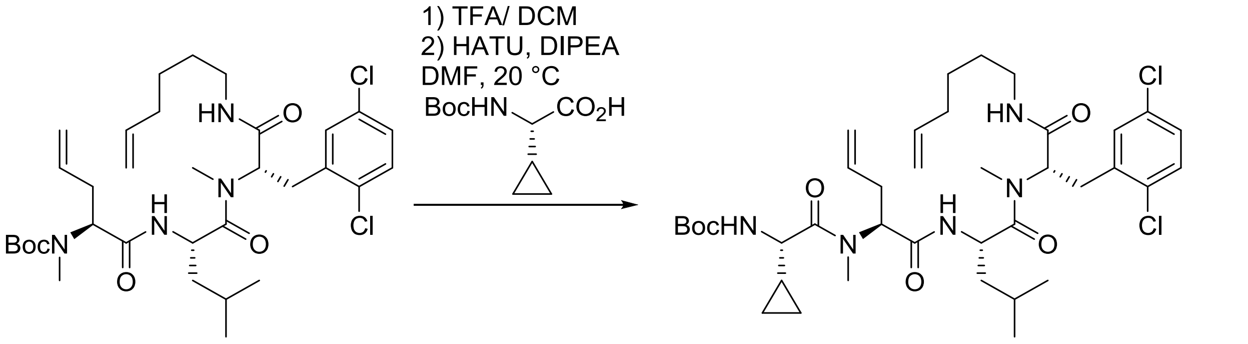

Tert-butyl ((1S,4S,7S,10S)-4-allyl-1-cyclopropyl-10-(2,5-dichlorobenzyl)-7-isobutyl-3,9-dimethyl-2,5,8,11-tetraoxo-3,6,9,12-tetraazaoctadec-17-en-1-yl)carbamate (SI-13) : tert-butyl ((S)-1-(((S)-1-(((S)-3-(2,5-dichlorophenyl)-1-(hex-5-en-1-ylamino)-1-oxopropan-2-yl)(methyl)amino)-4-methyl-1-oxopentan-2-yl)amino)-1-oxopent-4-en-2-yl)(methyl)carbamate (2.7 g, 4.1 mmol, 1 eq.) was dissolved in DCM (15 mL) and TFA (15 mL) and allowed to sit at room temperature for 30 minutes until the Boc group was removed as confirmed by LCMS. The reaction mixture was concentrated by rotary evaporation. The residue was resuspended in toluene

and concentrated to remove residual TFA, and this procedure was repeated two times. The crude residue was used in the next reaction without further purification.

To a 500mL round-bottom flask containing the residue was added a solution of (S)-2-((tert-butoxycarbonyl)amino)-2-cyclopropylacetic acid (1.1 g, 5.0 mmol, 1.2 eq.), HATU (1.9 g, 5.0 mmol, 1.2 eq.), and DIPEA (1.9 g, 2.5 mL, 14 mmol, 3.5 eq.). in DMF (20 mL). The reaction mixture was checked by pH paper and DIPEA was added in 1 eq. portions until the reaction was confirmed to be basic (pH ~9). The reaction was stirred for 2 hours at room temperature. The reaction mixture was diluted in water (100 mL) and extracted with ethyl acetate (100 mL x 3). The combined organics were washed with brine (150 mL), dried over anhydrous magnesium sulfate, and filtered over celite. The organic extract was concentrated under rotary evaporation to give a yellow-orange oil. The crude material was purified by silica gel chromatography (0-100% ethyl acetate in hexanes) and the eluent was concentrated to give tert-butyl ((1S,4S,7S,10S)-4-allyl-1-cyclopropyl-10-(2,5-dichlorobenzyl)-7-isobutyl-3,9-dimethyl-2,5,8,11-tetraoxo-3,6,9,12-tetraazaoctadec-17-en-1-yl)carbamate (3.02 g, 4.02 mmol, 97 % yield) as a pale yellow oil.

HRMS: MS (ESI) mass calcd. for$C_{38}H_57Cl_2N_5O_6$: 749.37 m/z; Found 750.3776 $[M+H]^+$.

^{1}H NMR: (400 MHz, D_3COD) δ = 8.74 - 8.25 (m, 1H), 8.01 - 7.49 (m, 1H), 7.47 - 7.36 (m, 1H), 7.35 - 7.28 (m, 1H), 7.28 - 7.17 (m, 1H), 5.89 - 5.64 (m, 2H), 5.24 - 5.04 (m, 2H), 5.03 - 4.90 (m, 5H), 4.75 - 4.62 (m, 2H), 4.61 - 4.34 (m, 1H), 4.29 - 3.96 (m, 1H), 3.53 - 3.35 (m, 1H), 3.29 - 3.21 (m, 1H), 3.18 - 3.11 (m, 1H), 3.11 - 3.01 (m, 3H), 2.90 (s, 1H), 2.76 (d, J = 13.6 Hz, 1H), 2.65 - 2.36 (m, 2H), 2.12 - 1.75 (m, 2H), 1.65 - 1.47 (m, 3H), 1.46 - 1.42 (m, 9H), 1.42 - 1.39 (m, 2H), 1.38 - 1.04 (m, 3H), 0.95 - 0.85 (m, 3H), 0.74 - 0.67 (m, 2H), 0.66 - 0.56 (m, 1H), 0.56 - 0.30 (m, 4H)

^{13}C NMR: (400 MHz, D_3COD) δ = 174.401, 174.143, 174.087, 173.893, 173.7, 173.26, 173.161, 172.994, 171.599, 171.515, 171.129, 170.954, 170.875, 170.518, 170.427, 169.851, 169.767, 169.68, 168.808, 168.774, 156.502, 156.187, 150.518, 138.326, 138.288, 138.269, 137.659, 137.613, 137.313, 137.215, 133.756, 133.688, 133.521, 132.933, 132.482, 132.444, 132.285, 131.697, 131.303, 131.17, 130.601, 130.548, 128.83, 128.777, 128.466, 128.224, 120.681, 117.473, 117.427, 116.896, 113.938, 113.9, 113.84, 79.255, 79.19, 79.08, 60.445, 60.316, 59.713, 59.05, 58.936, 58.872, 58.86, 57.396, 56.653, 56.183, 53.964, 53.233, 53.153, 49.562, 49.467, 48.454, 48.36, 48.291, 48.079, 47.863, 47.65, 47.438, 47.226, 47.104, 47.013, 39.869, 39.402,

39.368, 39.19, 39.156, 39.031, 38.45, 37.98, 37.313, 34.177, 33.475, 33.43, 33.122, 33.103, 32.296, 32.262, 31.901, 31.613, 31.552, 31.094, 31.01, 29.224, 28.803, 28.655, 28.454, 28.412, 28.306, 27.563, 27.434, 27.343, 26.008, 25.894, 24.579, 24.469, 23.816, 23.71, 22.481, 22.258, 22.22, 20.248, 19.944, 19.342, 19.015, 12.69, 12.531, 12.189, 12.121, 3.831, 3.74, 2.739, 2.694, 2.375, 2.326, 1.704, 1.617.

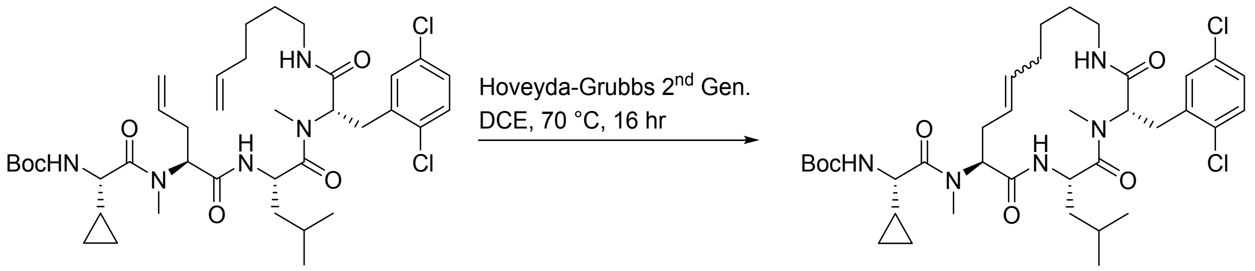

Tert-butyl ((S)-1-cyclopropyl-2-(((3S,6S,9S)-3-(2,5-dichlorobenzyl)-6-isobutyl-4-methyl-2,5,8-trioxo-1,4,7-triazacyclohexadec-11-en-9-yl)(methyl)amino)-2-oxoethyl)carbamate (SI-14): tert-butyl ((1S,4S,7S,10S)-4-allyl-1-cyclopropyl-10-(2,5-dichlorobenzyl)-7-isobutyl-3,9-dimethyl-2,5,8,11-tetraoxo-3,6,9,12-tetraazaoctadec-17-en-1-yl)carbamate (3.02 g, 4.02 mmol, 1 eq.) was dissolved in DCE (300 mL) to give a 13.4 mM solution. The solution was degassed with nitrogen for 20 minutes. To the solution was added Hoyveda-Grubbs II catalyst (M720 Umicore, 378 mgs, 0.15 eq.) and the solution was degassed for an additional 5 minutes. The reaction was heated to 70 ˚C and stirred under nitrogen overnight. Consumption of the starting material was confirmed by LCMS. The reaction was concentrated by rotary evaporation and purified by reverse-phase flash chromatography (50-100% acetonitrile in water, 0.1% TFA buffer) to give tert-butyl ((S)-1-cyclopropyl-2-(((3S,6S,9S)-3-(2,5-dichlorobenzyl)-6-isobutyl-4-methyl-2,5,8-trioxo-1,4,7-triazacyclohexadec-11-en-9-yl)(methyl)amino)-2-oxoethyl)carbamate (1.65 g, 2.3 mmol, 57.3 % yield) as a dark brown solid which was carried on without further purification. An aliquot was further purified by reverse-phase HPLC (50-100% acetonitrile in water, 0.05% formic acid buffer) for analytical purposes. Isolated as a mixture of isomers (~3:1). Analytical data given for major peak.

HRMS: MS (ESI) mass calcd. for $C_{36}H_{53}Cl_2N_5O_6$: 721.34 m/z; Found 722.3469 $[M+H]^+$.

^{1}H NMR: (400 MHz, D_3COD) δ =7.48 - 7.38 (m, 1H), 7.35 - 7.27 (m, 1H), 7.16 - 7.02 (m, 1H), 5.56 - 5.40 (m, 1H), 5.40 - 5.07 (m, 1H), 4.67 (br dd, J = 2.0, 12.4 Hz, 2H), 4.59 (br s, 9H), 4.22 -

4.12 (m, 1H), 3.67 - 3.49 (m, 1H), 3.49 - 3.46 (m, 1H), 3.09 (s, 1H), 2.79 (d, J = 7.2 Hz, 4H), 2.21 - 1.84 (m, 2H), 1.83 - 1.52 (m, 2H), 1.52 - 1.39 (m, 9H), 1.38 - 1.21 (m, 2H), 1.20 - 1.06 (m, 1H), 1.01 - 0.70 (m, 6H), 0.70 - 0.30 (m, 4H)

^{13}C NMR: (400 MHz, D_3COD) δ = 173.832, 172.782, 172.532, 171.887, 170.181, 169.703, 156.642, 137.996, 132.789, 132.478, 132.312, 131.856, 130.692, 130.605, 128.33, 128.311, 125.797, 124.943, 79.277, 79.228, 63.502, 59.482, 56.096, 54.294, 53.388, 48.568, 48.261, 48.049, 47.836, 47.624, 47.411, 47.195, 46.983, 39.74, 38.822, 38.735, 38.523, 38.007, 32.353, 31.716, 31.442, 31.37, 30.912, 30.237, 28.564, 28.306, 27.798, 27.696, 27.472, 27.298, 25.428, 25.018, 24.931, 24.499, 22.523, 22.341, 19.482, 12.603, 2.826, 2.682, 1.761, 1.7.

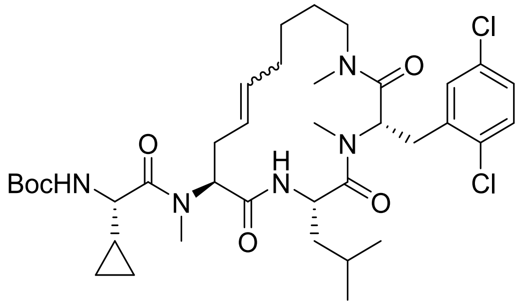

tert-butyl ((S)-1-cyclopropyl-2-(((3S,6S,9S)-3-(2,5-dichlorobenzyl)-6-isobutyl-1,4-dimethyl-2,5,8-trioxo-1,4,7-triazacyclohexadec-11-en-9-yl)(methyl)amino)-2-oxoethyl)carbamate (SI-15) SI-15 was synthesized via the procedure outlined in Method A using N-methylhex-5-en-1-amine as a starting material and was purified by reverse-phase flash chromatography (50-100% acetonitrile in water, 0.1% TFA buffer) to give tert-butyl ((S)-1-cyclopropyl-2-(((3S,6S,9S)-3-(2,5-dichlorobenzyl)-6-isobutyl-1,4-dimethyl-2,5,8-trioxo-1,4,7-triazacyclohexadec-11-en-9-yl)(methyl)amino)-2-oxoethyl)carbamate (2.31 g, 3.14 mmol) as a dark brown solid which was carried on without further purification. An aliquot was further purified by reverse-phase HPLC (50-100% acetonitrile in water, 0.05% formic acid buffer) for analytical purposes. Isolated as a mixture of isomers (~10:1). Analytical data given for major peak.

LCMS: MS (ESI) mass calcd. for $C_{37}H_{56}Cl_2N_5O_6$: 736.36 m/z; Found 736.4 $[M+H]^+$.

^{1}H NMR: (400 MHz, D_3COD) δ =7.40 (d, J = 8.4 Hz, 1H), 7.33 - 7.26 (m, 1H), 7.26 - 7.15 (m, 1H), 5.62 - 5.48 (m, 1H), 5.48 - 5.26 (m, 1H), 5.15 - 4.96 (m, 1H), 4.89 (br d, J = 1.6 Hz, 2H), 4.82 (br d, J = 3.6 Hz, 1H), 4.78 - 4.55 (m, 1H), 4.54 - 4.38 (m, 1H), 4.28 (dt, J = 5.6, 13.2 Hz, 1H),

4.22 - 4.09 (m, 1H), 3.53 - 3.32 (m, 2H), 3.29 - 3.14 (m, 1H), 3.13 - 3.01 (m, 2H), 2.98 - 2.86 (m, 3H), 2.82 - 2.73 (m, 3H), 2.64 - 2.47 (m, 1H), 2.16 - 1.76 (m, 3H), 1.73 - 1.54 (m, 2H), 1.53 - 1.41 (m, 9H), 1.41 - 1.21 (m, 3H), 1.18 - 0.97 (m, 2H), 0.97 - 0.87 (m, 3H), 0.79 (br d, J = 6.4 Hz, 2H), 0.69 - 0.24 (m, 4H)

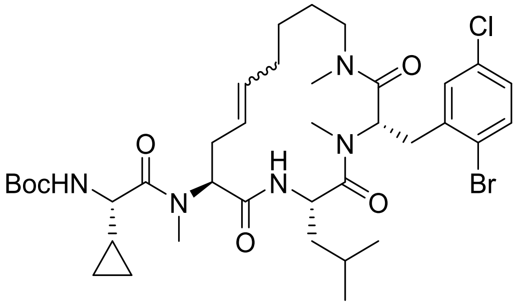

Tert-butyl ((S)-2-(((3S,6S,9S)-3-(2-bromo-5-chlorobenzyl)-6-isobutyl-1,4-dimethyl-2,5,8-trioxo-1,4,7-triazacyclohexadec-11-en-9-yl)(methyl)amino)-1-cyclopropyl-2-oxoethyl)carbamate (SI-16) was synthesized using the procedure outlined in Method A using N-methylhex-5-en-1-amine and **SI-2** as starting material. **SI-16** was purified column chromatography (SiO_2, 0 to 100% EtOAc in Hexane) and was isolated as a brown solid after concentration (10.35 g, 13.21 mmol). Isolated as a mixture of isomers (~10:1). Analytical data given for major peak.

LCMS: MS (ESI) mass calcd. For $C_{37}H_{56}BrClN_5O_6$ 780.30 m/z; Found 780.30 $[M+H]^+$.

^{1}H NMR (400 MHz, DMSO-d_6) δ 8.04-7.74 (m, 1H), 7.57 (br d, J = 8.2 Hz, 1H), 7.29 (br s, 1H), 7.22 (br d, J = 7.7 Hz, 1H), 6.62 - 6.20 (m, 1H), 5.56-5.43 (m, 1H), 5.40-5.20 (m, 1H), 4.93-4.93 (m, 1H), 4.91-4.88 (m, 1H), 5.01-4.55 (m, 2H), 4.35-4.09 (m, 2H), 3.36-3.11 (m, 2H), 3.06 (s, 3H), 3.00-2.79 (m, 8H), 2.78-2.58 (m, 2H), 2.09 (br s, 1H), 1.92 (br d, J = 10.0 Hz, 2H), 1.66-1.45 (m, 3H), 1.40 (br s, 9H), 1.29 (br s, 3H), 1.15-1.04 (m, 1H), 0.91-0.66 (m, 6H), 0.55-0.22 (m, 4H)

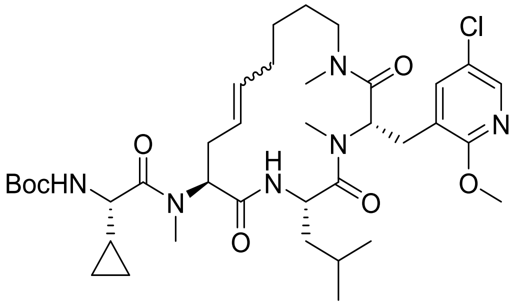

Tert-butyl ((S)-2-(((3S,6S,9S)-3-((5-chloro-2-methoxypyridin-3-yl)methyl)-6-isobutyl-1,4-dimethyl-2,5,8-trioxo-1,4,7-triazacyclohexadec-11-en-9-yl)(methyl)amino)-1-cyclopropyl-2-oxoethyl)carbamate (SI-17) was synthesized using the procedure outlined in Method A using N-methylhex-5-en-1-amine and **SI-5** as starting material. **SI-17** was purified by RP-HPLC (C18, 20% to 80% MeCN+0.1% TFA in water+0.1% TFA) and was isolated as a brown solid after lyophilization (2.5 g, 3.4 mmol, 68% yield). Isolated as a mixture of isomers.

LCMS: MS (ESI) mass calcd. for C37H57ClN6O7: 733.35 m/z; Found 733.77 [M+H]$^{+}$.

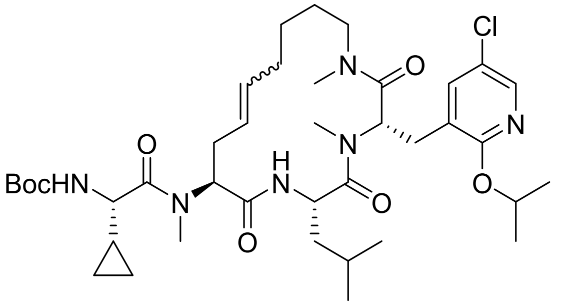

Tert-butyl ((S)-2-(((3S,6S,9S)-3-((5-chloro-2-isopropoxypyridin-3-yl)methyl)-6-isobutyl-1,4-dimethyl-2,5,8-trioxo-1,4,7-triazacyclohexadec-11-en-9-yl)(methyl)amino)-1-cyclopropyl-2-oxoethyl)carbamate (SI-18) was synthesized using the procedure outlined in Method A using N-methylhex-5-en-1-amine and **SI-3** as starting material. **SI-18** was purified by RP-HPLC (C18, 20% to 80% MeCN+0.1% TFA in water+0.1% TFA) and was isolated as a brown solid after lyophilization (0.74 g, 0.97 mmol, 40% yield). Isolated as a mixture of isomers.

LCMS: MS (ESI) mass calcd. for C39H61ClN6O7: 761.40 m/z; Found 761.98 [M+H]$^{+}$.

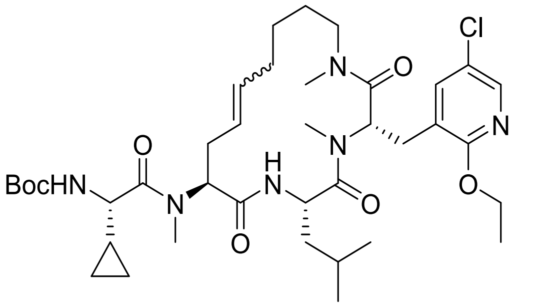

Tert-butyl ((S)-2-(((3S,6S,9S)-3-((5-chloro-2-ethoxypyridin-3-yl)methyl)-6-isobutyl-1,4-dimethyl-2,5,8-trioxo-1,4,7-triazacyclohexadec-11-en-9-yl)(methyl)amino)-1-cyclopropyl-2-

oxoethyl)carbamate (SI-19) was synthesized using the procedure outlined in Method A using N-methylhex-5-en-1-amine and **SI-6** as starting material. **SI-19** was purified by RP-HPLC (C18, 20% to 80% MeCN+0.1% TFA in water+0.1% TFA) and was isolated as a brown solid after lyophilization (0.8 g, 1.00 mmol, 50% yield). Isolated as a mixture of isomers.

LCMS: MS (ESI) mass calcd. for C38H59ClN6O7: 747.38 m/z; Found 747.88 [M+H]$^+$.

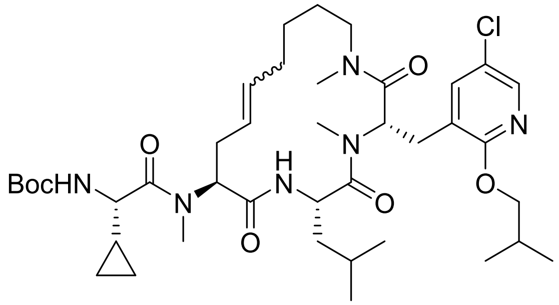

Tert-butyl ((S)-2-(((3S,6S,9S)-3-((5-chloro-2-isobutoxypyridin-3-yl)methyl)-6-isobutyl-1,4-dimethyl-2,5,8-trioxo-1,4,7-triazacyclohexadec-11-en-9-yl)(methyl)amino)-1-cyclopropyl-2-oxoethyl)carbamate (SI-20) was synthesized using the procedure outlined in Method A using N-methylhex-5-en-1-amine and **SI-7** as starting material. **SI-20** was purified by RP-HPLC (C18, 20% to 80% MeCN+0.1% TFA in water+0.1% TFA) and was isolated as a brown solid after lyophilization (1.65 g, 2.13 mmol, 57% yield). Isolated as a mixture of isomers.

LCMS: MS (ESI) mass calcd. for C40H63ClN6O7: 775.43 m/z; Found 775.94 [M+H]$^+$.

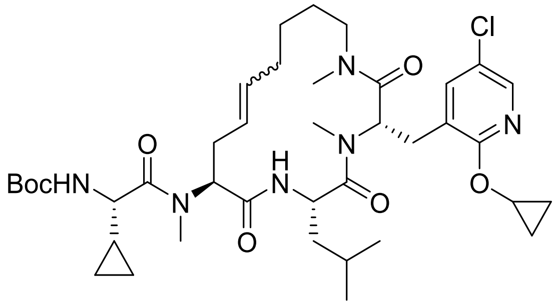

Tert-butyl ((S)-2-(((3S,6S,9S)-3-((5-chloro-2-cyclopropoxypyridin-3-yl)methyl)-6-isobutyl-1,4-dimethyl-2,5,8-trioxo-1,4,7-triazacyclohexadec-11-en-9-yl)(methyl)amino)-1-cyclopropyl-2-oxoethyl)carbamate (SI-21) was synthesized using the procedure outlined in Method A using N-methylhex-5-en-1-amine and **SI-4** as starting material. **SI-21** was purified by

column chromatography (SiO_2, 0 to 100% EtOAc) and was isolated as a brown solid (52 g, 68.5 mmol)

LCMS: MS (ESI) mass calcd. for $C_{39}H_{60}ClN_6O_7$: 758.41 m/z; Found 759.4 $[M+H]^+$.

^{1}H NMR: (400 MHz, CD_3OD_3) δ= 8.17 - 8.05 (m, 1H), 8.00 - 7.80 (m, 1H), 7.53 - 7.30 (m, 1H), 5.55 - 5.07 (m, 2H), 5.00 - 4.83 (m, 1H), 4.45 - 4.02 (m, 4H), 3.17 - 2.89 (m, 5H), 2.86 - 2.65 (m, 7H), 2.61 - 2.43 (m, 1H), 1.91 (s, 3H), 1.88 - 1.75 (m, 1H), 1.63 - 1.46 (m, 2H), 1.43 - 1.21 (m, 13H), 1.10-0.96 (m, 2H), 0.86 - 0.63 (m, 11H), 0.50 - 0.15 (m, 4H)

Method B – Hydrogenation

Representative procedure for Method B:

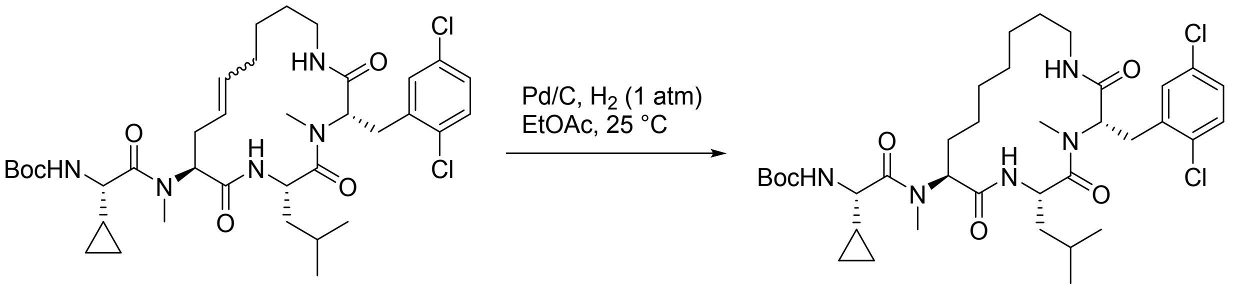

Tert-butyl ((S)-1-cyclopropyl-2-(((3S,6S,9S)-3-(2,5-dichlorobenzyl)-6-isobutyl-4-methyl-2,5,8-trioxo-1,4,7-triazacyclohexadecan-9-yl)(methyl)amino)-2-oxoethyl)carbamate (SI-22):

tert-butyl ((S)-1-cyclopropyl-2-(((3S,6S,9S)-3-(2,5-dichlorobenzyl)-6-isobutyl-4-methyl-2,5,8-trioxo-1,4,7-triazacyclohexadec-11-en-9-yl)(methyl)amino)-2-oxoethyl)carbamate (1.6 g, 2.2 mmol, 1 eq.) was dissolved in EtOAc (200 mL). To the solution was added 10% Palladium on Carbon (1.6 g, 1.5 mmol, 0.68 eq.) as a solid. The reaction vessel was evacuated and backfilled with hydrogen gas 10 times. The reaction was stirred under 1 atm of hydrogen for 2 hours. Consumption of the starting material was confirmed by LCMS. The reaction was filtered through a pad of Celite and the filtrate was concentrated by rotary evaporation to give tert-butyl ((S)-1-cyclopropyl-2-(((3S,6S,9S)-3-(2,5-dichlorobenzyl)-6-isobutyl-4-methyl-2,5,8-trioxo-1,4,7-triazacyclohexadecan-9-yl)(methyl)amino)-2-oxoethyl)carbamate (1.5 g, 2.1 mmol, 93 % yield) as a pale brown solid which was carried on without further purification. An aliquot was further purified by reverse-phase HPLC (50-100% acetonitrile in water, 0.05% formic acid buffer) for analytical purposes.

HRMS: MS (ESI) mass calcd. for $C_{36}H_{56}Cl_2N_5O_6$: 724.36 m/z; Found 724.3622 $[M+H]^+$.

^{1}H NMR: (400 MHz, CD_3OD_3) δ=7.66 - 7.57 (m, 1H), 7.53 - 7.45 (m, 1H), 7.37 - 7.20 (m, 1H), 5.38 - 5.23 (m, 1H), 4.93 - 4.84 (m, 2H), 4.73 - 4.64 (m, 1H), 4.62 - 4.31 (m, 2H), 3.94 - 3.76 (m, 1H), 3.72 - 3.64 (m, 1H), 3.48 - 3.26 (m, 1H), 3.26 - 3.19 (m, 1H), 3.18 - 2.91 (m, 5H), 1.90 - 1.74 (m, 1H), 1.73 - 1.54 (m, 17H), 1.51 - 1.35 (m, 2H), 1.31 - 1.21 (m, 1H), 1.12 - 1.04 (m, 2H), 1.00 - 0.95 (m, 2H), 0.93 - 0.78 (m, 3H), 0.75 (br s, 1H)

^{13}C NMR (101MHz, CD_3OD_3) δ = 223.748, 209.755, 174.41, 174.118, 172.277, 172.145, 171.86, 171.784, 171.481, 170.779, 170.487, 170.115, 168.84, 156.235, 156.227, 137.786, 137.354, 132.933, 132.444, 132.341, 132.273, 132.144, 131.795, 131.184, 130.588, 128.854, 128.763, 128.289, 79.341, 79.277, 59.963, 58.878, 56.484, 55.896, 53.145, 52.432, 48.759, 48.736, 48.709, 48.531, 48.227, 47.802, 47.587, 47.374, 46.949, 46.668, 39.432, 39.235, 38.95, 37.926, 36.81, 31.889, 31.775, 31.688, 30.136, 30.003, 29.1, 28.891, 28.622, 28.478, 28.383, 28.079, 27.95, 27.635, 27.464, 27.411, 27.271, 26.967, 26.315, 26.265, 26.14, 25.84, 24.418, 23.924, 23.757, 23.617, 23.386, 23.044, 22.911, 22.494, 22.35, 22.126, 22.069, 21.09, 19.614, 19.269, 18.923, 12.598, 12.461, 12.203, 2.524, 2.437, 2.247, 1.788, 1.727, 1.53, 0.767

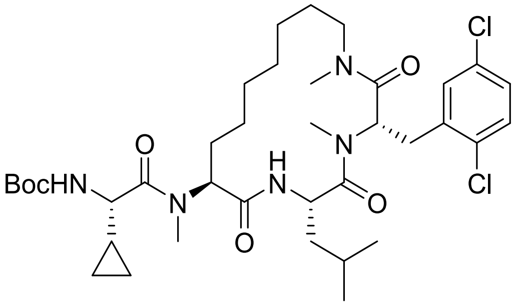

Tert-butyl ((S)-1-cyclopropyl-2-(((3S,6S,9S)-3-(2,5-dichlorobenzyl)-6-isobutyl-1,4-dimethyl-2,5,8-trioxo-1,4,7-triazacyclohexadecan-9-yl)(methyl)amino)-2-oxoethyl)carbamate (SI-23) was synthesized using the procedure outlined in Method B using **SI-15** as a starting material to give tert-butyl ((S)-1-cyclopropyl-2-(((3S,6S,9S)-3-(2,5-dichlorobenzyl)-6-isobutyl-1,4-dimethyl-2,5,8-trioxo-1,4,7-triazacyclohexadecan-9-yl)(methyl)amino)-2-oxoethyl)carbamate (2.1 g, 2.8 mmol) as a pale brown solid which was carried on without further purification. An aliquot was further purified by reverse-phase HPLC (50-100% acetonitrile in water, 0.05% formic acid buffer) for analytical purposes.

LCMS: MS (ESI) mass calcd. for $C_{37}H_{58}Cl_2N_5O_6$: 738.37 m/z; Found 738.4 $[M+H]^+$.

^{1}H NMR: (400 MHz, CD_3OD_3) δ = 8.30 - 8.04 (m, 1H), 7.24 - 7.13 (m, 1H), 7.09 - 6.96 (m, 2H), 6.91 - 6.36 (m, 1H), 4.75 - 4.66 (m, 1H), 4.31 (br s, 1H), 4.25 - 3.91 (m, 3H), 3.19 (br s, 1H), 3.01 (br s, 3H), 2.82 - 2.71 (m, 3H), 2.69 - 2.58 (m, 3H), 2.53 (s, 1H), 2.06 - 1.72 (m, 1H), 1.54 - 1.40 (m, 2H), 1.39 - 1.31 (m, 1H), 1.31 - 1.18 (m, 13H), 1.18 - 1.07 (m, 5H), 1.07 (br s, 2H), 0.92 - 0.85 (m, 1H), 0.65 (br s, 4H), 0.61 - 0.51 (m, 3H), 0.40 - -0.04 (m, 4H)

^{13}C NMR (101 MHz, CD_3OD_3) δ = 224.022, 223.908, 223.46, 223.156, 223.13, 223.084, 222.758, 221.415, 221.195, 221.153, 220.975, 220.967, 220.865, 220.857, 220.755, 220.732, 220.182, 218.994, 215.556, 212.005, 211.007, 210.885, 206.579, 197.499, 192.87, 192.854, 188.605, 184.7, 183.251, 182.325, 172.941, 172.338, 172.076, 171.985, 171.871, 171.264, 169.314, 156.223, 137.582, 132.831, 132.125, 132.038, 130.516, 128.828, 128.221, 126.362, 119.979, 112.694, 79.201, 58.821, 56.294, 52.534, 48.77, 48.645, 48.588, 48.25, 48.167, 47.954, 47.825, 47.61, 47.396, 46.971, 39.011, 38.908, 34.848, 34.662, 32.659, 30.007, 29.362, 28.565, 27.859, 27.457, 27.294, 27.085, 26.869, 26.228, 25.78, 24.399, 24.308, 24.008, 23.867, 23.56, 23.446, 23.15, 22.494, 21.113, 20.058, 20.043, 19.739, 13.554, 13.069, 12.26, 2.179, 2.031, 1.788, 0.9.

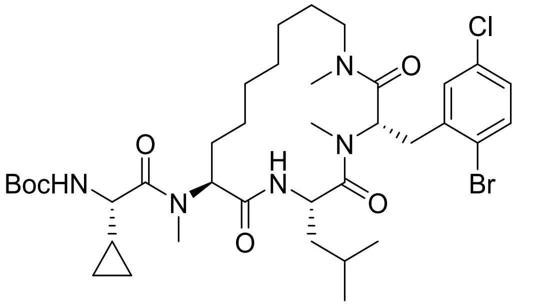

Tert-butyl ((S)-2-(((3S,6S,9S)-3-(2-bromo-5-chlorobenzyl)-6-isobutyl-1,4-dimethyl-2,5,8-trioxo-1,4,7-triazacyclohexadecan-9-yl)(methyl)amino)-1-cyclopropyl-2-oxoethyl)carbamate (SI-24) was synthesized using the procedure outlined in Method B with the following modification: 0.1 equiv of Pt_2O was used instead of Pd/C. **SI-24** was isolated as an off white solid (11.8 g, 15.3 mmol, 98% yield). The reaction was used without further purification.

LCMS: MS (ESI) mass calcd. for $C_{37}H_{58}BrClN_5O_6$: 782.32 m/z; Found 782.20 $[M+H]^+$.

^{1}H NMR: (400 MHz, DMSO-d6) δ 8.21-8.50 (m, 1H), 7.49-7.64 (m, 1H), 7.13-7.38 (m, 2H), 6.81 (d, J = 3.6 Hz, 1H), 4.81 (d, J = 12.4 Hz, 1H), 4.32-4.50 (m, 1H), 4.13-4.27 (m, 2H), 3.03-3.36 (m,

2H), 2.69-2.98 (m, 10H), 2.53-2.64 (m, 1H), 1.79-2.04 (m, 1H), 1.49-1.65 (m, 2H), 1.31-1.43 (m, 14H), 0.99-1.27 (m, 8H), 0.80-0.87 (m, 3H), 0.66 (d, J = 5.6 Hz, 3H), 0.31-0.47 (m, 2H), 0.11-0.28 (m, 2H)

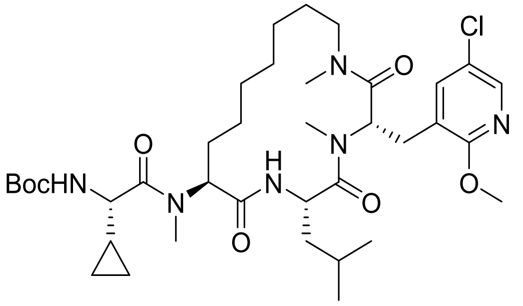

Tert-butyl ((S)-2-(((3S,6S,9S)-3-((5-chloro-2-methoxypyridin-3-yl)methyl)-6-isobutyl-1,4-dimethyl-2,5,8-trioxo-1,4,7-triazacyclohexadecan-9-yl)(methyl)amino)-1-cyclopropyl-2-oxoethyl)carbamate (SI-25) was synthesized using the procedure outlined in Method B. **SI-25** was isolated as an off white solid (0.45 g, 0.614 mmol, 100% yield). The reaction was used without further purification.

LCMS: MS (ESI) mass calcd. for C37H59ClN6O7: 735.36 m/z; Found 735.78 $[M+H]^+$.

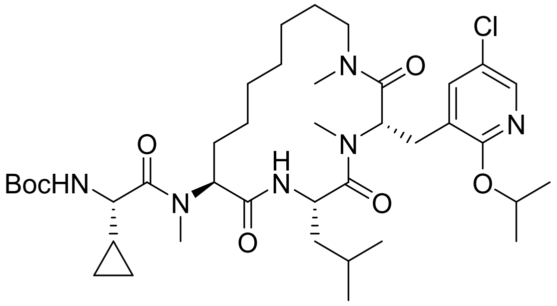

Tert-butyl ((S)-2-(((3S,6S,9S)-3-((5-chloro-2-isopropoxypyridin-3-yl)methyl)-6-isobutyl-1,4-dimethyl-2,5,8-trioxo-1,4,7-triazacyclohexadecan-9-yl)(methyl)amino)-1-cyclopropyl-2-oxoethyl)carbamate (SI-26) was synthesized using the procedure outlined in Method B. **SI-26** was isolated as an off white solid (0.3 g, 0.4 mmol, 99% yield). The reaction was used without further purification.

LCMS: MS (ESI) mass calcd. for C39H63ClN6O7: 763.42 m/z; Found 763.95 $[M+H]^+$.

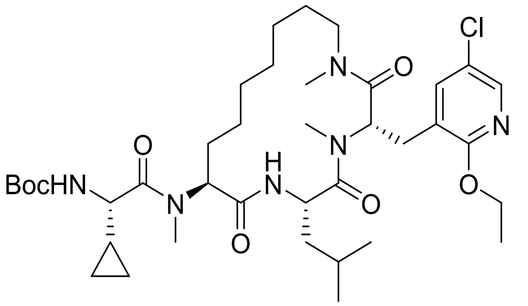

Tert-butyl ((S)-2-(((3S,6S,9S)-3-((5-chloro-2-ethoxypyridin-3-yl)methyl)-6-isobutyl-1,4-dimethyl-2,5,8-trioxo-1,4,7-triazacyclohexadecan-9-yl)(methyl)amino)-1-cyclopropyl-2-oxoethyl)carbamate (SI-27) was synthesized using the procedure outlined in Method B. **SI-27** was isolated as an off white solid (0.46 g, 0.602 mmol, 100% yield). The reaction was used without further purification.

LCMS: MS (ESI) mass calcd. for C38H61ClN6O7: 749.39 m/z; Found 749.85 $[M+H]^+$.

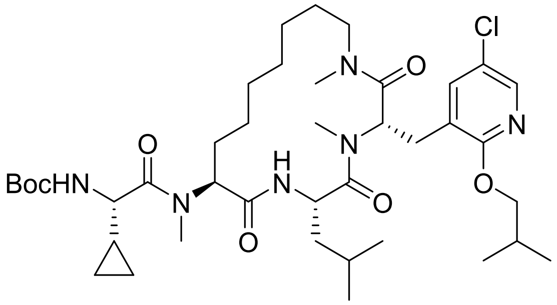

Tert-butyl ((S)-2-(((3S,6S,9S)-3-((5-chloro-2-isobutoxypyridin-3-yl)methyl)-6-isobutyl-1,4-dimethyl-2,5,8-trioxo-1,4,7-triazacyclohexadecan-9-yl)(methyl)amino)-1-cyclopropyl-2-oxoethyl)carbamate (SI-28) was synthesized using the procedure outlined in Method B . **SI-28** was isolated as an off white solid (0.44 g, 0.580 mmol, 99% yield). The reaction was used without further purification.

LCMS: MS (ESI) mass calcd. for C40H65ClN6O7: 777.45 m/z; Found 777.96 $[M+H]^+$.

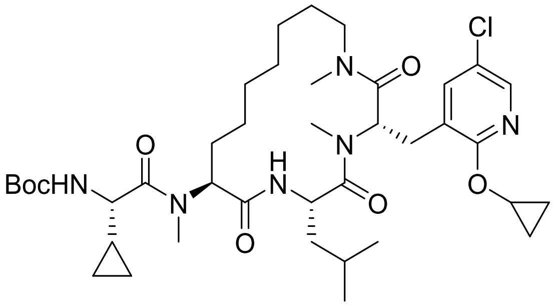

Tert-butyl ((S)-2-(((3S,6S,9S)-3-((5-chloro-2-cyclopropoxypyridin-3-yl)methyl)-6-isobutyl-1,4-dimethyl-2,5,8-trioxo-1,4,7-triazacyclohexadecan-9-yl)(methyl)amino)-1-cyclopropyl-2-oxoethyl)carbamate (SI-29) was synthesized using the procedure outlined in Method B. **SI-29** was isolated as a off-white solid (52g, 68.5 mmol, quant.) which was used without further purification.

LCMS: MS (ESI) mass calcd. for $C_{39}H_{62}ClN_6O_7$: 761.43 m/z; Found 761.4 $[M+H]^+$.

^{1}H NMR: (400 MHz, CD_3OD) δ 7.85 - 7.78 (d, J = 2.4 Hz, 1H), 7.37 - 7.29 (d, J = 2.4 Hz, 1H), 4.19 - 4.01 (m, 4H), 2.98 - 2.79 (m, 5H), 2.78 - 2.67 (m, 7H), 2.54 - 2.43 (m, 1H), 1.48 - 1.29 (m, 4H), 1.26 - 1.20 (m, 13H), 1.18 - 1.08 (m, 5H), 0.96 - 0.82 (m, 2H), 0.79 - 0.67 (m, 5H), 0.53- 0.64 (m, 7H), 0.33 - 0.12 (m, 3H), 0.02 (m, 1H).

Method C – Suzuki Coupling

Representative procedure for Method C:

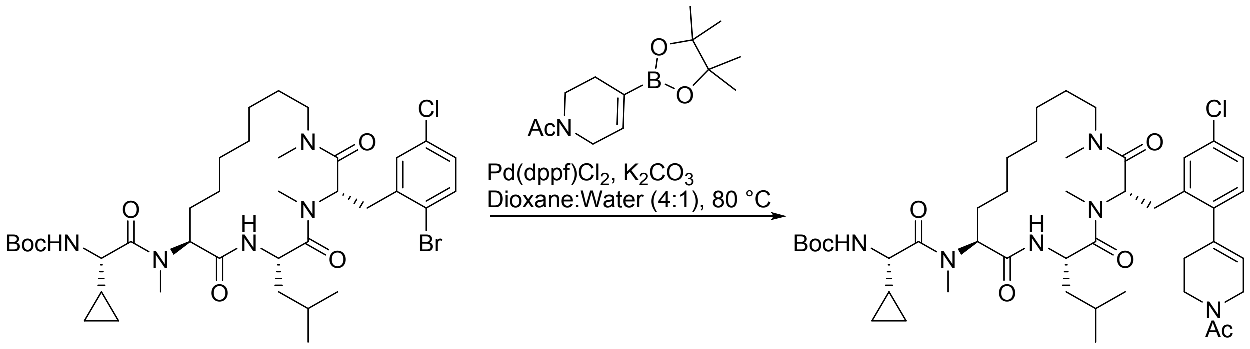

tert-butyl ((S)-2-(((3S,6S,9S)-3-(2-(1-acetyl-1,2,3,6-tetrahydropyridin-4-yl)-5-chlorobenzyl)-6-isobutyl-1,4-dimethyl-2,5,8-trioxo-1,4,7-triazacyclohexadecan-9-yl)(methyl)amino)-1-cyclopropyl-2-oxoethyl)carbamate (SI-30): SI-24 (100 mg, 1 Eq, 128 µmol), 1-(4-(4,4,5,5-tetramethyl-1,3,2-dioxaborolan-2-yl)-3,6-dihydropyridin-1(2H)-yl)ethan-1-one (48.1 mg, 1.5 Eq,

192 μmol), and $PdCl_2$(dppf) (14.0 mg, 0.15 Eq, 19.2 μmol) were dissolved in 1,4-dioxane (1 mL). 2 M K_2CO_3 (, 200 μL) was added and the reaction was degassed with a stream of N_2 (5 min). The reaction was then heated to 80 °C and stirred until the complete consumption of starting material (~2 hr). The reaction was diluted with MeCN (2 mL), filtered and directly purified by RP chromatography (15.5g C18, 30-80% MeCN+0.1% TFA in Water+0.1% TFA). **SI-30** (79.3 mg, 95.8 μmol, 75.1 %) was isolated as an off-white solid after lyophilization.

LCMS: MS (ESI) mass calcd. for $C_{44}H_{68}ClN_6O_7$: 827.48; m/z Found 827.80 $[M+H]^+$.

^{1}H NMR: (400 MHz, CD_3OD_3) δ= 7.30 - 7.20 (m, 1H), 7.17 (br s, 1H), 7.10 (br d, J = 8.2 Hz, 1H), 5.81 (br d, J = 8.2 Hz, 1H), 4.38 - 4.27 (m, 2H), 4.18 (br d, J = 8.8 Hz, 2H), 3.96 - 3.65 (m, 2H), 3.31 (t, J = 1.6 Hz, 3H), 3.18 (br d, J = 7.4 Hz, 1H), 3.09 (s, 3H), 3.00 - 2.82 (m, 6H), 2.66 (br d, J = 12.8 Hz, 1H), 2.59 - 2.36 (m, 2H), 2.35 - 2.18 (m, 1H), 2.16 (d, J = 10.8 Hz, 2H), 2.05 (br d, J = 10.6 Hz, 1H), 1.81 - 1.62 (m, 2H), 1.61 - 1.49 (m, 2H), 1.46 (br s, 3H), 1.43 (s, 9H), 1.38 - 1.15 (m, 7H), 1.15 - 0.99 (m, 2H), 0.98 - 0.86 (m, 3H), 0.85 - 0.69 (m, 3H), 0.65 - 0.15 (m, 4H)

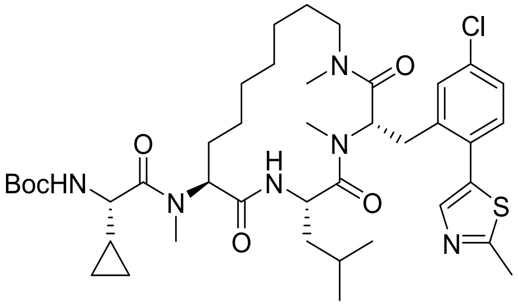

Tert-butyl ((S)-2-(((3S,6S,9S)-3-(5-chloro-2-(2-methylthiazol-5-yl)benzyl)-6-isobutyl-1,4-dimethyl-2,5,8-trioxo-1,4,7-triazacyclohexadecan-9-yl)(methyl)amino)-1-cyclopropyl-2-oxoethyl)carbamate (SI-31) was synthesized using the procedure outlined in Method C using **SI-24** and 2-methyl-5-(4,4,5,5-tetramethyl-1,3,2-dioxaborolan-2-yl)thiazole as starting material. **SI-31** was purified by RP-HPLC (C18, 20% to 80% MeCN+0.1% TFA in water+0.1% TFA) and was isolated as a fluffy off-white solid after lyophilization (101 mg, 131 μmol, 85% yield).

LCMS: MS (ESI) mass calcd. for **(SI-31)** : 772.37 m/z; Found 772.90 $[M+H]^+$.

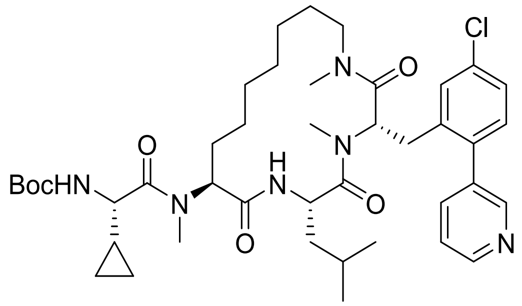

Tert-butyl ((S)-2-(((3S,6S,9S)-3-(5-chloro-2-(pyridin-3-yl)benzyl)-6-isobutyl-1,4-dimethyl-2,5,8-trioxo-1,4,7-triazacyclohexadecan-9-yl)(methyl)amino)-1-cyclopropyl-2-oxoethyl)carbamate (SI-32) was synthesized using the procedure outlined in Method C using **SI-24** and pyridin-3-ylboronic acid as starting material. **SI-32** was purified by RP-HPLC (C18, 20% to 80% ACN+0.1% TFA in water+0.1% TFA) and was isolated as a fluffy off-white solid after lyophilization (0.055 g, 0.070 mmol, 92% yield).

LCMS: MS (ESI) mass calcd. for $C_{42}H_{62}ClN_6O_6$: 781.44 m/z; Found 781.20 $[M+H]^+$.

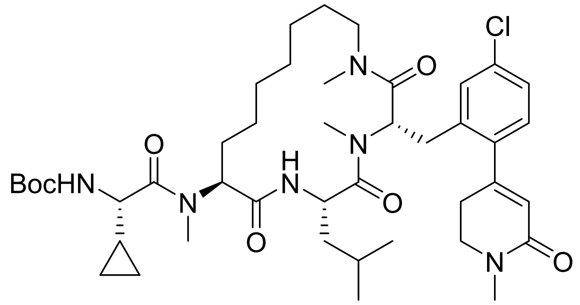

tert-butyl ((S)-2-(((3S,6S,9S)-3-(5-chloro-2-(1-methyl-6-oxo-1,2,3,6-tetrahydropyridin-4-yl)benzyl)-6-isobutyl-1,4-dimethyl-2,5,8-trioxo-1,4,7-triazacyclohexadecan-9-yl)(methyl)amino)-1-cyclopropyl-2-oxoethyl)carbamate (SI-33) was synthesized using the procedure outlined in Method C using **SI-24** and 1-methyl-4-(trifluoro-l4-boraneyl)-5,6-dihydropyridin-2(1H)-one, potassium salt as starting material. **SI-33** was purified by RP-HPLC (C18, 20% to 80% MeCN+0.1% TFA in water+0.1% TFA) and was isolated as a fluffy off-white solid after lyophilization (0.045 g, 0.055 mmol, 43% yield).

LCMS: MS (ESI) mass calcd. for C43H65ClN6O7: 813.48 m/z; Found 813.93 $[M+H]^+$.

Method D – Dipeptide Addition

Representative procedure for Method D:

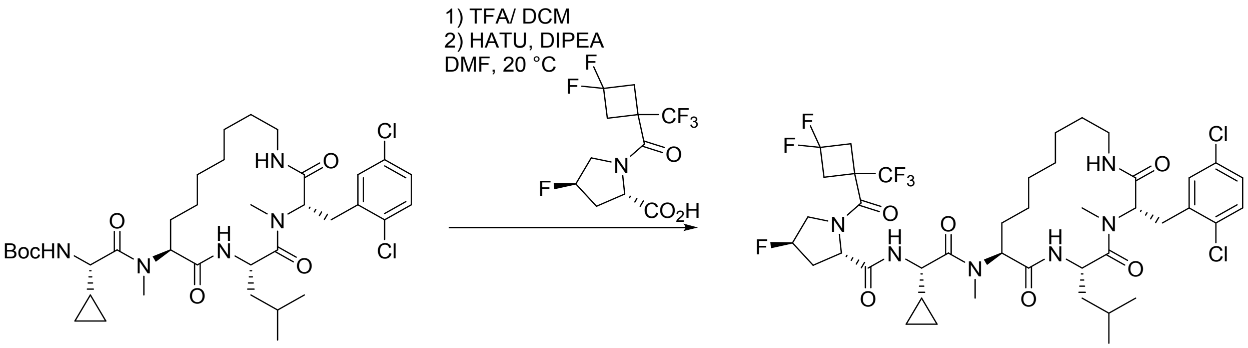

(2S,4R)-N-((S)-1-cyclopropyl-2-(((3S,6S,9S)-3-(2,5-dichlorobenzyl)-6-isobutyl-4-methyl-2,5,8-trioxo-1,4,7-triazacyclohexadecan-9-yl)(methyl)amino)-2-oxoethyl)-1-(3,3-difluoro-1-(trifluoromethyl)cyclobutane-1-carbonyl)-4-fluoropyrrolidine-2-carboxamide (Compound 23): tert-butyl ((S)-1-cyclopropyl-2-(((3S,6S,9S)-3-(2,5-dichlorobenzyl)-6-isobutyl-4-methyl-2,5,8-trioxo-1,4,7-triazacyclohexadecan-9-yl)(methyl)amino)-2-oxoethyl)carbamate (0.05 g, 0.069 mmol, 1 eq.) was dissolved in DCM (1 mL) and TFA (1 mL) and allowed to sit at room temperature for 30 minutes until the Boc group was removed as confirmed by LCMS. The reaction mixture was concentrated by rotary evaporation. The residue was resuspended in toluene and concentrated to remove residual TFA, and this procedure was repeated two times. The crude residue was used in the next reaction without further purification.

To a vial containing the residue was added a solution of (2S,4R)-1-(3,3-difluoro-1-(trifluoromethyl)cyclobutane-1-carbonyl)-4-fluoropyrrolidine-2-carboxylic acid (0.026 g, 0.083 mmol, 1.2 eq.), HATU (0.031 g, 0.083 mmol, 1 eq.), and DIPEA (0.031 g, 0.042 mL, 0.24 mmol, 3.5 eq.). in DMF (1 mL). The reaction mixture was checked by pH paper and DIPEA was added in 1 eq. portions until the reaction was confirmed to be basic (pH ~9). The reaction was allowed to sit for 2 hours at room temperature. The reaction mixture was diluted in water (100 mL) and extracted with ethyl acetate (100 mL x 3). The combined organics were washed with brine (150 mL), dried over anhydrous magnesium sulfate, and filtered over celite. The organic extract was concentrated under rotary evaporation to give a yellow-orange oil. The crude material was purified by reverse-phase HPLC (50-100% acetonitrile in water, 0.05% formic acid buffer) and the eluent was lyophilized to give (2S,4R)-N-((S)-1-cyclopropyl-2-(((3S,6S,9S)-3-(2,5-dichlorobenzyl)-6-

isobutyl-4-methyl-2,5,8-trioxo-1,4,7-triazacyclohexadecan-9-yl)(methyl)amino)-2-oxoethyl)-1-(3,3-difluoro-1-(trifluoromethyl)cyclobutane-1-carbonyl)-4-fluoropyrrolidine-2-carboxamide (0.044 g, 0.048 mmol, 69 % yield) as a fluffy white solid.

HRMS: MS (ESI) mass calcd. For $C_{42}H_{57}Cl_2F_6N_6O_6$: 925.36 m/z; Found 925.3624 $[M+H]^+$.

^{1}H NMR: (400 MHz, CD_3OD_3) δ = 7.66 - 7.56 (m, 1H), 7.53 - 7.42 (m, 2H), 7.35 - 7.21 (m, 1H), 5.61 - 5.37 (m, 1H), 5.11 - 5.05 (m, 1H), 4.95 - 4.65 (m, 4H), 4.55 - 4.29 (m, 1H), 4.15 - 4.00 (m, 1H), 3.96 - 3.78 (m, 2H), 3.72 - 3.61 (m, 2H), 3.45 - 3.27 (m, 6H), 3.27 - 3.21 (m, 1H), 3.13 - 3.04 (m, 2H), 3.03 - 2.92 (m, 2H), 2.84 - 2.68 (m, 1H), 2.44 - 2.09 (m, 2H), 1.96 - 1.73 (m, 2H), 1.68 (br s, 13H), 1.11 - 1.02 (m, 2H), 0.99 - 0.95 (m, 2H), 0.92 - 0.87 (m, 1H), 0.86 - 0.77 (m, 2H), 0.76 - 0.47 (m, 4H)

^{13}C NMR (101 MHz, CD_3OD_3) δ = 174.156, 172.13, 171.644, 171.625, 171.105, 170.585, 170.13, 168.836, 165.626, 137.889, 137.441, 132.971, 132.501, 132.395, 132.319, 132.205, 131.863, 131.207, 131.135, 130.615, 128.786, 128.3, 126.362, 118.913, 116.185, 93.096, 91.324, 63.697, 63.663, 60.256, 60.017, 59.557, 59.231, 56.404, 55.831, 54.344, 54.219, 52.762, 52.045, 48.778, 48.531, 48.25, 47.611, 46.971, 46.725, 41.291, 41.029, 40.764, 39.553, 39.436, 39.261, 39.034, 38.491, 37.956, 36.818, 34.966, 31.942, 31.726, 30.189, 30.03, 28.884, 28.368, 28.132, 27.013, 26.436, 26.319, 25.829, 24.6, 24.524, 23.867, 23.773, 23.681, 23.116, 22.923, 22.513, 22.399, 22.228, 22.092, 21.113, 19.64, 19.307, 19.132, 11.999, 11.714, 2.486, 1.989, 1.921, 1.856, 1.207

Final Compounds Made Using Method D

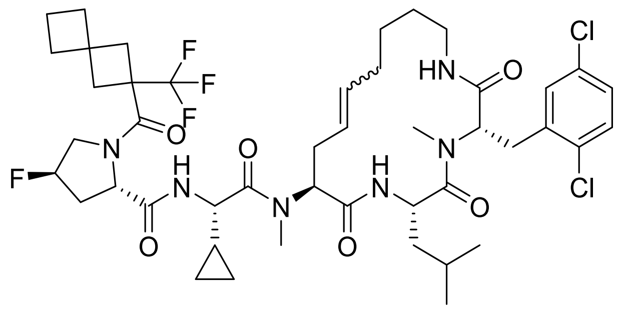

(2S,4R)-N-((S)-1-cyclopropyl-2-(((3S,6S,9S)-3-(2,5-dichlorobenzyl)-6-isobutyl-4-methyl-2,5,8-trioxo-1,4,7-triazacyclohexadec-11-en-9-yl)(methyl)amino)-2-oxoethyl)-4-fluoro-1-(2-(trifluoromethyl)spiro[3.3]heptane-2-carbonyl)pyrrolidine-2-carboxamide (Compound 19)

was synthesized using method D using **SI-14** and **SI-9** as starting materials. **Compound 19** was purified using RP-HPLC. **Compound 19** (11.46 mg, 0.012 mmol, 28.0% Yield) was isolated as a fluffy white solid after lyophilization. Isolated as a~9:1 mixture of E:Z isomers. Major isomer was not elucidated. Data given for major isomer

LCMS: MS (ESI) mass calcd. for $C_{45}H_{61}Cl_2F_4N_6O_6$: 927.3966 m/z; Found 927.3941 $[M+H]^+$.

^{1}H NMR: (400 MHz, 7621) δ 7.68 - 7.55 (m, 1H), 7.55 - 7.45 (m, 1H), 7.39 - 7.21 (m, 1H), 5.74 - 5.58 (m, 1H), 5.57 - 5.36 (m, 2H), 5.36 - 5.23 (m, 1H), 4.90 - 4.69 (m, 3H), 4.64 - 4.54 (m, 1H), 4.34 - 4.00 (m, 2H), 3.95 - 3.75 (m, 2H), 3.73 - 3.63 (m, 2H), 3.43 - 3.24 (m, 3H), 3.01 - 2.90 (m, 5H), 2.81 - 2.61 (m, 4H), 2.45 - 2.04 (m, 8H), 2.03 - 1.89 (m, 3H), 1.88 - 1.54 (m, 6H), 1.53 - 1.28 (m, 3H), 1.19 - 0.95 (m, 6H), 0.93 - 0.64 (m, 5H)

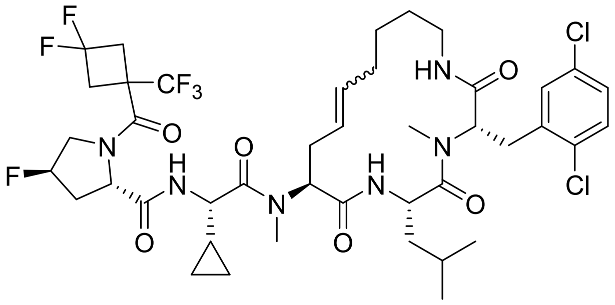

(2S,4R)-N-((S)-1-cyclopropyl-2-(((3S,6S,9S)-3-(2,5-dichlorobenzyl)-6-isobutyl-4-methyl-2,5,8-trioxo-1,4,7-triazacyclohexadec-11-en-9-yl)(methyl)amino)-2-oxoethyl)-1-(3,3-difluoro-1-(trifluoromethyl)cyclobutane-1-carbonyl)-4-fluoropyrrolidine-2-carboxamide (Compound 20): was synthesized using method D using **SI-8** and **SI-14** as starting materials. **Compound 20** was purified using RP-HPLC. **Compound 20** (29.78 mg, 0.032 mmol, 23.3% Yield) was isolated as a fluffy white solid after lyophilization. Isolated as a~9:1 mixture of E:Z isomers. Major isomer was not elucidated. Data given for major isomer

LCMS: MS (ESI) mass calcd. for $C_{42}H_{55}Cl_2F_6N_6O_6$: 923.3464 m/z; Found 923.3491 $[M+H]^+$.

^{1}H NMR: (400 MHz, D_3COD) δ 7.60 - 7.45 (m, 1H), 7.36 - 7.27 (m, 1H), 7.18-7.24 (m, 1H), 7.08 - 6.95 (m, 1H), 5.02 (dd, J = 3.0, 11.8 Hz, 3H), 4.95 (br dd, J = 3.6, 10.8 Hz, 1H), 4.72 - 4.54 (m, 2H), 4.52 - 4.42 (m, 1H), 4.37 (s, 1H), 4.09 - 3.72 (m, 2H), 3.43-3.66 (m, 2H), 3.41 - 3.23 (m, 3H), 3.18 - 2.96 (m, 5H), 2.77 (s, 1H), 2.74 - 2.60 (m, 5H), 2.56 - 2.38 (m, 1H), 2.20 - 1.72 (m, 4H),

1.72 - 1.41 (m, 2H), 1.40 - 1.24 (m, 2H), 1.24 - 1.00 (m, 3H), 0.98 - 0.70 (m, 6H), 0.70 - 0.27 (m, 5H)

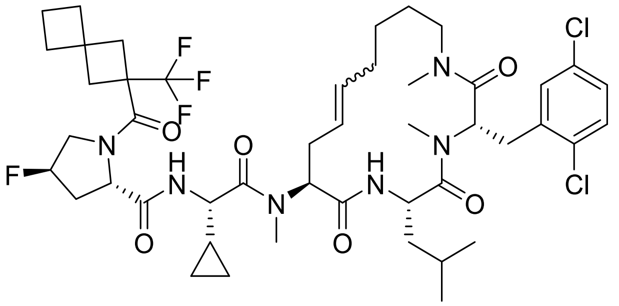

(2S,4R)-N-((S)-1-cyclopropyl-2-(((3S,6S,9S)-3-(2,5-dichlorobenzyl)-6-isobutyl-1,4-dimethyl-2,5,8-trioxo-1,4,7-triazacyclohexadec-11-en-9-yl)(methyl)amino)-2-oxoethyl)-4-fluoro-1-(2-(trifluoromethyl)spiro[3.3]heptane-2-carbonyl)pyrrolidine-2-carboxamide (Compound 21) was synthesized using method D using **SI-15** and **SI-9** as starting materials. **Compound 21** was purified using RP-HPLC. **Compound 21** (23.2 mg, 0.025 mmol, 53.50% Yield) was isolated as a fluffy white solid after lyophilization.

LCMS: MS (ESI) mass calcd. for $C_{46}H_{63}Cl_2F_4N_6O_6$: 941.4122 m/z; Found 941.4153 $[M+H]^+$.

^{1}H NMR: (400 MHz, D_3COD) δ 7.37 - 7.26 (m, 1H), 7.22 - 7.03 (m, 2H), 5.53 - 5.35 (m, 1H), 5.34 - 5.09 (m, 2H), 4.91 (br s, 1H), 4.47 (br s, 2H), 4.42 - 4.32 (m, 1H), 4.31 - 4.25 (m, 1H), 4.24 - 4.09 (m, 1H), 3.94 - 3.72 (m, 1H), 3.65 - 3.45 (m, 1H), 3.32 - 3.24 (m, 1H), 3.18 - 2.92 (m, 4H), 2.91 - 2.81 (m, 3H), 2.80 - 2.59 (m, 6H), 2.55 - 2.35 (m, 5H), 2.10 - 1.81 (m, 8H), 1.43 (br s, 6H), 1.32 - 1.14 (m, 3H), 1.13 - 1.02 (m, 1H), 1.01 - 0.90 (m, 1H), 0.88 - 0.82 (m, 2H), 0.78 (br d, J = 6.4 Hz, 1H), 0.73 - 0.64 (m, 3H), 0.62 - 0.27 (m, 4H)

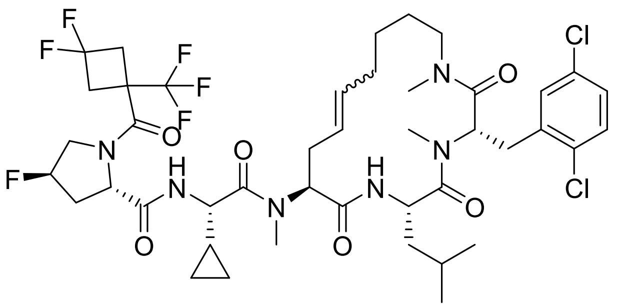

(2S,4R)-N-((S)-1-cyclopropyl-2-(((3S,6S,9S)-3-(2,5-dichlorobenzyl)-6-isobutyl-1,4-dimethyl-2,5,8-trioxo-1,4,7-triazacyclohexadec-11-en-9-yl)(methyl)amino)-2-oxoethyl)-1-(3,3-difluoro-1-(trifluoromethyl)cyclobutane-1-carbonyl)-4-fluoropyrrolidine-2-carboxamide (Compound 22) was synthesized using method D using **SI-15** and **SI-8** as starting materials. **Compound 22** was purified using RP-HPLC. **Compound 22** (11.66 mg, 0.012 mmol, 28.20% Yield) was isolated as a fluffy white solid after lyophilization.

LCMS: MS (ESI) mass calcd. for $C_{43}H_{57}Cl_2F_6N_6O_6$: 937.3621 m/z; Found 937.3528 $[M+H]^+$.

^{1}H NMR: (400 MHz, D_3COD) δ 8.20 - 7.99 (m, 1H), 7.37 - 7.26 (m, 1H), 7.25 - 7.04 (m, 2H), 5.55 - 5.36 (m, 1H), 5.33 - 5.17 (m, 3H), 4.44 - 4.04 (m, 4H), 3.93 - 3.38 (m, 5H), 3.13 - 3.01 (m, 4H), 2.78 (s, 5H), 2.75 - 2.61 (m, 4H), 2.39 (br s, 2H), 2.15 - 1.95 (m, 2H), 1.92 - 1.78 (m, 1H), 1.71 (br d, J = 14.2Hz, 2H), 1.49 - 1.37 (m, 1H), 1.31 (br s, 8H), 0.94 - 0.60 (m, 7H), 0.58 - 0.25 (m, 3H)

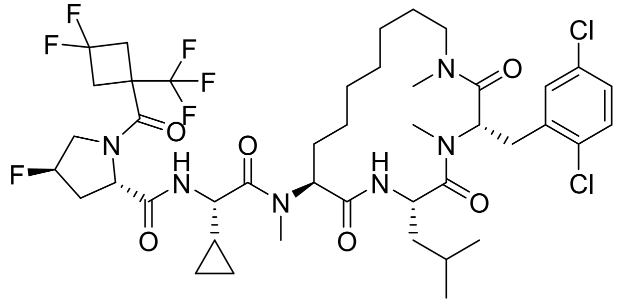

(2S,4R)-N-((S)-1-cyclopropyl-2-(((3S,6S,9S)-3-(2,5-dichlorobenzyl)-6-isobutyl-1,4-dimethyl-2,5,8-trioxo-1,4,7-triazacyclohexadecan-9-yl)(methyl)amino)-2-oxoethyl)-1-(3,3-difluoro-1-(trifluoromethyl)cyclobutane-1-carbonyl)-4-fluoropyrrolidine-2-carboxamide (Compound 24) was synthesized using method D using **SI-23** and **SI-8** as starting materials. **Compound 24** was purified using RP-HPLC. **Compound 24** (22.34 mg, 0.024 mmol, 25.30% Yield) was isolated as a fluffy white solid after lyophilization.

LCMS: MS (ESI) mass calcd. for $C_{43}H_{59}Cl_2F_6N_6O_6$: 939.3777 m/z; Found 939.3799 $[M+H]^+$.

^{1}H NMR: (400 MHz, D_3COD) δ= 7.31 - 7.25 (m, 1H), 7.23 - 7.13 (m, 2H), 5.29 - 5.11 (m, 1H), 4.80 (br s, 2H), 4.71 (br s, 2H), 4.52 (br s, 2H), 4.35 - 4.15 (m, 2H), 3.89 - 3.74 (m, 1H), 3.68 - 3.50 (m, 1H), 3.44 - 3.30 (m, 2H), 3.18 - 2.95 (m, 7H), 2.94 - 2.70 (m, 6H), 2.66 - 2.36 (m, 2H),

2.29 - 1.85 (m, 2H), 1.71 - 1.44 (m, 3H), 1.40 - 0.99 (m, 11H), 0.95 - 0.76 (m, 4H), 0.74 - 0.64 (m, 3H), 0.56 - 0.16 (m, 4H)

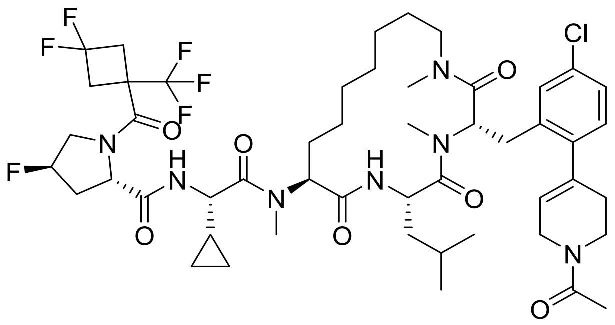

(2S,4R)-N-((S)-2-(((3S,6S,9S)-3-(2-(1-acetyl-1,2,3,6-tetrahydropyridin-4-yl)-5-chlorobenzyl)-6-isobutyl-1,4-dimethyl-2,5,8-trioxo-1,4,7-triazacyclohexadecan-9-yl)(methyl)amino)-1-cyclopropyl-2-oxoethyl)-1-(3,3-difluoro-1-(trifluoromethyl)cyclobutane-1-carbonyl)-4-fluoropyrrolidine-2-carboxamide (Compound 25) was synthesized using method D using **SI-30** and **SI-8** as starting materials. **Compound 25** was purified using RP-HPLC. **Compound 25** (26.32 mg, 0.025 mmol, 20.10% Yield) was isolated as a fluffy white solid after lyophilization.

LCMS: MS (ESI) mass calcd. for $C_{51}H_{68}ClF_6N_7O_7$: 1028.48 m/z; Found 1028.4835 $[M+H]^+$.

^{1}H NMR: (400 MHz, D_3COD) δ = 7.31 - 7.20 (m, 1H), 7.17 (br s, 1H), 7.10 (d, J = 8.2 Hz, 1H), 5.81 (br d, J = 8.4 Hz, 1H), 5.40 - 5.21 (m, 1H), 4.90 (br s, 1H), 4.79 - 4.63 (m, 2H), 4.60 - 4.51 (m, 1H), 4.37 - 4.23 (m, 2H), 4.23 - 4.09 (m, 2H), 4.08 - 3.77 (m, 2H), 3.77 - 3.66 (m, 2H), 3.54 - 3.41 (m, 1H), 3.25 - 3.15 (m, 3H), 3.15 - 3.10 (m, 3H), 3.09 - 3.00 (m, 1H), 2.99 - 2.91 (m, 3H), 2.90 - 2.79 (m, 3H), 2.70 - 2.62 (m, 1H), 2.60 - 2.33 (m, 3H), 2.16 (d, J = 10.8 Hz, 3H), 2.13 - 1.91 (m, 2H), 1.77 - 1.60 (m, 2H), 1.60 - 1.52 (m, 1H), 1.52 - 1.33 (m, 6H), 1.33 - 1.21 (m, 4H), 1.21 - 1.11 (m, 2H), 1.11 - 1.00 (m, 1H), 0.97 (br d, J = 6.2 Hz, 2H), 0.94 - 0.84 (m, 1H), 0.84 - 0.70 (m, 3H), 0.70 - 0.27 (m, 4H)

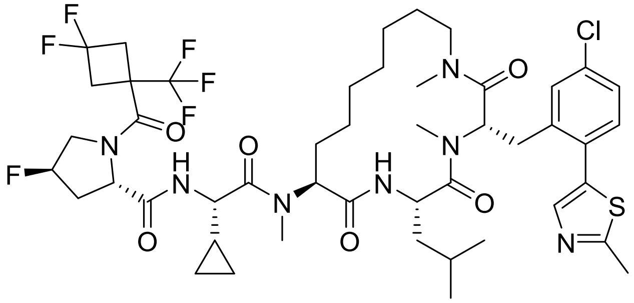

(2S,4R)-N-((S)-2-(((3S,6S,9S)-3-(5-chloro-2-(2-methylthiazol-5-yl)benzyl)-6-isobutyl-1,4-dimethyl-2,5,8-trioxo-1,4,7-triazacyclohexadecan-9-yl)(methyl)amino)-1-cyclopropyl-2-oxoethyl)-1-(3,3-difluoro-1-(trifluoromethyl)cyclobutane-1-carbonyl)-4-fluoropyrrolidine-2-carboxamide (Compound 26) was synthesized using method D using **SI-31** and **SI-8** as starting materials. **Compound 26** was purified using RP-HPLC. **Compound 26** (22 mg, 39 μmol 58% Yield) was isolated as a fluffy white solid after lyophilization.

LCMS: MS (ESI) mass calcd. for $C_{47}H_{63}ClF_6N_7O_6S$: 1002.4153 m/z; Found 1002.4130 $[M+H]^+$.

^{1}H NMR: (400 MHz, D_3COD) δ 7.74 - 7.65 (m, 1H), 7.39 - 7.28 (m, 3H), 5.42 - 5.23 (m, 1H), 4.95 - 4.89 (m, 2H), 4.82 - 4.69 (m, 2H), 4.67 - 4.52 (m, 2H), 4.40 - 4.20 (m, 2H), 4.02 - 3.85 (m, 1H), 3.81 - 3.61 (m, 1H), 3.57 - 3.36 (m, 2H), 3.29 - 3.10 (m, 7H), 2.90 - 2.82 (m, 6H), 2.65 - 2.49 (m, 2H), 2.23 - 1.98 (m, 2H), 1.78 - 1.61 (m, 2H), 1.58 - 1.12 (m, 14H), 1.05 - 0.88 (m, 4H), 0.86 - 0.75 (m, 3H), 0.63 - 0.27 (m, 4H)

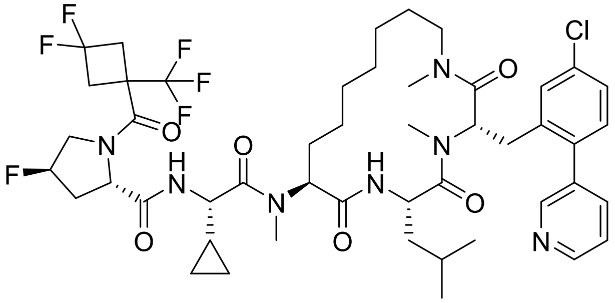

(2S,4R)-N-((S)-2-(((3S,6S,9S)-3-(5-chloro-2-(pyridin-3-yl)benzyl)-6-isobutyl-1,4-dimethyl-2,5,8-trioxo-1,4,7-triazacyclohexadecan-9-yl)(methyl)amino)-1-cyclopropyl-2-oxoethyl)-1-(3,3-difluoro-1-(trifluoromethyl)cyclobutane-1-carbonyl)-4-fluoropyrrolidine-2-carboxamide (Compound 27) was synthesized using method D using **SI-32** and **SI-8** as starting

materials. **Compound 27** was purified using RP-HPLC. **Compound 27** (36.17 mg, 0.037 mmol, 45.80% Yield) was isolated as a fluffy white solid after lyophilization.

LCMS: MS (ESI) mass calcd. for $C_{48}H_{63}ClF_6N_7O_6$: 982.4433 m/z; Found 982.4423 $[M+H]^+$.

^{1}H NMR: (400 MHz, D_3COD) δ 8.72 - 8.55 (m, 2H), 8.41 - 8.25 (m, 1H), 8.00 - 7.91 (m, 1H), 7.61 - 7.50 (m, 1H), 7.44 - 7.29 (m, 2H), 7.27 - 7.18 (m, 1H), 5.43 - 5.21 (m, 1H), 4.77 - 4.65 (m, 1H), 4.70 - 4.45 (m, 1H), 4.37 (br s, 1H), 4.03 - 3.84 (m, 1H), 3.81 - 3.60 (m, 1H), 3.54 - 3.42 (m, 1H), 3.26 - 3.07 (m, 7H), 2.92 - 2.73 (m, 6H), 2.64 - 2.48 (m, 2H), 2.27 - 1.90 (m, 2H), 1.77 - 1.60 (m, 2H), 1.56 (br s, 1H), 1.04 - 0.87 (m, 4H), 0.84 - 0.73 (m, 3H), 0.71 - 0.28 (m, 4H)

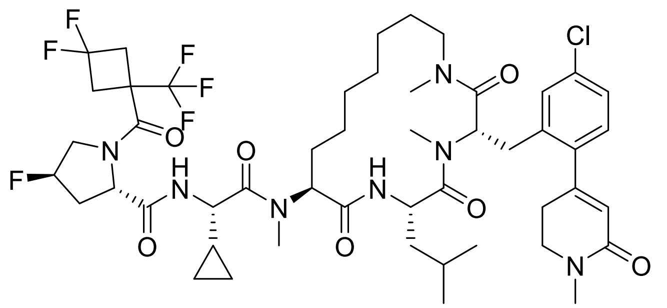

(2S,4R)-N-((S)-2-(((3S,6S,9S)-3-(5-chloro-2-(1-methyl-6-oxo-1,2,3,6-tetrahydropyridin-4-yl)benzyl)-6-isobutyl-1,4-dimethyl-2,5,8-trioxo-1,4,7-triazacyclohexadecan-9-yl)(methyl)amino)-1-cyclopropyl-2-oxoethyl)-1-(3,3-difluoro-1-(trifluoromethyl)cyclobutane-1-carbonyl)-4-fluoropyrrolidine-2-carboxamide (Compound 28) was synthesized using method D using **SI-34** and **SI-8** as starting materials. **Compound 28** was purified using RP-HPLC. **Compound 28** (57.61 mg, 0.057 mmol, 57.60% Yield) was isolated as a fluffy white solid after lyophilization.

LCMS: MS (ESI) mass calcd. for $C_{49}H_{67}ClF_6N_7O_7$: 1014.4695 m/z; Found 1014.4672 $[M+H]^+$.

^{1}H NMR: (400 MHz, D_3COD) δ 8.45 - 8.31 (m, 1H), 7.40 - 7.27 (m, 2H), 7.23 - 7.13 (m, 1H), 5.98 - 5.88 (m, 1H), 5.44 - 5.19 (m, 1H), 4.95 - 4.90 (m, 1H), 4.78 - 4.69 (m, 1H), 4.63 - 4.53 (m, 2H), 4.43 - 4.24 (m, 2H), 4.02 - 3.83 (m, 1H), 3.79 - 3.59 (m, 3H), 3.53 - 3.43 (m, 1H), 3.29 - 3.14 (m, 5H), 3.03 (s, 6H), 3.01 - 2.89 (m, 6H), 2.86 - 2.77 (m, 3H), 2.64 - 2.49 (m, 1H), 2.20 - 2.01 (m, 2H), 1.72 - 1.15 (m, 15H), 1.05 - 0.88 (m, 4H), 0.85 - 0.74 (m, 3H), 0.68 - 0.31 (m, 4H)

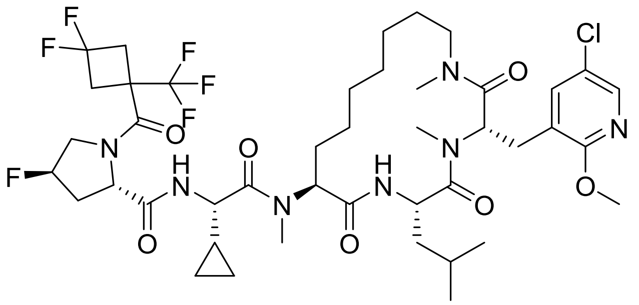

(2S,4R)-N-((S)-2-(((3S,6S,9S)-3-((5-chloro-2-methoxypyridin-3-yl)methyl)-6-isobutyl-1,4-dimethyl-2,5,8-trioxo-1,4,7-triazacyclohexadecan-9-yl)(methyl)amino)-1-cyclopropyl-2-oxoethyl)-1-(3,3-difluoro-1-(trifluoromethyl)cyclobutane-1-carbonyl)-4-fluoropyrrolidine-2-carboxamide (Compound 29) was synthesized using method D using **SI-25** and **SI-8** as starting materials. **Compound 29** was purified using RP-HPLC. **Compound 29** (59.95 mg, 0.064 mmol, 31.40% Yield) was isolated as a fluffy white solid after lyophilization.

LCMS: MS (ESI) mass calcd. for $C_{43}H_{61}ClF_6N_7O_7$: 936.4225 m/z; Found 936.4215 $[M+H]^+$.

^{1}H NMR: (400 MHz, D_3COD) δ 8.03 (d, J = 2.5 Hz, 1H), 7.56 - 7.45 (m, 1H), 5.41 - 5.24 (m, 1H), 4.99 - 4.94 (m, 1H), 4.73 (br t, J = 8.7 Hz, 1H), 4.65 - 4.53 (m, 3H), 4.45 - 4.28 (m, 2H), 4.07 - 3.86 (m, 4H), 3.43 (br d, J = 4.9 Hz, 3H), 3.23 - 3.09 (m, 7H), 3.05 - 2.80 (m, 7H), 2.65 - 2.46 (m, 1H), 2.22 - 1.95 (m, 2H), 1.79 - 1.54 (m, 3H), 1.47 - 1.29 (m, 9H), 1.26 - 1.12 (m, 3H), 0.98 - 0.75 (m, 7H), 0.65 - 0.43 (m, 3H), 0.37 - 0.28 (m, 1H)

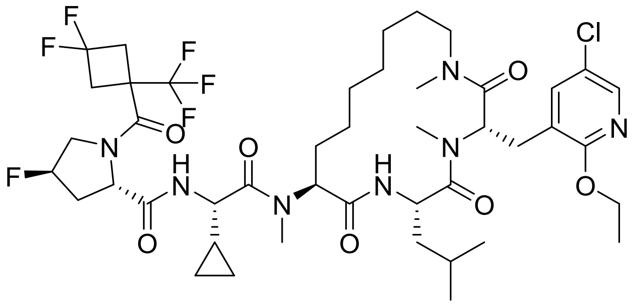

(2S,4R)-N-((S)-2-(((3S,6S,9S)-3-((5-chloro-2-ethoxypyridin-3-yl)methyl)-6-isobutyl-1,4-dimethyl-2,5,8-trioxo-1,4,7-triazacyclohexadecan-9-yl)(methyl)amino)-1-cyclopropyl-2-oxoethyl)-1-(3,3-difluoro-1-(trifluoromethyl)cyclobutane-1-carbonyl)-4-fluoropyrrolidine-

2-carboxamide (Compound 30) was synthesized using method D using **SI-27** and **SI-8** as starting materials. **Compound 30** was purified using RP-HPLC. **Compound 30** (61.87 mg, 0.065 mmol, 33.10% Yield) was isolated as a fluffy white solid after lyophilization.

LCMS: MS (ESI) mass calcd. for $C_{44}H_{63}ClF_6N_7O_7$: 950.4382 m/z; Found 950.4404 $[M+H]^+$.

^{1}H NMR: (400 MHz, D_3COD) δ 8.20 - 7.95 (m, 1H), 7.65 - 7.42 (m, 1H), 5.43 - 5.21 (m, 1H), 4.96 - 4.93 (m, 1H), 4.73 (br t, J = 8.6 Hz, 1H), 4.64 - 4.54 (m, 3H), 4.47 - 4.27 (m, 4H), 4.00 - 3.83 (m, 1H), 3.78 - 3.59 (m, 1H), 3.57 - 3.41 (m, 2H), 3.21 (br d, J = 3.9 Hz, 7H), 3.02 - 2.81 (m, 7H), 2.65 - 2.50 (m, 1H), 2.24 - 1.98 (m, 2H), 1.74 - 1.54 (m, 3H), 1.52 - 1.42 (m, 7H), 1.40 - 1.28 (m, 5H), 1.25 - 1.16 (m, 2H), 1.01 - 0.78 (m, 7H), 0.61 - 0.28 (m, 4H)

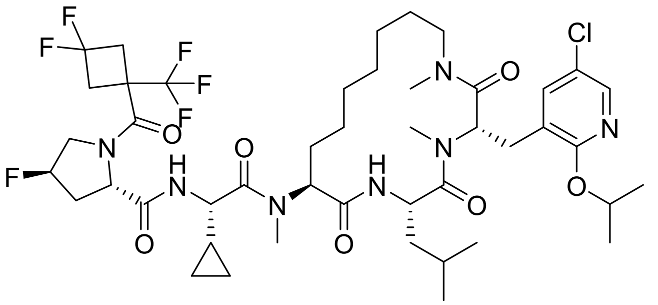

(2S,4R)-N-((S)-2-(((3S,6S,9S)-3-((5-chloro-2-isopropoxypyridin-3-yl)methyl)-6-isobutyl-1,4-dimethyl-2,5,8-trioxo-1,4,7-triazacyclohexadecan-9-yl)(methyl)amino)-1-cyclopropyl-2-oxoethyl)-1-(3,3-difluoro-1-(trifluoromethyl)cyclobutane-1-carbonyl)-4-fluoropyrrolidine-2-carboxamide (Compound 31) was synthesized using method D using **SI-26** and **SI-8** as starting materials. **Compound 31** was purified using RP-HPLC. **Compound 31** (34.54 mg, 0.035 mmol, 27.3% Yield) was isolated as a fluffy white solid after lyophilization.

LCMS: MS (ESI) mass calcd. for $C_{45}H_{65}ClF_6N_7O_7$: 964.4538 m/z; Found 964.4506 $[M+H]^+$.

^{1}H NMR: (400 MHz, D_3COD) δ 8.45 - 8.29 (m, 1H), 8.11 - 7.91 (m, 1H), 7.64 - 7.32 (m, 1H), 5.45 - 5.21 (m, 2H), 4.95 - 4.90 (m, 1H), 4.76 - 4.70 (m, 1H), 4.66 - 4.53 (m, 2H), 4.45 - 4.29 (m, 2H), 4.01 - 3.84 (m, 1H), 3.80 - 3.39 (m, 2H), 3.02 (br d, J = 6.8 Hz, 8H), 2.99 - 2.80 (m, 6H), 2.78 (s, 1H), 2.63 - 2.51 (m, 1H), 2.21 - 1.98 (m, 2H), 1.74 - 1.53 (m, 3H), 1.49 - 1.14 (m, 17H), 1.03 - 0.87 (m, 4H), 0.84 - 0.77 (m, 3H), 0.67 - 0.29 (m, 4H)

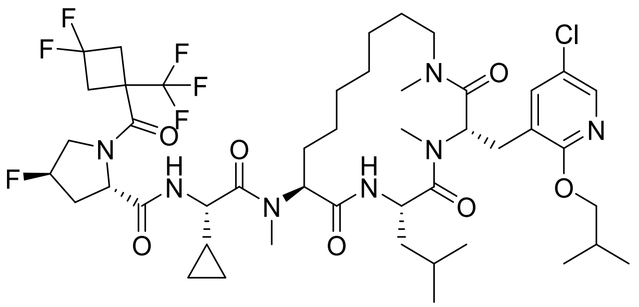

(2S,4R)-N-((S)-2-(((3S,6S,9S)-3-((5-chloro-2-isobutoxypyridin-3-yl)methyl)-6-isobutyl-1,4-dimethyl-2,5,8-trioxo-1,4,7-triazacyclohexadecan-9-yl)(methyl)amino)-1-cyclopropyl-2-oxoethyl)-1-(3,3-difluoro-1-(trifluoromethyl)cyclobutane-1-carbonyl)-4-fluoropyrrolidine-2-carboxamide (Compound 32) was synthesized using method D using **SI-28** and **SI-8** as starting materials. **Compound 32** was purified using RP-HPLC. **Compound 32** (55.65 mg, 0.057 mmol, 29.10% Yield) was isolated as a fluffy white solid after lyophilization.

LCMS: MS (ESI) mass calcd. for $C_{46}H_{67}ClF_6N_7O_7$: 978.4695 m/z; Found 978.4709 $[M+H]^+$.

^{1}H NMR: (400 MHz, D_3COD) δ 8.13 - 7.93 (m, 1H), 7.59 - 7.44 (m, 1H), 5.44 - 5.23 (m, 1H), 4.94 - 4.90 (m, 1H), 4.77 - 4.69 (m, 1H), 4.66 - 4.54 (m, 2H), 4.40 - 4.26 (m, 2H), 4.23 - 4.07 (m, 2H), 3.98 - 3.84 (m, 1H), 3.79 - 3.59 (m, 1H), 3.56 - 3.40 (m, 1H), 3.24 - 3.07 (m, 7H), 3.03 (s, 7H), 2.64 (br s, 1H), 2.25 - 2.01 (m, 3H), 1.72 - 1.53 (m, 3H), 1.50 - 1.16 (m, 12H), 1.12 - 1.03 (m, 6H), 1.00 - 0.87 (m, 4H), 0.85 - 0.72 (m, 3H), 0.63 - 0.28 (m, 4H)

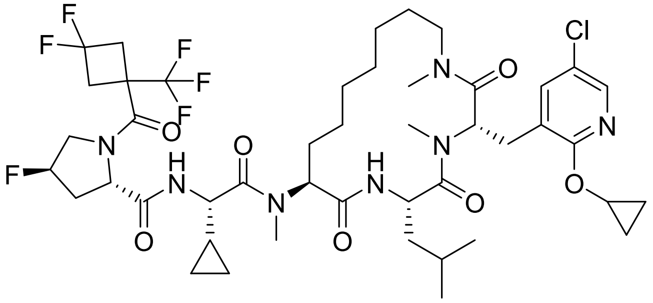

(2S,4R)-N-((S)-2-(((3S,6S,9S)-3-((5-chloro-2-cyclopropoxypyridin-3-yl)methyl)-6-isobutyl-1,4-dimethyl-2,5,8-trioxo-1,4,7-triazacyclohexadecan-9-yl)(methyl)amino)-1-cyclopropyl-2-oxoethyl)-1-(3,3-difluoro-1-(trifluoromethyl)cyclobutane-1-carbonyl)-4-fluoropyrrolidine-2-carboxamide (Compound 33) was synthesized using method D using **SI-29** and **SI-8** as starting

materials. **Compound 33** was purified using RP-HPLC. **Compound 33** (24.3 g, 25.3 mmol, 37% yield) was isolated as a fluffy white solid after lyophilization.

LCMS: MS (ESI) mass calcd. for $C_{45}H_{63}ClF_6N_7O_7$: 962.4382 m/z; Found 962.4399 $[M+H]^+$.

^{1}H NMR: (400 MHz, D_3COD) δ 8.09 (d, J=2.4 Hz, 0.3H, active hydrogen that has not been completely exchanged), 7.79 (d, J=3.2 Hz, 1H), 7.30 (d, J=3.6 Hz, 1H), 5.08 (d, J=23.6 Hz, 1H), 4.67 (s, 1H), 4.37-4.60 (m, 2H), 4.35 - 4.28 (m, 1H), 4.26 - 4.01 (m, 3H), 3.78 - 3.59 (m, 1H), 3.57 - 3.35 (m, 1H), 3.32 - 3.12 (m, 1H), 3.06 - 2.85 (m, 6H), 2.85 - 2.78 (m, 1H), 2.77 - 2.53 (m, 7H), 2.50 - 2.23 (m, 2H), 2.09 - 1.70 (m, 2H), 1.49 - 1.27 (m, 3H), 1.27 - 0.86 (m, 11H), 0.48 (br s, 11H), 0.43 - 0.04 (m, 4H).

^{13}C NMR: (101 MHz, D_3COD) δ 172.088, 172.046, 171.913, 171.769, 171.583, 171.447, 171.143, 171.01, 169.671, 169.413, 169.352, 165.622, 165.413, 161.365, 143.061, 139.729, 139.274, 127.28, 127.242, 124.491, 124.457, 123.884, 122.317, 121.71, 118.947, 116.223, 113.499, 93.138, 93.02, 91.362, 91.241, 59.573, 59.079, 56.473, 56.203, 55.911, 54.67, 54.454, 54.253, 52.891, 52.174, 50.99, 50.371, 48.322, 47.894, 47.681, 47.469, 47.044, 42.031, 41.933, 41.891, 41.796, 41.743, 41.644, 41.602, 41.504, 41.314, 41.064, 40.809, 39.607, 39.345, 39.083, 38.863, 35.072, 34.981, 34.852, 34.765, 34.533, 34.446, 30.121, 29.373, 28.576, 27.863, 26.22, 25.734, 25.275, 24.471, 23.64, 23.439, 23.131, 22.577, 22.467, 22.278, 22.266, 22.133, 20.631, 20.547, 19.88, 12.83, 12.082, 11.813, 5.074, 4.918, 2.812, 2.228, 2.152, 1.962, 1.386

Table S4: HPLC purity of Solution Phase Compounds

Compound ID	Analytical Method	Rt (min)	% Purity	m/z observed	Calcd m/z
19	B	0.883	99	927.45	927.39
20	B	0.806	99	923.38	923.34
21	B	0.897	99	941.43	941.4
22	B	0.873	99	937.37	937.35
23	B	0.841	99	925.46	925.35
24	B	0.816	99	939.5	939.37
25	B	0.779	99	1028.55	1028.48
26	B	0.760	99	1002.47	1002.41
27	C	2.446	96	982.42	982.44

28	C	2.897	96	1014.47	1014.46
29	C	3.189	99	936.46	936.41
30	C	3.434	96	950.47	950.43
31	C	3.673	95	964.44	964.45
32	C	3.837	99	978.51	978.46
33	C	3.367	95	962.36	962.43

Biochemical Probe Synthesis

Synthesis of fluorescence polarization probe ("FP-2") performed as previously described.[1]

Synthesis of FP2 Probe:

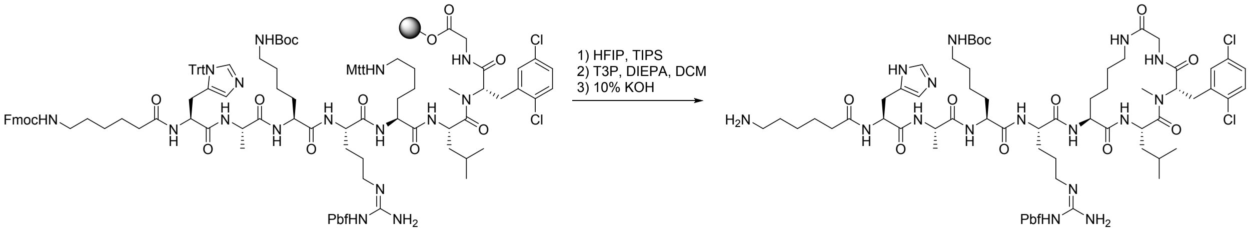

tert-butyl ((S)-6-(((S)-5-(((Z)-amino((2,2,4,6,7-pentamethyl-2,3-dihydrobenzofuran)-5-sulfonamido)methylene)amino)-1-(((6S,9S,12S)-6-(2,5-dichlorobenzyl)-9-isobutyl-7-methyl-2,5,8,11-tetraoxo-1,4,7,10-tetraazacyclohexadecan-12-yl)amino)-1-oxopentan-2-yl)amino)-5-((S)-2-((S)-2-(6-aminohexanamido)-3-(1H-imidazol-5-yl)propanamido)propanamido)-6-oxohexyl)carbamate: The FP2 Probe Linear Intermediate in **Table 2b** with Fmoc-Ahx still intact was cleaved from the solid phase with a 2 mL solution of 24% HFIP, 2% TIPS, in DCM. The material was concentrated under reduced pressure and purified via reverse-phase chromatography to yield a white powder. **LCMS:** MS (ESI) mass calcd. For cleaved linear intermediate $C_{103}H_{132}Cl_2N_{16}O_{17}S$ 1966.91 m/z found 1968.65 $[M+H]^+$.

The deprotected and purified linear product was transferred to a 50 mL conical vial and dissolved in 1 mL NMP followed by the addition of DIEA (0.5 mL) and DCM (35 mL). T3P (3 eqv) was added to the solution and the reaction pH was adjusted to pH 9 via dropwise addition of DIPEA. The closed conical vial was agitated at room temperature for 2 hours at 150 rotations per minute. The solution was concentrated at 45°C under reduced pressure in a Genevac system. The Fmoc group was then removed with the addition of a 10% of KOH/Water solution (5 mL) heated at 70°C

for 30 minutes. The resulting LCMS trace revealed that the trityl group had been unexpectedly removed during the cyclization and Fmoc-deprotection steps. The cyclic peptide was then purified via reverse phase HPLC using an Acetonitrile/Water gradient with 0.05% formic acid. The purified fractions were pooled and lyophilized. **LCMS:** MS (ESI) mass calcd. For $C_{69}H_{106}Cl_2N_{16}O_{14}S$ 1484.72 m/z found 1485.94 $[M+H]^+$.

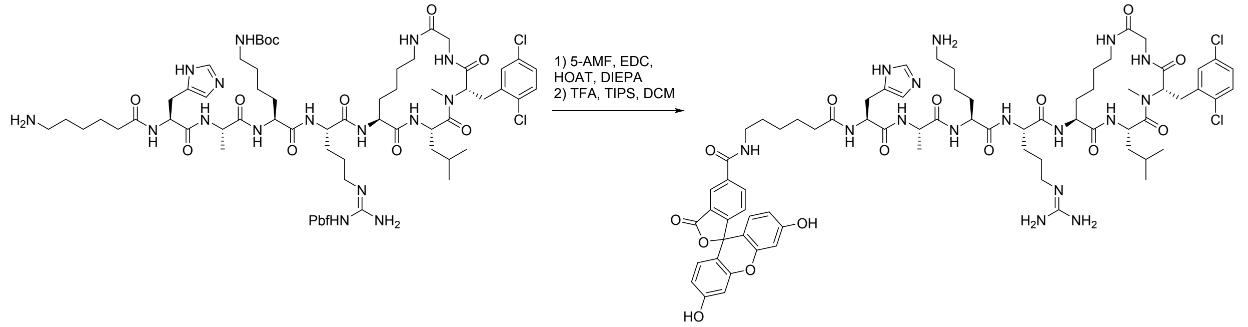

N-((6S,9S,12S,15S)-15-((1H-imidazol-5-yl)methyl)-1,1-diamino-9-(4-aminobutyl)-6-(((6S,9S,12S)-6-(2,5-dichlorobenzyl)-9-isobutyl-7-methyl-2,5,8,11-tetraoxo-1,4,7,10-tetraazacyclohexadecan-12-yl)carbamoyl)-12-methyl-8,11,14,17-tetraoxo-2,7,10,13,16-pentaazadocos-1-en-22-yl)-3',6'-dihydroxy-3-oxo-3H-spiro[isobenzofuran-1,9'-xanthene]-5-carboxamide (FP2 Probe): The probe was fluorescently labeled via a peptide coupling in solution. A solution of 5-carboxyfluorescein (4 Equiv.), EDC (4 Equiv.), HOAt (3.9 Equiv.) and DIPEA (8 Equiv.) in 1.0 mL of anhydrous DCM was prepared. The mixture was allowed to pre-activate at room temperature for 5 minutes. The macrocycle with free N-terminal amine was added to the coupling solution, and the reaction was agitated at room temperature until starting material was not observed by LCMS.

LCMS: MS (ESI) mass calcd. For $C_{90}H_{116}Cl_2N_{16}O_{20}S$ 1842.76 m/z found 1844.29 $[M+H]^+$.
The Boc and Pbf protecting groups were removed by dissolving the cyclic peptide in a 1 mL solution of 90% TFA, 5% TIPS, 5% DCM and agitating for 1 hour. The reaction was monitored by LCMS for the disappearance of starting material. Upon completion, the reaction was concentrated. The crude material was co-evaporated with DCE (5 mL x 2), and then purified via reverse phase-HPLC to yield **FP2 Probe** (0.7 mg, >99% purity by UV). **LCMS:** MS (ESI) mass calcd. For $C_{72}H_{92}Cl_2N_{16}O_{15}$ 1490.63 m/z found 1493.4 $[M+H]^+$.

Spectra For the Synthesis of Compound 23

Tert-butyl (S)-(3-(2,5-dichlorophenyl)-1-(hex-5-en-1-ylamino)-1-oxopropan-2-yl)(methyl)carbamate (SI-10):

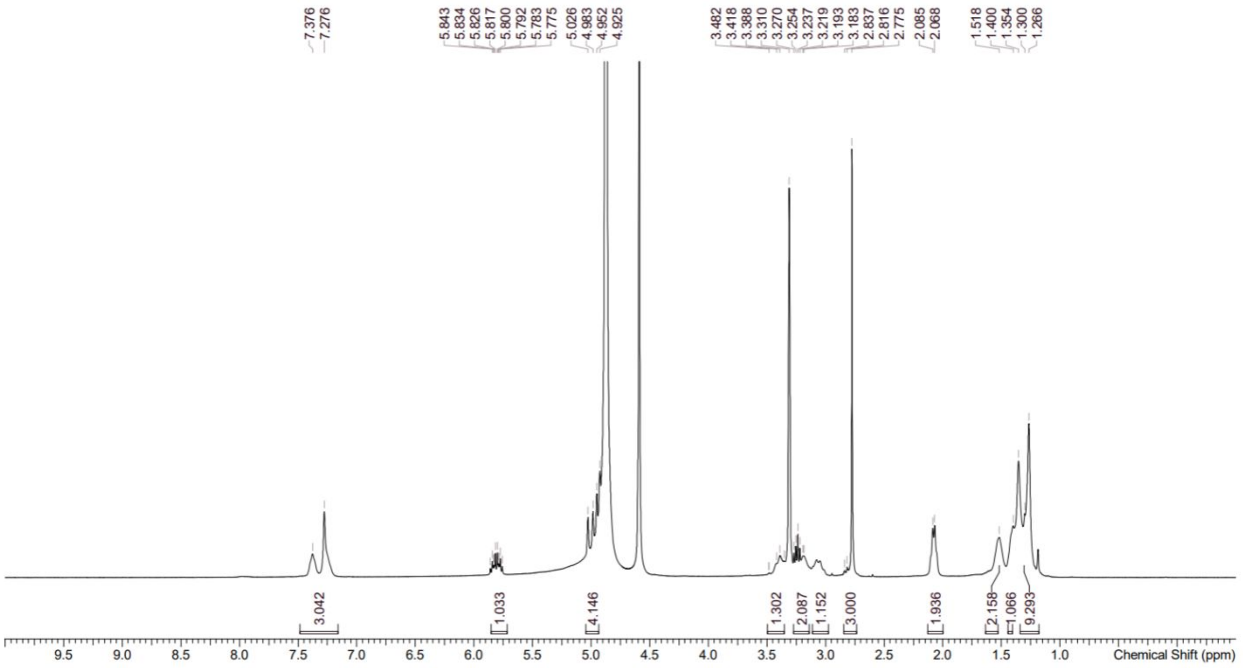

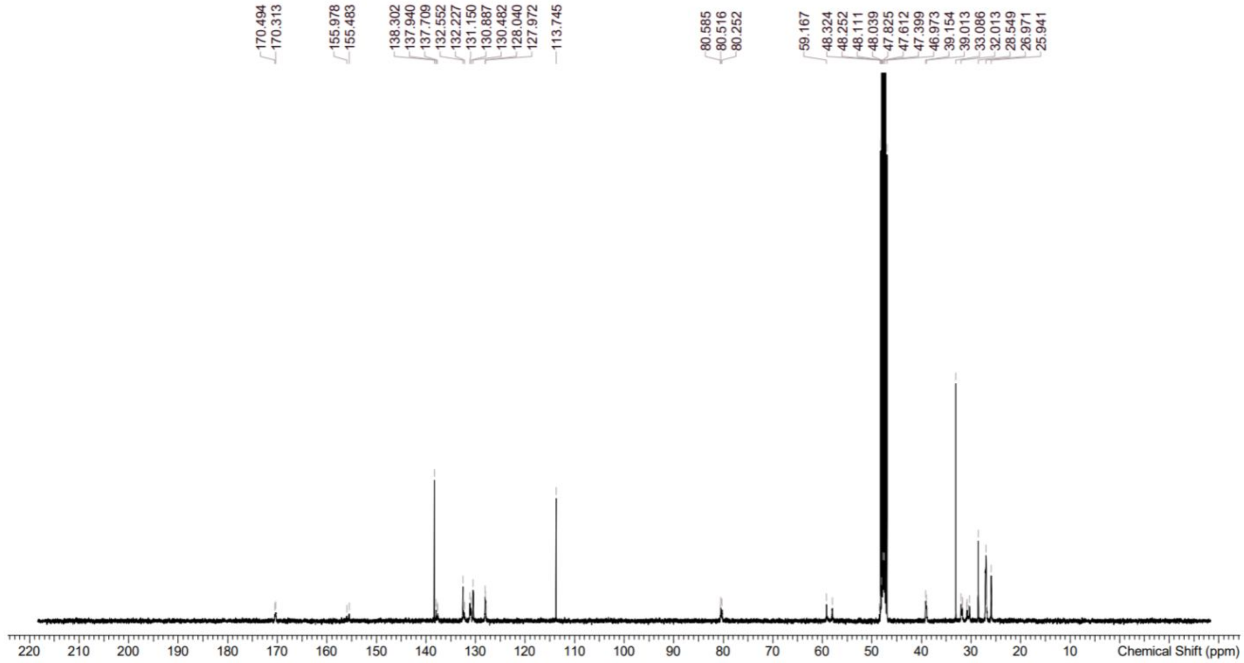

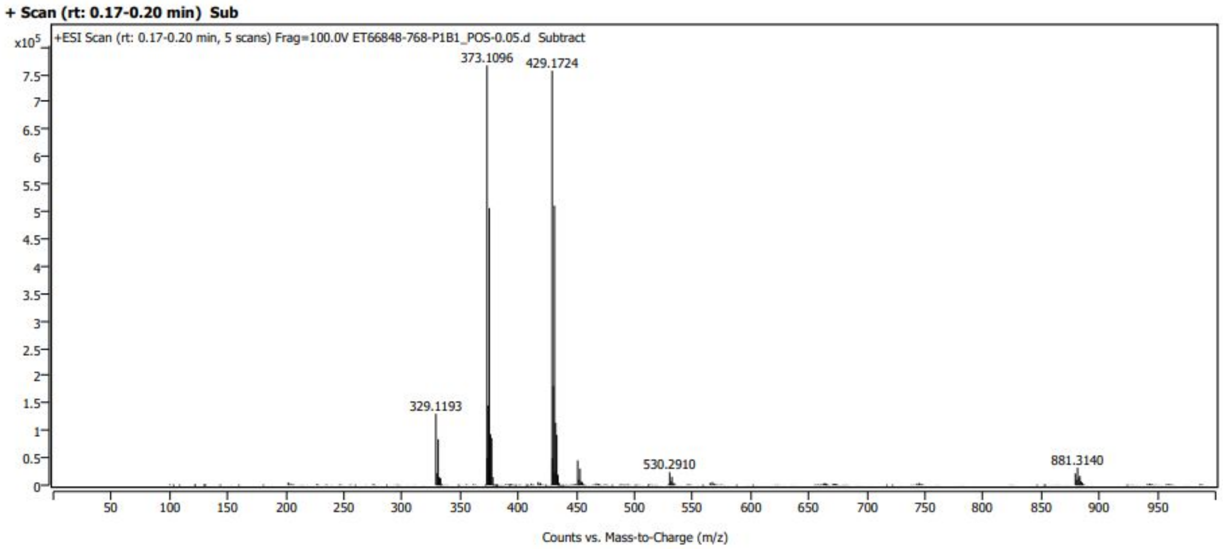

Tert-butyl ((S)-1-(((S)-3-(2,5-dichlorophenyl)-1-(hex-5-en-1-ylamino)-1-oxopropan-2-yl)(methyl)amino)-4-methyl-1-oxopentan-2-yl)carbamate (SI-11):

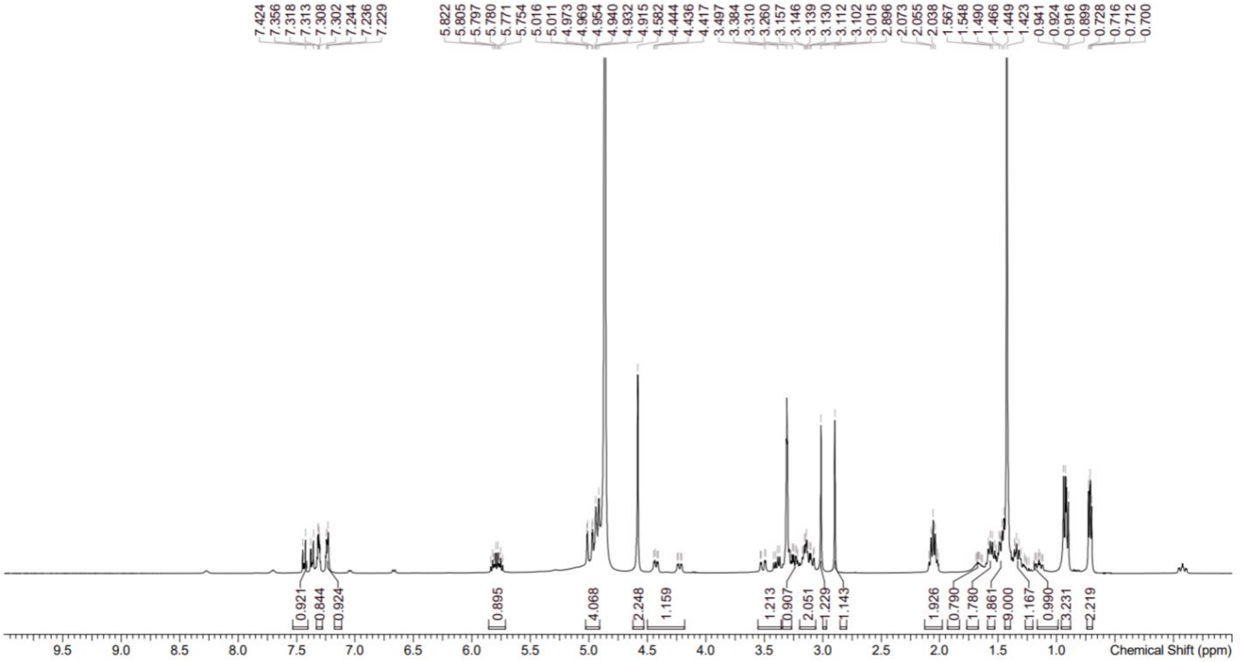

7.424
7.356
7.318
7.313
7.308
7.302
7.244
7.236
7.229
5.822
5.805
5.797
5.780
5.771
5.754
5.016
5.011
4.973
4.969
4.954
4.940
4.932
4.915
4.582
4.444
4.436
4.417
3.497
3.384
3.310
3.260
3.157
3.146
3.139
3.130
3.112
3.102
3.015
2.896
2.073
2.055
2.038
1.567
1.548
1.490
1.466
1.449
1.423
0.941
0.924
0.916
0.899
0.728
0.716
0.712
0.700
0.921
0.844
0.924
0.895
4.068
2.248
1.159
1.213
0.907
2.051
1.229
1.143
1.926
0.790
1.780
1.861
9.000
1.167
0.990
3.231
2.219
9.5
9.0
8.5
8.0
7.5
7.0
6.5
6.0
5.5
5.0
4.5
4.0
3.5
3.0
2.5
2.0
1.5
1.0
Chemical Shift (ppm)

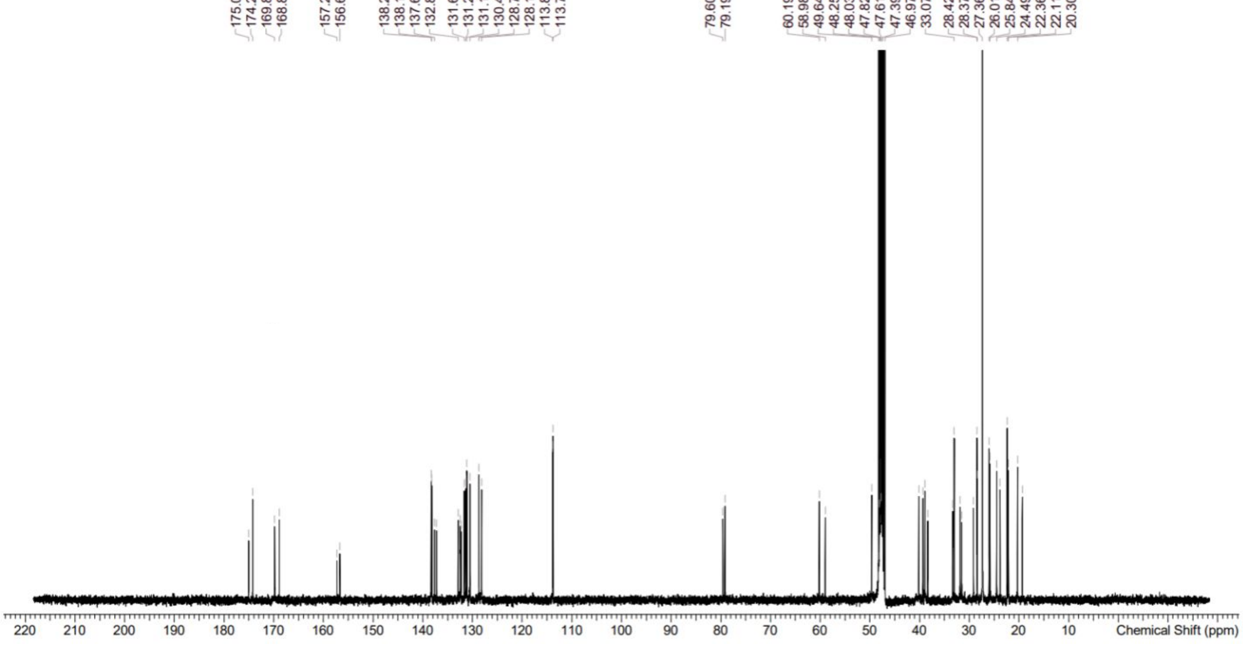

175.037
174.214
169.800
168.872
157.256
156.682
138.298
138.172
137.630
132.884
131.631
131.295
131.110
130.489
128.709
128.163
113.846
113.759
79.602
79.191
60.192
58.986
49.646
48.252
48.039
47.825
47.612
47.399
46.977
33.075
28.426
28.376
27.365
26.017
25.840
24.493
22.369
22.117
20.303
220
210
200
190
180
170
160
150
140
130
120
110
100
90
80
70
60
50
40
30
20
10
Chemical Shift (ppm)

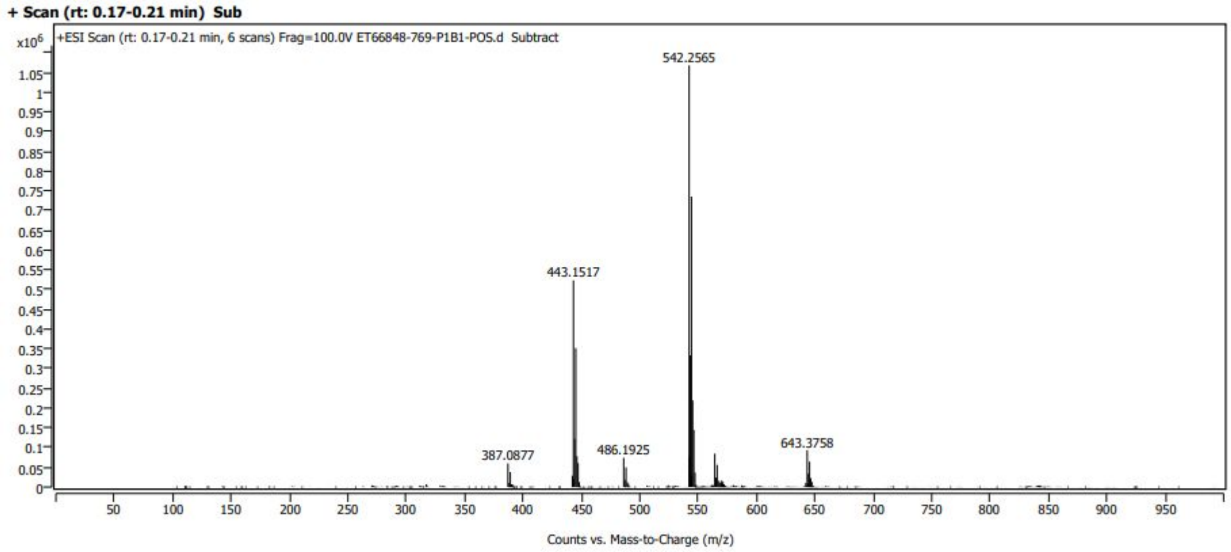

Tert-butyl ((S)-1-(((S)-1-(((S)-3-(2,5-dichlorophenyl)-1-(hex-5-en-1-ylamino)-1-oxopropan-2-yl)(methyl)amino)-4-methyl-1-oxopentan-2-yl)amino)-1-oxopent-4-en-2-yl)(methyl)carbamate (SI-12):

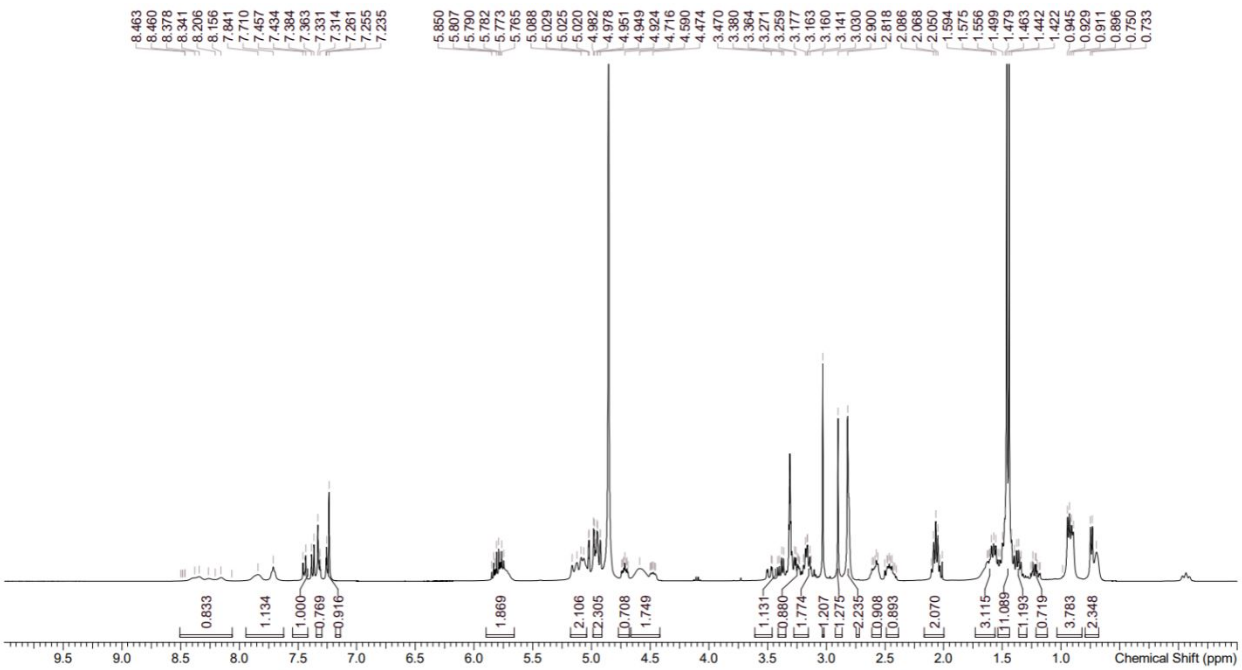

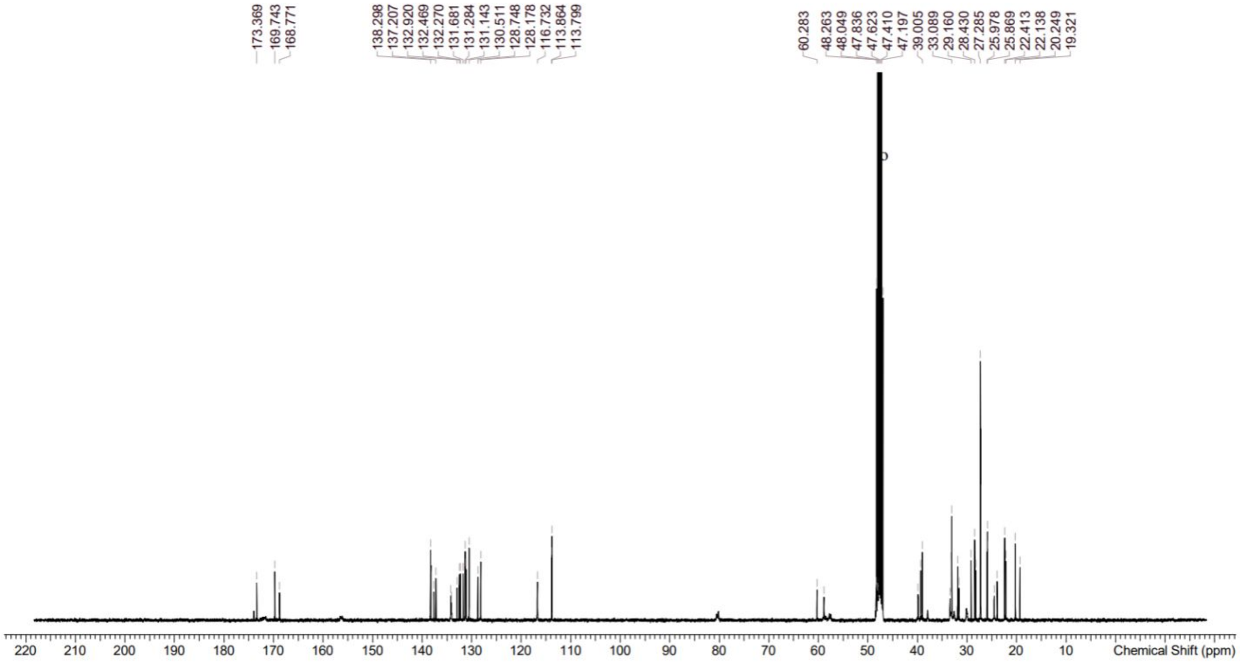

173.369
169.743
168.771
138.298
137.207
132.920
132.469
132.270
131.681
131.284
131.143
130.511
128.748
128.178
116.732
113.864
113.799
60.283
48.263
48.049
47.836
47.623
47.410
47.197
39.005
33.089
29.160
28.430
27.285
25.978
25.869
22.413
22.138
20.249
19.321
220 210 200 190 180 170 160 150 140 130 120 110 100 90 80 70 60 50 40 30 20 10
Chemical Shift (ppm)

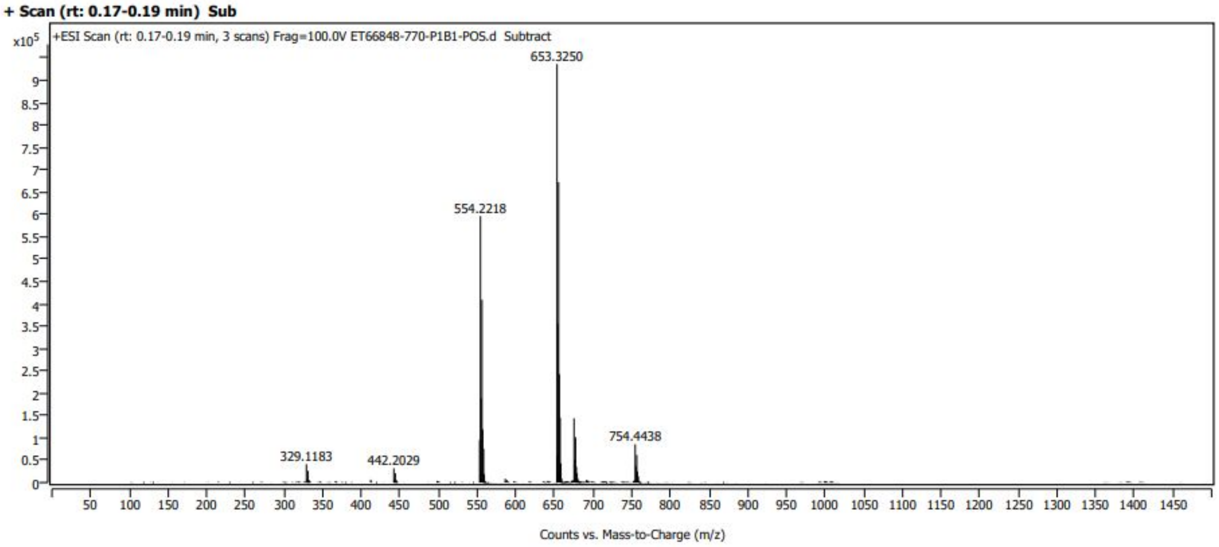

+ Scan (rt: 0.17-0.19 min) Sub
x10^5
+ESI Scan (rt: 0.17-0.19 min, 3 scans) Frag=100.0V ET66848-770-P1B1-POS.d Subtract
653.3250
554.2218
754.4438
329.1183
442.2029
Counts vs. Mass-to-Charge (m/z)

Tert-butyl ((1S,4S,7S,10S)-4-allyl-1-cyclopropyl-10-(2,5-dichlorobenzyl)-7-isobutyl-3,9-dimethyl-2,5,8,11-tetraoxo-3,6,9,12-tetraazaoctadec-17-en-1-yl)carbamate (SI-13):

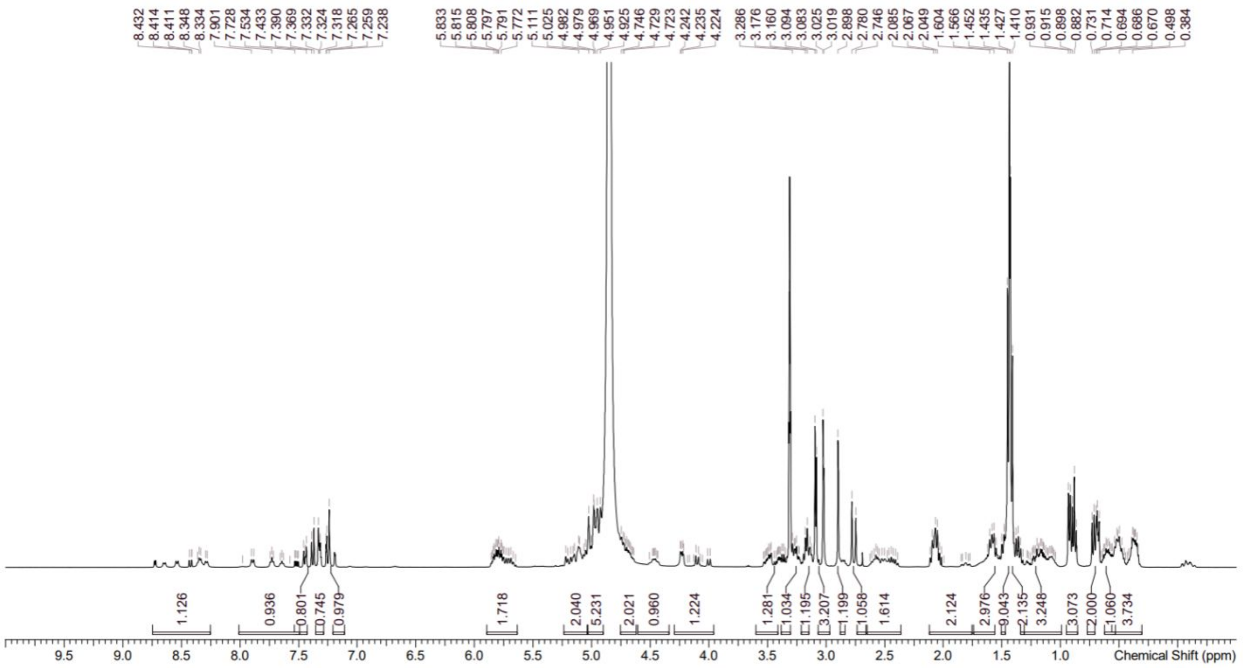

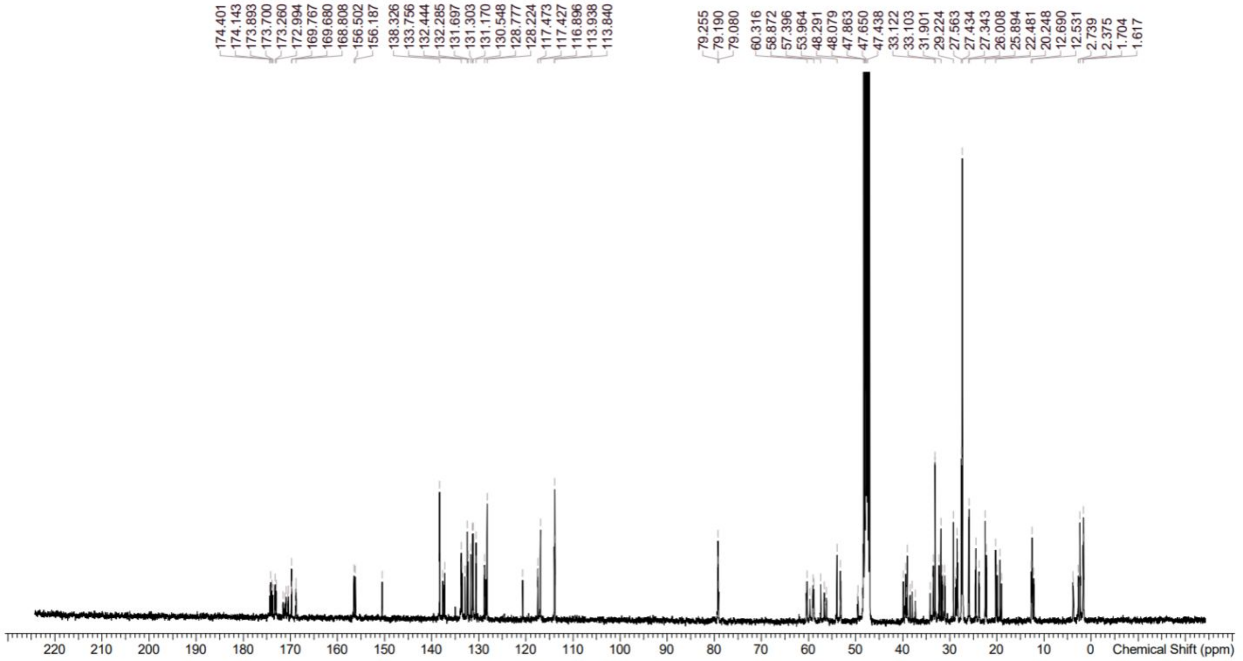

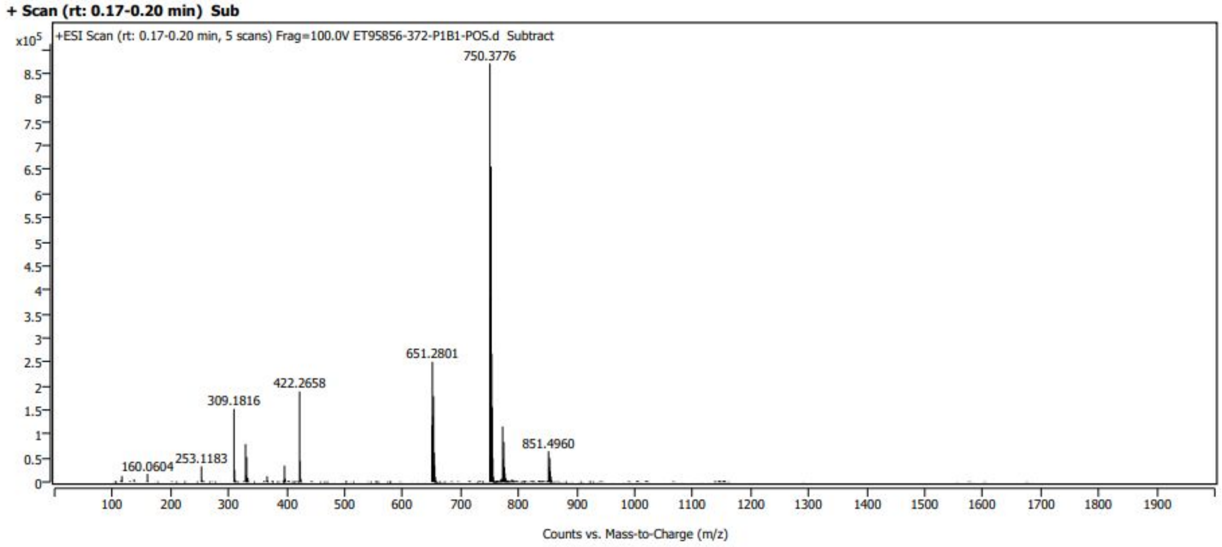

Tert-butyl ((S)-1-cyclopropyl-2-(((3S,6S,9S)-3-(2,5-dichlorobenzyl)-6-isobutyl-4-methyl-2,5,8-trioxo-1,4,7-triazacyclohexadec-11-en-9-yl)(methyl)amino)-2-oxoethyl)carbamate (SI-14):

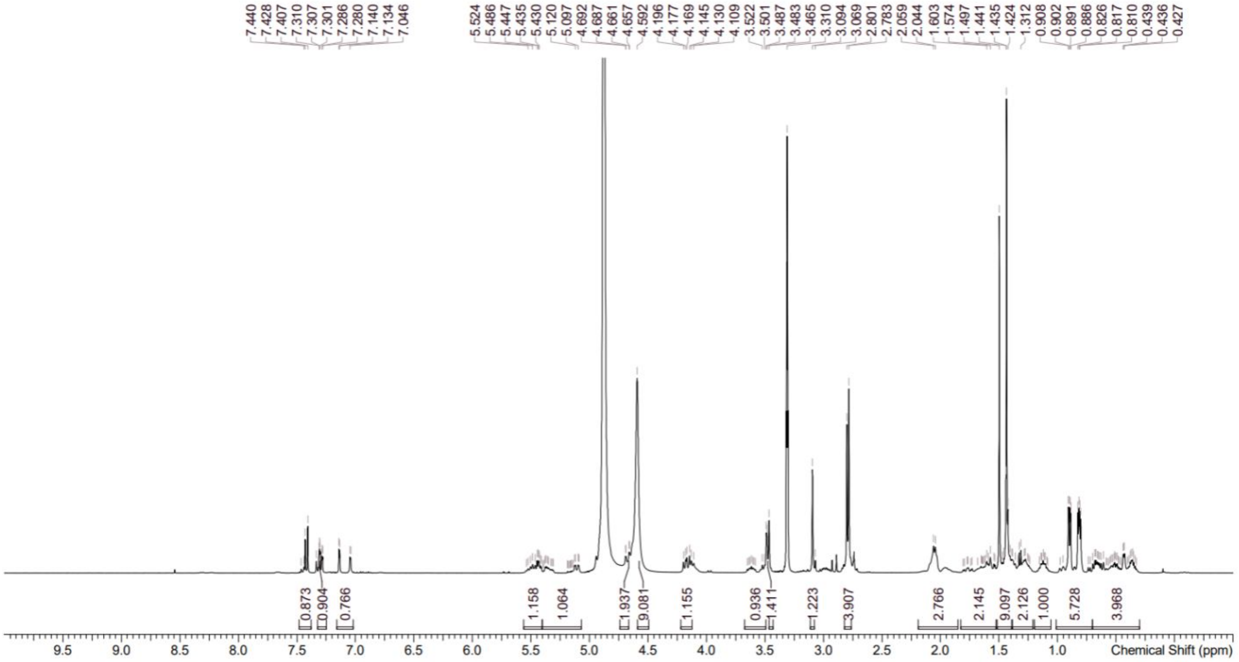

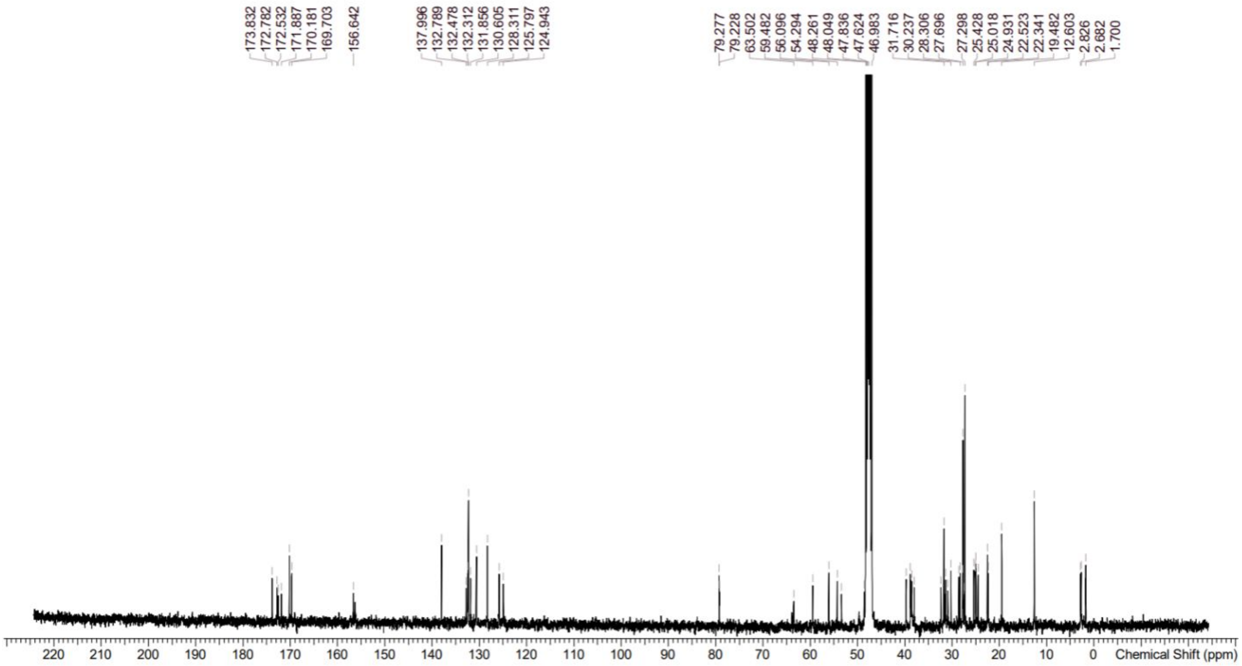
173.832
172.782
172.532
171.887
170.181
169.703
156.642
137.996
132.789
132.478
132.312
131.856
130.605
128.311
125.797
124.943
79.277
79.228
63.502
59.482
56.096
54.294
48.261
48.049
47.836
47.624
46.983
31.716
30.237
28.306
27.696
27.298
25.428
25.018
24.931
22.523
22.341
19.482
12.603
2.826
2.682
1.700
Chemical Shift (ppm)

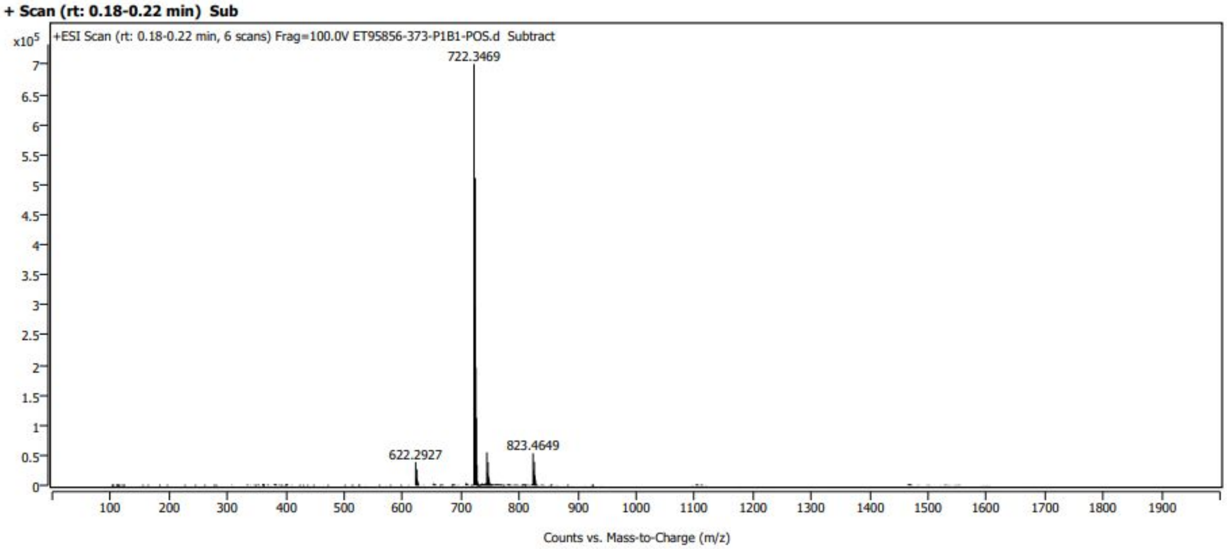
+ Scan (rt: 0.18-0.22 min) Sub
+ESI Scan (rt: 0.18-0.22 min, 6 scans) Frag=100.0V ET95856-373-P1B1-POS.d Subtract
x10^5
722.3469
622.2927
823.4649
Counts vs. Mass-to-Charge (m/z)

Tert-butyl ((S)-1-cyclopropyl-2-(((3S,6S,9S)-3-(2,5-dichlorobenzyl)-6-isobutyl-4-methyl-2,5,8-trioxo-1,4,7-triazacyclohexadecan-9-yl)(methyl)amino)-2-oxoethyl)carbamate (SI-22):

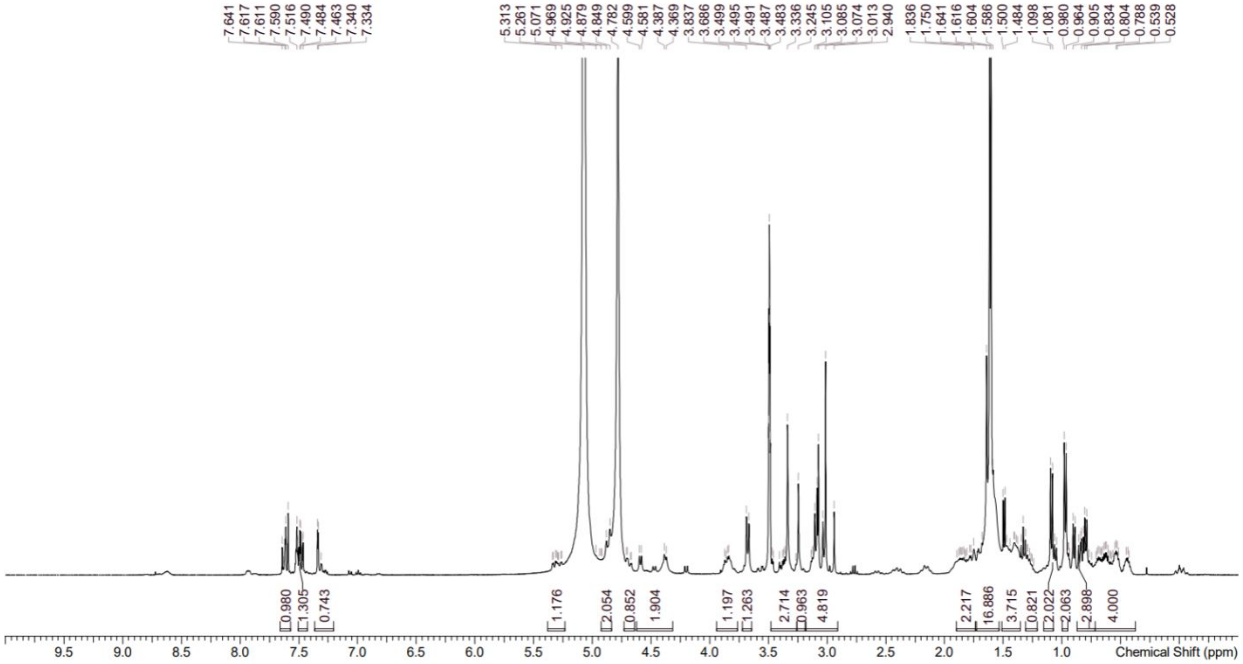

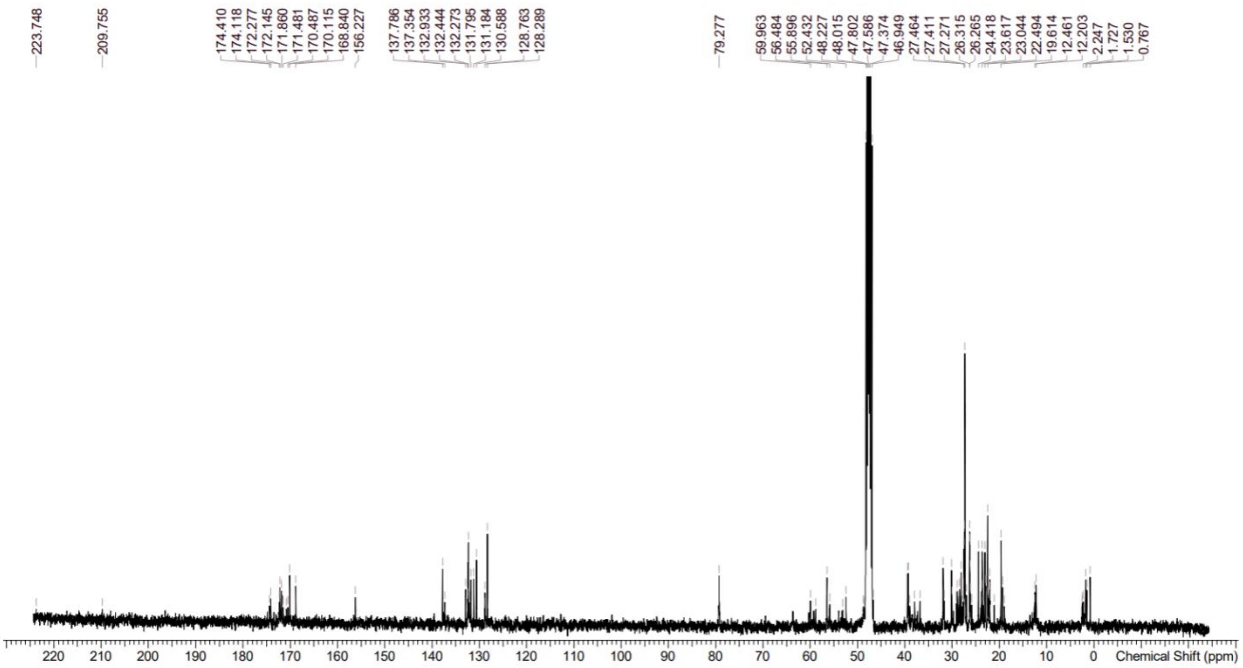

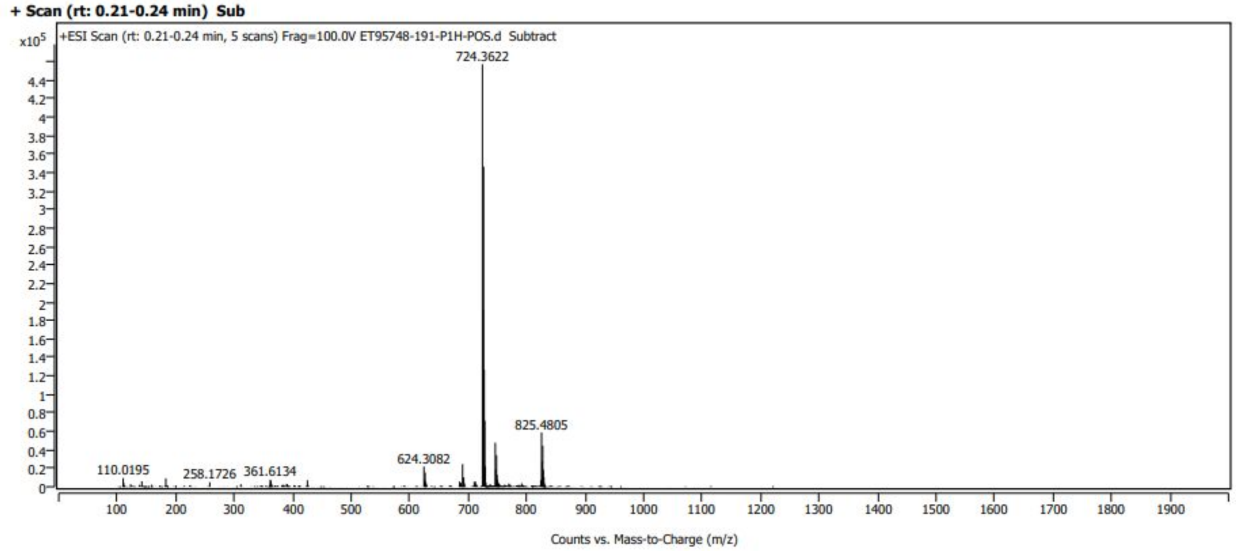

(2S,4R)-N-((S)-1-cyclopropyl-2-(((3S,6S,9S)-3-(2,5-dichlorobenzyl)-6-isobutyl-4-methyl-2,5,8-trioxo-1,4,7-triazacyclohexadecan-9-yl)(methyl)amino)-2-oxoethyl)-1-(3,3-difluoro-1-(trifluoromethyl)cyclobutane-1-carbonyl)-4-fluoropyrrolidine-2-carboxamide (Compound 23):

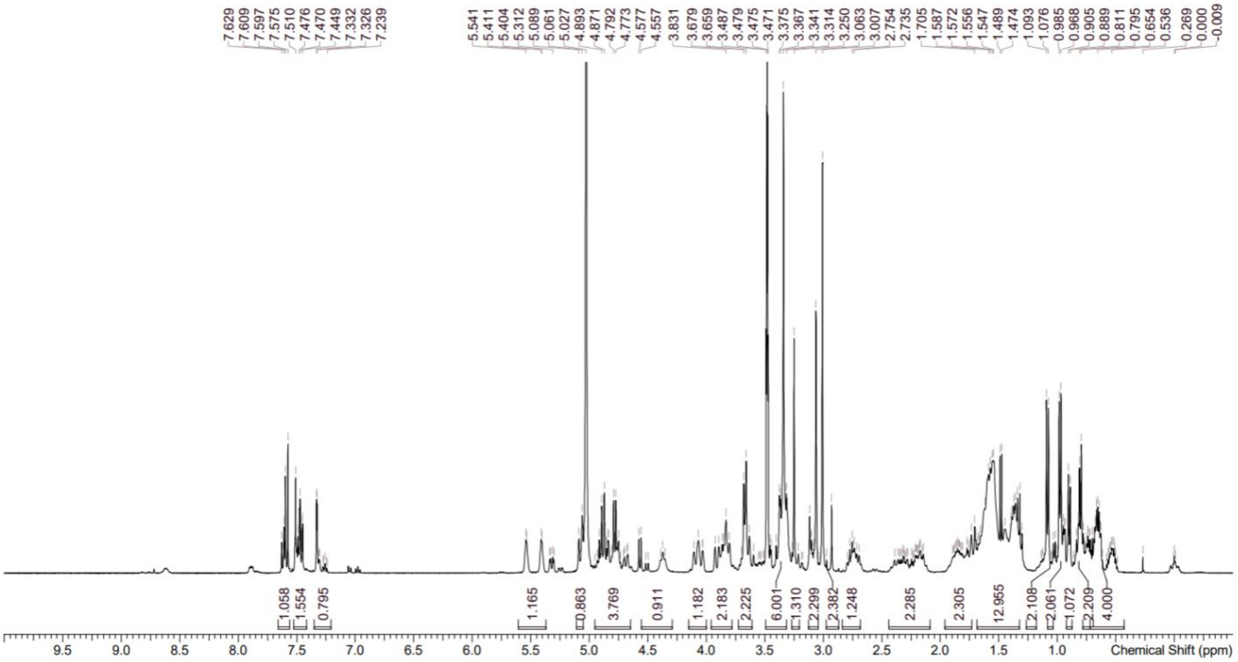

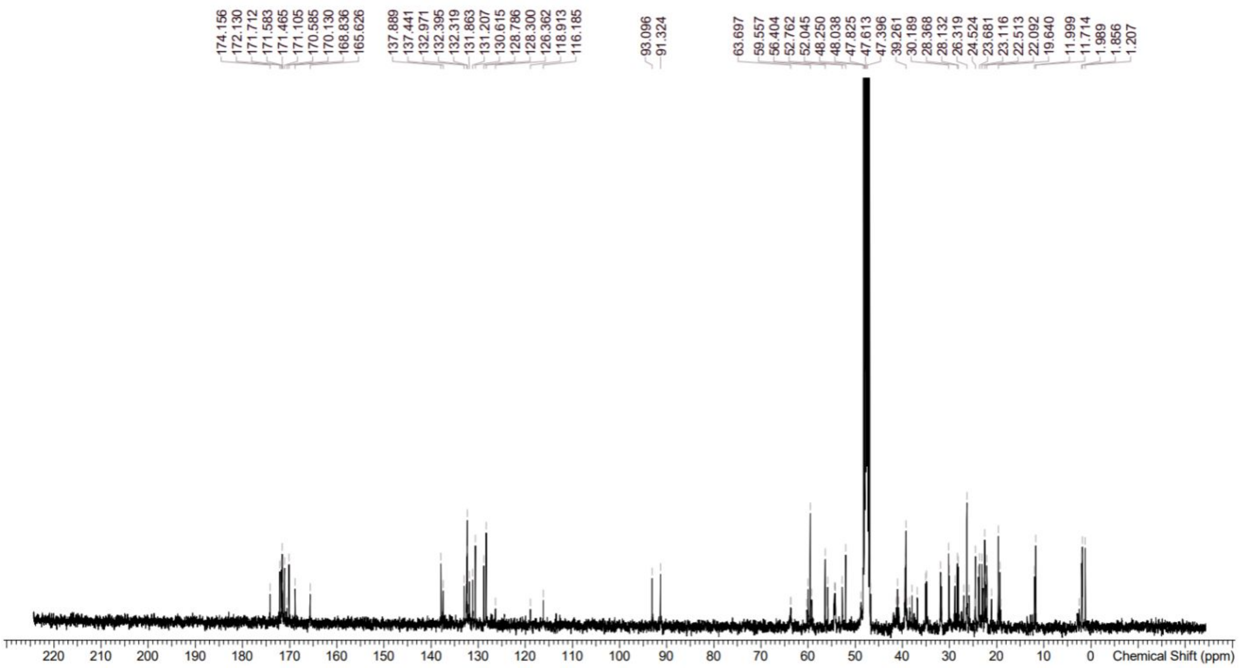
174.156
172.130
171.712
171.583
171.465
171.105
170.585
170.130
168.836
165.626
137.889
137.441
132.971
132.395
132.319
131.863
131.207
130.615
128.786
128.300
126.362
118.913
116.185
93.096
91.324
63.697
59.557
56.404
52.762
52.045
48.250
48.038
47.825
47.613
47.396
39.261
30.189
28.368
28.132
26.319
24.524
23.681
23.116
22.513
22.092
19.640
11.999
11.714
1.989
1.856
1.207
Chemical Shift (ppm)

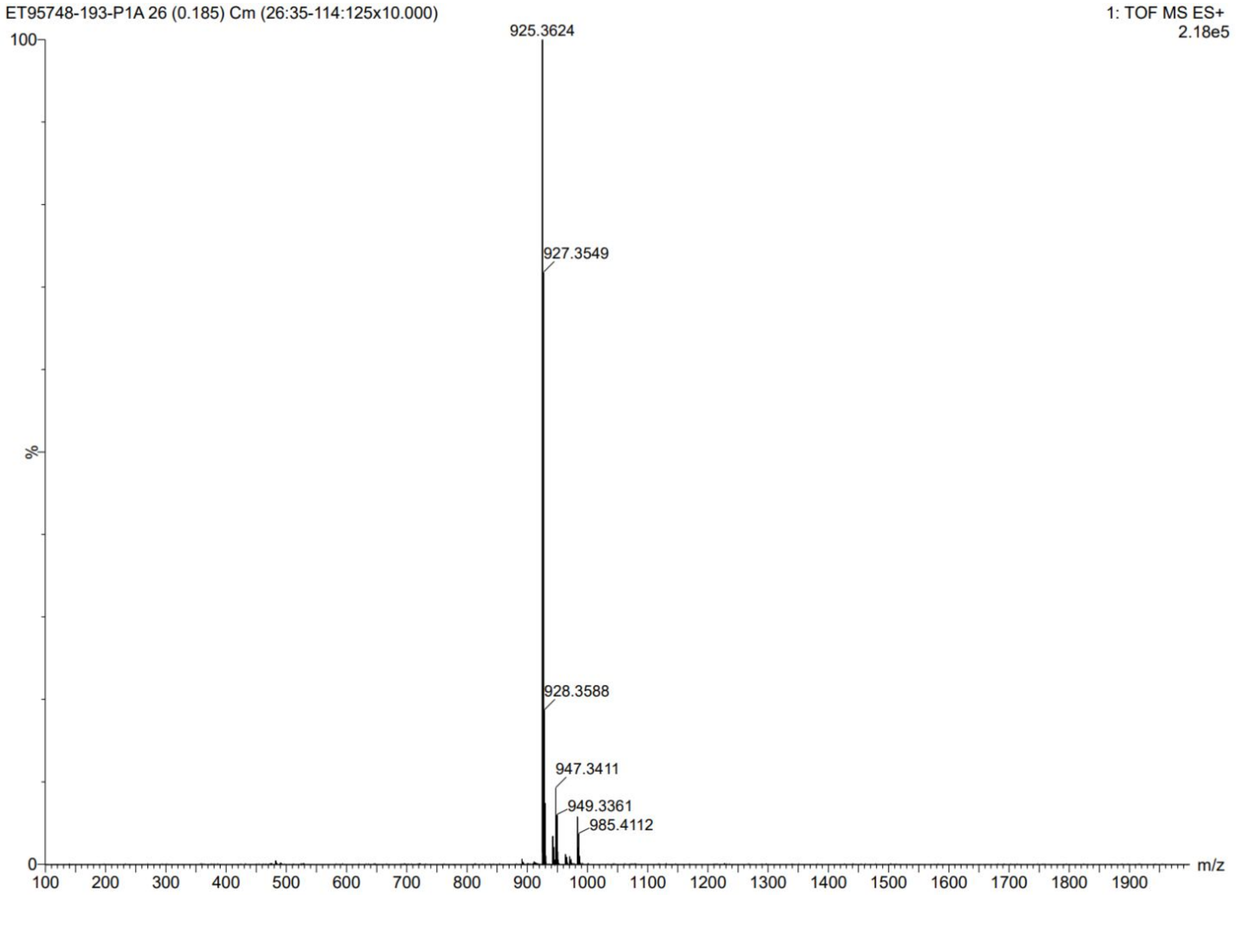
ET95748-193-P1A 26 (0.185) Cm (26:35-114:125x10.000)
1: TOF MS ES+
2.18e5
925.3624
927.3549
928.3588
947.3411
949.3361
985.4112
%
m/z

DMPK Assays

MDCK-MDR1 Permeability: Cell-based compound permeability was measured in Madin Darby Canine Kidney (MDCK) cells transfected with the human MDR1 gene, which encoded the P-glycoprotein (P-gp) efflux transporter. In this experiment, MDCK-MDR1 cells were plated into 96-well plates at 7,500 cells per well (75 µL) and incubated for three days at 37ºC with 5% CO2. Cells were washed with Hank's Balanced Salt Solution (HBSS) with 5 mM HEPES for 30 minutes before starting the experiment. Test compound solutions were prepared by diluting DMSO stock into HBSS buffer with 10 µM of P-gp inhibitor Elacridar, resulting in a final test compound concentration at 6 µM with final DMSO concentration of 0.2% (v/v). Prior to the experiment, cell monolayer integrity is verified by transendothelial electrical resistance (TEER). Transport experiment was initiated by adding test compounds to the apical (75 µL) side and blank HBSS buffer with Elacridar in the basolateral (250 µL) side. Assay is carried out in duplicate. Transport plates were incubated at 37ºC for one hour in a humidified incubator with 5% CO2. Samples were taken from the apical and basolateral compartments after one hour and analyzed by liquid chromatography with tandem mass spectrometry (LC/MS/MS). Cyclosporin was used as a reference control. Apparent permeability (Papp) values are calculated using the following equation

Papp = (dQ/dt)/A/C0

Where dQ/dt is the initial rate of amount of test compound transported across cell monolayer, A is the surface area of the filter membrane, and C0 is the initial concentration of the test compound, calculated for each direction using a 4-point calibration curve by LC/MS/MS. All samples were analyzed on LC/MS/MS using an AB Sciex API 4000 instrument, coupled to a Shimadzu LC-20AD LC Pump system. Analytical samples were separated using a Waters Atlantis T3 dC18 reverse phase HPLC column (20 mm x 2.1 mm) at a flow rate of 0.5 mL/min. The mobile phase consists of 0.1% formic acid in water (solvent A) and 0.1% formic acid in 100% acetonitrile (solvent B).

Plasma Protein Binding: Plasma protein binding was evaluated using ultracentrifugation method. Mouse plasma (K2-EDTA) was obtained from BioIVT, pH was adjusted to 7.4 before the assay. Test compounds in DMSO stock solution at 1 mM were spiked into the plasma to make a final concentration of 2 µM and incubate for 30 minutes at 37°C prior to the

centrifugation. An equal volume of blank phosphate buffer saline (PBS) was added to the plate to make the matrix at 50:50 plasma: buffer. Spiked plasma was transferred (200 µL/tube) to four centrifuge tubes (Polycarbonate, 7x20 mm, Beckman Coulter, Inc., Brea, CA). Two tubes were used for actual ultracentrifugation samples and two other tubes are used for reference samples. Actual samples were centrifuged at 85,000 rpm (346,000 xg) for 4 hours at 37ºC, while the reference samples were incubated at 37°C for 4 hours. After 4 hours, gently mix the reference samples. The supernatants (30 µL) were removed from the two replicate samples and the mixed reference samples (30 uL) were removed from the two mixed reference samples. The supernatant and mixed plasma samples were diluted with an equal volume of blank plasma and pH 7.4 PBS, respectively. At last, 150 µL of quench solution (50% acetonitrile, 50% methanol and 0.05% formic acid) containing internal standards (bucetin) was added to each well. Plates were sealed and vortex for 20 minutes, then centrifuged at 4°C for 15 minutes at 4,000 rpm (163,000 xg). The supernatant was transferred to fresh plates for LC/MS/MS analysis. Reference compound propranolol was included in every experiment. All samples were analyzed on LC/MS/MS using an AB Sciex API 4000 instrument, coupled to a Shimadzu LC-20AD LC Pump system. Analytical samples were separated using a Waters Atlantis T3 dC18 reverse phase HPLC column (20 mm x 2.1 mm) at a flow rate of 0.5 mL/min. The mobile phase consisted of 0.1% formic acid in water (solvent A) and 0.1% formic acid in acetonitrile (solvent B).

The percentage of test compound bound to protein was calculated by the following equation:

% Free = (Concentration of supernatant/Concentration of reference plasma) × 100%

% Bound = 100% - % Free

Kinetic Solubility: Kinetic solubility was measured based on samples supplied in DMSO solution. Test compounds at 10 mM in DMSO were diluted with PBS buffer (pH 7.4) resulting in a final DMSO concentration of 5% and mixed by shaking for 1.5 hours at room temperature followed by vacuum filtration. The samples were then assayed via reverse phase HPLC with UV detection. Quantitation was achieved by reference to a three-point standard curve constructed via serial dilution of drug substance dissolved in 100% DMSO. Amiodarone and testosterone were used as reference controls.

Mouse Liver Microsomal Stability Assay: Microsomal stability was assessed in pooled liver microsomes (0.5 mg/mL) incubated in 100 mM phosphate buffer (pH 7.4) containing 5 mM $MgCl_2$ at 37 °C. Reactions (final volume 400 µL) were initiated by addition of 1 mM NADPH following a 5 min pre-warm, with test compounds or verapamil (positive control) added at 1 µM. Negative controls were prepared without NADPH to account for non-enzymatic degradation. Aliquots (50 µL) were withdrawn at 0, 15, 30, 45, and 60 min and quenched with four volumes of ice-cold acetonitrile containing internal standards (100 nM alprazolam, 200 nM imipramine, 200 nM labetalol, and 2 µM ketoprofen). After centrifugation (3220 × g, 40 min), supernatants were diluted 1:1 with water and analyzed by LC–MS/MS. The natural logarithm of the remaining parent compound versus time was used to calculate the first-order rate constant (k) and in vitro half-life ($t_{1/2} = 0.693/k$). Intrinsic clearance (CLint, µL/min/mg protein) was determined from $t_{1/2}$ using standard scaling.

Met ID of Compound 2

Metabolite Identification: Metabolite identification was performed using mouse plasma samples pooled from mouse samples collected between 5 min to 1 hour post an Intravenous (IV) dose at 5 mg/kg. 25 µL of blood was taken from each mouse. 60 µL of pooled mouse sample was precipitated with 120 µL of Acetonitrile (ACN) following by vigorous vortex mixing for 5 minutes. The samples were then centrifuged at 4°C, 20,000 g for 30 minutes. The clear supernatant was collected and transferred prior to liquid chromatography – high resolution mass spectrometry (LC-HRMS) analysis. 15 µL of sample was injected, with autosampler temperature at 5°C. The metabolite profiling was performed using LC-HRMS. 0.1% (v/v) Formic Acid in purified water was used as mobile phase A, and pure ACN was used as mobile phase B for the LC. Thermo Accucore C18, 2.1 × 150 mm, 2.6 µm column was used with column compartment temperature at 40°C. The LC gradient started with 10% B from 0 to 0.3 minutes and gradually increased to 50% B from 0.3 to 10.5 minutes, followed by increasing from 50% to 90% from 10.5 to 14 minutes. Then the gradient was held at 90% B for 4 minutes, and re-equilibration back to 10%B from 18 minutes to 22 minutes. A full MS scan from 100 to 1500 m/z in both positive and negative mode was performed. Data dependent-MS^2 (tandem mass spectrometry) was performed with stepped collision energy (CE) at 10, 25, and 40V.

Compound 2 was dosed in mouse at 5 mg/kg IV, and mouse blood samples were collected and pooled from 5 min to 1 hour post dose to perform metabolite identification analysis. The parent compound 2, and its metabolites were detected in positive mode using liquid chromatography – high resolution mass spectrometry (LC-HRMS). Based on relative MS abundance (calculated based on MS peak area), 90.6% of parent compound 2 was observed. 2 was observed as $[M+H]^+$ at 15.1 min. Oxidation metabolite of compound 2 M988 with the relative abundance of 2.5%, was observed as $[M+H]^+$ at retention time 13.2 minutes. Its isomer, M988-Isomer with the relative abundance of 1.3%, was observed as $[M+H]^+$ at retention time 13.3 minutes. An unknown peak, with m/z same as the parent compound, was observed at retention time of 14 minutes.

Table S5: WI-38 GI_{50} Values

Compound #	WI-38 GI_{50} (μM)
**2**	9.27
**3**	18.4
**4**	14.1
**5**	NC
**6**	NC
**7**	11
**8**	7.44
**9**	7.17
**10**	9.15
**11**	20
**12**	8.62
**13**	8.14
**14**	NC
**15**	12.7
**16**	10.7
**17**	17.5
**18**	20
**19**	20
**20**	16
**21**	8.31
**22**	20
**23**	20
**24**	8.06
**25**	20
**26**	5.89

**27**	20
**28**	20
**29**	20
**30**	8.53
**31**	11.6
**32**	9.54
**33**	18.2

NC = Not Collected

Table S6: Mouse Liver Microsome Stability

Metabolic stability in mouse (liver microsomes)	In vitro t1/2 (min)	Pred CL (mL/min/kg)	E_H
Compound 2	4.66	83.9	0.93
Compound 29	4.53	84.2	0.94
Compound 33	12.6	75.6	0.83

In Vitro Safety Panels

KINOMEscan™ Profiling Assay conducted by Eurofins

Compound **33** was tested in this panel of 468 kinase assays using standard conditions to identify potential off-target activity.

Protocol Description for Kinase Assays.

For most assays, kinase-tagged T7 phage strains were grown in parallel in 24-well blocks in an E. coli host derived from the BL21 strain. E. coli were grown to log-phase and infected with T7 phage from a frozen stock (multiplicity of infection = 0.4) and incubated with shaking at 32°C until lysis (90-150 minutes). The lysates were centrifuged (6,000 x g) and filtered (0.2µm) to remove cell debris. The remaining kinases were produced in HEK-293 cells and subsequently tagged with DNA for qPCR detection. Streptavidin-coated magnetic beads were treated with biotinylated small molecule ligands for 30 minutes at room temperature to generate affinity resins for kinase assays. The liganded beads were blocked with excess biotin and washed with

blocking buffer (SeaBlock (Pierce), 1 % BSA, 0.05 % Tween 20, 1 mM DTT) to remove unbound ligand and to reduce non-specific phage binding. Binding reactions were assembled by combining kinases, liganded affinity beads, and test compounds in 1x binding buffer (20 % SeaBlock, 0.17x PBS, 0.05 % Tween 20, 6 mM DTT). Test compounds were prepared as 40x stocks in 100% DMSO and directly diluted into the assay. All reactions were performed in polypropylene 384-well plates in a final volume of 0.02 ml. The assay plates were incubated at room temperature with shaking for 1 hour and the affinity beads were washed with wash buffer (1x PBS, 0.05 % Tween 20). The beads were then re-suspended in elution buffer (1x PBS, 0.05 % Tween 20, 0.5 µM non-biotinylated affinity ligand) and incubated at room temperature with shaking for 30 minutes. The kinase concentration in the eluates was measured by qPCR. Confidential CIR001-01-p-00001 9/20/2021 2 of 19 Percent Control (%Ctrl). The compound(s) were screened at the concentration(s) requested, and results for primary screen binding interactions are reported as '% Ctrl', where lower numbers indicate stronger hits in the matrix on the following page(s).

Percent Control (%Ctrl) Calculation:

$$\text{Percent Control (\%Ctrl)} = \left(\frac{\text{Test compound signal} - \text{positive control signal}}{\text{Negative control signal} - \text{positive control signal}} \right) \text{X } 100$$

Where:

test compound = compound submitted by Circle Pharma Inc

negative control = DMSO (100%Ctrl)

positive control = control compound (0%Ctrl)

**Table S6. Results from KINOMEscan™ Profiling Assay with Compound 33 at 1 µM and 10 µM test concentrations** **Target Gene Symbol**	**%Control at 1 µM**	**%Control at 10 µM**
AAK1	100	100
ABL1(E255K)-phosphorylated	92	100

**<u>Table S6. Results from KINOMEscan™ Profiling Assay with Compound 33 at 1 µM and 10 µM test concentrations</u> Target Gene Symbol**	**%Control at 1 µM**	**%Control at 10 µM**
ABL1(F317I)-nonphosphorylated	100	94
ABL1(F317I)-phosphorylated	81	93
ABL1(F317L)-nonphosphorylated	100	100
ABL1(H396P)-nonphosphorylated	87	92
ABL1(H396P)-phosphorylated	91	100
ABL1(M351T)-phosphorylated	75	82
ABL1(Q252H)-nonphosphorylated	82	83
ABL1(Q252H)-phosphorylated	100	100
ABL1(T315I)-nonphosphorylated	100	93
ABL1(T315I)-phosphorylated	83	85
ABL1(Y253F)-phosphorylated	100	100
ABL1-nonphosphorylated	83	84
ABL1-phosphorylated	100	100
ABL2	100	100
ACVR1	100	100
ACVR1B	83	100
ACVR2A	88	100
ACVR2B	72	98
ACVRL1	100	100
ADCK3	100	99
ADCK4	100	87
AKT1	87	95
AKT2	100	100
AKT3	100	98
ALK	89	99
ALK(C1156Y)	53	60
ALK(L1196M)	71	76
AMPK-alpha1	100	64

**<u>Table S6. Results from KINOMEscan™ Profiling Assay with Compound 33 at 1 µM and 10 µM test concentrations</u> Target Gene Symbol**	**%Control at 1 µM**	**%Control at 10 µM**
AMPK-alpha2	100	96
ANKK1	92	68
ARK5	99	93
ASK1	100	100
ASK2	90	100
AURKA	91	83
AURKB	95	90
AURKC	100	100
AXL	100	100
BIKE	100	100
BLK	100	100
BMPR1A	100	96
BMPR1B	72	72
BMPR2	79	61
BMX	100	88
BRAF	100	93
BRAF(V600E)	98	88
BRK	85	86
BRSK1	100	79
BRSK2	88	98
BTK	100	100
BUB1	92	88
CAMK1	100	100
CAMK1B	100	100
CAMK1D	100	100
CAMK1G	100	100
CAMK2A	100	100
CAMK2B	100	100

**<u>Table S6. Results from KINOMEscan™ Profiling Assay with Compound 33 at 1 µM and 10 µM test concentrations</u> Target Gene Symbol**	**%Control at 1 µM**	**%Control at 10 µM**
CAMK2D	100	100
CAMK2G	97	100
CAMK4	100	100
CAMKK1	93	100
CAMKK2	94	100
CASK	85	100
CDC2L1	92	83
CDC2L2	88	95
CDC2L5	76	80
CDK11	95	89
CDK2	100	100
CDK3	90	100
CDK4	100	91
CDK4-cyclinD1	99	100
CDK4-cyclinD3	73	82
CDK5	92	99
CDK7	70	100
CDK8	88	90
CDK9	100	81
CDKL1	100	96
CDKL2	100	97
CDKL3	97	77
CDKL5	100	100
CHEK1	100	100
CHEK2	92	95
CIT	100	100
CLK1	88	100
CLK2	100	100

**<u>Table S6. Results from KINOMEscan™ Profiling Assay with Compound 33 at 1 µM and 10 µM test concentrations</u> Target Gene Symbol**	**%Control at 1 µM**	**%Control at 10 µM**
CLK3	82	100
CLK4	100	96
CSF1R	100	80
CSF1R-autoinhibited	100	100
CSK	90	99
CSNK1A1	85	58
CSNK1A1L	100	100
CSNK1D	100	100
CSNK1E	100	100
CSNK1G1	100	86
CSNK1G2	85	100
CSNK1G3	100	100
CSNK2A1	73	80
CSNK2A2	82	83
CTK	100	88
DAPK1	97	100
DAPK2	100	100
DAPK3	100	100
DCAMKL1	86	74
DCAMKL2	82	93
DCAMKL3	100	100
DDR1	100	100
DDR2	100	100
DLK	62	79
DMPK	100	100
DMPK2	100	92
DRAK1	99	100
DRAK2	100	100

**Table S6. Results from KINOMEscan™ Profiling Assay with Compound 33 at 1 μM and 10 μM test concentrations** **Target Gene Symbol**	**%Control at 1 μM**	**%Control at 10 μM**
DYRK1A	100	100
DYRK1B	100	100
DYRK2	100	81
EGFR	100	100
EGFR(E746-A750del)	100	93
EGFR(G719C)	100	100
EGFR(G719S)	100	100
EGFR(L747-E749del, A750P)	100	92
EGFR(L747-S752del, P753S)	97	100
EGFR(L747-T751del,Sins)	100	100
EGFR(L858R)	100	90
EGFR(L858R,T790M)	72	82
EGFR(L861Q)	100	100
EGFR(S752-I759del)	100	100
EGFR(T790M)	71	91
EIF2AK1	74	81
EPHA1	100	99
EPHA2	97	73
EPHA3	78	94
EPHA4	100	100
EPHA5	93	96
EPHA6	100	87
EPHA7	100	100
EPHA8	100	100
EPHB1	100	100
EPHB2	91	86
EPHB3	100	92
EPHB4	100	89

**<u>Table S6. Results from KINOMEscan™ Profiling Assay with Compound 33 at 1 µM and 10 µM test concentrations</u> Target Gene Symbol**	**%Control at 1 µM**	**%Control at 10 µM**
EPHB6	100	100
ERBB2	96	91
ERBB3	42	100
ERBB4	98	100
ERK1	91	100
ERK2	93	100
ERK3	100	100
ERK4	100	82
ERK5	100	95
ERK8	86	100
ERN1	100	75
FAK	100	100
FER	100	100
FES	100	83
FGFR1	100	100
FGFR2	100	86
FGFR3	100	97
FGFR3(G697C)	100	80
FGFR4	100	91
FGR	92	74
FLT1	100	100
FLT3	92	96
FLT3(D835H)	100	100
FLT3(D835V)	79	80
FLT3(D835Y)	98	100
FLT3(ITD)	97	100
FLT3(ITD,D835V)	78	77
FLT3(ITD,F691L)	100	100

**<u>Table S6. Results from KINOMEscan™ Profiling Assay with Compound 33 at 1 μM and 10 μM test concentrations</u> Target Gene Symbol**	**%Control at 1 μM**	**%Control at 10 μM**
FLT3(K663Q)	73	92
FLT3(N841I)	86	100
FLT3(R834Q)	100	100
FLT3-autoinhibited	73	69
FLT4	100	100
FRK	100	90
FYN	85	88
GAK	92	81
GCN2(Kin.Dom.2,S808G)	100	100
GRK1	80	82
GRK2	91	100
GRK3	70	66
GRK4	100	100
GRK7	72	92
GSK3A	87	88
GSK3B	100	100
HASPIN	84	77
HCK	96	59
HIPK1	94	53
HIPK2	100	100
HIPK3	100	100
HIPK4	100	43
HPK1	69	94
HUNK	100	100
ICK	74	75
IGF1R	95	100
IKK-alpha	92	90
IKK-beta	100	100

**<u>Table S6. Results from KINOMEscan™ Profiling Assay with Compound 33 at 1 µM and 10 µM test concentrations</u> Target Gene Symbol**	**%Control at 1 µM**	**%Control at 10 µM**
IKK-epsilon	76	100
INSR	68	91
INSRR	88	96
IRAK1	93	99
IRAK3	87	100
IRAK4	100	100
ITK	100	100
JAK1(JH1domain-catalytic)	100	93
JAK1(JH2domain-pseudokinase)	100	100
JAK2(JH1domain-catalytic)	67	69
JAK3(JH1domain-catalytic)	70	67
JNK1	96	89
JNK2	81	72
JNK3	73	69
KIT	96	84
KIT(A829P)	100	100
KIT(D816H)	54	51
KIT(D816V)	100	99
KIT(L576P)	89	83
KIT(V559D)	94	92
KIT(V559D,T670I)	92	98
KIT(V559D,V654A)	98	100
KIT-autoinhibited	100	100
LATS1	100	95
LATS2	100	100
LCK	85	100
LIMK1	100	100
LIMK2	100	100

**Table S6. Results from KINOMEscan™ Profiling Assay with Compound 33 at 1 µM and 10 µM test concentrations** **Target Gene Symbol**	**%Control at 1 µM**	**%Control at 10 µM**
LKB1	100	100
LOK	100	55
LRRK2	100	100
LRRK2(G2019S)	62	100
LTK	100	96
LYN	71	85
LZK	100	100
MAK	76	58
MAP3K1	80	78
MAP3K15	61	69
MAP3K2	74	61
MAP3K3	81	48
MAP3K4	100	93
MAP4K2	100	100
MAP4K3	98	100
MAP4K4	82	90
MAP4K5	81	100
MAPKAPK2	100	100
MAPKAPK5	66	67
MARK1	89	99
MARK2	77	90
MARK3	100	100
MARK4	94	86
MAST1	100	100
MEK1	94	92
MEK2	93	84
MEK3	77	87
MEK4	94	83

**<u>Table S6. Results from KINOMEscan™ Profiling Assay with Compound 33 at 1 μM and 10 μM test concentrations</u> Target Gene Symbol**	**%Control at 1 μM**	**%Control at 10 μM**
MEK5	79	81
MEK6	100	100
MELK	100	100
MERTK	91	100
MET	100	100
MET(M1250T)	100	100
MET(Y1235D)	100	93
MINK	93	74
MKK7	100	98
MKNK1	88	93
MKNK2	69	65
MLCK	96	95
MLK1	92	100
MLK2	100	100
MLK3	96	100
MRCKA	100	98
MRCKB	93	100
MST1	100	90
MST1R	77	55
MST2	100	100
MST3	100	100
MST4	100	100
MTOR	77	100
MUSK	95	92
MYLK	56	96
MYLK2	86	95
MYLK4	100	100
MYO3A	100	100

**Table S6. Results from KINOMEscan™ Profiling Assay with Compound 33 at 1 µM and 10 µM test concentrations** **Target Gene Symbol**	**%Control at 1 µM**	**%Control at 10 µM**
MYO3B	100	96
NDR1	88	82
NDR2	76	81
NEK1	100	100
NEK10	100	100
NEK11	64	60
NEK2	94	75
NEK3	82	87
NEK4	75	74
NEK5	94	99
NEK6	85	96
NEK7	100	92
NEK9	98	99
NIK	68	73
NIM1	98	100
NLK	73	100
OSR1	57	59
PAK1	100	100
PAK2	89	100
PAK3	100	100
PAK4	89	100
PAK6	100	100
PAK7	71	88
PCTK1	100	100
PCTK2	87	85
PCTK3	100	100
PDGFRA	75	83
PDGFRB	100	96

**<u>Table S6. Results from KINOMEscan™ Profiling Assay with Compound 33 at 1 µM and 10 µM test concentrations</u> Target Gene Symbol**	**%Control at 1 µM**	**%Control at 10 µM**
PDPK1	69	76
PFCDPK1(P.falciparum)	100	89
PFPK5(P.falciparum)	71	80
PFTAIRE2	100	100
PFTK1	85	100
PHKG1	100	88
PHKG2	100	100
PIK3C2B	94	92
PIK3C2G	62	82
PIK3CA	90	89
PIK3CA(C420R)	94	98
PIK3CA(E542K)	98	94
PIK3CA(E545A)	100	91
PIK3CA(E545K)	91	93
PIK3CA(H1047L)	81	85
PIK3CA(H1047Y)	100	96
PIK3CA(I800L)	96	99
PIK3CA(M1043I)	79	99
PIK3CA(Q546K)	76	84
PIK3CB	100	100
PIK3CD	100	100
PIK3CG	66	69
PIK4CB	75	79
PIKFYVE	100	100
PIM1	94	100
PIM2	87	96
PIM3	90	100
PIP5K1A	87	87

<u>**Table S6. Results from KINOMEscan™ Profiling Assay with Compound 33 at 1 µM and 10 µM test concentrations**</u> **Target Gene Symbol**	**%Control at 1 µM**	**%Control at 10 µM**
PIP5K1C	64	77
PIP5K2B	88	98
PIP5K2C	98	92
PKAC-alpha	77	85
PKAC-beta	100	100
PKMYT1	85	100
PKN1	93	100
PKN2	83	99
PKNB(M.tuberculosis)	85	100
PLK1	99	93
PLK2	94	94
PLK3	93	85
PLK4	90	90
PRKCD	96	94
PRKCE	100	100
PRKCH	100	95
PRKCI	92	79
PRKCQ	44	74
PRKD1	100	100
PRKD2	95	90
PRKD3	100	100
PRKG1	76	100
PRKG2	80	84
PRKR	100	100
PRKX	100	90
PRP4	100	100
PYK2	97	97
QSK	70	93

**<u>Table S6. Results from KINOMEscan™ Profiling Assay with Compound 33 at 1 µM and 10 µM test concentrations</u> Target Gene Symbol**	**%Control at 1 µM**	**%Control at 10 µM**
RAF1	85	100
RET	100	100
RET(M918T)	90	100
RET(V804L)	86	100
RET(V804M)	100	100
RIOK1	89	100
RIOK2	100	92
RIOK3	100	100
RIPK1	100	100
RIPK2	100	93
RIPK4	65	69
RIPK5	84	88
ROCK1	94	98
ROCK2	70	68
ROS1	78	100
RPS6KA4(Kin.Dom.1-N-terminal)	97	100
RPS6KA4(Kin.Dom.2-C-terminal)	68	78
RPS6KA5(Kin.Dom.1-N-terminal)	100	94
RPS6KA5(Kin.Dom.2-C-terminal)	100	100
RSK1(Kin.Dom.1-N-terminal)	100	100
RSK1(Kin.Dom.2-C-terminal)	93	100
RSK2(Kin.Dom.1-N-terminal)	78	70
RSK2(Kin.Dom.2-C-terminal)	92	91
RSK3(Kin.Dom.1-N-terminal)	73	95
RSK3(Kin.Dom.2-C-terminal)	95	100
RSK4(Kin.Dom.1-N-terminal)	79	68
RSK4(Kin.Dom.2-C-terminal)	82	100
S6K1	100	100

**<u>Table S6. Results from KINOMEscan™ Profiling Assay with Compound 33 at 1 µM and 10 µM test concentrations</u> Target Gene Symbol**	**%Control at 1 µM**	**%Control at 10 µM**
SBK1	59	52
SGK	83	89
SGK2	100	100
SGK3	100	100
SIK	84	100
SIK2	100	100
SLK	89	100
SNARK	82	65
SNRK	94	86
SRC	97	100
SRMS	97	100
SRPK1	100	100
SRPK2	100	100
SRPK3	97	69
STK16	100	100
STK33	97	100
STK35	100	100
STK36	86	94
STK39	58	65
SYK	93	100
SgK110	46	43
TAK1	100	100
TAOK1	100	100
TAOK2	100	100
TAOK3	85	100
TBK1	66	90
TEC	99	100
TESK1	100	79

**<u>Table S6. Results from KINOMEscan™ Profiling Assay with Compound 33 at 1 μM and 10 μM test concentrations</u> Target Gene Symbol**	**%Control at 1 μM**	**%Control at 10 μM**
TGFBR1	84	100
TGFBR2	99	100
TIE1	93	100
TIE2	100	95
TLK1	100	100
TLK2	100	100
TNIK	70	100
TNK1	100	100
TNK2	100	100
TNNI3K	95	73
TRKA	78	99
TRKB	70	64
TRKC	100	100
TRPM6	84	84
TSSK1B	100	90
TSSK3	93	75
TTK	99	92
TXK	100	88
TYK2(JH1domain-catalytic)	100	100
TYK2(JH2domain-pseudokinase)	100	100
TYRO3	74	100
ULK1	67	56
ULK2	79	90
ULK3	85	77
VEGFR2	81	70
VPS34	80	89
VRK2	100	100
WEE1	78	100

**<u>Table S6. Results from KINOMEscan™ Profiling Assay with Compound 33 at 1 µM and 10 µM test concentrations</u> Target Gene Symbol**	**%Control at 1 µM**	**%Control at 10 µM**
WEE2	99	100
WNK1	100	100
WNK2	96	86
WNK3	100	100
WNK4	57	51
YANK1	88	88
YANK2	100	100
YANK3	94	100
YES	92	100
YSK1	100	94
YSK4	61	58
ZAK	100	100
ZAP70	100	100
p38-alpha	100	100
p38-beta	100	88
p38-delta	94	59
p38-gamma	59	100

<u>Eurofins SAFETYscan 47 E/IC50 ELECT panel assay protocols and results.</u>

Compound **33** was tested in this panel of 78 assays to evaluate off-target pharmacological activity against a board range of biological targets that are commonly associated with safety risks. The panel includes 48 functional assays (20 Calcium Flux assays and 28 cAMP assays) against G-protein coupled receptors (GPCR), 13 functional assays against ion channels, 6 functional assays against non-kinase enzymes, 4 assays against nuclear hormone receptor (NHR), 4 binding enzymes against kinase, and 3 functional assays against transporters.

Calcium Mobilization Assay

The calcium mobilization assay evaluates GPCR activity via Gq-mediated signaling using a calcium-sensitive dye. Cells are seeded into 384-well plates and loaded with dye in a specialized buffer. For agonist testing, compounds are added post-dye loading and calcium flux is measured in real-time using a FLIPR Tetra system. For antagonist testing, cells are pre-incubated with the compound, followed by stimulation with an EC80 concentration of a known agonist. Fluorescence changes are recorded over two minutes, and data analysis calculates percent activity or inhibition using area under the curve metrics, with values capped between 0% and 100% for primary screens.

GPCR cAMP Modulation Assay

The cAMP assay protocol involves using DiscoverX's HitHunter cAMP XS+ system to assess GPCR activity via Gs and Gi signaling pathways. Cells are seeded into 384-well plates and treated with test compounds under different formats: Gs agonist, Gi agonist (with forskolin), and antagonist (with EC80 agonist). After incubation, signal detection is performed using a chemiluminescent readout following lysis and enzyme complementation steps. Data analysis calculates percent activity or inhibition based on relative luminescence units (RLU), with responses capped between 0% and 100% for primary screens.

Ion Channel Assays

The ion channel assay protocol involves seeding cells into 384-well plates and loading them with a fluorescent dye in the presence of freshly prepared probenecid. For agonist/opener assays, compounds are added directly to induce ion channel activity, while antagonist/blocker assays involve pre-incubation with the compound followed by stimulation with an EC80 agonist. After incubation, fluorescence changes are measured using a FLIPR Tetra system. Data analysis calculates percent activity or inhibition based on relative luminescence units (RLU), with results capped between 0% and 100% for primary screens.

KINOMEscan Kinase Binding Assays

The binding to 4 target kinases (INSR, LCK, ROCK1, VEGFR2) used KINOMEscan platform to measure compound interactions with kinase active sites via a competition binding format. Kinase-tagged phage or HEK293-expressed kinases are incubated with immobilized ligands and test compounds in 384-well plates. Compounds that bind the kinase prevent its capture on the

ligand-coated beads, and the remaining kinase is quantified using qPCR of DNA tags. Binding affinity (Kd) is calculated from dose-response curves using the Hill equation, and percent response is derived from control comparisons, with values capped between 0% and 100% for primary screens.

Nuclear Hormone Receptor Assays

The nuclear hormone receptor (NHR) assay protocol uses PathHunter cell lines to evaluate receptor activation via protein interaction or nuclear translocation. Cells are seeded into 384-well plates and incubated with test compounds under agonist or antagonist formats. Agonist assays involve direct incubation, while antagonist assays include pre-incubation followed by EC80 agonist challenge. Signal detection is performed using chemiluminescence after adding PathHunter detection reagents. Data analysis calculates percent activity or inhibition, with inverse agonist activity assessed for constitutively active targets. All responses are normalized and capped between 0% and 100% for primary screens.

Transporter Assays

The transporter assay protocol involves seeding cells into 384-well plates and incubating them with test compounds in HBSS/BSA buffer. After a 30-minute incubation at 37 °C, a fluorescent dye is added to assess neurotransmitter uptake inhibition. Cells are further incubated for 30–60 minutes before fluorescence signal detection using a PerkinElmer Envision reader. Data analysis calculates percent inhibition based on relative fluorescence units (RFU), comparing test samples to vehicle and positive controls, with responses capped between 0% and 100% for primary screens.

Non-Kinase Enzymatic Assays

The enzymatic assay protocol involves measuring enzyme activity by tracking substrate consumption or product formation using various detection methods—absorbance, fluorescence, or luminescence. Enzymes such as AChE, COX1/2, MAOA, and PDEs are incubated with test compounds before substrate addition. Specific conditions and reagents are used for each enzyme type, and reactions are terminated appropriately to enable signal detection using a PerkinElmer Envision reader. Data analysis calculates percent inhibition relative to vehicle and positive controls, with results capped between 0% and 100% for primary screens.

Table S7. Results with Compound 33 at up to 10 µM: agonist effect

Target Class	Assay Name	Target	EC_{50} (μM)	Max Response (%)
GPCR	Calcium Flux	ADORA2A	>10	2.87
GPCR	Calcium Flux	ADRA1A	>10	1.15
GPCR	Calcium Flux	AVPR1A	>10	1.32
GPCR	Calcium Flux	CCKAR	>10	0
GPCR	Calcium Flux	CHRM1	>10	1.8
GPCR	Calcium Flux	CHRM3	>10	0
GPCR	Calcium Flux	EDNRA	>10	0
GPCR	Calcium Flux	HRH1	>10	0.79
GPCR	Calcium Flux	HTR2A	>10	0.14
GPCR	Calcium Flux	HTR2B	>10	3.24
GPCR	cAMP	ADRA2A	>10	4.4
GPCR	cAMP	ADRB1	>10	0.92
GPCR	cAMP	ADRB2	>10	1.15
GPCR	cAMP	CHRM2	10	72.09
GPCR	cAMP	CNR1	>10	5.11
GPCR	cAMP	CNR2	>10	8.65
GPCR	cAMP	DRD1	>10	0.21
GPCR	cAMP	DRD2S	>10	14.93
GPCR	cAMP	HRH2	>10	0.05
GPCR	cAMP	HTR1A	>10	13.96
GPCR	cAMP	HTR1B	>10	20.87
GPCR	cAMP	OPRD1	>10	27.15
GPCR	cAMP	OPRK1	>10	19.67
GPCR	cAMP	OPRM1	>10	34.17
Ion Channel	Opener	GABAA	>10	2.5
Ion Channel	Opener	HTR3A	>10	3.93
Ion Channel	Opener	KvLQT1/minK	>10	2.8
Ion Channel	Opener	nAChR(a4/b2)	>10	4.96
Ion Channel	Opener	NMDAR(1A/2B)	>10	2.93

NHR	Translocation	GR	>10	0
NHR	Protein Interaction	AR	>10	0.11

Table S8. Results with Compound 33 at up to 10 μM: antagonist effect

Target Class	Assay Name	Target	EC_{50} (μM)	Max Response (%)
GPCRCalcium FluxADORA2A>102.33GPCRCalcium FluxADRA1A>100GPCR	Calcium Flux	AVPR1A	>10	4.05
GPCR	Calcium Flux	CCKAR	>10	0
GPCR	Calcium Flux	CHRM1	>10	0
GPCR	Calcium Flux	CHRM3	>10	0
GPCR	Calcium Flux	EDNRA	>10	0
GPCR	Calcium Flux	HRH1	>10	0
GPCR	Calcium Flux	HTR2A	>10	0
GPCR	Calcium Flux	HTR2B	>10	0
GPCR	cAMP	ADRA2A	>10	0
GPCR	cAMP	ADRB1	>10	39.4
GPCR	cAMP	ADRB2	>10	49.01
GPCR	cAMP	CHRM2	>10	0
GPCR	cAMP	CNR1	>10	0
GPCR	cAMP	CNR2	>10	0
GPCR	cAMP	DRD1	>10	45.51
GPCR	cAMP	DRD2S	>10	0
GPCR	cAMP	HRH2	>10	34.65
GPCR	cAMP	HTR1A	>10	0
GPCR	cAMP	HTR1B	>10	0
GPCR	cAMP	OPRD1	>10	0
GPCR	cAMP	OPRK1	>10	0
GPCR	cAMP	OPRM1	>10	0
NHR	Translocation	AR	>10	10.72
NHR	Protein Interaction	GR	>10	15.62

Table S9. Results with Compound 33 at up to 10 μM: Ion channel opener and blocker effect

Target Class	Mode	Target	EC_{50} or IC_{50} (μM)	Max Response (%)
Ion Channel	Opener	GABAA	>10	2.5
Ion Channel	Opener	HTR3A	>10	3.93
Ion Channel	Opener	KvLQT1/minK	>10	2.8
Ion Channel	Opener	nAChR(a4/b2)	>10	4.96
Ion Channel	Opener	NMDAR(1A/2B)	>10	2.93
Ion Channel	Blocker	CAV1.2	>10	0
Ion Channel	Blocker	GABAA	>10	7.87
Ion Channel	Blocker	hERG	>10	13.55
Ion Channel	Blocker	HTR3A	>10	0
Ion Channel	Blocker	KvLQT1/minK	>10	18.71
Ion Channel	Blocker	NAV1.5	>10	25.15
Ion Channel	Blocker	NMDAR(1A/2B)	>10	0
Ion Channel	Blocker	nAChR(a4/b2)	>10	0

Table S10. Results with Compound 33 at up to 10 μM: Enzymatic and transporter inhibition effect

Target Class	Assay Name	Target	IC_{50} (μM)	Max Response (%)
Kinase	Binding	INSR	>10	19.04
Kinase	Binding	LCK	>10	21.94
Kinase	Binding	ROCK1	>10	12.14
Kinase	Binding	VEGFR2	>10	0.11
Non-Kinase Enzymes	Enzymatic	AChE	>10	0
Non-Kinase Enzymes	Enzymatic	COX1	>10	0
Non-Kinase Enzymes	Enzymatic	COX2	>10	2.38
Non-Kinase Enzymes	Enzymatic	MAOA	>10	0
Non-Kinase Enzymes	Enzymatic	PDE3A	>10	0
Non-Kinase Enzymes	Enzymatic	PDE4D2	>10	5.43
Transporter	Blocker	DAT	>10	0
Transporter	Blocker	NET	>10	6.01
Transporter	Blocker	SERT	>10	2.41

Table S11: Eurofins SAFETYscan 47 E/IC50 ELECT panel reference controls

Compound Name	Target Class	Mode	Assay Target	Result Type	RC50 (μM)

NECA	GPCR	Agonist	ADORA2A	EC50	0.01714
SCH 442416	GPCR	Antagonist	ADORA2A	IC50	0.07938
A 61603 Hydrobromide	GPCR	Agonist	ADRA1A	EC50	0.00011
Tamsulosin	GPCR	Antagonist	ADRA1A	IC50	0.00117
UK 14304	GPCR	Agonist	ADRA2A	EC50	0.00003
Yohimbine	GPCR	Antagonist	ADRA2A	IC50	0.00447
(−)-Isoproterenol	GPCR	Agonist	ADRB1	EC50	0.00092
Betaxolol	GPCR	Antagonist	ADRB1	IC50	0.00602
(−)-Isoproterenol	GPCR	Agonist	ADRB2	EC50	0.00097
ICI 118,551 hydrochloride	GPCR	Antagonist	ADRB2	IC50	0.0009
[Arg8]-Vasopressin	GPCR	Agonist	AVPR1A	EC50	0.00116
SR 49059	GPCR	Antagonist	AVPR1A	IC50	0.00123
CCK fragment 26-33 Amide	GPCR	Agonist	CCKAR	EC50	0.00006
SR 27897	GPCR	Antagonist	CCKAR	IC50	0.0487
Acetylcholine chloride	GPCR	Agonist	CHRM1	EC50	0.01302
Atropine	GPCR	Antagonist	CHRM1	IC50	0.00904
Acetylcholine chloride	GPCR	Agonist	CHRM2	EC50	0.04167
Atropine	GPCR	Antagonist	CHRM2	IC50	0.00138
Acetylcholine chloride	GPCR	Agonist	CHRM3	EC50	0.01651
Atropine	GPCR	Antagonist	CHRM3	IC50	0.0037
CP 55940	GPCR	Agonist	CNR1	EC50	0.00002
AM 251	GPCR	Antagonist	CNR1	IC50	0.00083
CP 55940	GPCR	Agonist	CNR2	EC50	0.00017
SR 144528	GPCR	Antagonist	CNR2	IC50	0.0455
Dopamine	GPCR	Agonist	DRD1	EC50	0.05179
SCH 39166	GPCR	Antagonist	DRD1	IC50	0.0013
Dopamine	GPCR	Agonist	DRD2S	EC50	0.00192
Risperidone	GPCR	Antagonist	DRD2S	IC50	0.0011
Endothelin 1	GPCR	Agonist	EDNRA	EC50	0.00239
BMS 182874	GPCR	Antagonist	EDNRA	IC50	0.18769
Histamine	GPCR	Agonist	HRH1	EC50	0.01426
Mepyramine	GPCR	Antagonist	HRH1	IC50	0.00849

Histamine	GPCR	Agonist	HRH2	EC50	0.33478
Tiotidine	GPCR	Antagonist	HRH2	IC50	0.08408
Serotonin Hydrochloride	GPCR	Agonist	HTR1A	EC50	0.00311
Spiperone	GPCR	Antagonist	HTR1A	IC50	0.03764
Serotonin Hydrochloride	GPCR	Agonist	HTR1B	EC50	0.00002
SB 224289	GPCR	Antagonist	HTR1B	IC50	0.00883
Serotonin Hydrochloride	GPCR	Agonist	HTR2A	EC50	0.00442
Altanserin	GPCR	Antagonist	HTR2A	IC50	0.01944
Serotonin Hydrochloride	GPCR	Agonist	HTR2B	EC50	0.00193
LY 272015	GPCR	Antagonist	HTR2B	IC50	0.00082
DADLE	GPCR	Agonist	OPRD1	EC50	0.00005
Naltriben	GPCR	Antagonist	OPRD1	IC50	0.00058
Dynorphin A (1-17)	GPCR	Agonist	OPRK1	EC50	0.01118
nor-Binaltorphimine	GPCR	Antagonist	OPRK1	IC50	0.00136
DAMGO	GPCR	Agonist	OPRM1	EC50	0.00086
Naloxone	GPCR	Antagonist	OPRM1	IC50	0.00183
Isradipine	Ion Channel	Blocker	CAV1.2	IC50	0.01404
Picrotoxin	Ion Channel	Blocker	GABAA	IC50	2.67078
GABA	Ion Channel	Opener	GABAA	EC50	5.84033
Astemizole	Ion Channel	Blocker	hERG	IC50	0.23862
Bemesetron	Ion Channel	Blocker	HTR3A	IC50	0.00746
Serotonin Hydrochloride	Ion Channel	Opener	HTR3A	EC50	0.39975
XE 991	Ion Channel	Blocker	KvLQT1/minK	IC50	0.92793
ML-277	Ion Channel	Opener	KvLQT1/minK	EC50	2.65858
Dihydro-β-erythroidine	Ion Channel	Blocker	nAChR(a4/b2)	IC50	0.56808
(−)-Nicotine	Ion Channel	Opener	nAChR(a4/b2)	EC50	1.64972

Lidocaine Hydrochloride	Ion Channel	Blocker	NAV1.5	IC50	38.10624
(+)-MK 801	Ion Channel	Blocker	NMDAR(1A/2B)	IC50	0.08444
L-Glutamic Acid	Ion Channel	Opener	NMDAR(1A/2B)	EC50	0.45497
BMS-754807	Kinases	Inhibitor	INSR	IC50	0.00036
Gleevec	Kinases	Inhibitor	LCK	IC50	0.01403
Staurosporine	Kinases	Inhibitor	ROCK1	IC50	0.00055
SU-11248	Kinases	Inhibitor	VEGFR2	IC50	0.00013
BMS-564929	NHR	Agonist	AR	EC50	0.0012
Geldanamycin	NHR	Antagonist	AR	IC50	0.04035
Dexamethasone	NHR	Agonist	GR	EC50	0.03359
Mifepristone	NHR	Antagonist	GR	IC50	0.06996
Physostigmine	Non-Kinase Enzymes	Inhibitor	AChE	IC50	0.0405
Indomethacin	Non-Kinase Enzymes	Inhibitor	COX1	IC50	0.05316
NS-398	Non-Kinase Enzymes	Inhibitor	COX2	IC50	0.38773
Clorgyline	Non-Kinase Enzymes	Inhibitor	MAOA	IC50	0.00142
Cilostamide	Non-Kinase Enzymes	Inhibitor	PDE3A	IC50	0.02297
Cilomilast	Non-Kinase Enzymes	Inhibitor	PDE4D2	IC50	0.0103
GBR 12909	Transporter	Blocker	DAT	IC50	0.00142
Desipramine	Transporter	Blocker	NET	IC50	0.01395
Clomipramine	Transporter	Blocker	SERT	IC50	0.00145

Pharmaron Cytochrome P450 inhibition assay protocols and results

Cytochrome P450 (CYP) direct and time dependent inhibition (TDI) of Compound 33 was evaluated on seven major CYP enzymes using pooled human liver microsomes. The study included three incubation conditions: no pre-incubation, 30-minute pre-incubation with NADPH, and 30-minute pre-incubation without NADPH, to distinguish between direct and time-dependent inhibition. Each test compound was evaluated across a range of concentrations, and

reactions were initiated by adding NADPH and specific substrates for each CYP isoform. The reactions were terminated with cold methanol containing internal standards, followed by centrifugation and LC-MS/MS analysis. The IC_{50} values were calculated using GraphPad Prism to assess inhibitory potency and potential metabolic liabilities.

The bioanalytical method employed a Shimadzu LC system coupled with AB Sciex QTRAP® 4500 or Triple Quad™ 5500 mass spectrometers using electrospray ionization (ESI) in multiple reaction monitoring (MRM) mode. Chromatographic separation was achieved using an Xselect® HSS T3 column with a gradient of 0.1% formic acid in water and acetonitrile. Specific elution programs were optimized for each metabolite, such as acetaminophen, hydroxybupropion, and dextrorphan. The MS parameters, including ion spray voltage, gas flows, and collision energies, were tailored for each CYP substrate-metabolite pair to ensure sensitive and selective detection. This robust LC-MS/MS method enabled accurate quantification of metabolite formation, which was critical for determining the inhibitory effects of the test compounds.

Table S12: Pharmaron Cytochrome P450 inhibition assay results of Compound 33 at concentration up to 25 µM

CYP Isoform	Substrate	IC_{50} (µM)		
		0-min pre-incubation	30-minutes pre-incubation with NADPH	30-minutes pre-incubation without NADPH
CYP1A2	Phenacetin	> 25	> 25	> 25
CYP2B6	Bupropion	> 25	> 25	> 25
CYP2C8	Paclitaxel	7.757	13.55	9.419
CYP2C9	Diclofenac	> 25	> 25	> 25
CYP2C19	(S)-Methenytoin	10.96	18.89	13.95
CYP2D6	Dextromethorphan	1.336	2.154	1.322
CYP3A4/5	Midazolam	1.14	0.66	1.10
CYP3A4/5	Testosterone	7.46	2.22	8.58

Table S13: Pharmaron Cytochrome P450 inhibition results with reference compounds

CYP Isoform	Reference Compound	Substrate	IC_{50} (µM)		
			0-min pre-incubation	30-minutes pre-incubation with NADPH	30-minutes pre-incubation without NADPH
CYP1A2	Furafylline	Phenacetin	2.55	0.42	2.62
CYP2B6	Thiotepa	Bupropion	28.26	3.49	33.62
CYP2C8	Gemfibrozil Glucuronide	Paclitaxel	28.26	3.49	33.62
CYP2C9	Ticrynafen	Diclofenac	1.17	0.19	1.44
CYP2C19	Fluoxetine	(S)-Methenytoin	14.02	1.45	14.28
CYP2D6	Paroxetine	Dextromethorphan	0.99	0.10	1.21
CYP3A4/5	Mifepristone	Midazolam	5.17	0.30	7.11
CYP3A4/5	Mifepristone	Testosterone	6.82	0.36	6.82

References

1. Bockus, A. T.; Leung, S. S. F.; Fraga-Walton, B.; Baldomero, M. P.; Hernandez, L.; Dupper, N. J.; Shapiro, J. A.; Lent, B. M.; Spellmeyer, D. C.; DeMart, M. K.; Luna, J.; Hoang, D.; Chand, M.; Gritsenko, Y.; Ramaseshan, M.; Gleason, C. E.; Hamkins-Indik, F.; Membreno, M. W.; Zheng, J.; Odeh, R.; Nosrati, M.; He, D.; Situ, G.; Bambal, R.; Cremin, P.; Fang, J.; Levin, B.; Wang, E. W.; Evangelista, M.; Earp, D. J.; Kreatsoulas, C.; Singh, R.; Garcia, P. D.; Aggen, J. B. Discovery of Cell-Permeable Macrocyclic Cyclin A/B RxL In-hibitors that Demonstrate Anti-Tumor Activity in E2F-driven Cancers. In Press, Journal of Medicinal Chemistry **2025**.
2. Andrew T. Bockus, Sik Fai Siegfried Leung, David J. Earp, Pablo Santiago Garcia Dominguez, David C. Spellmeyer, Luis Hernandez, Miguel Paolo Baldomero, Catherine E. Gleason, Breena F. Walton, Rajinder Singh, James B. Aggen, Nathan J. Dupper, Justin A. Shapiro, Constantine Kreatsoulas, Ramesh B. Bambal, Chat Cheong Gabriel Fung, Mahesh Ramaseshan, "Preparation of cyclic peptidic compounds as cyclin inhibitors for treating cancers" US20240218021 (WO2024086814 A2)

3. Andrew T. Bockus, Breena F. Walton, Constantine Kreatsoulas, James B. Aggen, Justin A. Shapiro, Megan DeMart, Nathan J. Dupper, Sik Fai Siegfried Leung, Chinmay Bhatt, Samuel Metobo, Kai Yang, Ming Hsun-Ho, Rajinder Singh, Ramesh Bambal "Cyclin Inhibitors" US2024243010 (WO2024243010 A1)
